# Whole-genome sequencing reveals mutational landscape underlying phenotypic differences between two widespread Chinese cattle breeds

**DOI:** 10.1371/journal.pone.0183921

**Published:** 2017-08-25

**Authors:** Yao Xu, Yu Jiang, Tao Shi, Hanfang Cai, Xianyong Lan, Xin Zhao, Martin Plath, Hong Chen

**Affiliations:** 1 College of Animal Science and Technology, Northwest A & F University, Shaanxi Key Laboratory of Molecular Biology for Agriculture, Yangling, Shaanxi, China; 2 Institute of Biology and Medicine, College of Life Science and Health, Wuhan University of Science and Technology, Wuhan, Hubei, China; Wageningen UR Livestock Research, NETHERLANDS

## Abstract

Whole-genome sequencing provides a powerful tool to obtain more genetic variability that could produce a range of benefits for cattle breeding industry. Nanyang (*Bos indicus*) and Qinchuan (*Bos taurus*) are two important Chinese indigenous cattle breeds with distinct phenotypes. To identify the genetic characteristics responsible for variation in phenotypes between the two breeds, in the present study, we for the first time sequenced the genomes of four Nanyang and four Qinchuan cattle with 10 to 12 fold on average of 97.86% and 98.98% coverage of genomes, respectively. Comparison with the Bos_taurus_UMD_3.1 reference assembly yielded 9,010,096 SNPs for Nanyang, and 6,965,062 for Qinchuan cattle, 51% and 29% of which were novel SNPs, respectively. A total of 154,934 and 115,032 small indels (1 to 3 bp) were found in the Nanyang and Qinchuan genomes, respectively. The SNP and indel distribution revealed that Nanyang showed a genetically high diversity as compared to Qinchuan cattle. Furthermore, a total of 2,907 putative cases of copy number variation (CNV) were identified by aligning Nanyang to Qinchuan genome, 783 of which (27%) encompassed the coding regions of 495 functional genes. The gene ontology (GO) analysis revealed that many CNV genes were enriched in the immune system and environment adaptability. Among several CNV genes related to lipid transport and fat metabolism, Lepin receptor gene (*LEPR*) overlapping with CNV_1815 showed remarkably higher copy number in Qinchuan than Nanyang (log_2_ (ratio) = -2.34988; *P* value = 1.53E-102). Further qPCR and association analysis investigated that the copy number of the *LEPR* gene presented positive correlations with transcriptional expression and phenotypic traits, suggesting the *LEPR* CNV may contribute to the higher fat deposition in muscles of Qinchuan cattle. Our findings provide evidence that the distinct phenotypes of Nanyang and Qinchuan breeds may be due to the different genetic variations including SNPs, indels and CNV.

## Introduction

Integrating phenotypic variability with genomic variation is pivotal in both fundamental and applied biological sciences[1,2]. Most phenotypic traits, including naturally selected traits in wild populations[3] and those under artificial selection in domesticated animals[4], show quantitative genetic inheritance, and have complex genetic architectures[5,6]. Considerable progress has been made through high throughput sequencing to obtain whole genome sequences, which offer extensively promising efficient approaches for screening the molecular targets of selection and speciation[7,8].

Cattle are important farm animals and provide a major source of protein and fat for human populations worldwide, and thus variable traits related to the growth, carcass and meat quality are of vital interest in large-scale breeding projects of cattle[9]. Since the bovine genome was firstly sequenced from an inbred Hereford cow and her sire by capillary sequencing[10], the researches of resequencing and assembling genomes from different cattle breeds have quickly progressed. The whole-genome resequencing was firstly performed in a single Germany Fleckvieh bull, and 2.44 million single nucleotide polymorphisms (SNPs) and 115,000 small indels were identified by aligning to the reference assembly genome[11]. Kawahara-Miki et al.[12] resequenced the whole genome of a Japanese *Kuchinoshima-Ushi* cattle and revealed a total of 11,713 non-synonymous SNPs in protein-coding regions of 4,643 genes, and further phylogenetic analysis showed that the genetic background of *Kuchinoshima-Ushi* is quite distinct from the previously sequenced European domestic cattle breeds. The whole genome of a Korean Hanwoo bull was sequenced at a high coverage (45.6-fold), and 25 genes associated with meat quality and disease resistance were determined in the homozygous regions[13]. Moreover, gene copy number variations (CNVs) have been identified systematically at genome-wide level in cattle. Bickhart et al.[14] reported 1,265 CNV regions in six individuals from four American cattle breeds using high throughput sequencing. Additionally, the genome resequencing of two North American breeds, Black Angus and Holstein, revealed 790 putative CNV regions, which could be considered to be promising genetic markers for the identification across the genomes[15].

Even though genomic variability of traditional breeds is of vital interest from an agro-economic perspective as their traits could be desirable in primary breeding programs[2], we are still far from having a comprehensive understanding of genomic variations of different cattle breeds worldwide. For example, whole genome of the most widespread Chinese native cattle breeds has not been sequenced. Therefore, our study was designed to select a maximally informative subset of genomic markers for complex traits of agro-economic importance Similar to the case of natural selection, allele frequencies of loci underlying traits are expected to increase during the domestication process[16]. Therefore, comparing the whole genome sequences from distinct populations or breeds may contribute to identify the causative loci that affect phenotypic traits shaped by man-made selection.

We focused on two phenotypically distinct cattle breeds, Nanyang and Qinchuan, which belong to the native thoroughbred stock in China. Nanyang breed mainly originated from *Bos indicus* and was firstly domesticated in 1950. Qinchuan breed, known as *Bos taurus* in 6000~7000 B.C., and until 126 B.C. a little bloodline of *Bos indicus* was introduced into the breed and formed the domestic Qinchuan cattle. In general, Nanyang breed is characterized as a high acromion and narrow hindquarters, and have an average 380 kg of body weight for adult individuals, while Qinchuan has a much larger body weight (approximately 600 kg for adult male and 400 kg for female) and a typical thriving dewlap. In addition, as a well-known beef cattle breed in China, Qinchuan have higher meat production and quality than Nanyang due to its high marbling levels. We hypothesized that the phenotypic variability may be caused by different genetic background between the two breeds. However, the whole genomic information of Nanyang and Qinchuan are unavailable so far, and comparative analyses of SNPs, indels and CNVs have not been feasible. In this study, we firstly sequenced the whole genomes of Nanyang and Qinchuan cattle by high throughput sequencing, and the results not only provide novel insight into the genetic difference at a whole-genome scale but also identify genomic loci that may be of vital interest in the programs for cattle breeding.

## Materials and methods

### Ethics statement

This study was approved by the Review Committee for the Use of Animal Subjects of Northwest A&F University. All animal experiments were carried out in strict accordance with institutional and state guidelines for animal care and all efforts were made to minimize suffering.

### Sample preparation

Two famous Chinese cattle breeds (Nanyang and Qinchuan) were selected in our sequencing strategy. Blood samples (5 mL of each containing 20 U/mL heparin) were obtained from four Nanyang bulls and four Qinchuan bulls, which aged 4 years old and reared in the elite reservation farms from the Henan and Shaanxi province, respectively. All cattle were raised on a corn-corn silage diet. Genomic DNA (gDNA) was extracted from the whole blood with a QIAamp DNA Blood Maxi Kit (QIAgen, Valencia, CA, USA) according to manufacturer’s instructions.

The adult (4 years old) Qinchuan cattle were slaughtered for tissue sampling in a commercial slaughterhouse located in the city of Yangling, under the supervision of the Animal Ethics Committee (AEC) and Northwest A&F University. Samples (fat, skeletal muscle, spleen, kidney, intestine, liver, heart, and lung) were immediately submerged in liquid nitrogen within 25 minutes of slaughter. In addition, fat from 16 cattle and skeletal muscle from 20 cattle were collected for total RNA and gDNA isolation. Total RNA was isolated with Trizol reagent (Takara, Liaoning, China) according to the manufacturer’s protocol. The gDNA was also extracted from the fat and skeletal muscle (10 mg) by two rounds of proteinase K digestion and phenol-chloroform extraction. Further association analysis of CNV locus with phenotypic traits were conducted in a total of 191 Qinchuan cattle, and the traits were including body weight, body height, body length, heart girth, chest width, chest depth, height at hip cross, hucklebone width, hip width and rump length. Blood samples were collected and gDNA was isolated according to the procedures.

### Preparation of fragment libraries and high-throughput sequencing

Libraries were prepared according to Illumina protocols. Each gDNA was sheared, polished and purified using minor modifications of the original Illumina Sample Preparation kit (Illumina 2006). Briefly, 10 μg gDNA was fragmented by nebulization for 5 min at a pressure of 32 psi N_2_, and the sheared fragments were purified and concentrated using a QIAquick PCR purification spin column. To repair damaged gDNA ends and obtain 5’-phosphorylated blunt-ends (5’P), the fragments were end-repaired with T4 DNA polymerase, T4 phosphonucleotide kinase (PNK) and the Klenow fragment of *Escherichia coli* DNA polymerase. Terminal (3’) A-residues were added following a brief incubation with dATP and Klenow 3’-5’exo-. Fragments were then ligated with solexa adaptors provided by the manufacturer. Adaptor-ligated fragments in the range of ~150–300 bp were selected using agarose electrophoresis. These small insert libraries were amplified independently using 18 rounds of PCR amplification and standard primers PE 1.0 and PE 2.0 supplied by Illumina. After spin column extraction and quantitation, libraries were mixed (multiplex) at equimolar ratios to yield a total oligonucleotide mix of 10 nM.

Aliquots of multiplex libraries (5 pmol) were loaded onto the cluster generation station for single molecule bridge amplification on slides containing attached primers. The slide with amplified clusters was then subjected to step-wise sequencing using four-color labeled nucleotides on the Illumina 1G sequencing system for 36 cycles, which produces a theoretical fixed read length of 36 bp.

### Sequence alignment and mapping

Paired-end sequence reads from Nanyang and Qinchuan were mapped against bovine genome assembly Bos_taurus_UMD_3.1, which was downloaded from the NCBI database. In this study, sequence scaffolds not yet assigned to specific chromosomes were excluded and no repeat masker was applied to the assembly.

The BWA algorithm ver. 0.5.0 (http://bio-bwa.sourceforge.net/bwa.shtml) was used for sequence alignment and consensus assembly. To obtain reliable alignment hits, the main parameters were defined for mapping, for instance, two mismatches were allowed between the read and the reference (mismatch penalty, misMsc = 2) when the sequence length < 60 bp, while it would be three mismatches if the sequence length > 60 bp. The sequence reads were not aligned with the inserting gaps, thus the parameter for read trimming (trimQual) was designated as 0. Moreover, the remaining indices were set according to the BWA default values. After read mapping, we discarded the reads mapped to multiple chromosomal positions and unmapped reads. Only reads with unique ungapped alignment were used for consensus calling and SNP detection.

### SNP identification and annotation

Alignments of the reads from the Nanyang and Qinchuan were processed using SAMtools (http://samtools.sourceforge.net/samtools.shtml) to filter and report high quality SNP positions. SNP detection was performed using SAMtools ‘pileup’ command at default settings appropriate for diploid organisms. SNP filtering was performed in the following restricted conditions. The low-quality data were discarded (five bases with Q score < 20), and a minimum of 2× coverage depth (3× for the heterozygosity) was allowed for the initial identification of putative SNPs using SAMtools ‘varFilter’ command specifying fairly permissive minimum quality cutoffs. In addition, the heterozygous and homozygous SNPs were distinguished using an 80% cutoff of percent aligned reads calling the SNP. Consensus sequences for the SNP positions were generated using another tool from which SAMtools (and BWA) inherited code: MAQ[17].

The SNPs annotations were based on the 313,678 Bos taurus RefSeq in NCBI database. The cattle RefSeqs were aligned against Bos_taurus_UMD_3.1 using BLAT with the ‘fine’ option to obtain the genomic positions of genes, introns, and coding regions. In total, 63,213 RefSeqs were aligned against the reference genome. Among the aligned RefSeqs, the sequences with >90% coverage and a <1% error rate were selected. The selected genes were used to predefine the annotation data of all possible variants and pre-calculate the SIFT [18] predictions and scores. We selected the non-synonymous and frame shift (NS/F) that showed SIFT scores of <0.05 as the potentially damaging mutations.

### Small indels and CNVs identification

A list of putative indels was generated for the two breeds from the paired-end reads, by combining the analysis of the algorithm BreakDancer [19] (http://breakdancer.sourceforge.net/breakdancermax.html) and the Pindel [20] program (http://trac.nbic.nl/pindel/), which computes the precise break points as well as the fragments inserted or deleted compared to the reference genome. In the preprocessing step, the BWA was first applied for mapping all the reads to the reference genome, and then the aligned results were examined to keep those paired reads that only one end can be mapped. For each paired read, the mapped end must be uniquely located in the genome without mismatch bases while the other end cannot be mapped to anywhere in the genome under a given threshold alignment score (20 to 36 bp reads). In the present study, for identifying small indels, the lenghth of 1 to 3 bp indels were obtained by setting relevant parameters.

The CNVs calls on the cattle genomes were identified using the CNV-seq program. Briefly, the Nanyang and Qinchuan reads were mapped to the Bos_taurus_UMD_3.1 using the BWA, and the output of BAM was converted into the "best-hit" format required by CNV-seq using the best-hit.Solexa.pl Perl script. Four consecutive sliding windows exhibiting a significant difference of read depth (minimum-windows-required = 4) enabled us to classify the region as a CNV. The Perl script was run with the default threshold values (*P*-value = 0.001 and log_2_ threshold = 1) and a window size setting of 2, to generate the putative CNVs from the best-hit files.

### Quantitative PCR validation

Quantitative real-time PCR (qPCR) was performed to validate the CNVs from the whole genomes of Nanyang and Qinchuan cattle. Primers were designed *in silico* for 10 genic and 10 non-genic CNVs (S7 Table). Amplification efficiency for all primers was measured with different dilutions of gDNA (0.005 ng, 0.05 ng, 0.5 ng, 5 ng, 50 ng, 500 ng). Correlation coefficients (*R*^2^), derived from linear regressions, ranged between 0.951 and 0.990, whereas the slope was between -3.164 and -3.842, indicating nearly 100% PCR efficiency for all the loci. The qPCR experiments were conducted in triplicate reactions, and each with a reaction volume of 20 μl containing 100 ng of gDNA, 10 μl SYBR^®^ Premix Ex Taq TM II (Takara, Liaoning, China), and 10 pmol of primers. All reactions were amplified on a Bio-Rad CFX 96^™^ Real Time Detection System (Bio-Rad, Hercules, CA, USA). Gene relative expression was normalized to the expression of bovine glyceraldehyde-3-phosphate dehydrogenase (*GAPDH*) gene. Accordingly, the *LEPR* gene expression levels were quantified using Gene Expression Macro software (Applied Biosystems) by employing an optimized comparative Ct (ΔΔCt) value method, commonly designated as 2^-ΔΔCt^. In addition, the bovine *BTF3* gene was chosen as the diploid internal reference gene for the genomic qPCR analysis, and the ΔCt for each sample was calculated normalizing to *BTF3*[21], In addition, the copy number was confirmed based on the assumption that there were two copies of the DNA segment in the calibrator animals.

### CNV annotation and gene ontology analysis

Gene content of cattle CNVs was assessed using Ensembl genes (ftp://ftp.ensembl.org/pub/current_fasta/bos_taurus/pep/). A total of 47,100 bovine peptides were retrieved from the Ensembl (Bos_taurus.UMD3.1.75.pep.abinitio). The canonical transcript record for each CNV gene was used to obtain a specific Ensembl protein ID, and the GO terms associated with the overlapping genes were analyzed using the agriGO server's Singular Enrichment Analysis (SEA) tool [22]. The significance of term enrichments were under or overrepresented in CNVs after Bonferroni correction.

### Association analysis

SPSS v20.0 software (SPSS, Chicago, IL, USA) was used to analyze the associations of *LEPR* CNV with phenotypic traits in Qinchuan cattle by the One-way ANOVA method, and the relative copy number of *LEPR* was fitted as a continuous variable. Effects associated with farm, sex and season of birth (spring versus fall) were not into linear model, as the preliminary statistical analyses indicated that these effects did not have significant influence on variability of traits in the tested breeds. Thus the following model is used: *Y*_*ijk*_ = *μ* + *A*_*i*_ + *CNV*_*j*_ + *e*_*ijk*_, where *Y*_*ijk*_ is the observation of the growth traits; *μ* is the overall mean of each trait, *A*_*i*_ is the effect due to *i*th age, *CNV*_*j*_ is the fixed effect of *j*th CNVs type of *LEPR* gene and *e*_*ijk*_ is the random residual error.

## Results

### Whole-genome sequencing and mapping

We used Illumina technology to sequence gDNA from four Nanyang and four Qinchuan bulls, which were covered with an average mapping depth of 10 to 12 fold, respectively (Table 1). In total, approximately 333,930,957 reads (149,589,163 for Nanyang and 184,341,794 for Qinchuan) comprising 67.45 Gbp were generated, and values of quality ≥ 20 (Q_20_) reached 100% (S1 Table). The obtained reads were mapped to the reference sequence (Bos_taurus_UMD_3.1) using the BWA algorithm[17], 96% of reads were successfully mapped to unique positions on the reference genome. On average, 97.86% (Nanyang) and 98.98% (Qinchuan) of bovine chromosome sequences were covered in our present study (Table 1; S1 Fig). Raw sequencing data in this study has been deposited in GenBank SRA database under accession number of SRP049655.

**Table 1:** Average coverage of the Nanyang and Qinchuan genomes.

Genome	Total reads	Mapped (%)	Identity (%)	Depth	Coverage^a^ (%)
Nanyang	368,683,586	96.45%	99.40%	11.76	98.98%
Qinchuan	299,178,320	95.55%	99.24%	9.37	97.86%

^a^Fold coverage was calculated by aligning the assembling sequences to the reference chromosomes (Bos_taurus_UMD_3.1) used for mapping.

### SNP/Indel annotation and comparison of two cattle breeds

We used SAMtools[23] to identify putative SNPs while mapping the aligned reads to the reference assembly. SNPs with quality values < 20 and a sequencing depth < 3 were filtered and discarded, resulting in final sets of 9,010,096 and 6,965,062 SNPs for the genomes of Nanyang and Qinchuan, respectively. 28.59% of all SNPs were transversions and 71.41% were transitions; 34.02% were homozygous and 65.98% heterozygous, with a ratio of 1:1.94 (S2 Table). To assess the false negative rates of SNPs in both breeds, the SNP list was compared to the locus obtained using Sanger sequencing assay. For Nanyang, a set of 517 SNPs were selected and validated by sequencing, and of these, 464 (89.7%) were identified as SNPs. Based on these results we calculated the false positive rate for SNP detection in Nanyang as (1–464/517) * 100 = 10.3%. Additionally, the false positive rate for SNP validation of Qinchuan was calculated as (1–470/502) * 100 = 6.4%.

We compared the identified SNP sets with those already published in the cattle dbSNP database (Build 133; ftp://ftp.ncbi.nlm.nih.gov/snp/organisms/cow_9913/chr_rpts/). The SNPs of Nanyang had 4,429,836 (49.17%) positions overlapping with the cattle dbSNP database, whereas the Qinchuan shared 4,942,730 (70.98%) SNPs with the dbSNP database. Distributions of the remaining novel SNPs on each of the 30 chromosomes were depicted in S2 Fig. Using Pindel and BreakDancer, 154,934 and 115,032 small indels of 1 to 3 bp length were identified in the Nanyang and Qinchuan, respectively. The frequencies of three indel types (insertions, deletions, and insertions within deletions) in each chromosome were shown in S3 Table. Indels of 1 bp length prevail in frequency (S3 Fig), which was in accordance with a previous study by Kawahara-Miki et al. [12]. In addition, we found that the SNPs and small indels in each chromosome of Nanyang were more than that in Qinchuan (S4 Fig). All SNPs and indels in this study have been submitted to the dbSNP at GenBank under the handle NWAF_LMBA.

**Fig 1:**
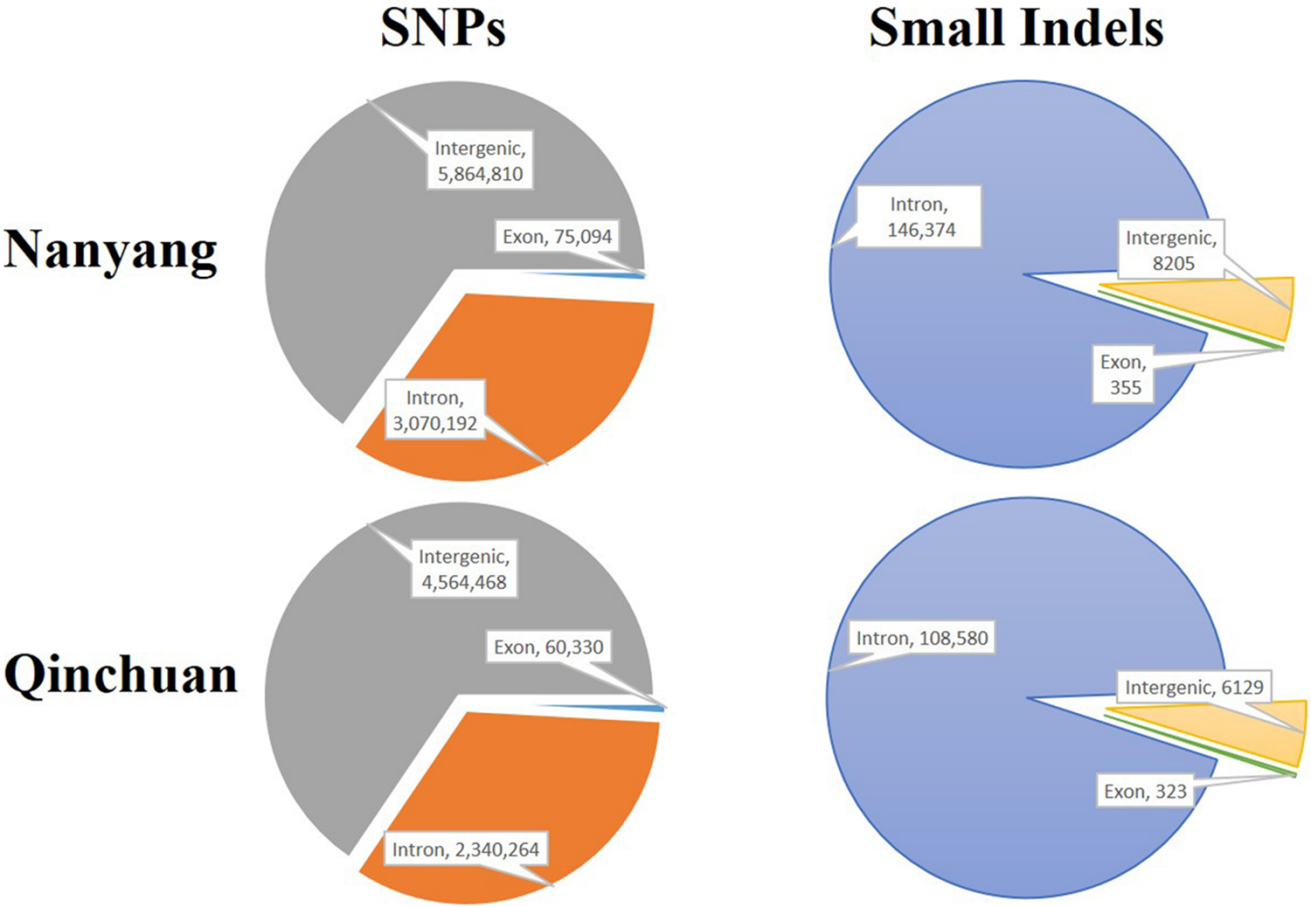
The number of SNPs and small indels distributed in the Nanyang and Qinchuan genomes (exon, intron, and intergenic).

All detected SNPs and indels in Nanyang and Qinchuan were annotated on the basis of the NCBI Reference Sequence Database (RefSeq: ref_Bos_taurus_UMD_ 3.1_ gnomon_top_level.gff3). Most of the SNPs and small indels were located in intergenic regions or introns, and only 0.84% were located in exons (Fig 1). Of these SNPs in coding regions of the Nanyang, 37,309 (49.68%) were non-synonymous substitutions, including missense and nonsense mutations, which were distributed in 11,712 functional genes. Additionally, a total of 355 small indels were located in coding regions, 239 of which (67.32%) were identified as variations that may cause gains or losses of stop codons by shifting the open reading frame (ORF); these occurred in 226 genes. By contrast, 30,389 non-synonymous SNPs (50.37%) were found in 10,991 genes in Qinchuan cattle, and 231 indels (71.52%) in 215 genes led to frame-shifts (S4 Table).

Non-synonymous SNPs, frame-shift, and indels (NS/FS/Indel) within a coding DNA sequence can affect the expression and function of important genes. Thus, a list of genes with more than 100 SNPs altogether, or more than 50 NS/FS/Indel, was compiled in Nanyang and Qinchuan genomes. Comparison between the two breeds revealed that the Nanyang genome had 32 NS/FS/Indel genes, while the Qinchuan had only 25 NS/FS/Indel genes. Among those genes, the obscurin-like 1 (*OBSL1*) gene, a novel cytoskeletal protein related to obscurin [24], showed more NS/FS/Indel in Nanyang than Qinchuan (102 *vs*. 78). The *OBSL1* gene has a larger genome span (22.091 kb) and transcript (6.046 kb) compared to several other functional genes. Importantly, two splice variants of the *OBSL1* gene (ENSBTAP00000053524 and ENSBTAT00000060992) were located on chromosome 2, and 42 mutations (NS/FS/Indel) in the two variants were recorded in Ensembl.

### Copy number variation and validation through qPCR

**Fig 2:**
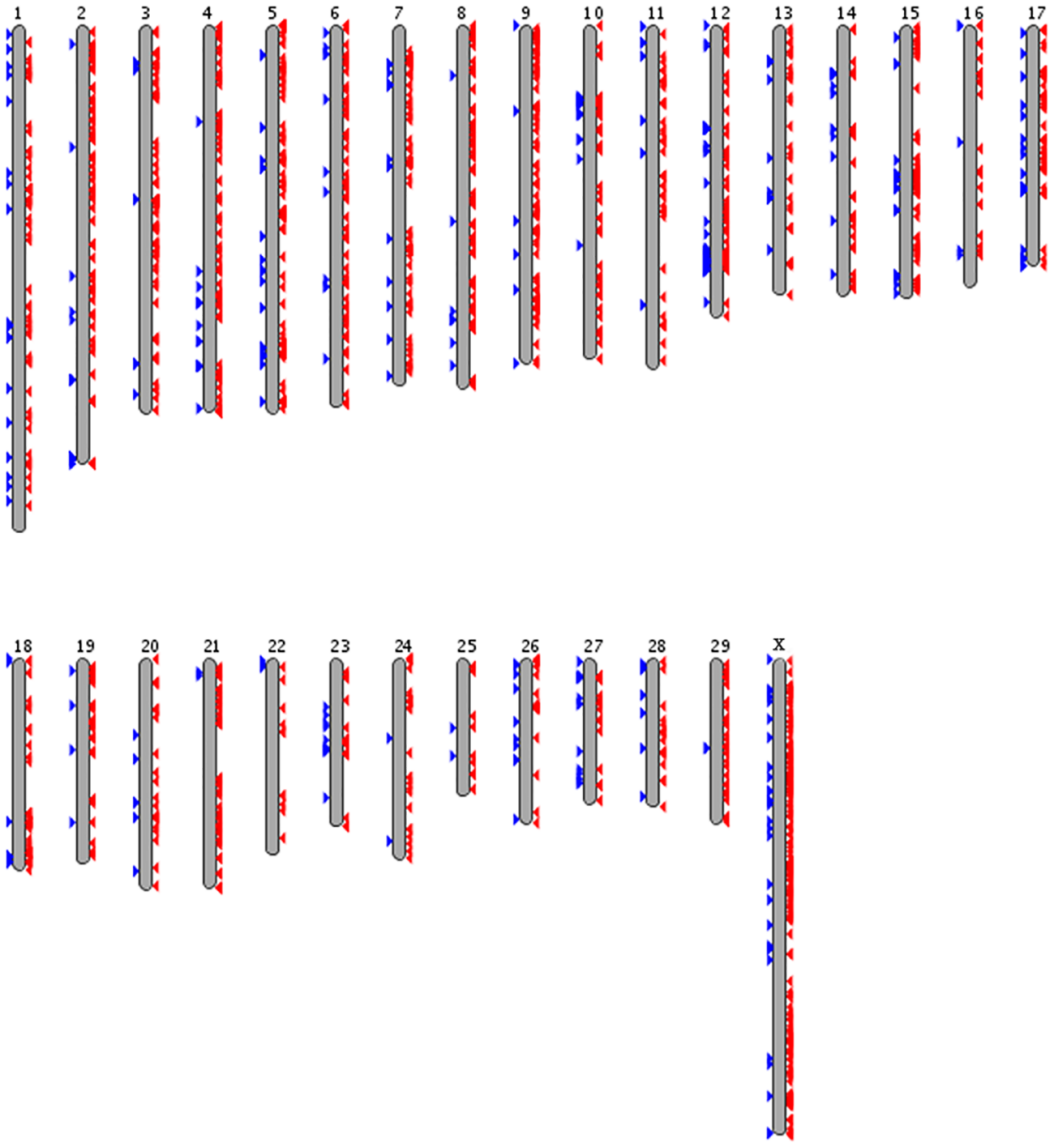
Schematic diagram of copy number variations (CNVs) regions in cattle genome. Blue arrowheads located on the chromosome ideograms represent copy number gains in Nanyang against with Qinchuan; while the red arrowheads represent copy number gains in Qinchuan against with Nanyang (Nanyang CNV losses). Note that several CNVs may combine and display as a single arrowhead due to their proximity in the genome.

Read sets of Nanyang and Qinchuan were conducted by aligning to the Hereford reference sequence assembly (Bos_taurus_UMD_3.1), while copy number variations (CNVs) were identified by compring the significantly different regions between the Nanyang and Qinchuan (control sample) mapped read sets. Overall, we detected 2,907 cases of CNV, amounting to approximately 9.9 Mbp and corresponding to 0.37% of the cattle genome (Table 2). The detected CNVs were unevenly distributed across chromosomes (Fig 2). Specifically, the percentage of CNVs numbers ranged from 0.413% to 5.917% on most other chromosomes, while 12.969% of CNVs (377) were located on chromosome 12, identifying chromosome 12 as the most polymorphic chromosome. The greatest number (449; 15.445%) of CNVs, however, was discovered on chromosome X, which is consistent with an earlier study [14]. Regions with CNVs varied in size between 1,776 bp and 158,952 bp, and the mean and median CNV length were 3,183 bp and 2,220 bp, respectively (Table 2). Detailed information on CNVs is provided in S5 Table.

**Table 2:** The detailed information of CNVs in our study.

Chr	Chromosome length	No. CNV	Total CNV length^a^	% length in CNV	% No. CNV	Max length	Mean length	Median length
1	158337067	99	277056	0.1750	3.406	10212	2799	2220
2	137060424	160	606060	0.4422	5.504	22644	3788	2664
3	121430405	110	376956	0.3104	3.784	48396	3427	2220
4	120829699	105	306360	0.2535	3.612	22644	2918	2220
5	121191424	172	808968	0.6675	5.917	158952	4703	2220
6	119458736	84	202908	0.1699	2.890	11988	2416	1776
7	112638659	113	393828	0.3496	3.887	24420	3485	2220
8	113384836	98	284160	0.2506	3.371	22200	2900	2220
9	105708250	113	317904	0.3007	3.887	12432	2813	2220
10	104305016	119	391164	0.3750	4.094	25308	3287	2220
11	107310763	47	121212	0.1130	1.617	9324	2579	2220
12	91163125	377	1499388	1.6447	12.969	52836	3977	2664
13	84240350	48	121212	0.1439	1.651	6216	2525	1998
14	84648390	49	127872	0.1511	1.686	10656	2610	1776
15	85296676	116	366300	0.4294	3.990	22200	3158	2220
16	81724687	37	103008	0.1260	1.273	22644	2784	1776
17	75158596	90	291708	0.3881	3.096	13764	3241	2220
18	66004023	89	267732	0.4056	3.062	10212	3008	2220
19	64057457	47	188700	0.2946	1.617	25752	4015	2220
20	72042655	28	68820	0.0955	0.963	4884	2458	1998
21	71599096	66	196692	0.2747	2.270	14652	2980	2220
22	61435874	14	33300	0.0542	0.482	4440	2379	2220
23	52530062	54	182928	0.3482	1.858	9768	3388	2664
24	62714930	29	147408	0.2350	0.998	64380	5083	2220
25	42904170	12	31524	0.0735	0.413	7548	2627	1998
26	51681464	29	88800	0.1718	0.998	17760	3062	2220
27	45407902	48	154956	0.3413	1.651	11988	3228	2220
28	46312546	24	55056	0.1189	0.826	5328	2294	1776
29	51505224	81	327228	0.6353	2.786	35076	4040	2220
X	148823899	449	1582860	1.0636	15.445	47952	3525	2220
Total	2660906405	2907	9922068	0.0037	-	158952	3183	2220

^a^The distribution and size characteristics of CNVs detected through comparison of the read sets mapped to the Bos_taurus_UMD_3.1 reference assembly.

**Fig 3:**
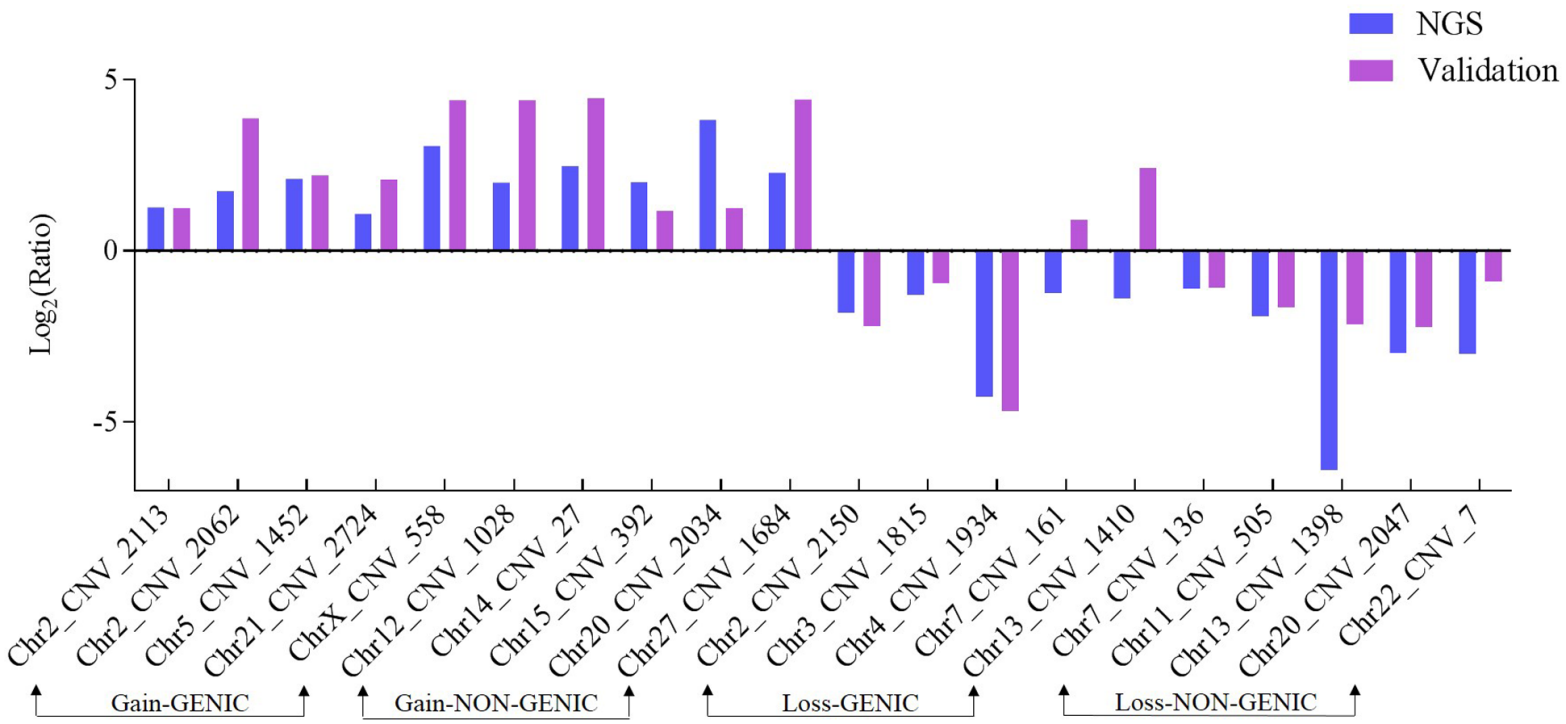
Validation of copy number variations (CNVs) detected from whole-genome resequencing using quantitative PCR. Validation results are shown for four CNVs groups: gain in genic, gain in non-genic, loss in genic, and loss in non-genic. Values on y-axis represented the log_2_ (2^Sample signal) for resequencing and log_2_ 2^-ΔΔCt^ for qPCR validation, respectively. The two values located in the same side of the "0" line represented the same CNV types, while the values located in two side represented the different CNV types.

To confirm the accuracy of CNV assessment from genome sequencing, quantitative PCR (qPCR) was conducted for a randomly selected subset of 20 CNVs, which were divided into two groups (10 genic and 10 non-genic CNVs). Both groups contained five gains (log_2__Ratio > 0.5) and five losses (log_2__Ratio < -0.5) of CNV status (Table 3). 90% of our qPCR results (i.e., 18 of 20 validated) agreed with the CNV predictions in these regions, and only CNVR_161 and CNVR_1410, harboring the *SSBP2* and *SNTA1* genes, respectively, were not congruent with the predictions from out genome sequencing results (Fig 3).

**Table 3:** The log_2_ ratio and *P*-value of CNVs selected for qPCR validation.

CNV position	Ensembl gene	CNV type	log_2_ ratio^a^	*P*-value^a^
Chr2_CNV_2113	ACTL8	gain	1.290	4.01E-87
Chr2_CNV_2062	RAPH1	gain	1.750	1.17E-45
Chr5_CNV_1452	LOC617219	gain	2.106	1.94E-112
Chr21_CNV_2724	GABRG3	gain	1.087	1.66E-20
ChrX_CNV_558	IL1RAPL2	gain	3.060	8.08E-59
Chr12_CNV_1028	-	gain	1.992	1.87E-39
Chr14_CNV_27	-	gain	2.484	2.52E-86
Chr15_CNV_392	-	gain	2.018	4.19E-43
Chr20_CNV_2034	-	gain	3.822	8.51E-184
Chr27_CNV_1684	-	gain	2.283	4.47E-168
Chr2_CNV_2150	COL5A2	loss	-1.278	1.88E-20
Chr3_CNV_1815	LEPR	loss	-2.350	1.53E-102
Chr4_CNV_1934	SHH	loss	-1.720	9.08E-45
Chr7_CNV_161	SSBP2	loss	-1.228	2.15E-32
Chr13_CNV_1410	SNTA1	loss	-1.393	4.28E-44
Chr7_CNV_136	-	loss	-1.108	4.69E-41
Chr11_CNV_505	-	loss	-1.907	2.89E-39
Chr13_CNV_1398	-	loss	-6.409	1.21E-101
Chr20_CNV_2047	-	loss	-2.976	1.18E-70
Chr22_CNV_7	-	loss	-3.015	4.01E-70

^a^The log_2_ ratio and *P*-value were obtained from the CNV-seq software. Positive log_2_ ratios represented that the copy number in Nanyang was higher than the Qinchuan, while the negative values indicated higher copy number in Qinchuan than Nanyang.

To further evaluate CNV between breeds, the same 20 CNV regions were quantified in 10 Nanyang and 10 Qinchuan individuals. Relative copy numbers (CNs) were calculated based on the assumption that there were two copies of each DNA segment in the calibrator (the Qinchuan sequenced in our initial CNV survey). As illustrated in S5 Fig, average copy numbers of Nanyang were larger than those of Qinchuan breed in the group of CNV gains. In contrast, mean copy numbers of Nanyang were always lower than those of Qinchuan in the case of CNV losses.

### Gene content and gene ontologies of cattle CNV regions

To identify potential functional roles associated with the putative CNVs, genes completely or partially overlapping with these CNVs were retrieved from Ensembl[25]. 27% (783/2907) of all CNVs involved 495 partially or entirely functional genes. Gene ontology (GO), a standardized gene classification system[26], was performed to determine the likely biological effects of CNV genes using the web-based tool agriGO [27]. Statistically significant over-representations were observed for multiple categories (Table 4), which showed that the GO terms “G-protein coupled receptor signaling pathway” (GO:0007186; *P*<0.01), “immune system process” (GO:0002376; *P*<0.01), “Olfactory receptor activity” (GO:0004984; *P*<0.01), “Integral to membrane” (GO:0016021; *P*<0.01), were enriched among the CNV genes in the Nanyang. The analyzed CNV genes, including ATP-binding cassette A13 (*ABCA13*), leptin receptor (*LEPR*) and attractin (*ATRN*), were consistent with earlier reports by Liu et al.[21] and Wang et al.[28]. The results imply that artificial selection may have driven the gain or loss of copy numbers and thus, specific gene dosages are necessary to form several breed-specific phenotypic characteristics.

**Table 4:** Enriched gene ontology (GO) terms of genes in identified CNV regions (kolmogorov-smirnov *P*-value ≤ 0.05).

Ontology^a^	GO.ID	Term	KS *P*-value	Validated in previous study
CNV.GO_BP	GO:0050911	Detection of chemical stimulus involved in sensory perception of smell	< 1e-30	-
CNV. GO_BP	GO:0007186	G-protein coupled receptor signaling pathway	< 1e-30	Cattle (Liu. G. E. etal. 2010) Pig (Wang. J. etal 2012)
CNV.GO_BP	GO:0006334	Nucleosome assembly	8.50E-08	Pig (Wang. J. etal 2012)
CNV.GO_BP	GO:0050877	Neurological system process	0.00066	Cattle (Liu. G. E. etal. 2010) Pig (Wang. J. etal 2012)
CNV.GO_BP	GO:0002376	Immune system process	0.00081	Cattle (Bickhart. D. M. etal 2012)
CNV.GO_BP	GO:0007165	Signal transduction	0.0054	Cattle (Liu. G. E. etal. 2010)
CNV.GO_BP	GO:0019882	Antigen processing and presentation	0.01789	Pig (Wang. J. etal 2012)
CNV.GO_BP	GO:0007600	Sensory perception	0.03015	Cattle (Liu. G. E. etal. 2010)
CNV.GO_BP	GO:0051056	Regulation of small GTPase mediated signal transduction	0.03445	Pig (Wang. J. etal 2012)
CNV.GO_BP	GO:0007166	Cell surface receptor signaling pathway	0.03971	Cattle (Liu. G. E. etal. 2010) Pig (Wang. J. etal 2012)
CNV.GO_MF	GO:0004984	Olfactory receptor activity	< 1e-30	Cattle (Liu. G. E. etal. 2010) Pig (Wang. J. etal 2012)
CNV.GO_MF	GO:0004930	G-protein coupled receptor activity	< 1e-30	Cattle (Liu. G. E. etal. 2010)
CNV.GO_MF	GO:0046982	Protein heterodimerization activity	0.00736	Pig (Wang. J. etal 2012)
CNV.GO_MF	GO:0070330	Aromatase activity	0.00905	Pig (Wang. J. etal 2012)
CNV.GO_MF	GO:0003824	Catalytic activity	0.04975	Cattle (Bickhart. D. M. etal 2012)
CNV.GO_CC	GO:0016021	Integral to membrane	< 1e-30	Cattle (Liu. G. E. etal. 2010) Pig (Wang. J. etal 2012)
CNV.GO_CC	GO:0005886	Plasma membrane	< 1e-30	Cattle (Liu. G. E. etal. 2010)
CNV.GO_CC	GO:0000786	Nucleosome	5.70E-07	Pig (Wang. J. etal 2012)
CNV.GO_CC	GO:0044464	Cell part	0.03365	Cattle (Bickhart. D. M. etal 2012)
CNV.GO_CC	GO:0005623	Cell	0.03365	Cattle (Bickhart. D. M. etal 2012)

^a^The three Ontologies of GO enrichments BP, MF, and CC represented Biological Process, Molecular Function, and Cellular Component, respectively.

### Functional validation of copy number variation of *LEPR* gene

**Fig 4:**
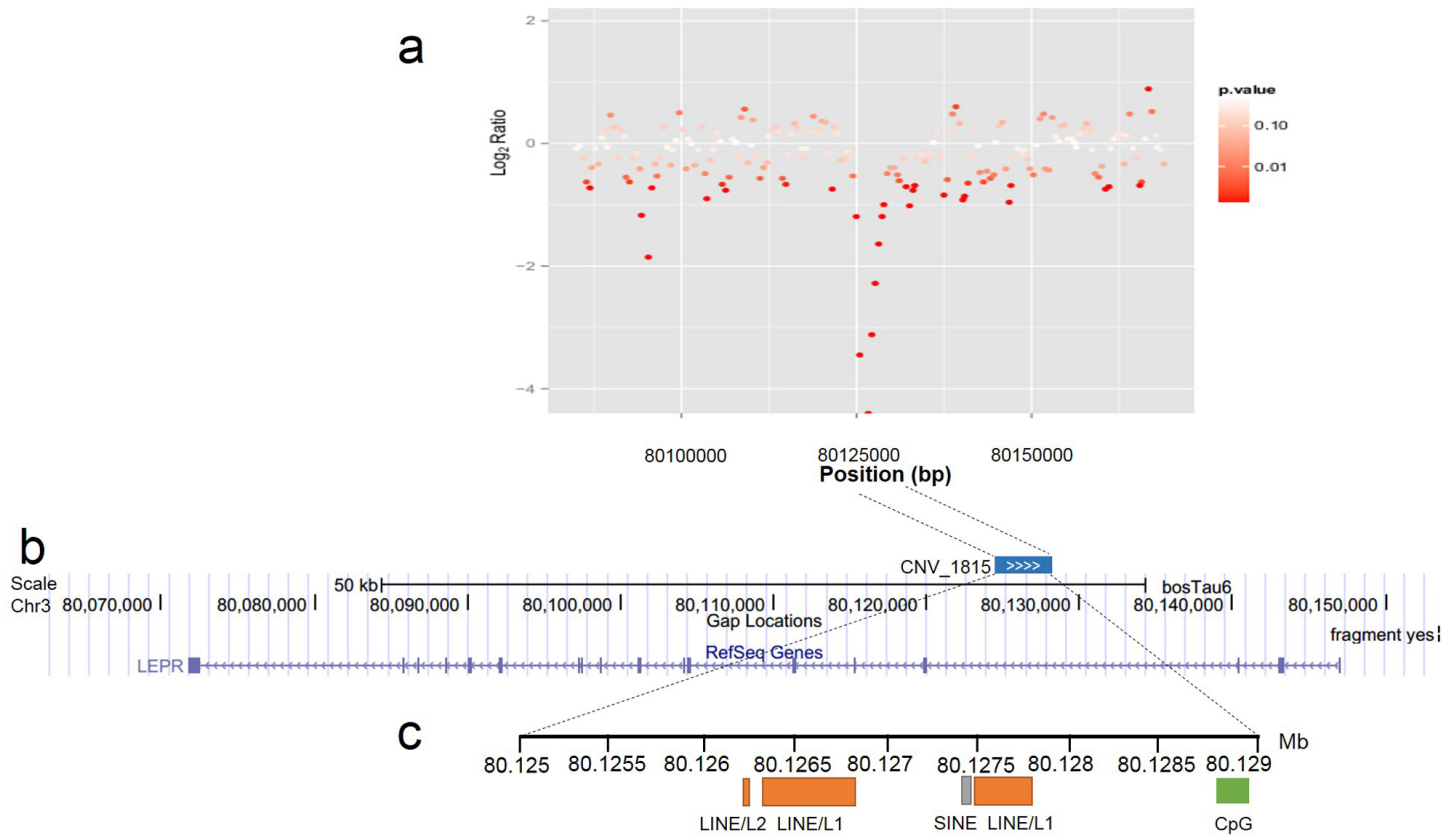
The bovine Lepin receptor (*LEPR*) gene overlapping with copy number variation (CNV) region. a represented log_2_ ratio plot of the *LEPR* gene region. Each point shows the log_2_ ratio of Nanyang reads mapped to Qinchuan reads. Points are coloured based on the log_10_ p-value calculated by the CNV-seq software. b represented the *LEPR* gene region as visualized using the UCSC Genome Browser. The precise boundary of the CNV_1815 that resides in this region is shown and labelled. c represented the localization and composition of important elements in the CNV_1815. The small bars in orange are partial LINEs, and the gray shows SINEs. A small CpG island is marked with a green bar.

According to the above GO enrichment analysis, many CNV genes were over-represented in the terms related to environmental response, which is consistent with other studies of mammalian genomes [29,30]. However, in our study, it was intriguing to note that the leptin receptor (*LEPR*) gene was overlapped with a CNV region (CNV_1815) in chromosome 3 (Fig 4a and 4b). Sequence analysis revealed that this region (*LEPR* CNV) contained partial repetitive elements (LINEs and SINEs) and a small CpG island (Fig 4c), which play important roles in the RNA-mediated gene duplication and methylated regulation [31,32]. In addition, the CNV region showed significantly higher copy numbers in Qinchuan cattle compared with Nanyang, thus we hypothesized that the *LEPR* gene may contribute to higher meat quality of Qinchuan than Nanyang cattle due to its role in lipid metabolism[33].

**Fig 5:**
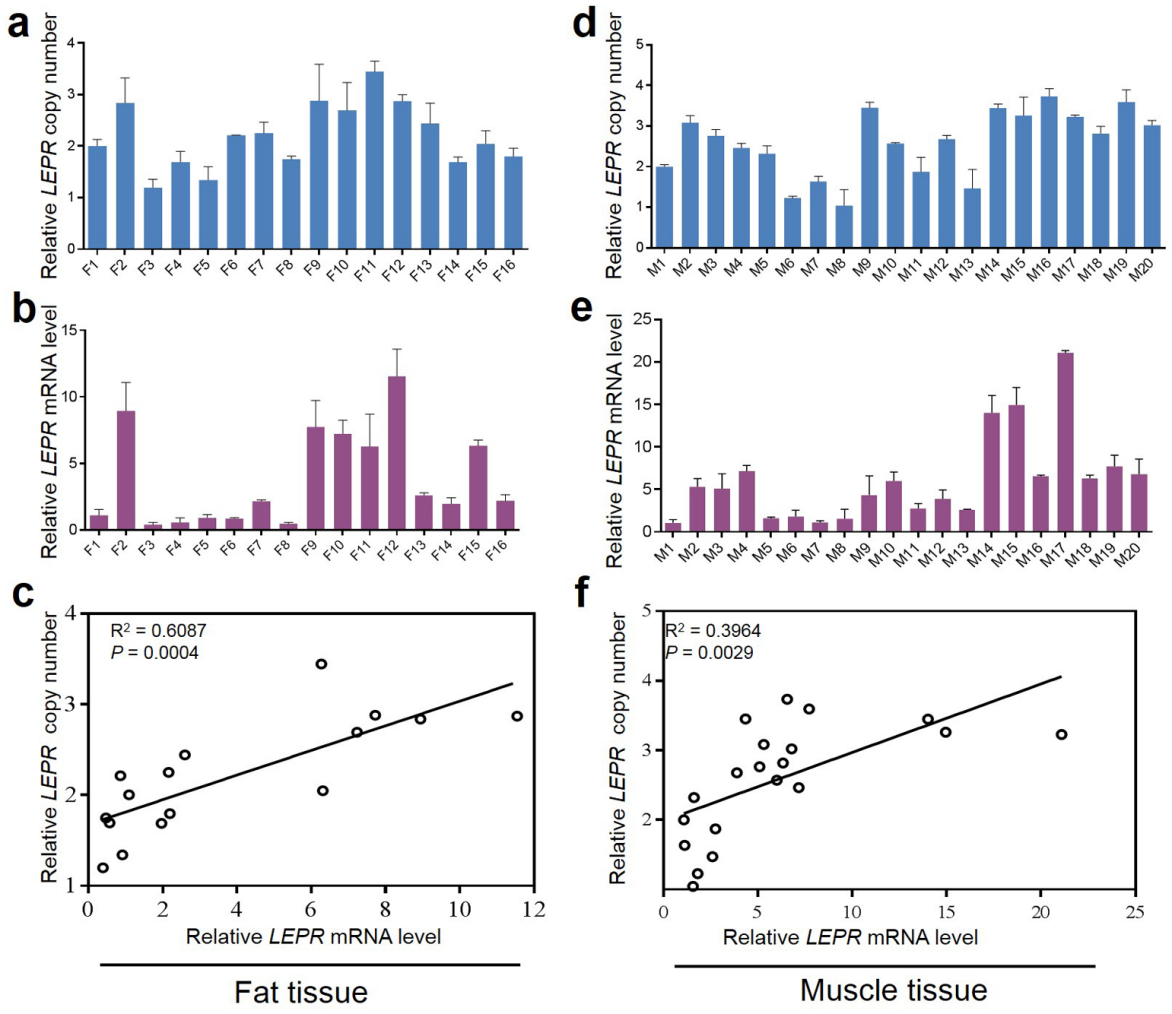
Relationship between the copy number variation (CNV) region of the Lepin receptor (*LEPR*) gene and transcript levels in fat and muscle tissue. a~c represented the correlation result in fat tissue (n = 16, F1~F16). The relative copy numbers and expression of *LEPR* CNV region were normalized as compared to that from the F1 individual. d~f represented the correlation result in muscle tissue (n = 18, M1~M20). The relative copy numbers and expression of *LEPR* CNV region were normalized as compared to that from the M1 individual.

To further test the hypothesis, we sought to determine whether copy number variation affects the expression level of *LEPR* gene in Qinchuan cattle. Firstly, expression profiling of the *LEPR* gene in different tissues revealed that *LEPR* was expressed at a high level in fat and at a moderate level in skeletal muscle tissue (S6 Fig). Thus, correlations between copy number and mRNA levels of the *LEPR* gene were examined in fat (*n* = 16) and skeletal muscle (*n* = 20) separately; results are shown in Fig 5. In both data sets, positive correlations were uncovered (linear regressions: *R*^2^ = 0.61, *P* = 0.0004 for fat; *R*^2^ = 0.40, *P* = 0.0029 for skeletal muscle). In addition, to evaluate whether the potential effects of CNV locus is causative for the different phenotypes between Nanyang and Qinchuan cattle, the association analysis of *LEPR* CNV with growth traits were conducted in Qinchuan population. In the CNV testing analysis, aberrant segments were identified as a CNV locus according to the log_2_ (ratio of test/control). Similarly, the copy number types of the *LEPR* were classified as gain (>0.5), loss (<-0.5) and median (<|±0.5|) based on the log_2_ 2^-ΔΔCt^ relative to the control sample by qPCR analysis. In the statistical model, the *LEPR* copy numbers were normalized to the sequenced Qinchuan cattle (control sample). Correspondingly, the copy number of the gain, loss and median was designated as ≥3, <2 and 2 copies, respectively. As shown in Table 5, the *LEPR* CNV was significantly associated with body weight, hucklebone width and rump length, the individuals with copy number gain had higher values than those with loss or median (*P* < 0.05), which was consistent with the correlation analysis of *LEPR* CNV and transcriptional level.

**Table 5:** Statistical association analysis of bovine *LEPR* gene copy number variations with phenotypic traits in Qinchuan cattle.

Growth traits	CNVs types^a^ (Mean ± SE)			*P*-value
	Gain (n = 32)	Loss (n = 36)	Median (n = 123)	
Body weight, kg	408.06±18.93^a^	381.20±17.84^a^^b^	366.37±9.77^b^	0.043
Body height, cm	127.38±1.45	127.64±1.37	127.27±0.75	0.972
Body length, cm	138.19±2.98	133.89±2.81	131.58±1.54	0.144
Heart girth, cm	178.16±2.99	174.17±2.82	172.30±1.55	0.222
Chest width, cm	38.06±1.12	37.47±1.06	37.05±0.58	0.714
Chest depth, cm	62.50±1.12	61.33±1.05	61.73±0.58	0.740
Height at hip cross, cm	124.56±1.55	125.03±1.46	124.50±0.80	0.950
Hucklebone width, cm	24.41±1.04^a^	22.72±0.98^a^^b^	21.93±0.54^b^	0.037
Hip width, cm	42.56±0.87	40.72±0.82	41.57±0.45	0.311
Rump length, cm	44.75±0.73^a^	42.72±0.69^b^	42.98±0.38^b^	0.035

^a^ The copy number types of the gain, loss and median was designated as ≥3, <2 and 2 copies, respectively. Values with different superscripts (a, b) within the same row differ significantly at *P* <0.05.

## Discussion

The primary goal of our present study was to provide a comprehensive list of sequence variation at the whole genome scale for two widespread Chinese cattle breeds (Nanyang and Qinchuan) and to identify potential loci that might be related to phenotypic differences between the two breeds. In previous studies, examining whole sequence variation is often relied on SNP array[34], array comparative genomic hybridization (aCGH) [21,35] or exome sequencing[36]. However, the SNP array and aCGH approaches obviously have a limited sensitivity and are predisposed to high false positive/negative calling [37]. Exome sequencing provides limited information as the sequence information of regulatory and intergenic regions is unavailable [38]. Thus, in this study, next-generation sequencing, allowing sequence construction at a higher effective resolution and sensitivity[39], was used to identify polymorphisms from genomes of the investigated breeds.

Phenotypic diversity of domesticated animals has been shaped by man-made selection, including selective sweeps, which ought to leave a signature in the genome of domesticated strains or breeds [40]. Our study was motivated by the idea that comparing breeds with contrasting phenotypes may provide molecular basis underlying phenotypic differences, and thus provide novel insights into the effects of artificial selection on the genomes of domesticated animals. The Nanyang and Qinchuan breeds presented marked differences in several phenotypic traits of agro-economic interest, for example, Qinchuan cattle have a higher meat quality and especially higher marbling grades than Nanyang cattle. In fact, our genomic sequencing approach identified a number of loci that could be related to these phenotypic differences. The sequencing results indicated that Nanyang showed more genetic variation than Qinchuan, including SNPs, small indels and CNVs, suggesting that Nanyang cattle could have a larger effective population size (*Nm*), while in case of Qinchuan, they may own fewer breeding bulls, leading to the loss of genetic variability due to drift and bottlenecks. In addition, Nanyang may possess higher divergence comparing to the *Bos taurus* reference sequence, which indicate that Nanyang has inherited the genetic characteristics from *Bos indicus*, and it may be phylogenetically more distinct from the reference cattle genome.

We sequenced the Nanyang and Qinchuan genomes at 10–12 fold depth, and the averages of 97.9% and 98.9% of the whole genome sequences were covered, respectively. We used a slightly higher read depth than a previous study on the Fleckvieh breed (7.4-fold), which uncovered 2.44 million SNPs [11]. Lee et al. [13] sequenced the genome with 45.6-fold depth in the case of the Korean Hanwoo cattle; however only 4,781,758 SNPs were identified—by far less than in our present study (9,008,518 for Nanyang; 6,963,517 for Qinchuan). Sequencing Japanese Kuchinoshima-Ushi cattle uncovered 6,303,790 SNPs [12]. while 3,755,663 (19-fold) and 3,246,211 SNPs (22-fold) were found in Holstein and Black Angus (North American cattle), respectively [15]. These investigations suggested that the number of genomic SNPs showed remarkable differences among different cattle breeds, and the genomic variabilities may become valuable resources for future breeding campaigns—e.g, the international cattle industry my focus on desirable phenotypic traits as seen in Chinese breeds, like the famous meat structure and quality of Qinchuan cattle.

It is straight forward to predict that non-synonymous SNPs have a high predictive power to explain phenotypic differences[41], because non-synonymous substitutions lead to an altered protein structure and/or spatiotemporal patterns of gene expression [42]. In our study, the percentage of non-synonymous SNPs in coding regions was 41.14% and 40.13% for Nanyang and Qinchuan, respectively, which was higher than any previous report from whole-genome resequencing approaches in cattle [12,15]. Several genes with non-synonymous SNPs (equal or more than 10 non-synonymous SNPs per gene) have been reported to be associated with agro-economically important traits, such as growth rate and meat production [43]. Growth-related genes like growth hormone (*GH*) [44], growth hormone receptor (*GHR*) [43] and insulin-like growth factor 1 (*IGF1*) [45], comprised more non-synonymous SNPs in Nanyang than that in Qinchuan. Contrarily, Qinchuan genome showed a larger number of non-synonymous SNPs in genes associated with meat (i.e., muscle tissue) development, such as diacylglycerol O-acyltransferase 1(*DGAT1*) [46] and leptin (*LEP*) [47]. Future studies will need to be conducted to understand the functional roles of these non-synonymous substitutions during muscle formation and development in Qinchuan cattle—research that will be of interest for the international cattle industry seeking to improve meat quality of different breeds worldwide.

Moreover, a list of genes with > = 50 NS/FS/Indel were identified in this study, and most of the genes were important in cellular defense, adaptive immunity, and environmental response, which corresponded well with the previous studies in human and other animals [29,30,48,49]. For example, sequence variations of the major histocompatibility complex (MHC), a master coordinator of specificity in both adaptive and innate immune systems, are related to a large number of infectious, autoimmune and other diseases [50]. In addition, Bergen et al. [51] established the genome-wide association study and showed that SNPs of MHC were significantly associated with schizophrenia in a Swedish population.

A substantial number of CNVs were detected in the Nanyang when aligning it to the Qinchuan. Statistics analysis showed that the minimal and mean length of CNVs were 1,776 bp and 3,183 bp, respectively, which is in accordance with other sequencing-based studies on cattle [52], but considerably shorter than what has been reported based on SNP/CGH array methodology (several mega-base pairs) [21,53]. This discrepancy can be attributed, in part, to different criteria for reporting CNVs. The approach we used here can artificially break a single CNV into multiple CNVs [54]. We also compared our results to previous reports on CNVs in cattle, which were identified using two different technologies (S6 Table). Stothard et al. [15] applied whole-genome resequencing to detect CNVs in Holstein and found 790 cases of presumed CNV by mapping the Holstein genome to the Btau4.0 reference sequence. Only 11 out of those 790 CNVs (1.4%) were identical or overlapping with CNVs detected in our present study. Using next-generation sequencing approach, Bickhart et al. [14] reported 1,265 CNVs across all chromosomes in five cattle, and 5.1% (65/1265) overlapped with our results. This comparison suggests that we detected a great number of previously unknown cases of CNV in the cattle genome. Moreover, in order to validate the sequencing-based CNV call set, we selected 20 CNVs for qPCR and achieved a confirmed rate of 90%, which was higher than most confirmation rates in previous studies, such as the study by Hou et al. [53] in 15 cattle (60.00%), Bickhart et al. [14] detected 12 CNV loci in BINE, BTAN1, BTAN2, BTAN3 and BTHO cattle (82.14%) but a little lower than that in modern domesticated cattle (91.67%) reported by Liu et al. [21]. Notably, the previous study has not detect breed-specific CNVs, and we for the first time demonstrated major differences in copy numbers of certain loci in randomly selected individuals from Nanyang and Qinchuan (*n* = 10 individuals each). The results were completely concordant with our sequencing data.

We detected a considerable number of annotated genes (495 Ensembl genes) in CNVs regions. As previously shown, genes in a CNV region can contribute to phenotypic variation by changing gene structure, alternating gene regulation, exposing recessive alleles, and other mechanisms [55]. In recent years, many functional CNV genes have been investigated in cattle, for instance, the *PLA2G2D* gene, related to milk production and meat quality, was highly duplicated in beef cattle breeds [15]. Xu et al. [56,57] reported that the duplicated bovine *MYH3* and *MICAL-L1* genes, located on the quantitative trait loci (QTLs) for body weight, were associated with growth traits in Chinese cattle. In our study, GO analysis of CNV genes revealed that terms such as “innate and adaptive immunity”, and “receptor recognition” were enriched, which is consistent with investigations on CNVs in human, mouse, dog, and cattle [29,30,48,58]. Among several genes related to lipid transport and fat metabolism, the *LEPR* gene was explored in more detail as it showed a great deal of copy number differences between the Nanyang and Qinchuan breeds. The CNV region contains variable copies of the LINEs, SINEs and CpG elements, which could be relevant for the mechanism of action of this noncoding variation. Wright et al. [59] has reported that in chicken the copy number variations in intron 1 of *SOX5* were associated with the pea-comb phenotypes. The LEPR protein, a receptor of leptin, is produced by fat cells, and influences food intake, fat metabolism, and reproductive functions [60]. A previous study reported that an increased copy number in the "E2 DNA" region (exon-intron junction) of the *LEPR* gene lead to an increased fat deposition in humans, and *LEPR* CNV is thought to be involved in obesity and type 2 diabetes mellitus [61]; this suggests that artificial selection in cattle seems to have selected for a trait that in other biological systems occurs as a rare disease. By referring to the cattle QTLdb (quantitative trait loci database) [62], the bovine *LEPR* gene was mapped at an 84 cM interval on chromosome 3, in which the QTL region (no. 13,158) for the fat thickness at the 12^th^ rib trait was located [43]. Additionally, significantly positive correlations were observed between the copy number of the *LEPR* CNV and its transcription levels in skeletal muscle and fat tissues, suggesting that the CNV region of the *LEPR* gene may indeed affect phenotypic traits of cattle by affecting the copy number of transcripts and ultimately, the LEPR protein. A straight forward working hypothesis arising from our study is that the higher copy number of the *LEPR* CNV in Qinchuan cattle is one of the main factors responsible for increased fat deposition in muscle tissue—a desirable trait for meat production.

Whole genome sequencing presented the first description of genomic variations in the Chinese Nanyang and Qinchuan cattle. In comparison with the studies previously reported, the two Chinese cattle genomes showed a higher degree of genetic diversity than those of other cattle breeds, and the Nanyang presented more abundant variations than Qinchuan. According to the GO enrichment analysis results, we conclude that the bovine *LEPR* gene may be one of the causative genes contributing to the different phenotypes. Positive correlations have been observed between the intronic CNV region of *LEPR* gene and its mRNA levels. In summary, our findings provide a comprehensive appreciation of the full dimension of bovine genetic variations, which may unravel the genetic basis for the improvement of economic phenotypes in cattle.

## Supporting information

S1 Fig Sequencing coverage of the Nanyang and Qinchuan genomes. The x-axis indicated 30 chromosomes (including autosomes and the X chromosome) of the reference genome. The left y-axis represented the length of chromosome (0~160 Mbp), and the right y-axis represented the percentage scale of sequencing coverage (0%~100%). Bars in blue showed the covered region by the sequenced reads, and bars in red showed the uncovered sequence region. The black lines above indicated the percentage of sequencing coverage in each chromosome.
(PDF)

S2 Fig Novel and common SNPs in each chromosome of Nanyang and Qinchuan cattle genome. A, The number of novel SNPs. B, The number of common SNPs. Blue bars indicated the Nanyang and purple bars showed the Qinchuan genome. Herein, the "novel" means a variant that was not found in dbSNP.
(PDF)

S3 Fig Distribution of different indel size in whole genome and CDS region. The x-axis indicated indel size of 1 bp, 2 bp, and 3 bp. The left y-axis represented the insertion and deletion in whole genome, and the right y-axis represented the distribution in CDS region.
(PDF)

S4 Fig Statistics of SNPs and indels in each chromosome. The left y-axis represented the number of genetic variations (0~600,000 for SNPs; 0~10,000 for indels), and the right y-axis represented the scale of chromosome size (0~200 Mbp). Blue and red bars indicated the statistical results of Nanyang and Qinchuan genome, respectively. The green lines represented the length of chromosome.
(PDF)

S5 Fig Validation of CNVs in individual animals of Nanyang and Qinchuan breeds. The validation results for genic (including gains and losses) and non-genic (including gains and losses) CNV region were provided. The scattergrams of relative copy number were shown for Nanyang (n = 10) and Qinchuan (n = 10). The name of the overlapping genes were given in parentheses for genic CNVs.
(PDF)

S6 Fig Expression profiling of *LEPR* gene in different tissues of adult cattle. The values are the averages of three independent experiments measured by 2^-ΔΔCt^. Error bars represent the standard deviation (SD) (n = 3), and the relative mRNA expression levels of *LEPR* are normalized to *GAPDH*.
(PDF)

S1 Table Evaluation of the sequencing data in Nanyang and Qinchuan genome. (PDF)

S2 Table The percentage of SNPs with transition, transversion, and heterozygosity in each chromosome of Nanyang and Qinchuan genomes. (PDF)

S3 Table The percentage of small indels with insertion, deletion, and both insertion and deletion in each chromosome of Nanyang and Qinchuan genomes. (PDF)

S4 Table Statistical number of the genes harboring different mutations. (PDF)

S5 Table The list of all CNVs detected in this work. The position of start and end, CNV size, log_2_, and p.value were shown for each CNV.
(PDF)

S6 Table The CNVs in our study were identical or overlapped with those reported in previous papers. (PDF)

S7 Table The information of primers used in this study. A total of 20 primer pairs were used for validation in mRNA level, and 2 primer pairs were used for detection in DNA level.
(PDF)
