# Supplementary figures and images for: Whole-genome sequencing reveals mutational landscape underlying phenotypic differences between two widespread Chinese cattle breeds
Source: PLoS One. 2017 Aug 25;12(8):e0183921. doi: 10.1371/journal.pone.0183921 (PMC5571935; doi:10.1371/journal.pone.0183921)

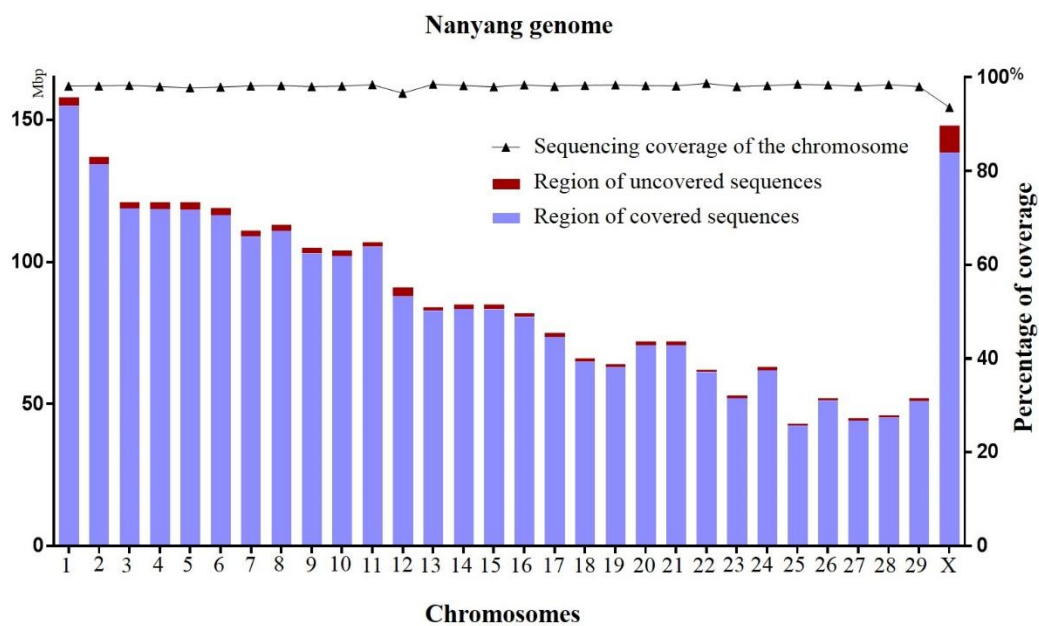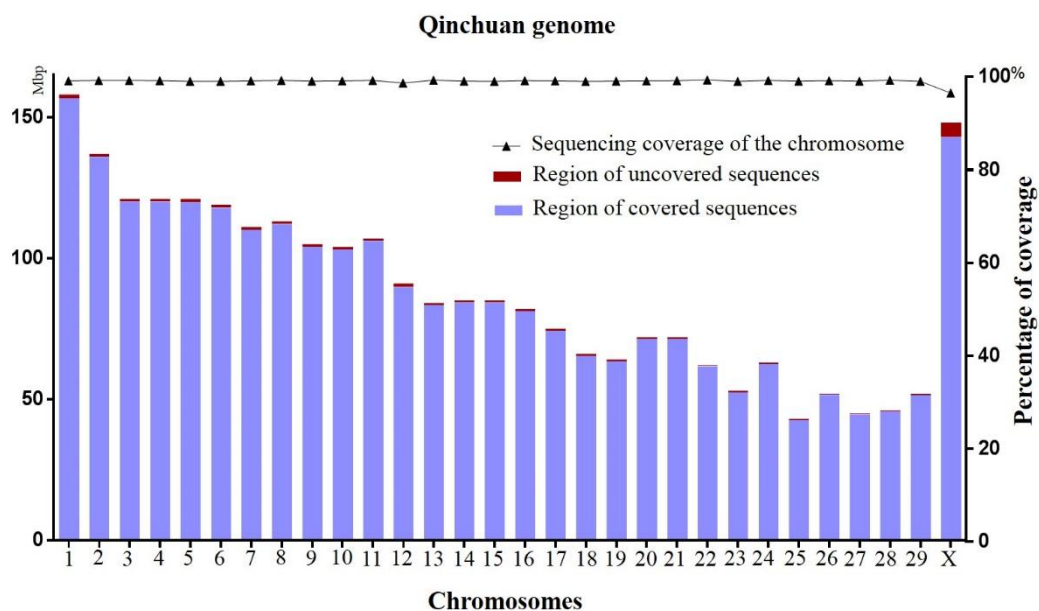

Supplement: S1 Fig — The x-axis indicated 30 chromosomes (including autosomes and the X chromosome) of the reference genome. The left y-axis represented the length of chromosome (0~160 Mbp), and the right y-axis represented the percentage scale of sequencing coverage (0%~100%). Bars in blue showed the covered region by the sequenced reads, and bars in red showed the uncovered sequence region. The black lines above indicated the percentage of sequencing coverage in each chromosome. (PDF) [file pone.0183921.s001.pdf]

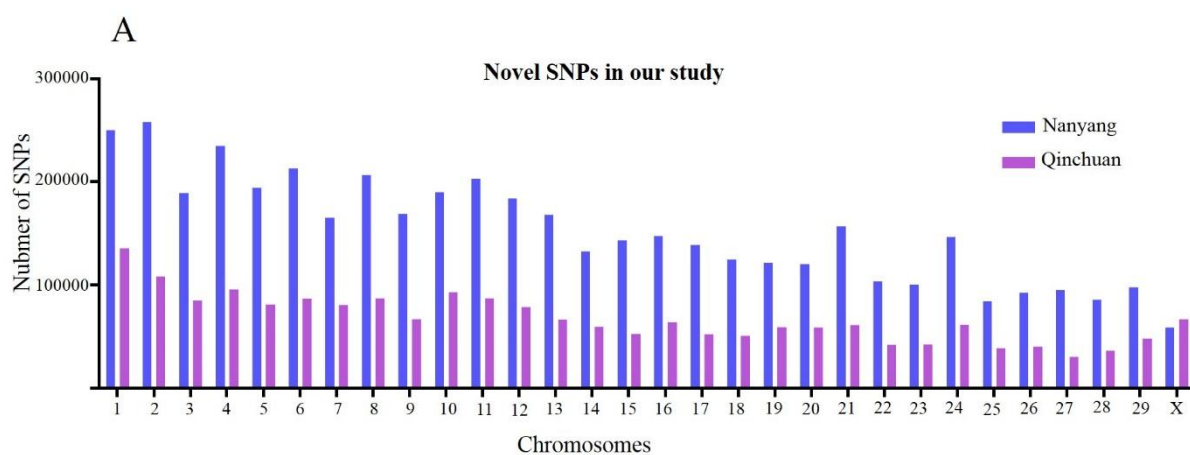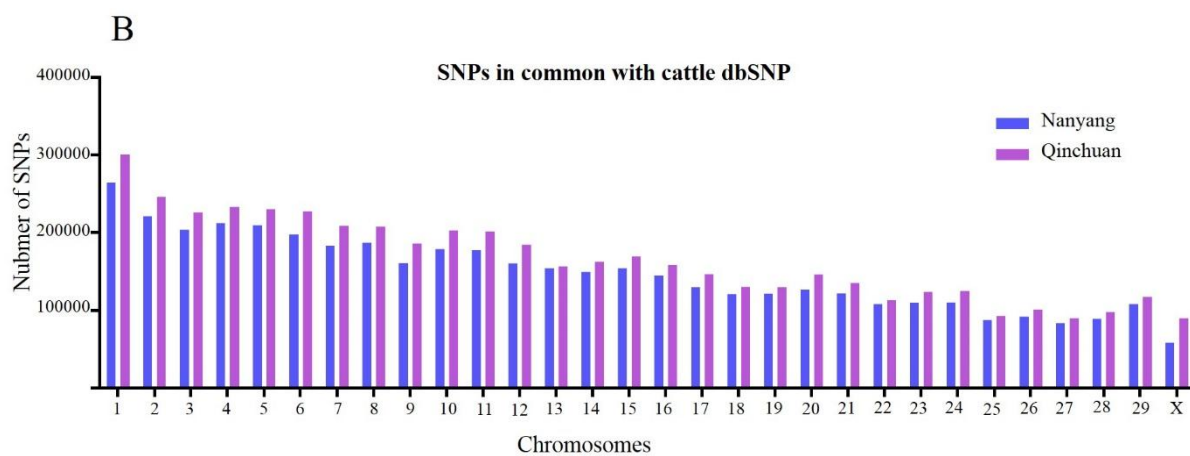

Supplement: S2 Fig — A, The number of novel SNPs. B, The number of common SNPs. Blue bars indicated the Nanyang and purple bars showed the Qinchuan genome. Herein, the "novel" means a variant that was not found in dbSNP. (PDF) [file pone.0183921.s002.pdf]

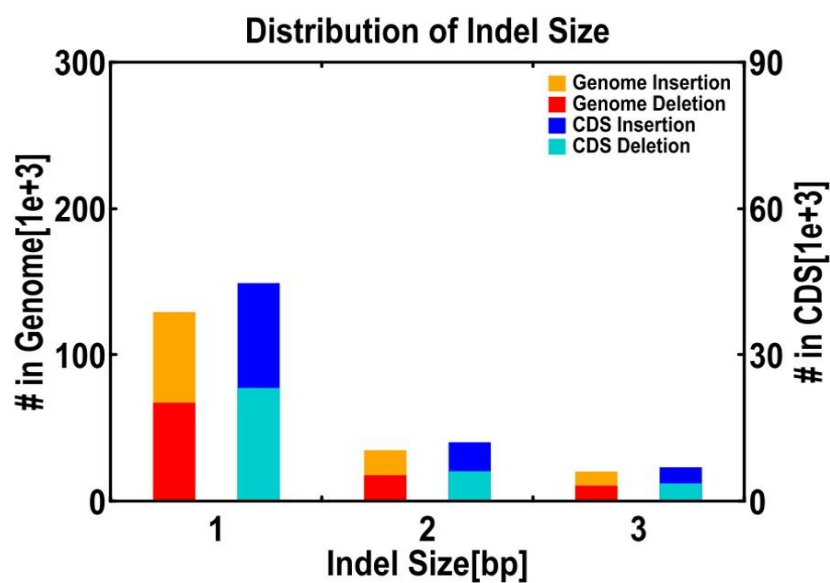

**Indels of Nanyang genome**

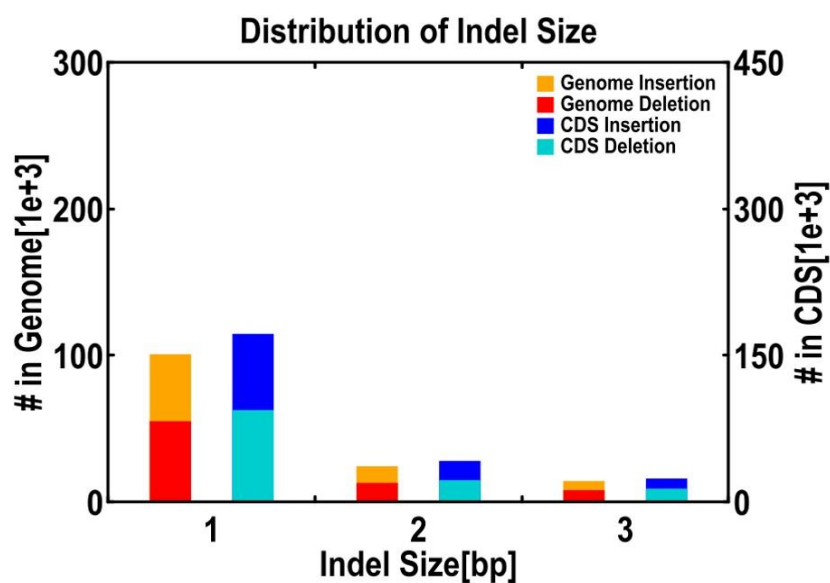

**Indels of Qinchuan genome**

Supplement: S3 Fig — The x-axis indicated indel size of 1 bp, 2 bp, and 3 bp. The left y-axis represented the insertion and deletion in whole genome, and the right y-axis represented the distribution in CDS region. (PDF) [file pone.0183921.s003.pdf]

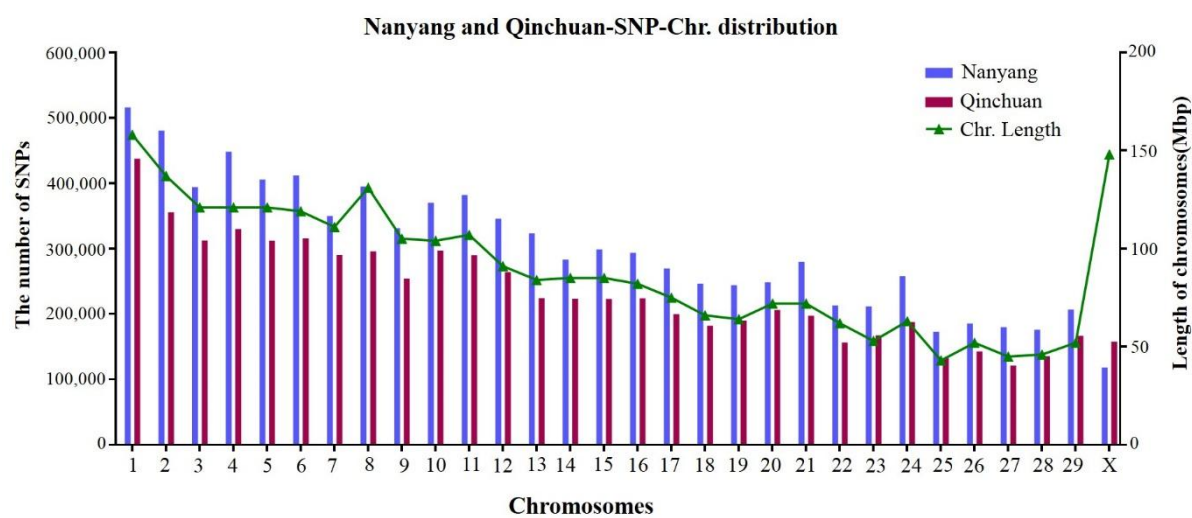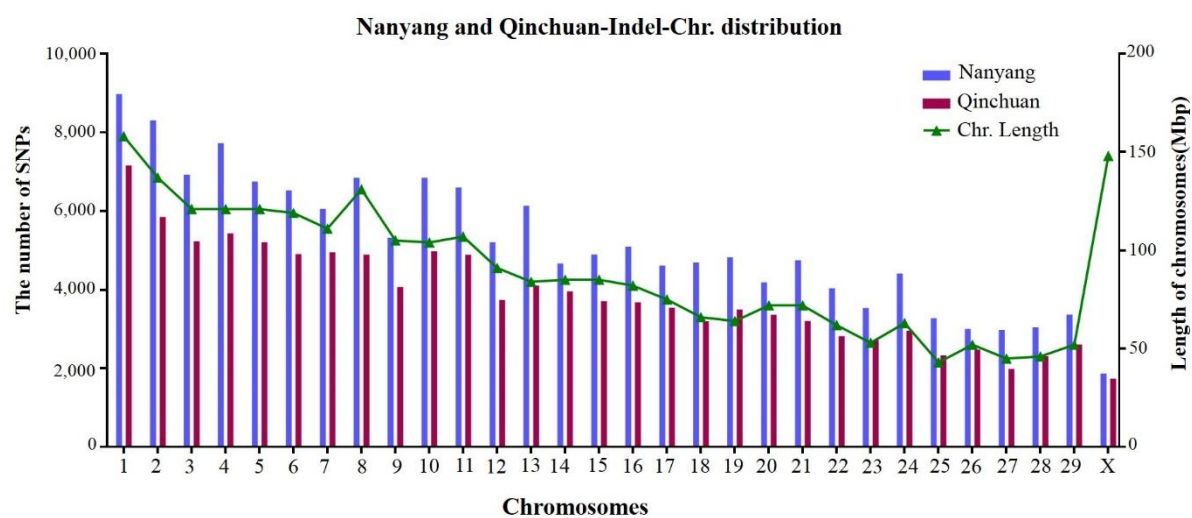

Supplement: S4 Fig — The left y-axis represented the number of genetic variations (0~600,000 for SNPs; 0~10,000 for indels), and the right y-axis represented the scale of chromosome size (0~200 Mbp). Blue and red bars indicated the statistical results of Nanyang and Qinchuan genome, respectively. The green lines represented the length of chromosome. (PDF) [file pone.0183921.s004.pdf]

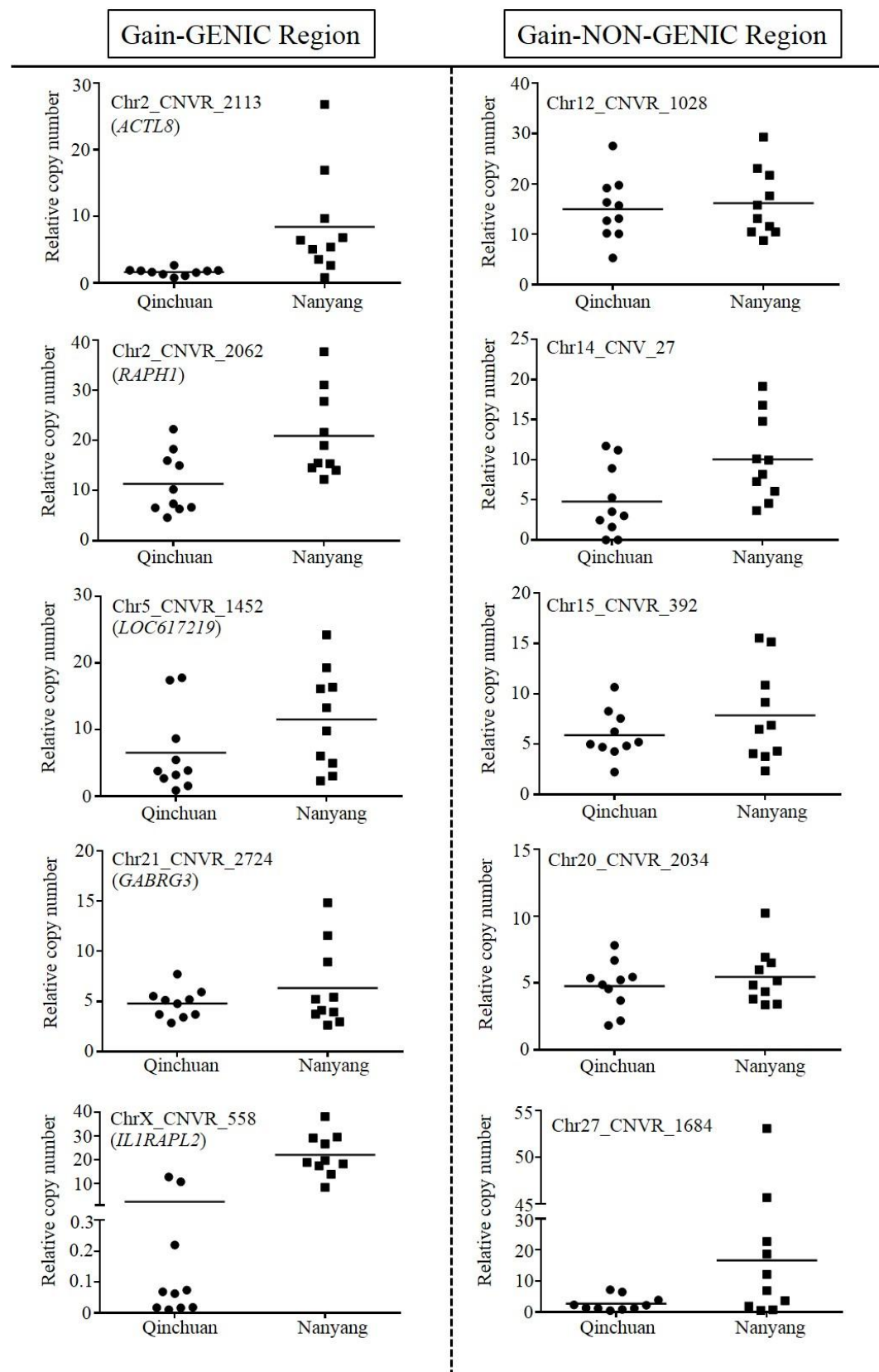

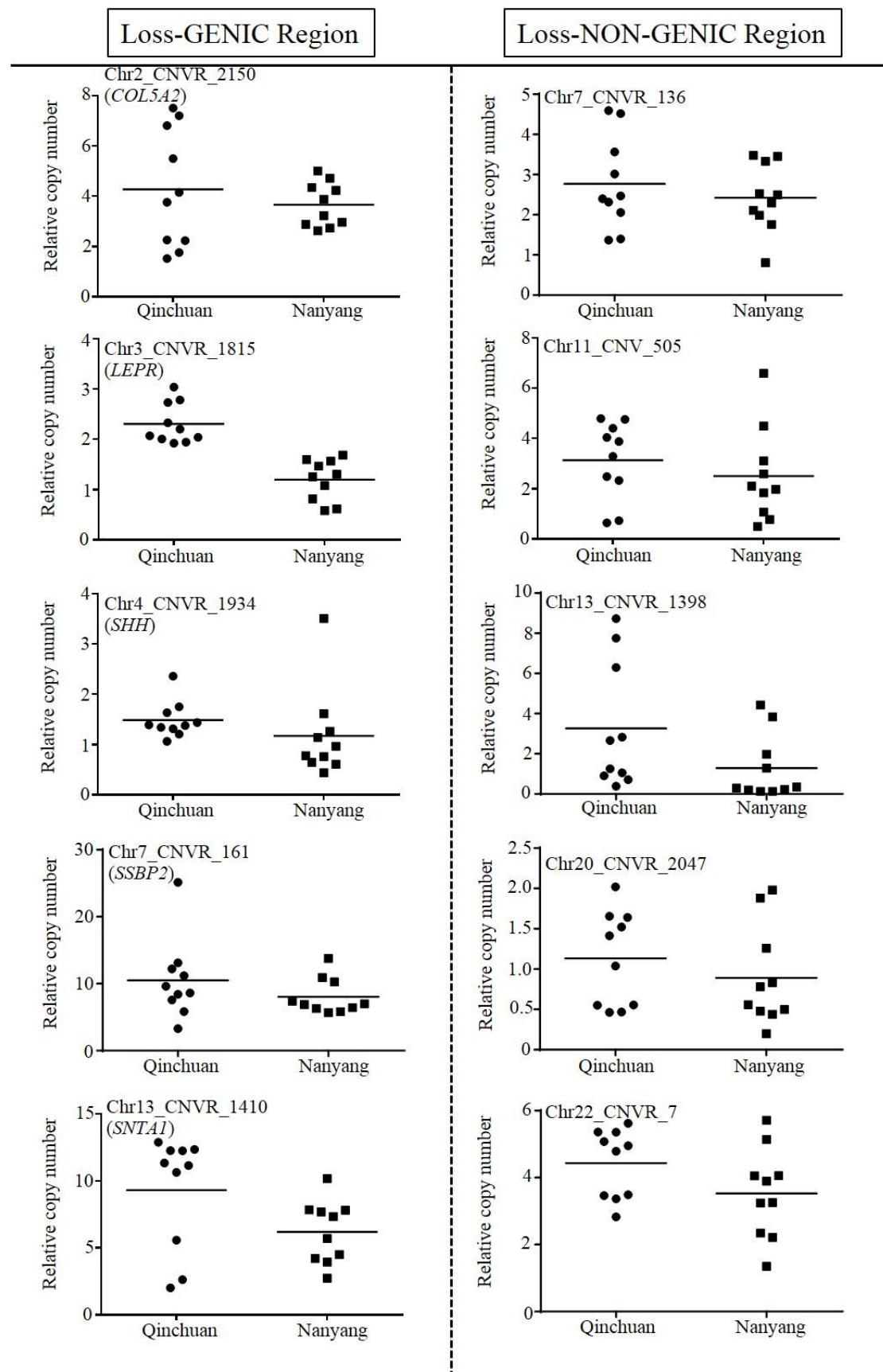

Supplement: S5 Fig — The validation results for genic (including gains and losses) and non-genic (including gains and losses) CNV region were provided. The scattergrams of relative copy number were shown for Nanyang (n = 10) and Qinchuan (n = 10). The name of the overlapping genes were given in parentheses for genic CNVs. (PDF) [file pone.0183921.s005.pdf]

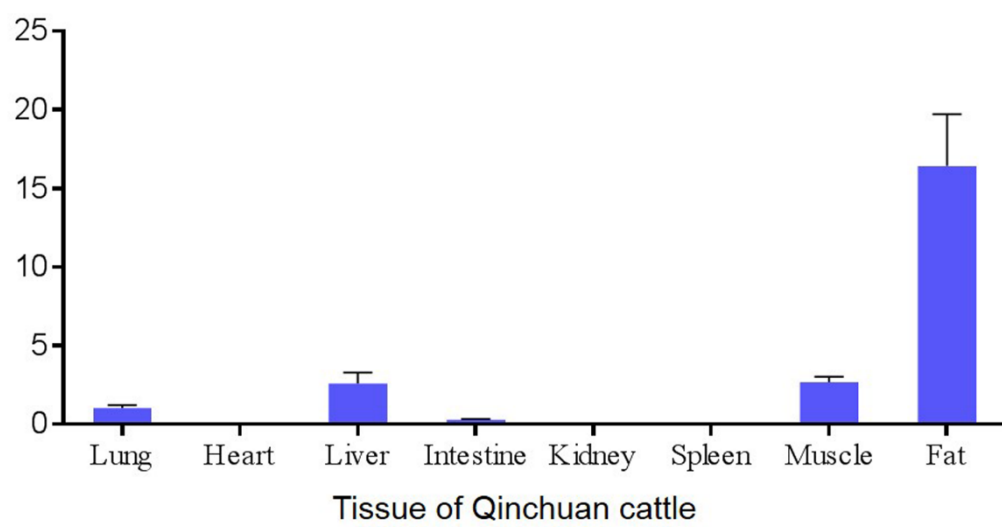

Supplement: S6 Fig — The values are the averages of three independent experiments measured by 2-ΔΔCt. Error bars represent the standard deviation (SD) (n = 3), and the relative mRNA expression levels of LEPR are normalized to GAPDH. (PDF) [file pone.0183921.s006.pdf]
