# Supplementary material for: Whole-genome sequencing reveals mutational landscape underlying phenotypic differences between two widespread Chinese cattle breeds
Source: PLoS One. 2017 Aug 25;12(8):e0183921. doi: 10.1371/journal.pone.0183921 (PMC5571935; doi:10.1371/journal.pone.0183921)
Supplement: S1 Table — (PDF) [file pone.0183921.s007.pdf]

S1 Table. Evaluation of the sequencing data in Nanyang and Qinchuan genome

Genome	Total reads	Total nucleotides (bp)	Q20 percentage	GC percentage
Nanyang	184,341,794	37,237,042,388	100.00	45.64
Qinchuan	149,589,163	30,217,010,926	100.00	45.99

Note: Q20 percentage represented the ratio of the values quality ≥ 20 (Q20).
