# Supplementary material for: Whole-genome sequencing reveals mutational landscape underlying phenotypic differences between two widespread Chinese cattle breeds
Source: PLoS One. 2017 Aug 25;12(8):e0183921. doi: 10.1371/journal.pone.0183921 (PMC5571935; doi:10.1371/journal.pone.0183921)
Supplement: S2 Table — (PDF) [file pone.0183921.s008.pdf]

S2 Table. The percentage of SNPs with transition, transversion, and heterozygosity in each chromosome of Nanyang and Qinchuan genomes.

#Chr	SNP number		Transition (%)		Transversion (%)		Heterozygosity (%)	
	Nanyang	Qinchuan	Nanyang	Qinchuan	Nanyang	Qinchuan	Nanyang	Qinchuan
1	514888	436399	70.90	71.24	29.10	28.76	63.72	91.34
2	479213	354634	71.11	71.43	28.89	28.57	65.35	91.60
3	393146	311372	71.40	71.64	28.60	28.36	64.96	91.50
4	447180	328873	71.14	71.45	28.86	28.55	63.85	92.18
5	404360	311096	71.41	71.83	28.59	28.17	66.96	90.69
6	410832	314488	70.70	70.96	29.30	29.04	67.08	90.53
7	348832	289353	71.22	71.38	28.78	28.62	70.58	91.49
8	394044	294986	71.08	71.46	28.92	28.54	65.68	91.75
9	330236	253240	70.88	71.39	29.12	28.61	66.24	90.43
10	369099	296203	71.27	71.64	28.73	28.36	65.21	91.43
11	381005	288947	71.94	72.23	28.06	27.77	67.57	90.67
12	344854	263108	70.79	71.17	29.21	28.83	69.58	90.88
13	322341	223070	72.25	72.59	27.75	27.41	61.76	91.57
14	282053	222107	71.80	72.10	28.20	27.90	75.05	90.57
15	297524	221955	70.97	71.18	29.03	28.82	66.35	90.76
16	292731	222714	71.64	71.90	28.36	28.10	63.58	91.54
17	268604	198984	71.35	71.67	28.65	28.33	68.76	91.09
18	245642	181168	71.82	72.30	28.18	27.70	63.38	92.14
19	243081	189259	72.30	72.65	27.70	27.35	62.49	91.72
20	247371	205164	71.33	71.85	28.67	28.15	64.82	90.41
21	278594	196533	71.81	72.21	28.19	27.79	66.61	92.05
22	211818	155471	71.95	72.40	28.05	27.60	71.65	92.14
23	210580	166438	72.05	72.15	27.95	27.85	67.45	91.11
24	256846	186896	71.91	72.35	28.09	27.65	63.85	92.16
25	172017	131887	73.44	74.19	26.56	25.81	63.50	92.35
26	184567	141623	71.79	72.24	28.21	27.76	68.88	91.52
27	178978	120316	72.17	72.21	27.83	27.79	68.71	91.74
28	174907	134508	71.12	71.53	28.88	28.47	66.05	92.20
29	205967	165622	71.91	72.15	28.09	27.85	68.18	91.72
X	117208	157103	67.67	68.42	32.33	31.58	40.98	89.15
Total	9008518	6963517	71.41	71.71	28.59	28.29	65.98	91.31
