# Supplementary material for: Whole-genome sequencing reveals mutational landscape underlying phenotypic differences between two widespread Chinese cattle breeds
Source: PLoS One. 2017 Aug 25;12(8):e0183921. doi: 10.1371/journal.pone.0183921 (PMC5571935; doi:10.1371/journal.pone.0183921)
Supplement: S3 Table — (PDF) [file pone.0183921.s009.pdf]

S3 Table. The percentage of small indels with insertion, deletion, and both insertion and deletion in each chromosome of Nanyang and Qinchuan genomes.

#Chr	Indel number		INS (%)		DEL (%)		IDEL (%)	
	Nanyang	Qinchuan	Nanyang	Qinchuan	Nanyang	Qinchuan	Nanyang	Qinchuan
1	8957	7147	51.90	54.78	47.67	44.66	0.42	0.56
2	8287	5830	50.22	55.28	49.67	44.43	0.11	0.29
3	6905	5213	51.89	53.00	47.91	46.84	0.20	0.15
4	7706	5415	51.10	55.94	48.72	43.92	0.18	0.15
5	6737	5196	51.43	55.04	48.32	44.80	0.25	0.15
6	6507	4887	51.76	53.39	48.01	46.53	0.23	0.08
7	6037	4935	51.75	54.41	48.05	45.49	0.20	0.10
8	6831	4874	52.10	53.82	47.61	45.98	0.29	0.21
9	5310	4051	52.18	54.83	47.68	45.03	0.13	0.15
10	6832	4961	51.70	53.84	48.20	45.96	0.10	0.20
11	6581	4868	51.21	53.64	48.66	46.18	0.14	0.18
12	5196	3723	52.14	52.03	47.71	47.76	0.15	0.21
13	6114	4088	50.49	52.15	49.41	47.68	0.10	0.17
14	4650	3939	52.41	53.47	47.20	45.93	0.39	0.61
15	4876	3694	51.21	52.87	48.65	46.89	0.14	0.24
16	5078	3664	51.54	53.19	48.31	46.45	0.16	0.35
17	4597	3526	50.45	54.57	49.23	45.21	0.33	0.23
18	4677	3187	50.65	52.59	49.24	47.32	0.11	0.09
19	4804	3481	50.12	52.31	49.71	47.57	0.17	0.11
20	4168	3347	51.73	54.23	48.13	45.59	0.14	0.18
21	4735	3193	50.79	54.68	49.06	45.19	0.15	0.13
22	4019	2805	51.28	54.08	48.59	45.92	0.12	0.00
23	3519	2697	50.78	53.84	49.10	46.09	0.11	0.07
24	4391	2940	50.49	52.96	49.35	46.97	0.16	0.07
25	3256	2313	50.58	52.49	49.23	47.25	0.18	0.26
26	2989	2460	51.69	55.89	48.14	44.07	0.17	0.04
27	2956	1967	51.25	51.65	48.51	48.14	0.24	0.20
28	3023	2297	50.18	50.20	49.62	49.63	0.20	0.17
29	3353	2588	50.01	53.44	49.78	46.41	0.21	0.15
X	1853	1719	49.33	52.07	50.62	47.76	0.05	0.17
Total	154944	115005	51.25	53.76	48.56	46.03	0.19	0.21
