# Supplementary material for: Whole-genome sequencing reveals mutational landscape underlying phenotypic differences between two widespread Chinese cattle breeds
Source: PLoS One. 2017 Aug 25;12(8):e0183921. doi: 10.1371/journal.pone.0183921 (PMC5571935; doi:10.1371/journal.pone.0183921)
Supplement: S4 Table — (PDF) [file pone.0183921.s010.pdf]

S4 Table. Statistical number of the genes harboring different mutations.

	Nanyang	Qinchuan
Genes with.Non-synonymous SNPs	11712	10991
Non-synonymous SNPs	37309	30389
Genes with (>=10) Non-synonymous SNPs	547	330
(>=10) Non-synonymous SNPs	8280	4969
Genes with Frame-shift InDels	226	215
Frame-shift Indels	239	231
Genes with Indels	340	300
Indels in Genes	355	323
