# Supplementary material for: Whole-genome sequencing reveals mutational landscape underlying phenotypic differences between two widespread Chinese cattle breeds
Source: PLoS One. 2017 Aug 25;12(8):e0183921. doi: 10.1371/journal.pone.0183921 (PMC5571935; doi:10.1371/journal.pone.0183921)
Supplement: S5 Table — The position of start and end, CNV size, log2, and p.value were shown for each CNV. (PDF) [file pone.0183921.s011.pdf]

S5 Table. The list of all CNVs detected in Nanyang and Qinchuan genomes. The position of start and end, CNV size, log2, and p.value were shown for each CNV.

CNV	Chromosome	Start	End	Size	Log2	P.value
CNVR_1	chrAC_000179.1	675991	677766	1776	1.1377	4.66E-23
CNVR_2	chrAC_000179.1	1695859	1698522	2664	1.6102	1.01E-55
CNVR_3	chrAC_000179.1	2602951	2607390	4440	1.8560	1.62E-109
CNVR_4	chrAC_000179.1	2599843	2602506	2664	-1.4603	2.05E-41
CNVR_5	chrAC_000179.1	6575419	6577638	2220	-2.1649	4.15E-54
CNVR_6	chrAC_000179.1	15406579	15409242	2664	-1.7958	3.41E-53
CNVR_7	chrAC_000179.1	20663095	20665314	2220	-3.0145	4.01E-70
CNVR_8	chrAC_000179.1	22886203	22887978	1776	-1.1257	7.98E-20
CNVR_9	chrAC_000179.1	22892419	22894638	2220	-1.2278	1.41E-27
CNVR_10	chrAC_000179.1	42736555	42738774	2220	-1.3755	3.44E-32
CNVR_11	chrAC_000179.1	42798715	42800490	1776	-1.0258	2.44E-17
CNVR_12	chrAC_000179.1	45528427	45530202	1776	-1.4136	4.99E-27
CNVR_13	chrAC_000179.1	47474035	47477142	3108	-1.7965	8.81E-62
CNVR_14	chrAC_000179.1	56007715	56009490	1776	-1.4075	7.06E-27
CNVR_15	chrAC_000171.1	14748571	14750790	2220	1.4160	3.35E-45
CNVR_16	chrAC_000171.1	15023407	15025626	2220	1.4318	6.50E-46
CNVR_17	chrAC_000171.1	15061147	15062922	1776	1.7063	1.00E-46
CNVR_18	chrAC_000171.1	15333763	15335538	1776	1.5449	4.07E-41
CNVR_19	chrAC_000171.1	15391483	15393258	1776	1.3783	3.78E-35
CNVR_20	chrAC_000171.1	15403471	15405690	2220	1.2284	9.92E-37
CNVR_21	chrAC_000171.1	19012303	19014078	1776	1.2341	6.27E-30
CNVR_22	chrAC_000171.1	21181687	21184350	2664	1.2459	1.12E-44
CNVR_23	chrAC_000171.1	21185683	21187458	1776	1.1465	8.84E-27
CNVR_24	chrAC_000171.1	32433979	32435754	1776	1.2016	9.30E-29
CNVR_25	chrAC_000171.1	32511235	32513010	1776	1.2958	3.66E-32
CNVR_26	chrAC_000171.1	34699267	34701042	1776	1.4348	3.46E-37
CNVR_27	chrAC_000171.1	40995631	40997850	2220	2.4843	2.52E-86
CNVR_28	chrAC_000171.1	61043119	61044894	1776	1.4430	1.75E-37
CNVR_29	chrAC_000171.1	77832535	77834310	1776	1.5824	1.94E-42
CNVR_30	chrAC_000171.1	1274947	1276722	1776	-1.9721	2.75E-46
CNVR_31	chrAC_000171.1	1324675	1326450	1776	-1.2976	6.19E-28
CNVR_32	chrAC_000171.1	11430559	11432334	1776	-2.0396	6.78E-48
CNVR_33	chrAC_000171.1	14573191	14575854	2664	-1.6579	2.12E-56
CNVR_34	chrAC_000171.1	15276043	15278262	2220	-1.9926	4.98E-58
CNVR_35	chrAC_000171.1	15517135	15519798	2664	-1.2083	3.90E-37
CNVR_36	chrAC_000171.1	32295451	32297226	1776	-1.6646	2.27E-38
CNVR_37	chrAC_000171.1	32316763	32320758	3996	-2.1449	7.20E-111
CNVR_38	chrAC_000171.1	32464171	32466390	2220	-1.4474	6.29E-40
CNVR_39	chrAC_000171.1	32536543	32544978	8436	-3.3406	0.00E+00
CNVR_40	chrAC_000171.1	32548975	32559630	10656	-3.2849	0.00E+00

CNVR_41	chrAC_000171.1	32560075	32568510	8436	-3.1497	0.00E+00
CNVR_42	chrAC_000171.1	33266923	33268698	1776	-1.1186	1.10E-22
CNVR_43	chrAC_000171.1	33450295	33452958	2664	-2.6393	4.15E-89
CNVR_44	chrAC_000171.1	33678511	33680286	1776	-1.5933	2.07E-36
CNVR_45	chrAC_000171.1	34400455	34404006	3552	-1.9276	1.43E-88
CNVR_46	chrAC_000171.1	42907051	42908826	1776	-1.2279	6.88E-26
CNVR_47	chrAC_000171.1	53552395	53555946	3552	-1.7345	1.06E-78
CNVR_48	chrAC_000171.1	53575927	53577702	1776	-1.2300	5.96E-26
CNVR_49	chrAC_000171.1	59416747	59418522	1776	-1.6465	7.09E-38
CNVR_50	chrAC_000171.1	59998387	60000606	2220	-1.3559	1.27E-36
CNVR_51	chrAC_000171.1	60611551	60613770	2220	-1.4242	4.28E-39
CNVR_52	chrAC_000171.1	60917467	60919242	1776	-1.2081	2.63E-25
CNVR_53	chrAC_000171.1	62796919	62798694	1776	-1.1913	8.17E-25
CNVR_54	chrAC_000171.1	65904919	65906694	1776	-1.6579	3.45E-38
CNVR_55	chrAC_000171.1	69066643	69069306	2664	-1.8504	5.31E-64
CNVR_56	chrAC_000171.1	69711331	69713106	1776	-1.5465	4.20E-35
CNVR_57	chrAC_000171.1	77817439	77820102	2664	-1.8710	8.79E-65
CNVR_58	chrAC_000171.1	79424719	79426494	1776	-2.0402	6.54E-48
CNVR_59	chrAC_000171.1	79915339	79919334	3996	-1.4525	9.10E-71
CNVR_60	chrAC_000171.1	81509299	81511074	1776	-1.2073	2.77E-25
CNVR_61	chrAC_000171.1	81648715	81650490	1776	-2.7754	2.93E-62
CNVR_62	chrAC_000171.1	81966175	81967950	1776	-1.3596	9.59E-30
CNVR_63	chrAC_000171.1	82141999	82145550	3552	-1.5792	2.94E-70
CNVR_64	chrAC_000164.1	12103663	12105438	1776	1.1749	3.35E-35
CNVR_65	chrAC_000164.1	12121867	12124086	2220	1.2013	4.29E-45
CNVR_66	chrAC_000164.1	12141847	12143622	1776	1.6871	1.02E-58
CNVR_67	chrAC_000164.1	14802295	14804070	1776	1.4327	3.52E-47
CNVR_68	chrAC_000164.1	18527899	18532338	4440	1.7236	6.28E-148
CNVR_69	chrAC_000164.1	18533227	18543438	10212	1.6836	0.00E+00
CNVR_70	chrAC_000164.1	18546103	18559422	13320	1.5813	0.00E+00
CNVR_71	chrAC_000164.1	18559867	18562974	3108	1.4922	5.40E-86
CNVR_72	chrAC_000164.1	18637123	18638898	1776	1.1213	9.67E-33
CNVR_73	chrAC_000164.1	18894643	18897306	2664	1.4580	1.29E-71
CNVR_74	chrAC_000164.1	41848111	41850774	2664	1.4820	2.84E-73
CNVR_75	chrAC_000164.1	44009503	44012166	2664	1.4446	1.09E-70
CNVR_76	chrAC_000164.1	44014831	44017494	2664	1.3173	7.83E-62
CNVR_77	chrAC_000164.1	66697459	66699234	1776	1.6802	2.06E-58
CNVR_78	chrAC_000164.1	80155099	80156874	1776	1.7283	1.61E-60
CNVR_79	chrAC_000164.1	87830971	87834078	3108	2.7945	2.63E-169
CNVR_80	chrAC_000164.1	98141095	98143314	2220	1.2580	2.23E-48
CNVR_81	chrAC_000164.1	109602067	109603842	1776	1.1990	2.56E-36
CNVR_82	chrAC_000164.1	7989115	7991334	2220	-1.4010	4.92E-48
CNVR_83	chrAC_000164.1	10036399	10039062	2664	-1.3197	8.27E-53
CNVR_84	chrAC_000164.1	10048387	10050606	2220	-1.3674	1.71E-46

CNVR_85	chrAC_000164.1	10051939	10058154	6216	-1.3025	1.66E-118
CNVR_86	chrAC_000164.1	10065703	10069254	3552	-1.4198	3.92E-77
CNVR_87	chrAC_000164.1	10073695	10079022	5328	-1.4109	9.19E-114
CNVR_88	chrAC_000164.1	10083019	10085238	2220	-1.5956	9.55E-57
CNVR_89	chrAC_000164.1	10105219	10107438	2220	-1.1455	3.02E-36
CNVR_90	chrAC_000164.1	10111879	10116762	4884	-1.4734	1.16E-110
CNVR_91	chrAC_000164.1	10143847	10145622	1776	-1.4318	6.69E-40
CNVR_92	chrAC_000164.1	11596171	11597946	1776	-2.8861	1.92E-80
CNVR_93	chrAC_000164.1	11736919	11739138	2220	-1.4551	1.71E-50
CNVR_94	chrAC_000164.1	11746243	11748462	2220	-2.8388	5.82E-99
CNVR_95	chrAC_000164.1	11756455	11758674	2220	-1.3475	1.39E-45
CNVR_96	chrAC_000164.1	11764003	11766222	2220	-1.6194	8.68E-58
CNVR_97	chrAC_000164.1	11770219	11774658	4440	-1.2941	2.00E-84
CNVR_98	chrAC_000164.1	11779543	11781318	1776	-1.6915	4.55E-49
CNVR_99	chrAC_000164.1	11787979	11789754	1776	-1.0885	3.14E-27
CNVR_100	chrAC_000164.1	11790199	11794638	4440	-1.2420	1.28E-79
CNVR_101	chrAC_000164.1	11799523	11802186	2664	-1.3174	1.11E-52
CNVR_102	chrAC_000164.1	11803519	11807070	3552	-1.6029	3.41E-90
CNVR_103	chrAC_000164.1	11811067	11812842	1776	-1.2318	1.56E-32
CNVR_104	chrAC_000164.1	11815063	11822610	7548	-3.2538	0.00E+00
CNVR_105	chrAC_000164.1	11830603	11832378	1776	-1.1885	6.29E-31
CNVR_106	chrAC_000164.1	11898091	11902530	4440	-1.7026	8.06E-121
CNVR_107	chrAC_000164.1	11920291	11925618	5328	-1.7168	5.05E-146
CNVR_108	chrAC_000164.1	11926507	11933166	6660	-2.2538	6.59E-243
CNVR_109	chrAC_000164.1	11938051	11940270	2220	-1.2729	3.83E-42
CNVR_110	chrAC_000164.1	11958031	11966022	7992	-2.6378	0.00E+00
CNVR_111	chrAC_000164.1	12005983	12008202	2220	-4.7241	4.64E-125
CNVR_112	chrAC_000164.1	12013087	12022854	9768	-2.7722	0.00E+00
CNVR_113	chrAC_000164.1	12307903	12310566	2664	-1.7414	3.21E-75
CNVR_114	chrAC_000164.1	15020299	15023406	3108	-2.2262	3.82E-113
CNVR_115	chrAC_000164.1	15090007	15094002	3996	-1.2981	1.68E-76
CNVR_116	chrAC_000164.1	15102883	15107322	4440	-1.3672	3.84E-91
CNVR_117	chrAC_000164.1	15391483	15394146	2664	-2.0897	1.56E-91
CNVR_118	chrAC_000164.1	15396811	15400362	3552	-2.5858	1.04E-146
CNVR_119	chrAC_000164.1	18628687	18630906	2220	-1.8615	6.66E-68
CNVR_120	chrAC_000164.1	18801403	18804066	2664	-2.0253	1.07E-88
CNVR_121	chrAC_000164.1	21710047	21721146	11100	-1.8536	0.00E+00
CNVR_122	chrAC_000164.1	23935819	23937594	1776	-1.4329	6.13E-40
CNVR_123	chrAC_000164.1	24560083	24562746	2664	-1.4038	1.95E-57
CNVR_124	chrAC_000164.1	25603927	25605702	1776	-1.4564	8.60E-41
CNVR_125	chrAC_000164.1	27696943	27699162	2220	-2.4184	1.50E-87
CNVR_126	chrAC_000164.1	27786187	27788850	2664	-1.4914	3.43E-62
CNVR_127	chrAC_000164.1	29455183	29458290	3108	-1.8734	5.70E-95
CNVR_128	chrAC_000164.1	29459179	29460954	1776	-1.7295	2.35E-50

CNVR_129	chrAC_000164.1	35866543	35868318	1776	-1.8826	2.32E-55
CNVR_130	chrAC_000164.1	38231731	38233950	2220	-1.3567	5.28E-46
CNVR_131	chrAC_000164.1	38652199	38653974	1776	-1.1794	1.37E-30
CNVR_132	chrAC_000164.1	38654419	38656194	1776	-1.2686	6.74E-34
CNVR_133	chrAC_000164.1	41482699	41484474	1776	-1.2528	2.59E-33
CNVR_134	chrAC_000164.1	42739219	42740994	1776	-1.4225	1.46E-39
CNVR_135	chrAC_000164.1	42743215	42747210	3996	-1.4886	4.01E-92
CNVR_136	chrAC_000164.1	42752983	42755646	2664	-1.1076	4.69E-41
CNVR_137	chrAC_000164.1	42776959	42779178	2220	-1.6087	2.53E-57
CNVR_138	chrAC_000164.1	42780067	42783618	3552	-1.4042	5.38E-76
CNVR_139	chrAC_000164.1	42792055	42795162	3108	-1.8442	2.45E-93
CNVR_140	chrAC_000164.1	43093975	43095750	1776	-3.5433	1.88E-90
CNVR_141	chrAC_000164.1	43828351	43830126	1776	-1.4682	1.85E-108
CNVR_142	chrAC_000164.1	47615227	47617002	1776	-2.0898	1.20E-61
CNVR_143	chrAC_000164.1	49404103	49405878	1776	-1.3177	1.03E-35
CNVR_144	chrAC_000164.1	64487671	64489446	1776	-1.5125	8.21E-43
CNVR_145	chrAC_000164.1	64611547	64615542	3996	-2.9490	4.04E-181
CNVR_146	chrAC_000164.1	66284095	66286314	2220	-1.2538	2.93E-41
CNVR_147	chrAC_000164.1	66692131	66695238	3108	-1.7965	1.25E-90
CNVR_148	chrAC_000164.1	69050659	69053322	2664	-1.4752	2.58E-61
CNVR_149	chrAC_000164.1	70191295	70193070	1776	-1.1524	1.36E-29
CNVR_150	chrAC_000164.1	70203727	70208166	4440	-1.2769	7.66E-83
CNVR_151	chrAC_000164.1	70233031	70234806	1776	-1.3071	2.53E-35
CNVR_152	chrAC_000164.1	70240579	70242354	1776	-1.3173	1.07E-35
CNVR_153	chrAC_000164.1	70246795	70249458	2664	-1.4049	1.69E-57
CNVR_154	chrAC_000164.1	70252123	70257450	5328	-1.1902	2.02E-89
CNVR_155	chrAC_000164.1	70261003	70262778	1776	-1.1829	1.01E-30
CNVR_156	chrAC_000164.1	71948203	71950422	2220	-1.3176	3.32E-44
CNVR_157	chrAC_000164.1	74952751	74954970	2220	-2.9595	9.58E-102
CNVR_158	chrAC_000164.1	79126351	79148994	22644	-3.9502	0.00E+00
CNVR_159	chrAC_000164.1	79149883	79156542	6660	-2.9995	8.69E-304
CNVR_160	chrAC_000164.1	80448139	80455686	7548	-1.3421	1.18E-149
CNVR_161	chrAC_000164.1	83874043	83875818	1776	-1.2280	2.15E-32
CNVR_162	chrAC_000164.1	86680567	86682786	2220	-2.2235	2.82E-81
CNVR_163	chrAC_000164.1	87109471	87133890	24420	-3.3700	0.00E+00
CNVR_164	chrAC_000164.1	87571231	87575670	4440	-4.2538	2.02E-240
CNVR_165	chrAC_000164.1	87607195	87609414	2220	-2.5116	2.52E-90
CNVR_166	chrAC_000164.1	89594539	89596314	1776	-1.4874	6.51E-42
CNVR_167	chrAC_000164.1	98106463	98110902	4440	-3.5845	2.27E-224
CNVR_168	chrAC_000164.1	99784339	99787446	3108	-1.5275	1.65E-74
CNVR_169	chrAC_000164.1	101355211	101356986	1776	-1.3423	1.27E-36
CNVR_170	chrAC_000164.1	101444011	101446230	2220	-1.6924	6.33E-61
CNVR_171	chrAC_000164.1	103093471	103095246	1776	-1.4864	7.10E-42
CNVR_172	chrAC_000164.1	105497287	105499062	1776	-1.4873	6.60E-42

CNVR_173	chrAC_000164.1	105652687	105654462	1776	-1.1523	1.38E-29
CNVR_174	chrAC_000164.1	106389727	106391502	1776	-2.5223	6.94E-73
CNVR_175	chrAC_000164.1	107360755	107362530	1776	-2.5667	6.79E-74
CNVR_176	chrAC_000164.1	108215455	108217230	1776	-1.2625	1.14E-33
CNVR_177	chrAC_000175.1	14875	17982	3108	1.5376	1.33E-62
CNVR_178	chrAC_000175.1	18427	20202	1776	1.2008	8.82E-26
CNVR_179	chrAC_000175.1	683095	684870	1776	1.2998	5.91E-29
CNVR_180	chrAC_000175.1	990343	992562	2220	2.0333	2.57E-63
CNVR_181	chrAC_000175.1	50901715	50903934	2220	1.2915	1.94E-35
CNVR_182	chrAC_000175.1	61549279	61551054	1776	1.3636	5.29E-31
CNVR_183	chrAC_000175.1	61662943	61664718	1776	1.3300	6.36E-30
CNVR_184	chrAC_000175.1	61666495	61675374	8880	1.7319	6.12E-205
CNVR_185	chrAC_000175.1	61677595	61679370	1776	2.3537	6.39E-59
CNVR_186	chrAC_000175.1	61713115	61714890	1776	1.1801	4.04E-25
CNVR_187	chrAC_000175.1	61717555	61719330	1776	1.1996	9.68E-26
CNVR_188	chrAC_000175.1	61731319	61733538	2220	1.2282	6.64E-33
CNVR_189	chrAC_000175.1	61736647	61738422	1776	1.2136	3.45E-26
CNVR_190	chrAC_000175.1	61774831	61776606	1776	1.3720	2.84E-31
CNVR_191	chrAC_000175.1	61790815	61792590	1776	1.2238	1.62E-26
CNVR_192	chrAC_000175.1	61809463	61811682	2220	1.1468	1.15E-29
CNVR_193	chrAC_000175.1	61815679	61817898	2220	1.2946	1.46E-35
CNVR_194	chrAC_000175.1	61826335	61831662	5328	1.3484	5.30E-88
CNVR_195	chrAC_000175.1	61868071	61872954	4884	1.4495	1.12E-89
CNVR_196	chrAC_000175.1	61874731	61876506	1776	1.3787	1.74E-31
CNVR_197	chrAC_000175.1	61883167	61884942	1776	1.2189	2.33E-26
CNVR_198	chrAC_000175.1	61888495	61891602	3108	1.4984	1.89E-60
CNVR_199	chrAC_000175.1	61894267	61896486	2220	1.1873	2.85E-31
CNVR_200	chrAC_000175.1	61896931	61898706	1776	1.3441	2.24E-30
CNVR_201	chrAC_000175.1	61902703	61905366	2664	1.5216	3.63E-53
CNVR_202	chrAC_000175.1	61906255	61908474	2220	1.5165	2.11E-44
CNVR_203	chrAC_000175.1	61912915	61914690	1776	1.1915	1.76E-25
CNVR_204	chrAC_000175.1	63508651	63510870	2220	1.2815	4.89E-35
CNVR_205	chrAC_000175.1	63513535	63515310	1776	1.2805	2.47E-28
CNVR_206	chrAC_000175.1	63520195	63529074	8880	1.4156	4.61E-156
CNVR_207	chrAC_000175.1	63532183	63533958	1776	1.3326	5.24E-30
CNVR_208	chrAC_000175.1	63545503	63553050	7548	1.2323	4.08E-108
CNVR_209	chrAC_000175.1	63558379	63565482	7104	1.2200	2.57E-100
CNVR_210	chrAC_000175.1	63579691	63581466	1776	1.1443	5.55E-24
CNVR_211	chrAC_000175.1	685759	687534	1776	-2.0327	9.18E-44
CNVR_212	chrAC_000175.1	698191	700854	2664	-1.9192	5.53E-61
CNVR_213	chrAC_000175.1	707515	709290	1776	-2.4427	8.76E-52
CNVR_214	chrAC_000175.1	4397599	4399818	2220	-1.2598	2.51E-30
CNVR_215	chrAC_000175.1	4434895	4437558	2664	-1.4211	1.69E-42
CNVR_216	chrAC_000175.1	13157275	13160382	3108	-1.4882	2.29E-52

CNVR_217	chrAC_000175.1	14714827	14716602	1776	-1.3659	1.96E-27
CNVR_218	chrAC_000175.1	15596167	15599274	3108	-1.8246	5.12E-67
CNVR_219	chrAC_000175.1	22094107	22095882	1776	-1.3111	5.71E-26
CNVR_220	chrAC_000175.1	22098991	22101654	2664	-1.2902	2.82E-37
CNVR_221	chrAC_000175.1	22104319	22106982	2664	-1.3228	1.39E-38
CNVR_222	chrAC_000175.1	27029611	27031830	2220	-1.3092	5.53E-32
CNVR_223	chrAC_000175.1	30697495	30699714	2220	-1.6740	7.94E-44
CNVR_224	chrAC_000175.1	30802723	30805830	3108	-1.6421	2.98E-59
CNVR_225	chrAC_000175.1	32179123	32180898	1776	-1.2715	6.61E-25
CNVR_226	chrAC_000175.1	48267907	48270126	2220	-1.2415	1.03E-29
CNVR_227	chrAC_000175.1	48370027	48373134	3108	-1.4838	3.63E-52
CNVR_228	chrAC_000175.1	48373579	48375798	2220	-1.2474	6.52E-30
CNVR_229	chrAC_000175.1	48378019	48380682	2664	-1.9576	2.81E-62
CNVR_230	chrAC_000175.1	48387787	48390006	2220	-1.6312	1.72E-42
CNVR_231	chrAC_000175.1	49710463	49713570	3108	-2.2719	9.25E-84
CNVR_232	chrAC_000175.1	50911039	50914590	3552	-1.5305	8.81E-62
CNVR_233	chrAC_000175.1	50921695	50923470	1776	-1.4569	7.65E-30
CNVR_234	chrAC_000175.1	50935015	50937234	2220	-1.4547	8.22E-37
CNVR_235	chrAC_000175.1	51023371	51028698	5328	-1.5155	1.43E-90
CNVR_236	chrAC_000175.1	51040687	51045570	4884	-2.0185	2.79E-116
CNVR_237	chrAC_000175.1	51053563	51058446	4884	-1.3698	1.10E-72
CNVR_238	chrAC_000175.1	51067327	51069102	1776	-1.2981	1.28E-25
CNVR_239	chrAC_000175.1	51091747	51100626	8880	-2.0301	5.14E-211
CNVR_240	chrAC_000175.1	51749755	51751530	1776	-1.2298	8.74E-24
CNVR_241	chrAC_000175.1	51755083	51757302	2220	-1.3522	2.03E-33
CNVR_242	chrAC_000175.1	52775839	52777614	1776	-1.2222	1.39E-23
CNVR_243	chrAC_000175.1	52783831	52785606	1776	-1.5133	2.57E-31
CNVR_244	chrAC_000175.1	56400655	56402430	1776	-1.1023	2.30E-20
CNVR_245	chrAC_000175.1	57565711	57571926	6216	-1.4167	2.53E-96
CNVR_246	chrAC_000175.1	58569151	58571814	2664	-1.2935	2.07E-37
CNVR_247	chrAC_000175.1	59650291	59652510	2220	-1.2797	5.39E-31
CNVR_248	chrAC_000175.1	60529411	60531186	1776	-1.5159	2.20E-31
CNVR_249	chrAC_000175.1	60532519	60541398	8880	-1.4893	2.95E-146
CNVR_250	chrAC_000175.1	60554719	60559158	4440	-1.2417	1.05E-57
CNVR_251	chrAC_000175.1	60580027	60583578	3552	-1.1627	2.68E-42
CNVR_252	chrAC_000175.1	60596011	60606222	10212	-1.3035	9.57E-140
CNVR_253	chrAC_000175.1	60606667	60608886	2220	-1.1705	2.47E-27
CNVR_254	chrAC_000175.1	60635971	60638634	2664	-1.2881	3.43E-37
CNVR_255	chrAC_000175.1	60645295	60647070	1776	-1.2940	1.65E-25
CNVR_256	chrAC_000175.1	61620763	61622982	2220	-2.7496	1.93E-70
CNVR_257	chrAC_000175.1	63179647	63182754	3108	-1.6848	4.11E-61
CNVR_258	chrAC_000175.1	63224491	63226266	1776	-1.5884	2.99E-33
CNVR_259	chrAC_000175.1	63235147	63237366	2220	-1.1632	4.35E-27
CNVR_260	chrAC_000175.1	63237811	63245358	7548	-1.4655	5.48E-122

CNVR_261	chrAC_000175.1	63259123	63260898	1776	-1.1894	1.07E-22
CNVR_262	chrAC_000175.1	63341707	63343482	1776	-1.3058	7.94E-26
CNVR_263	chrAC_000175.1	63800803	63803022	2220	-1.6823	4.40E-44
CNVR_264	chrAC_000175.1	63960643	63963750	3108	-2.8526	2.02E-100
CNVR_265	chrAC_000175.1	65998603	66000822	2220	-1.1745	1.82E-27
CNVR_266	chrAC_000166.1	15319	17982	2664	1.0644	2.53E-28
CNVR_267	chrAC_000166.1	26780971	26783190	2220	1.2891	6.51E-32
CNVR_268	chrAC_000166.1	61107943	61110162	2220	1.5185	3.67E-40
CNVR_269	chrAC_000166.1	71551711	71553486	1776	1.5729	5.19E-34
CNVR_270	chrAC_000166.1	82649935	82651710	1776	1.5185	1.80E-32
CNVR_271	chrAC_000166.1	105629155	105630930	1776	1.2991	3.75E-26
CNVR_272	chrAC_000166.1	1057387	1059162	1776	-1.3217	2.46E-23
CNVR_273	chrAC_000166.1	2170051	2171826	1776	-1.5734	3.77E-29
CNVR_274	chrAC_000166.1	2349427	2351646	2220	-1.5567	1.51E-35
CNVR_275	chrAC_000166.1	2672659	2674434	1776	-1.7914	6.77E-34
CNVR_276	chrAC_000166.1	3231655	3233430	1776	-1.2568	8.37E-22
CNVR_277	chrAC_000166.1	3418135	3422574	4440	-1.5240	1.79E-67
CNVR_278	chrAC_000166.1	3425239	3428346	3108	-1.3803	5.50E-42
CNVR_279	chrAC_000166.1	3431899	3434562	2664	-1.4361	3.53E-38
CNVR_280	chrAC_000166.1	3436339	3438114	1776	-1.3145	3.63E-23
CNVR_281	chrAC_000166.1	3637027	3638802	1776	-2.1111	3.52E-40
CNVR_282	chrAC_000166.1	3646795	3649458	2664	-1.3853	2.08E-36
CNVR_283	chrAC_000166.1	3901207	3903426	2220	-1.4328	5.28E-32
CNVR_284	chrAC_000166.1	3967807	3969582	1776	-1.3190	2.85E-23
CNVR_285	chrAC_000166.1	4176487	4178706	2220	-1.6772	6.89E-39
CNVR_286	chrAC_000166.1	4503715	4508598	4884	-1.3001	8.36E-60
CNVR_287	chrAC_000166.1	4672435	4674210	1776	-1.2785	2.57E-22
CNVR_288	chrAC_000166.1	5662555	5664330	1776	-1.5490	1.34E-28
CNVR_289	chrAC_000166.1	7533571	7535790	2220	-1.2014	3.25E-25
CNVR_290	chrAC_000166.1	7800415	7802634	2220	-1.6414	6.57E-38
CNVR_291	chrAC_000166.1	7916743	7918518	1776	-1.3494	5.47E-24
CNVR_292	chrAC_000166.1	8725267	8727042	1776	-1.7891	7.56E-34
CNVR_293	chrAC_000166.1	9957811	9959586	1776	-1.7733	1.63E-33
CNVR_294	chrAC_000166.1	10258843	10260618	1776	-1.2514	1.12E-21
CNVR_295	chrAC_000166.1	12210223	12212442	2220	-1.7224	4.13E-40
CNVR_296	chrAC_000166.1	13012531	13014306	1776	-1.2658	5.12E-22
CNVR_297	chrAC_000166.1	14240635	14243298	2664	-1.6770	2.61E-46
CNVR_298	chrAC_000166.1	14527015	14529234	2220	-1.3729	2.92E-30
CNVR_299	chrAC_000166.1	15257839	15259614	1776	-1.2321	3.21E-21
CNVR_300	chrAC_000166.1	16519243	16521018	1776	-1.2570	8.30E-22
CNVR_301	chrAC_000166.1	19739131	19741350	2220	-1.2112	1.68E-25
CNVR_302	chrAC_000166.1	25118635	25123962	5328	-1.7513	1.43E-95
CNVR_303	chrAC_000166.1	25270039	25271814	1776	-2.4464	8.14E-46
CNVR_304	chrAC_000166.1	25273591	25275810	2220	-3.5452	2.97E-72

CNVR_305	chrAC_000166.1	26146495	26149158	2664	-1.5885	2.22E-43
CNVR_306	chrAC_000166.1	28133395	28135170	1776	-1.7795	1.20E-33
CNVR_307	chrAC_000166.1	29177239	29179458	2220	-1.3746	2.61E-30
CNVR_308	chrAC_000166.1	30925267	30928818	3552	-1.6255	6.84E-59
CNVR_309	chrAC_000166.1	32833579	32835354	1776	-1.4842	4.03E-27
CNVR_310	chrAC_000166.1	36453067	36457506	4440	-1.4190	1.85E-61
CNVR_311	chrAC_000166.1	37954231	37956006	1776	-1.6474	8.49E-31
CNVR_312	chrAC_000166.1	38189107	38191770	2664	-1.3934	1.08E-36
CNVR_313	chrAC_000166.1	43560619	43562838	2220	-1.8544	1.43E-43
CNVR_314	chrAC_000166.1	44007283	44013054	5772	-2.7694	2.29E-159
CNVR_315	chrAC_000166.1	46344055	46346274	2220	-1.4390	3.49E-32
CNVR_316	chrAC_000166.1	49470703	49474698	3996	-1.3723	4.65E-53
CNVR_317	chrAC_000166.1	49475143	49479138	3996	-1.5255	5.46E-61
CNVR_318	chrAC_000166.1	49587031	49588806	1776	-1.2998	8.07E-23
CNVR_319	chrAC_000166.1	53242039	53244258	2220	-1.2779	1.79E-27
CNVR_320	chrAC_000166.1	56134255	56136030	1776	-1.7337	1.13E-32
CNVR_321	chrAC_000166.1	56307415	56309634	2220	-1.6401	7.14E-38
CNVR_322	chrAC_000166.1	56816683	56818458	1776	-1.3805	1.02E-24
CNVR_323	chrAC_000166.1	58250359	58252134	1776	-1.1554	2.10E-19
CNVR_324	chrAC_000166.1	58972747	58974522	1776	-1.4910	2.80E-27
CNVR_325	chrAC_000166.1	59165443	59167218	1776	-1.1877	3.62E-20
CNVR_326	chrAC_000166.1	59656063	59657838	1776	-1.1310	7.92E-19
CNVR_327	chrAC_000166.1	63587683	63589902	2220	-1.8254	7.97E-43
CNVR_328	chrAC_000166.1	63590347	63595230	4884	-2.3810	6.08E-120
CNVR_329	chrAC_000166.1	64355803	64360686	4884	-1.4535	1.30E-69
CNVR_330	chrAC_000166.1	64514755	64521414	6660	-2.0412	9.63E-141
CNVR_331	chrAC_000166.1	65012479	65015142	2664	-3.5048	7.53E-86
CNVR_332	chrAC_000166.1	67436275	67438050	1776	-1.2121	9.57E-21
CNVR_333	chrAC_000166.1	68369563	68371782	2220	-1.7596	4.21E-41
CNVR_334	chrAC_000166.1	71411851	71414958	3108	-1.4667	1.74E-45
CNVR_335	chrAC_000166.1	78969175	78970950	1776	-1.3807	1.01E-24
CNVR_336	chrAC_000166.1	79022899	79026450	3552	-2.5485	7.70E-93
CNVR_337	chrAC_000166.1	80041879	80043654	1776	-1.3861	7.56E-25
CNVR_338	chrAC_000166.1	82661035	82662810	1776	-1.8920	5.76E-36
CNVR_339	chrAC_000166.1	82672579	82674354	1776	-1.1487	3.02E-19
CNVR_340	chrAC_000166.1	82684567	82687230	2664	-1.6184	2.22E-44
CNVR_341	chrAC_000166.1	85047535	85049310	1776	-2.2634	7.34E-43
CNVR_342	chrAC_000166.1	85781023	85782798	1776	-1.3742	1.44E-24
CNVR_343	chrAC_000166.1	85801447	85805442	3996	-1.4529	2.94E-57
CNVR_344	chrAC_000166.1	85808551	85810326	1776	-1.3469	6.29E-24
CNVR_345	chrAC_000166.1	85814323	85817430	3108	-1.5024	6.47E-47
CNVR_346	chrAC_000166.1	85819207	85821870	2664	-1.3665	9.43E-36
CNVR_347	chrAC_000166.1	85827643	85829862	2220	-1.4312	5.87E-32
CNVR_348	chrAC_000166.1	85839187	85841406	2220	-1.0680	2.71E-21

CNVR_349	chrAC_000166.1	85852507	85855170	2664	-1.1736	6.08E-29
CNVR_350	chrAC_000166.1	85869379	85871598	2220	-1.1840	1.06E-24
CNVR_351	chrAC_000166.1	85947967	85955514	7548	-1.4880	6.74E-110
CNVR_352	chrAC_000166.1	85955959	85958622	2664	-1.4434	1.97E-38
CNVR_353	chrAC_000166.1	85962619	85964394	1776	-1.3372	1.06E-23
CNVR_354	chrAC_000166.1	88150207	88152870	2664	-1.3748	4.83E-36
CNVR_355	chrAC_000166.1	88207039	88214586	7548	-1.3928	1.38E-100
CNVR_356	chrAC_000166.1	88215031	88217250	2220	-1.1919	6.19E-25
CNVR_357	chrAC_000166.1	88220803	88223022	2220	-1.8414	3.08E-43
CNVR_358	chrAC_000166.1	88229683	88236342	6660	-1.2795	4.64E-79
CNVR_359	chrAC_000166.1	88241227	88243890	2664	-1.4081	3.32E-37
CNVR_360	chrAC_000166.1	88246111	88248330	2220	-1.8347	4.59E-43
CNVR_361	chrAC_000166.1	88255879	88258542	2664	-1.4822	8.99E-40
CNVR_362	chrAC_000166.1	88260763	88262538	1776	-1.1916	2.92E-20
CNVR_363	chrAC_000166.1	88274083	88277634	3552	-1.2432	2.65E-41
CNVR_364	chrAC_000166.1	88290511	88296282	5772	-1.7954	2.19E-106
CNVR_365	chrAC_000166.1	88300279	88312710	12432	-1.9786	1.42E-252
CNVR_366	chrAC_000166.1	88313155	88315374	2220	-1.7976	4.21E-42
CNVR_367	chrAC_000166.1	88317151	88319370	2220	-1.2743	2.30E-27
CNVR_368	chrAC_000166.1	88322035	88325586	3552	-1.2913	1.44E-43
CNVR_369	chrAC_000166.1	88406395	88409058	2664	-1.7318	4.38E-48
CNVR_370	chrAC_000166.1	88411723	88416162	4440	-1.6305	1.98E-73
CNVR_371	chrAC_000166.1	88416607	88427262	10656	-3.8567	0.00E+00
CNVR_372	chrAC_000166.1	88786903	88790898	3996	-1.5062	5.30E-60
CNVR_373	chrAC_000166.1	91067731	91069506	1776	-1.2585	7.62E-22
CNVR_374	chrAC_000166.1	91574779	91576554	1776	-1.9701	1.63E-37
CNVR_375	chrAC_000166.1	93565231	93567006	1776	-1.9603	2.53E-37
CNVR_376	chrAC_000166.1	99702199	99703974	1776	-1.3626	2.68E-24
CNVR_377	chrAC_000166.1	104733607	104735382	1776	-2.9172	1.27E-69
CNVR_378	chrAC_000166.1	105697087	105701526	4440	-1.1203	5.67E-44
CNVR_379	chrAC_000172.1	3757795	3760458	2664	1.8387	2.34E-57
CNVR_380	chrAC_000172.1	12053047	12054822	1776	1.8185	2.50E-38
CNVR_381	chrAC_000172.1	12055267	12057042	1776	1.5252	9.89E-31
CNVR_382	chrAC_000172.1	42190435	42192210	1776	1.3781	9.53E-27
CNVR_383	chrAC_000172.1	46478143	46480362	2220	1.3264	2.97E-31
CNVR_384	chrAC_000172.1	46554955	46563390	8436	1.8207	2.21E-175
CNVR_385	chrAC_000172.1	46580263	46582038	1776	1.3686	1.73E-26
CNVR_386	chrAC_000172.1	46949227	46951890	2664	1.3795	2.45E-39
CNVR_387	chrAC_000172.1	47991295	47993070	1776	1.1930	1.07E-21
CNVR_388	chrAC_000172.1	48011275	48013494	2220	1.1035	1.03E-23
CNVR_389	chrAC_000172.1	48072991	48075210	2220	1.3095	1.11E-30
CNVR_390	chrAC_000172.1	48467263	48469482	2220	1.1161	3.94E-24
CNVR_391	chrAC_000172.1	50732107	50733882	1776	1.5502	2.11E-31
CNVR_392	chrAC_000172.1	50832895	50834670	1776	2.0185	4.19E-43

CNVR_393	chrAC_000172.1	51085087	51087306	2220	2.0334	7.33E-54
CNVR_394	chrAC_000172.1	57676267	57679374	3108	1.5849	2.93E-55
CNVR_395	chrAC_000172.1	57765955	57767730	1776	1.1091	1.95E-19
CNVR_396	chrAC_000172.1	57769063	57772170	3108	1.7354	4.29E-62
CNVR_397	chrAC_000172.1	78024787	78026562	1776	1.6570	3.21E-34
CNVR_398	chrAC_000172.1	78027007	78029670	2664	1.5770	2.62E-47
CNVR_399	chrAC_000172.1	78332035	78341358	9324	2.1749	2.11E-236
CNVR_400	chrAC_000172.1	79081951	79084170	2220	2.4115	1.08E-63
CNVR_401	chrAC_000172.1	81000031	81001806	1776	1.0514	6.61E-18
CNVR_402	chrAC_000172.1	83768371	83770146	1776	1.2755	6.08E-24
CNVR_403	chrAC_000172.1	674659	676434	1776	-1.3549	4.03E-22
CNVR_404	chrAC_000172.1	1856587	1858806	2220	-1.1119	9.57E-21
CNVR_405	chrAC_000172.1	2158063	2159838	1776	-1.4750	1.17E-24
CNVR_406	chrAC_000172.1	4283935	4285710	1776	-2.8550	7.59E-47
CNVR_407	chrAC_000172.1	5046283	5048058	1776	-1.5035	2.99E-25
CNVR_408	chrAC_000172.1	5266507	5268282	1776	-1.3653	2.42E-22
CNVR_409	chrAC_000172.1	6114547	6116322	1776	-1.2349	1.50E-19
CNVR_410	chrAC_000172.1	7224103	7226766	2664	-1.2060	1.37E-27
CNVR_411	chrAC_000172.1	7341319	7343982	2664	-1.2696	1.24E-29
CNVR_412	chrAC_000172.1	7347091	7348866	1776	-1.3999	4.45E-23
CNVR_413	chrAC_000172.1	8646235	8652894	6660	-2.1655	7.46E-136
CNVR_414	chrAC_000172.1	8699515	8702178	2664	-1.2086	1.13E-27
CNVR_415	chrAC_000172.1	9736255	9745578	9324	-2.9045	1.18E-240
CNVR_416	chrAC_000172.1	19681855	19683630	1776	-1.4774	1.04E-24
CNVR_417	chrAC_000172.1	34065679	34067454	1776	-1.1280	2.98E-17
CNVR_418	chrAC_000172.1	35747107	35748882	1776	-1.3597	3.19E-22
CNVR_419	chrAC_000172.1	35749771	35754210	4440	-2.6374	5.42E-108
CNVR_420	chrAC_000172.1	41508451	41510226	1776	-1.5023	3.16E-25
CNVR_421	chrAC_000172.1	41792167	41793942	1776	-1.8649	2.29E-32
CNVR_422	chrAC_000172.1	43501567	43503342	1776	-1.4140	2.25E-23
CNVR_423	chrAC_000172.1	45107071	45109290	2220	-2.9476	2.44E-59
CNVR_424	chrAC_000172.1	45290887	45293106	2220	-3.0512	1.23E-60
CNVR_425	chrAC_000172.1	45490687	45493350	2664	-1.3306	1.38E-31
CNVR_426	chrAC_000172.1	45540415	45542190	1776	-1.2198	3.17E-19
CNVR_427	chrAC_000172.1	45884959	45887622	2664	-1.8137	2.95E-46
CNVR_428	chrAC_000172.1	46471039	46473258	2220	-1.1220	5.17E-21
CNVR_429	chrAC_000172.1	46523431	46525206	1776	-1.7526	3.02E-30
CNVR_430	chrAC_000172.1	46550515	46552290	1776	-2.1598	1.77E-37
CNVR_431	chrAC_000172.1	46564279	46566942	2664	-1.5721	3.77E-39
CNVR_432	chrAC_000172.1	46567831	46571382	3552	-2.5872	1.64E-85
CNVR_433	chrAC_000172.1	46572715	46574490	1776	-1.3049	4.72E-21
CNVR_434	chrAC_000172.1	46768075	46769850	1776	-1.3251	1.75E-21
CNVR_435	chrAC_000172.1	46774291	46776954	2664	-1.5018	5.49E-37
CNVR_436	chrAC_000172.1	46783171	46784946	1776	-1.4020	4.03E-23

CNVR_437	chrAC_000172.1	46807591	46809366	1776	-2.0602	7.91E-36
CNVR_438	chrAC_000172.1	46824019	46825794	1776	-1.5791	8.34E-27
CNVR_439	chrAC_000172.1	46827127	46835118	7992	-2.9144	4.75E-207
CNVR_440	chrAC_000172.1	46836007	46837782	1776	-1.7408	5.11E-30
CNVR_441	chrAC_000172.1	46844443	46846218	1776	-2.0352	2.11E-35
CNVR_442	chrAC_000172.1	46849771	46851546	1776	-1.1280	2.98E-17
CNVR_443	chrAC_000172.1	46862647	46866198	3552	-1.5137	3.18E-49
CNVR_444	chrAC_000172.1	46875079	46877298	2220	-1.2804	2.94E-25
CNVR_445	chrAC_000172.1	46877743	46892838	15096	-2.2984	0.00E+00
CNVR_446	chrAC_000172.1	46895503	46908822	13320	-3.3066	0.00E+00
CNVR_447	chrAC_000172.1	46909711	46922142	12432	-2.3726	7.18E-274
CNVR_448	chrAC_000172.1	47819467	47841666	22200	-3.8489	0.00E+00
CNVR_449	chrAC_000172.1	47842555	47849214	6660	-2.1938	1.52E-137
CNVR_450	chrAC_000172.1	47850103	47851878	1776	-1.7770	1.03E-30
CNVR_451	chrAC_000172.1	47882515	47884290	1776	-1.6005	3.07E-27
CNVR_452	chrAC_000172.1	47920699	47922474	1776	-1.5325	7.51E-26
CNVR_453	chrAC_000172.1	47958439	47961546	3108	-2.3108	2.33E-68
CNVR_454	chrAC_000172.1	48026815	48028590	1776	-1.7096	2.06E-29
CNVR_455	chrAC_000172.1	48725671	48727446	1776	-2.8104	2.37E-46
CNVR_456	chrAC_000172.1	48728335	48730554	2220	-1.4949	6.30E-31
CNVR_457	chrAC_000172.1	48745651	48752310	6660	-3.2893	4.55E-186
CNVR_458	chrAC_000172.1	49117723	49119498	1776	-1.6202	1.23E-27
CNVR_459	chrAC_000172.1	49829455	49831230	1776	-1.2460	8.65E-20
CNVR_460	chrAC_000172.1	49941343	49943118	1776	-2.2234	1.73E-38
CNVR_461	chrAC_000172.1	50056783	50058558	1776	-1.3713	1.80E-22
CNVR_462	chrAC_000172.1	50062555	50064330	1776	-1.2389	1.23E-19
CNVR_463	chrAC_000172.1	50066551	50068326	1776	-1.1432	1.41E-17
CNVR_464	chrAC_000172.1	50630875	50632650	1776	-3.0649	5.31E-49
CNVR_465	chrAC_000172.1	50716567	50718786	2220	-2.9557	1.91E-59
CNVR_466	chrAC_000172.1	50799151	50800926	1776	-1.4100	2.73E-23
CNVR_467	chrAC_000172.1	50802259	50804034	1776	-1.6823	7.09E-29
CNVR_468	chrAC_000172.1	51089083	51094410	5328	-2.3317	9.33E-117
CNVR_469	chrAC_000172.1	51450943	51452718	1776	-1.7129	1.78E-29
CNVR_470	chrAC_000172.1	52264351	52276338	11988	-2.5935	2.21E-284
CNVR_471	chrAC_000172.1	52278115	52280334	2220	-1.2019	3.74E-23
CNVR_472	chrAC_000172.1	52556947	52559610	2664	-2.6850	1.98E-66
CNVR_473	chrAC_000172.1	57220279	57223386	3108	-2.1194	4.18E-63
CNVR_474	chrAC_000172.1	57478243	57480462	2220	-1.8605	6.27E-40
CNVR_475	chrAC_000172.1	59697799	59699574	1776	-1.5501	3.27E-26
CNVR_476	chrAC_000172.1	66162883	66165990	3108	-2.9211	7.50E-82
CNVR_477	chrAC_000172.1	66543391	66545166	1776	-1.9537	5.61E-34
CNVR_478	chrAC_000172.1	68675923	68677698	1776	-1.3367	9.82E-22
CNVR_479	chrAC_000172.1	69294859	69298410	3552	-1.3219	3.55E-41
CNVR_480	chrAC_000172.1	69975955	69977730	1776	-1.7475	3.78E-30

CNVR_481	chrAC_000172.1	70164211	70170426	6216	-2.3608	2.25E-137
CNVR_482	chrAC_000172.1	71019799	71022018	2220	-2.2609	2.12E-48
CNVR_483	chrAC_000172.1	72053431	72056538	3108	-1.4137	1.20E-39
CNVR_484	chrAC_000172.1	73677583	73679802	2220	-1.8172	6.40E-39
CNVR_485	chrAC_000172.1	79533055	79536162	3108	-1.4453	8.34E-41
CNVR_486	chrAC_000172.1	79600099	79602318	2220	-1.7483	2.78E-37
CNVR_487	chrAC_000172.1	79717315	79720422	3108	-1.4540	4.02E-41
CNVR_488	chrAC_000172.1	80790019	80794458	4440	-1.4747	4.61E-59
CNVR_489	chrAC_000172.1	80795347	80798898	3552	-1.4230	1.88E-45
CNVR_490	chrAC_000172.1	80801119	80803782	2664	-1.3483	3.77E-32
CNVR_491	chrAC_000172.1	80804671	80807334	2664	-1.8174	2.31E-46
CNVR_492	chrAC_000172.1	81888919	81890694	1776	-1.7904	5.70E-31
CNVR_493	chrAC_000172.1	81901795	81904014	2220	-1.4313	2.90E-29
CNVR_494	chrAC_000172.1	82859059	82861278	2220	-1.4743	2.16E-30
CNVR_495	chrAC_000168.1	386059	388278	2220	1.4439	3.83E-41
CNVR_496	chrAC_000168.1	5351755	5355306	3552	1.9079	1.11E-92
CNVR_497	chrAC_000168.1	5419687	5421462	1776	1.8784	1.99E-46
CNVR_498	chrAC_000168.1	9595951	9597726	1776	1.3146	3.94E-29
CNVR_499	chrAC_000168.1	29696719	29698494	1776	1.3640	1.04E-30
CNVR_500	chrAC_000168.1	39930919	39932694	1776	1.5726	2.65E-37
CNVR_501	chrAC_000168.1	87374983	87378090	3108	1.2185	2.44E-44
CNVR_502	chrAC_000168.1	2742811	2745030	2220	-1.4204	2.80E-34
CNVR_503	chrAC_000168.1	9717163	9718938	1776	-1.8630	2.60E-38
CNVR_504	chrAC_000168.1	11396815	11398590	1776	-2.0208	1.09E-41
CNVR_505	chrAC_000168.1	14146951	14148726	1776	-1.9065	2.89E-39
CNVR_506	chrAC_000168.1	14152279	14154498	2220	-2.1112	5.88E-54
CNVR_507	chrAC_000168.1	17270491	17272266	1776	-3.1392	4.17E-59
CNVR_508	chrAC_000168.1	17369059	17371722	2664	-1.3148	8.89E-37
CNVR_509	chrAC_000168.1	17381491	17383266	1776	-1.8538	4.15E-38
CNVR_510	chrAC_000168.1	18095443	18097218	1776	-2.3043	3.60E-47
CNVR_511	chrAC_000168.1	19152163	19154382	2220	-2.3895	2.21E-60
CNVR_512	chrAC_000168.1	24252391	24254166	1776	-1.3433	8.37E-26
CNVR_513	chrAC_000168.1	24326983	24328758	1776	-1.2648	8.58E-24
CNVR_514	chrAC_000168.1	24636007	24637782	1776	-1.7466	1.12E-35
CNVR_515	chrAC_000168.1	30271699	30273918	2220	-1.3567	2.90E-32
CNVR_516	chrAC_000168.1	32754103	32755878	1776	-1.3554	4.14E-26
CNVR_517	chrAC_000168.1	33800611	33803718	3108	-3.0645	1.46E-100
CNVR_518	chrAC_000168.1	36097867	36100086	2220	-1.4871	2.28E-36
CNVR_519	chrAC_000168.1	36129391	36131166	1776	-1.3678	1.99E-26
CNVR_520	chrAC_000168.1	36261703	36263478	1776	-3.0685	2.60E-58
CNVR_521	chrAC_000168.1	39372367	39375474	3108	-2.6926	3.73E-92
CNVR_522	chrAC_000168.1	47059783	47062002	2220	-1.4942	1.37E-36
CNVR_523	chrAC_000168.1	47082427	47087754	5328	-2.6561	5.63E-155
CNVR_524	chrAC_000168.1	47103295	47112618	9324	-2.1419	1.22E-223

CNVR_525	chrAC_000168.1	50114503	50119386	4884	-2.3419	2.69E-128
CNVR_526	chrAC_000168.1	50119831	50121606	1776	-1.3699	1.76E-26
CNVR_527	chrAC_000168.1	52964983	52966758	1776	-1.1954	5.19E-22
CNVR_528	chrAC_000168.1	53502223	53503998	1776	-1.6425	3.14E-33
CNVR_529	chrAC_000168.1	56478355	56481906	3552	-1.9022	1.50E-76
CNVR_530	chrAC_000168.1	57904039	57907146	3108	-1.6500	3.74E-57
CNVR_531	chrAC_000168.1	58199743	58201518	1776	-2.1459	3.34E-44
CNVR_532	chrAC_000168.1	58220167	58221942	1776	-1.3674	2.04E-26
CNVR_533	chrAC_000168.1	59680927	59684034	3108	-1.4404	3.56E-48
CNVR_534	chrAC_000168.1	76030783	76033002	2220	-1.3305	1.98E-31
CNVR_535	chrAC_000168.1	84956071	84958290	2220	-1.6090	4.21E-40
CNVR_536	chrAC_000168.1	91045087	91047306	2220	-1.2546	5.25E-29
CNVR_537	chrAC_000168.1	93531487	93533262	1776	-1.5423	8.50E-31
CNVR_538	chrAC_000168.1	93827191	93832962	5772	-3.6738	7.34E-204
CNVR_539	chrAC_000168.1	93833851	93839178	5328	-4.5589	7.63E-204
CNVR_540	chrAC_000168.1	99358543	99360318	1776	-1.5198	3.06E-30
CNVR_541	chrAC_000168.1	104709631	104711850	2220	-1.2974	2.24E-30
CNVR_542	chrAC_000187.1	152515	155622	3108	1.2283	4.47E-25
CNVR_543	chrAC_000187.1	9572419	9574194	1776	1.2185	7.90E-15
CNVR_544	chrAC_000187.1	10241971	10243746	1776	1.4136	3.09E-18
CNVR_545	chrAC_000187.1	11925619	11929614	3996	1.9034	3.94E-57
CNVR_546	chrAC_000187.1	13326439	13328214	1776	1.3040	2.54E-16
CNVR_547	chrAC_000187.1	18821383	18823602	2220	1.2698	2.87E-19
CNVR_548	chrAC_000187.1	24626239	24628014	1776	1.1319	2.50E-13
CNVR_549	chrAC_000187.1	33901843	33903618	1776	1.0528	5.60E-12
CNVR_550	chrAC_000187.1	36957451	36959226	1776	1.5801	4.21E-21
CNVR_551	chrAC_000187.1	41358379	41361930	3552	1.3541	8.70E-33
CNVR_552	chrAC_000187.1	41363263	41365038	1776	1.2601	1.48E-15
CNVR_553	chrAC_000187.1	44342059	44345610	3552	1.4173	5.57E-35
CNVR_554	chrAC_000187.1	44362927	44365146	2220	1.2692	2.96E-19
CNVR_555	chrAC_000187.1	44377135	44378910	1776	1.2239	6.36E-15
CNVR_556	chrAC_000187.1	45508003	45509778	1776	1.2380	3.61E-15
CNVR_557	chrAC_000187.1	50770735	50772510	1776	1.2786	7.05E-16
CNVR_558	chrAC_000187.1	53023147	53025810	2664	3.0603	8.08E-59
CNVR_559	chrAC_000187.1	55294651	55296426	1776	1.3924	7.24E-18
CNVR_560	chrAC_000187.1	70475899	70477674	1776	1.2952	3.61E-16
CNVR_561	chrAC_000187.1	75337255	75339030	1776	1.9090	1.98E-26
CNVR_562	chrAC_000187.1	83229799	83233794	3996	1.6530	3.86E-48
CNVR_563	chrAC_000187.1	89658919	89662026	3108	1.6028	2.31E-36
CNVR_564	chrAC_000187.1	90360883	90362658	1776	1.5555	1.10E-20
CNVR_565	chrAC_000187.1	90711643	90715638	3996	1.6713	7.99E-49
CNVR_566	chrAC_000187.1	94068283	94070502	2220	1.3950	5.38E-22
CNVR_567	chrAC_000187.1	124878775	124880550	1776	1.1950	2.02E-14
CNVR_568	chrAC_000187.1	126673423	126675198	1776	1.2205	7.28E-15

CNVR_569	chrAC_000187.1	136669195	136671414	2220	1.2754	2.17E-19
CNVR_570	chrAC_000187.1	148220299	148222074	1776	1.6835	7.79E-23
CNVR_571	chrAC_000187.1	148307767	148309542	1776	1.0685	3.03E-12
CNVR_572	chrAC_000187.1	591631	609390	17760	-3.7864	0.00E+00
CNVR_573	chrAC_000187.1	4193803	4196022	2220	-2.2389	2.95E-31
CNVR_574	chrAC_000187.1	8408251	8410026	1776	-1.1956	1.73E-12
CNVR_575	chrAC_000187.1	8439331	8441106	1776	-1.8195	1.03E-20
CNVR_576	chrAC_000187.1	9394375	9396150	1776	-1.3136	4.08E-14
CNVR_577	chrAC_000187.1	10184695	10187358	2664	-1.5308	8.65E-25
CNVR_578	chrAC_000187.1	10447099	10449318	2220	-1.1619	1.18E-14
CNVR_579	chrAC_000187.1	10451539	10453314	1776	-1.1271	1.53E-11
CNVR_580	chrAC_000187.1	10751683	10753458	1776	-1.3464	1.44E-14
CNVR_581	chrAC_000187.1	12260839	12262614	1776	-1.8162	1.13E-20
CNVR_582	chrAC_000187.1	13219879	13221654	1776	-1.1553	6.24E-12
CNVR_583	chrAC_000187.1	14108767	14110986	2220	-1.3336	1.32E-17
CNVR_584	chrAC_000187.1	16322551	16324326	1776	-1.5110	8.62E-17
CNVR_585	chrAC_000187.1	17684299	17686962	2664	-3.0371	9.48E-47
CNVR_586	chrAC_000187.1	17717155	17718930	1776	-1.7957	2.00E-20
CNVR_587	chrAC_000187.1	19530895	19532670	1776	-1.1201	1.91E-11
CNVR_588	chrAC_000187.1	19774207	19775982	1776	-1.2569	2.46E-13
CNVR_589	chrAC_000187.1	19778793	19780569	1776	1.0547	3.53E-14
CNVR_590	chrAC_000187.1	21413011	21414786	1776	-1.3909	3.57E-15
CNVR_591	chrAC_000187.1	22664203	22665978	1776	1.2229	4.18E-41
CNVR_592	chrAC_000187.1	24622243	24624018	1776	-1.2745	1.41E-13
CNVR_593	chrAC_000187.1	26611807	26613582	1776	-1.4277	1.13E-15
CNVR_594	chrAC_000187.1	27167251	27169026	1776	-1.7978	1.89E-20
CNVR_595	chrAC_000187.1	27461179	27463398	2220	-1.7524	1.81E-24
CNVR_596	chrAC_000187.1	27861223	27863886	2664	-1.3251	1.22E-20
CNVR_597	chrAC_000187.1	29451187	29454738	3552	-1.3633	5.06E-28
CNVR_598	chrAC_000187.1	29927155	29929374	2220	-1.2071	1.96E-15
CNVR_599	chrAC_000187.1	30307219	30309438	2220	-4.4047	2.49E-46
CNVR_600	chrAC_000187.1	30315655	30319206	3552	-5.2568	2.60E-76
CNVR_601	chrAC_000187.1	30609583	30611358	1776	-1.6388	1.84E-18
CNVR_602	chrAC_000187.1	31110859	31113522	2664	-2.1328	1.52E-35
CNVR_603	chrAC_000187.1	31196107	31197882	1776	-1.2987	6.53E-14
CNVR_604	chrAC_000187.1	32389135	32390910	1776	-1.3544	1.12E-14
CNVR_605	chrAC_000187.1	32397571	32400234	2664	-1.5329	7.85E-25
CNVR_606	chrAC_000187.1	32436199	32437974	1776	-1.4613	3.98E-16
CNVR_607	chrAC_000187.1	32886859	32888634	1776	-1.8239	9.12E-21
CNVR_608	chrAC_000187.1	33791287	33793506	2220	-3.3050	3.56E-41
CNVR_609	chrAC_000187.1	34249051	34250826	1776	-1.0828	6.23E-11
CNVR_610	chrAC_000187.1	35516227	35564178	47952	-2.0720	0.00E+00
CNVR_611	chrAC_000187.1	35646763	35650314	3552	-1.4472	2.77E-30
CNVR_612	chrAC_000187.1	35651203	35656086	4884	-1.6144	5.00E-47

CNVR_613	chrAC_000187.1	35659639	35665410	5772	-1.5930	2.34E-54
CNVR_614	chrAC_000187.1	35665855	35672514	6660	-1.4152	1.17E-53
CNVR_615	chrAC_000187.1	35673847	35675622	1776	-1.2164	8.95E-13
CNVR_616	chrAC_000187.1	35687167	35689830	2664	-1.3757	1.13E-21
CNVR_617	chrAC_000187.1	35729347	35732898	3552	-1.4562	1.60E-30
CNVR_618	chrAC_000187.1	35733343	35740002	6660	-1.3057	4.13E-48
CNVR_619	chrAC_000187.1	35744443	35749326	4884	-1.5128	2.15E-43
CNVR_620	chrAC_000187.1	35762203	35766198	3996	-1.3144	8.06E-30
CNVR_621	chrAC_000187.1	35778631	35780406	1776	-1.6159	3.63E-18
CNVR_622	chrAC_000187.1	35781739	35783958	2220	-1.7354	3.33E-24
CNVR_623	chrAC_000187.1	35788843	35792394	3552	-1.7733	1.16E-38
CNVR_624	chrAC_000187.1	35792839	35794614	1776	-1.2250	6.80E-13
CNVR_625	chrAC_000187.1	35800831	35803050	2220	-1.0985	1.44E-13
CNVR_626	chrAC_000187.1	35809711	35811930	2220	-1.2442	4.51E-16
CNVR_627	chrAC_000187.1	35814151	35818590	4440	-1.9068	7.50E-52
CNVR_628	chrAC_000187.1	35821255	35826582	5328	-1.6117	4.90E-51
CNVR_629	chrAC_000187.1	35834131	35835906	1776	-2.3082	5.17E-26
CNVR_630	chrAC_000187.1	35836351	35838570	2220	-1.9164	6.41E-27
CNVR_631	chrAC_000187.1	35839015	35840790	1776	-2.2679	1.27E-25
CNVR_632	chrAC_000187.1	35841235	35846562	5328	-1.7225	3.13E-55
CNVR_633	chrAC_000187.1	35847895	35851002	3108	-1.4310	2.53E-26
CNVR_634	chrAC_000187.1	35856331	35858550	2220	-1.2577	2.64E-16
CNVR_635	chrAC_000187.1	35863435	35865654	2220	-1.9926	5.29E-28
CNVR_636	chrAC_000187.1	35872759	35875866	3108	-1.8676	5.10E-36
CNVR_637	chrAC_000187.1	35878087	35880306	2220	-1.8875	1.69E-26
CNVR_638	chrAC_000187.1	35881639	35895846	14208	-1.6431	3.60E-136
CNVR_639	chrAC_000187.1	35896291	35899842	3552	-1.5109	5.61E-32
CNVR_640	chrAC_000187.1	35902063	35910942	8880	-1.6625	4.55E-87
CNVR_641	chrAC_000187.1	35911387	35914938	3552	-1.7353	1.00E-37
CNVR_642	chrAC_000187.1	35915827	35923818	7992	-1.5320	5.39E-71
CNVR_643	chrAC_000187.1	35924263	35926926	2664	-1.8784	2.62E-31
CNVR_644	chrAC_000187.1	35927371	35937582	10212	-1.9248	5.12E-118
CNVR_645	chrAC_000187.1	35938471	35942910	4440	-2.0580	4.39E-56
CNVR_646	chrAC_000187.1	35944243	35946462	2220	-1.4507	1.36E-19
CNVR_647	chrAC_000187.1	35947351	35977098	29748	-1.6177	1.95E-277
CNVR_648	chrAC_000187.1	35977987	35979762	1776	-1.3839	4.44E-15
CNVR_649	chrAC_000187.1	35990419	35993970	3552	-1.3806	1.72E-28
CNVR_650	chrAC_000187.1	36011287	36017058	5772	-1.3735	6.42E-45
CNVR_651	chrAC_000187.1	36017947	36019722	1776	-1.6750	6.34E-19
CNVR_652	chrAC_000187.1	36021055	36023274	2220	-1.8556	4.98E-26
CNVR_653	chrAC_000187.1	36027715	36029490	1776	-1.2161	9.01E-13
CNVR_654	chrAC_000187.1	36034819	36037038	2220	-1.2940	6.28E-17
CNVR_655	chrAC_000187.1	36053023	36054798	1776	-1.2282	6.14E-13
CNVR_656	chrAC_000187.1	36056575	36058794	2220	-1.7813	6.53E-25

CNVR_657	chrAC_000187.1	36065899	36068118	2220	-1.1707	8.30E-15
CNVR_658	chrAC_000187.1	36069007	36070782	1776	-1.2937	7.66E-14
CNVR_659	chrAC_000187.1	36080107	36081882	1776	-1.0839	6.02E-11
CNVR_660	chrAC_000187.1	36095647	36097422	1776	-1.4689	3.14E-16
CNVR_661	chrAC_000187.1	36127171	36135606	8436	-1.4176	1.24E-67
CNVR_662	chrAC_000187.1	36136495	36146262	9768	-1.5228	1.08E-85
CNVR_663	chrAC_000187.1	36146707	36154698	7992	-1.6259	1.84E-76
CNVR_664	chrAC_000187.1	36155587	36166686	11100	-1.6847	4.30E-110
CNVR_665	chrAC_000187.1	36167131	36175566	8436	-1.9046	9.91E-97
CNVR_666	chrAC_000187.1	36176455	36190662	14208	-1.6698	7.19E-139
CNVR_667	chrAC_000187.1	36191551	36199098	7548	-1.6688	1.26E-74
CNVR_668	chrAC_000187.1	36199987	36204870	4884	-1.8774	6.48E-56
CNVR_669	chrAC_000187.1	36211531	36216414	4884	-1.5173	1.48E-43
CNVR_670	chrAC_000187.1	36217303	36219522	2220	-1.7441	2.44E-24
CNVR_671	chrAC_000187.1	36219967	36234618	14652	-1.8115	1.63E-157
CNVR_672	chrAC_000187.1	36235063	36242610	7548	-1.7929	3.89E-81
CNVR_673	chrAC_000187.1	36243055	36248382	5328	-1.7788	2.64E-57
CNVR_674	chrAC_000187.1	36249715	36256374	6660	-1.5755	1.36E-61
CNVR_675	chrAC_000187.1	36260815	36262590	1776	-1.1863	2.33E-12
CNVR_676	chrAC_000187.1	36294115	36299442	5328	-1.5768	1.10E-49
CNVR_677	chrAC_000187.1	36302551	36305658	3108	-1.3015	2.99E-23
CNVR_678	chrAC_000187.1	36306103	36308766	2664	-1.5678	1.62E-25
CNVR_679	chrAC_000187.1	36314095	36315870	1776	-1.1236	1.71E-11
CNVR_680	chrAC_000187.1	36336739	36340734	3996	-1.6281	3.45E-39
CNVR_681	chrAC_000187.1	36347395	36349614	2220	-1.3414	9.70E-18
CNVR_682	chrAC_000187.1	36358939	36361602	2664	-1.3386	6.45E-21
CNVR_683	chrAC_000187.1	36370483	36373146	2664	-1.7102	3.01E-28
CNVR_684	chrAC_000187.1	36378919	36382914	3996	-1.3860	5.25E-32
CNVR_685	chrAC_000187.1	36390019	36392682	2664	-1.2004	4.47E-18
CNVR_686	chrAC_000187.1	36413995	36416658	2664	-1.2811	9.75E-20
CNVR_687	chrAC_000187.1	36439303	36443742	4440	-1.3233	3.00E-33
CNVR_688	chrAC_000187.1	36451291	36456174	4884	-1.3847	1.08E-38
CNVR_689	chrAC_000187.1	36464167	36465942	1776	-1.3204	3.29E-14
CNVR_690	chrAC_000187.1	36526327	36528102	1776	-1.6279	2.53E-18
CNVR_691	chrAC_000187.1	36580051	36582714	2664	-1.3597	2.39E-21
CNVR_692	chrAC_000187.1	36597367	36599586	2220	-2.0053	3.51E-28
CNVR_693	chrAC_000187.1	36600475	36602250	1776	-1.2820	1.11E-13
CNVR_694	chrAC_000187.1	36604915	36606690	1776	-1.6744	6.45E-19
CNVR_695	chrAC_000187.1	36609355	36612462	3108	-1.9735	3.72E-38
CNVR_696	chrAC_000187.1	36612907	36615126	2220	-1.7406	2.76E-24
CNVR_697	chrAC_000187.1	36618679	36620898	2220	-2.0175	2.38E-28
CNVR_698	chrAC_000187.1	36626671	36628446	1776	-2.2169	4.08E-25
CNVR_699	chrAC_000187.1	36636439	36638658	2220	-1.2078	1.91E-15
CNVR_700	chrAC_000187.1	36723907	36726126	2220	-1.1657	1.01E-14

CNVR_701	chrAC_000187.1	36745663	36750546	4884	-1.5637	3.17E-45
CNVR_702	chrAC_000187.1	36791839	36794058	2220	-1.4360	2.40E-19
CNVR_703	chrAC_000187.1	36800275	36802494	2220	-1.2007	2.53E-15
CNVR_704	chrAC_000187.1	36815815	36818034	2220	-1.1759	6.75E-15
CNVR_705	chrAC_000187.1	36818923	36820698	1776	-1.1965	1.69E-12
CNVR_706	chrAC_000187.1	36830911	36832686	1776	-1.3169	3.67E-14
CNVR_707	chrAC_000187.1	36842011	36844230	2220	-1.0774	3.31E-13
CNVR_708	chrAC_000187.1	36849559	36851334	1776	-1.2914	8.25E-14
CNVR_709	chrAC_000187.1	36872647	36875754	3108	-1.3109	1.78E-23
CNVR_710	chrAC_000187.1	36917491	36919266	1776	-1.5318	4.57E-17
CNVR_711	chrAC_000187.1	36920155	36924594	4440	-1.3374	9.93E-34
CNVR_712	chrAC_000187.1	36939247	36941910	2664	-1.2602	2.62E-19
CNVR_713	chrAC_000187.1	36994747	36997410	2664	-1.5170	1.62E-24
CNVR_714	chrAC_000187.1	37031155	37032930	1776	-1.1134	2.37E-11
CNVR_715	chrAC_000187.1	37035151	37036926	1776	-1.3732	6.22E-15
CNVR_716	chrAC_000187.1	37052911	37054686	1776	-1.4745	2.64E-16
CNVR_717	chrAC_000187.1	37061347	37063122	1776	-1.2624	2.07E-13
CNVR_718	chrAC_000187.1	37092871	37095090	2220	-1.6397	1.07E-22
CNVR_719	chrAC_000187.1	37139491	37141266	1776	-1.5501	2.62E-17
CNVR_720	chrAC_000187.1	37146151	37151478	5328	-1.4087	5.07E-43
CNVR_721	chrAC_000187.1	37159915	37162134	2220	-1.3326	1.37E-17
CNVR_722	chrAC_000187.1	37164355	37167462	3108	-1.3764	4.92E-25
CNVR_723	chrAC_000187.1	37242055	37244274	2220	-2.1461	4.46E-30
CNVR_724	chrAC_000187.1	37246051	37248714	2664	-1.2156	2.18E-18
CNVR_725	chrAC_000187.1	37510675	37512450	1776	-1.6539	1.18E-18
CNVR_726	chrAC_000187.1	37617679	37619454	1776	-1.5763	1.19E-17
CNVR_727	chrAC_000187.1	37909387	37911162	1776	-1.2119	1.03E-12
CNVR_728	chrAC_000187.1	37954231	37957338	3108	-1.2345	1.21E-21
CNVR_729	chrAC_000187.1	37961335	37963554	2220	-1.4098	6.65E-19
CNVR_730	chrAC_000187.1	37967551	37971102	3552	-1.2155	5.49E-24
CNVR_731	chrAC_000187.1	37979983	37982646	2664	-1.6873	8.14E-28
CNVR_732	chrAC_000187.1	38116735	38118510	1776	-2.2552	1.69E-25
CNVR_733	chrAC_000187.1	38582935	38584710	1776	-1.1511	7.15E-12
CNVR_734	chrAC_000187.1	38694823	38700150	5328	-3.6124	1.15E-100
CNVR_735	chrAC_000187.1	39204979	39206754	1776	-1.4281	1.12E-15
CNVR_736	chrAC_000187.1	39365263	39367482	2220	-1.5521	2.82E-21
CNVR_737	chrAC_000187.1	40550743	40552962	2220	-1.3298	1.53E-17
CNVR_738	chrAC_000187.1	40553851	40560510	6660	-1.5481	2.95E-60
CNVR_739	chrAC_000187.1	40562287	40567614	5328	-1.4314	6.18E-44
CNVR_740	chrAC_000187.1	40569391	40574274	4884	-1.7000	5.20E-50
CNVR_741	chrAC_000187.1	40576939	40581822	4884	-1.6026	1.30E-46
CNVR_742	chrAC_000187.1	41465383	41467158	1776	-2.3994	7.23E-27
CNVR_743	chrAC_000187.1	41578159	41581710	3552	-1.3711	3.13E-28
CNVR_744	chrAC_000187.1	41986195	41988414	2220	-1.4338	2.62E-19

CNVR_745	chrAC_000187.1	42869755	42871530	1776	-1.2900	8.62E-14
CNVR_746	chrAC_000187.1	44566279	44568498	2220	-1.3679	3.42E-18
CNVR_747	chrAC_000187.1	45606571	45611898	5328	-3.3218	1.55E-96
CNVR_748	chrAC_000187.1	46274347	46276122	1776	-1.3644	8.19E-15
CNVR_749	chrAC_000187.1	47634763	47636538	1776	-1.5925	7.28E-18
CNVR_750	chrAC_000187.1	48810919	48821574	10656	-3.0896	2.81E-183
CNVR_751	chrAC_000187.1	49288663	49291326	2664	-1.9759	5.48E-33
CNVR_752	chrAC_000187.1	49366807	49368582	1776	-1.2624	2.07E-13
CNVR_753	chrAC_000187.1	52262131	52263906	1776	-1.6804	5.41E-19
CNVR_754	chrAC_000187.1	52418419	52422414	3996	-2.9585	4.34E-68
CNVR_755	chrAC_000187.1	52738099	52739874	1776	-1.1223	1.78E-11
CNVR_756	chrAC_000187.1	54786271	54788046	1776	-2.0354	3.33E-23
CNVR_757	chrAC_000187.1	55027363	55030914	3552	-1.6467	1.68E-35
CNVR_758	chrAC_000187.1	57377899	57381006	3108	-1.1968	9.72E-21
CNVR_759	chrAC_000187.1	57501775	57503550	1776	-2.5925	1.53E-28
CNVR_760	chrAC_000187.1	58836439	58838214	1776	-2.9152	6.01E-31
CNVR_761	chrAC_000187.1	60228823	60231042	2220	-1.3942	1.22E-18
CNVR_762	chrAC_000187.1	61684255	61686030	1776	-1.2584	2.35E-13
CNVR_763	chrAC_000187.1	63625867	63628974	3108	-1.5064	4.39E-28
CNVR_764	chrAC_000187.1	63629419	63639630	10212	-1.7370	3.50E-105
CNVR_765	chrAC_000187.1	63641407	63646734	5328	-1.7524	2.44E-56
CNVR_766	chrAC_000187.1	63647623	63650730	3108	-1.8690	4.77E-36
CNVR_767	chrAC_000187.1	63658279	63672042	13764	-1.5029	6.22E-118
CNVR_768	chrAC_000187.1	63676039	63677814	1776	-2.3629	1.57E-26
CNVR_769	chrAC_000187.1	63681811	63689358	7548	-1.9655	1.05E-89
CNVR_770	chrAC_000187.1	63689803	63691578	1776	-2.6453	5.75E-29
CNVR_771	chrAC_000187.1	63692911	63695574	2664	-2.3264	1.93E-38
CNVR_772	chrAC_000187.1	63698683	63701790	3108	-1.8317	2.82E-35
CNVR_773	chrAC_000187.1	63708007	63710226	2220	-1.6361	1.22E-22
CNVR_774	chrAC_000187.1	63711115	63717774	6660	-2.1989	1.02E-88
CNVR_775	chrAC_000187.1	63721327	63723990	2664	-1.8860	1.93E-31
CNVR_776	chrAC_000187.1	63724879	63726654	1776	-1.4983	1.27E-16
CNVR_777	chrAC_000187.1	63729763	63731982	2220	-2.7535	7.94E-37
CNVR_778	chrAC_000187.1	63735535	63739974	4440	-1.8239	2.06E-49
CNVR_779	chrAC_000187.1	63740419	63746190	5772	-2.0535	3.36E-72
CNVR_780	chrAC_000187.1	63751075	63752850	1776	-2.0463	2.53E-23
CNVR_781	chrAC_000187.1	63760843	63762618	1776	-2.2004	5.99E-25
CNVR_782	chrAC_000187.1	63763951	63766614	2664	-2.1085	3.67E-35
CNVR_783	chrAC_000187.1	63777271	63783930	6660	-1.9187	2.25E-77
CNVR_784	chrAC_000187.1	63792811	63796362	3552	-2.3226	1.34E-50
CNVR_785	chrAC_000187.1	63799915	63803022	3108	-1.5612	2.41E-29
CNVR_786	chrAC_000187.1	63807019	63811014	3996	-1.7814	1.70E-43
CNVR_787	chrAC_000187.1	63811903	63824334	12432	-1.7460	2.15E-128
CNVR_788	chrAC_000187.1	63824779	63835434	10656	-1.7863	2.83E-113

CNVR_789	chrAC_000187.1	63835879	63844314	8436	-2.0665	2.62E-105
CNVR_790	chrAC_000187.1	63845647	63848310	2664	-1.7759	1.82E-29
CNVR_791	chrAC_000187.1	63849643	63852750	3108	-1.4917	9.64E-28
CNVR_792	chrAC_000187.1	63853639	63861630	7992	-1.8493	7.86E-89
CNVR_793	chrAC_000187.1	63862519	63870954	8436	-1.4259	3.64E-68
CNVR_794	chrAC_000187.1	63871399	63873618	2220	-1.3663	3.65E-18
CNVR_795	chrAC_000187.1	63877171	63887382	10212	-1.5022	6.05E-88
CNVR_796	chrAC_000187.1	63890047	63893598	3552	-1.8345	3.90E-40
CNVR_797	chrAC_000187.1	63894043	63918906	24864	-1.8392	1.87E-270
CNVR_798	chrAC_000187.1	63919795	63923346	3552	-1.3034	2.16E-26
CNVR_799	chrAC_000187.1	63932671	63936666	3996	-1.1375	2.22E-24
CNVR_800	chrAC_000187.1	63937111	63941994	4884	-1.8715	1.00E-55
CNVR_801	chrAC_000187.1	63945991	63948210	2220	-1.1581	1.37E-14
CNVR_802	chrAC_000187.1	63949099	63951762	2664	-1.5448	4.57E-25
CNVR_803	chrAC_000187.1	63953983	63956202	2220	-1.4559	1.12E-19
CNVR_804	chrAC_000187.1	63961087	63963750	2664	-1.3272	1.10E-20
CNVR_805	chrAC_000187.1	63968635	63970854	2220	-1.4767	5.01E-20
CNVR_806	chrAC_000187.1	63971299	63977958	6660	-1.6175	1.27E-63
CNVR_807	chrAC_000187.1	63993943	63998382	4440	-1.2739	1.44E-31
CNVR_808	chrAC_000187.1	63999271	64001490	2220	-1.5001	2.03E-20
CNVR_809	chrAC_000187.1	64005487	64007706	2220	-1.5440	3.83E-21
CNVR_810	chrAC_000187.1	64012147	64017918	5772	-1.6496	1.03E-56
CNVR_811	chrAC_000187.1	64019695	64026354	6660	-1.4962	1.06E-57
CNVR_812	chrAC_000187.1	64026799	64029906	3108	-1.1535	1.06E-19
CNVR_813	chrAC_000187.1	64041895	64045002	3108	-1.9659	5.27E-38
CNVR_814	chrAC_000187.1	64047223	64048998	1776	-1.7962	1.97E-20
CNVR_815	chrAC_000187.1	64055215	64063650	8436	-1.3074	1.42E-60
CNVR_816	chrAC_000187.1	64066315	64068534	2220	-1.4093	6.78E-19
CNVR_817	chrAC_000187.1	64074307	64076082	1776	-1.5974	6.30E-18
CNVR_818	chrAC_000187.1	64080079	64093842	13764	-1.6803	1.29E-135
CNVR_819	chrAC_000187.1	64094287	64101834	7548	-1.7799	1.80E-80
CNVR_820	chrAC_000187.1	64104943	64117818	12876	-2.1528	2.83E-166
CNVR_821	chrAC_000187.1	64118707	64130250	11544	-1.9868	1.05E-137
CNVR_822	chrAC_000187.1	64131583	64134690	3108	-1.8497	1.19E-35
CNVR_823	chrAC_000187.1	64139131	64141350	2220	-1.7768	7.65E-25
CNVR_824	chrAC_000187.1	64143571	64145790	2220	-1.7778	7.39E-25
CNVR_825	chrAC_000187.1	64147123	64149342	2220	-1.5314	6.19E-21
CNVR_826	chrAC_000187.1	64155115	64156890	1776	-1.4540	4.99E-16
CNVR_827	chrAC_000187.1	64158223	64160886	2664	-1.7828	1.36E-29
CNVR_828	chrAC_000187.1	64163995	64172874	8880	-1.7845	1.30E-94
CNVR_829	chrAC_000187.1	64175983	64178646	2664	-1.5201	1.41E-24
CNVR_830	chrAC_000187.1	64183087	64185750	2664	-1.0954	6.45E-16
CNVR_831	chrAC_000187.1	64210171	64212390	2220	-1.8124	2.21E-25
CNVR_832	chrAC_000187.1	64216387	64221270	4884	-1.5213	1.06E-43

CNVR_833	chrAC_000187.1	64229263	64231926	2664	-1.9857	3.74E-33
CNVR_834	chrAC_000187.1	64238587	64243470	4884	-1.2662	2.88E-34
CNVR_835	chrAC_000187.1	64243915	64245690	1776	-1.2307	5.66E-13
CNVR_836	chrAC_000187.1	64246135	64252794	6660	-1.7050	9.13E-68
CNVR_837	chrAC_000187.1	64253683	64256790	3108	-1.3262	7.69E-24
CNVR_838	chrAC_000187.1	64258123	64260342	2220	-1.7094	8.45E-24
CNVR_839	chrAC_000187.1	64261231	64269666	8436	-1.4169	1.37E-67
CNVR_840	chrAC_000187.1	64278103	64279878	1776	-1.2916	8.19E-14
CNVR_841	chrAC_000187.1	64285207	64288314	3108	-1.5083	3.98E-28
CNVR_842	chrAC_000187.1	64288759	64300302	11544	-1.7980	1.80E-123
CNVR_843	chrAC_000187.1	64418407	64420626	2220	-1.3711	3.02E-18
CNVR_844	chrAC_000187.1	64422847	64429506	6660	-1.7354	3.51E-69
CNVR_845	chrAC_000187.1	64431283	64434390	3108	-1.7981	1.44E-34
CNVR_846	chrAC_000187.1	64435279	64445046	9768	-1.6496	1.11E-94
CNVR_847	chrAC_000187.1	64445935	64447710	1776	-2.3079	5.19E-26
CNVR_848	chrAC_000187.1	64449487	64461918	12432	-1.6986	2.73E-124
CNVR_849	chrAC_000187.1	64462807	64464582	1776	-1.5313	4.64E-17
CNVR_850	chrAC_000187.1	64465471	64471242	5772	-1.8547	6.96E-65
CNVR_851	chrAC_000187.1	64471687	64473906	2220	-1.5177	1.04E-20
CNVR_852	chrAC_000187.1	64475683	64483230	7548	-1.4966	3.54E-65
CNVR_853	chrAC_000187.1	64483675	64487226	3552	-1.7258	1.72E-37
CNVR_854	chrAC_000187.1	64487671	64490778	3108	-1.3229	9.22E-24
CNVR_855	chrAC_000187.1	64494331	64497438	3108	-1.3304	6.11E-24
CNVR_856	chrAC_000187.1	64497883	64503654	5772	-1.6998	9.15E-59
CNVR_857	chrAC_000187.1	64504099	64512090	7992	-1.5295	7.58E-71
CNVR_858	chrAC_000187.1	64512979	64520970	7992	-1.7266	3.76E-82
CNVR_859	chrAC_000187.1	64529851	64531626	1776	-1.5229	5.99E-17
CNVR_860	chrAC_000187.1	64532071	64535622	3552	-1.4885	2.21E-31
CNVR_861	chrAC_000187.1	64536955	64546278	9324	-1.9347	1.61E-108
CNVR_862	chrAC_000187.1	64548943	64551162	2220	-1.4449	1.71E-19
CNVR_863	chrAC_000187.1	64554271	64556046	1776	-1.7159	1.93E-19
CNVR_864	chrAC_000187.1	64556935	64558710	1776	-1.4672	3.32E-16
CNVR_865	chrAC_000187.1	64559599	64561818	2220	-1.2592	2.48E-16
CNVR_866	chrAC_000187.1	64564927	64566702	1776	-2.5004	9.14E-28
CNVR_867	chrAC_000187.1	64567147	64571142	3996	-1.7488	1.34E-42
CNVR_868	chrAC_000187.1	64572475	64574250	1776	-2.3667	1.45E-26
CNVR_869	chrAC_000187.1	64576027	64578246	2220	-2.5799	3.98E-35
CNVR_870	chrAC_000187.1	64578691	64581354	2664	-1.8503	8.25E-31
CNVR_871	chrAC_000187.1	64581799	64584906	3108	-1.4613	4.92E-27
CNVR_872	chrAC_000187.1	65340151	65342370	2220	-1.6046	3.94E-22
CNVR_873	chrAC_000187.1	65342815	65344590	1776	-1.4386	8.05E-16
CNVR_874	chrAC_000187.1	65345479	65347698	2220	-1.6199	2.22E-22
CNVR_875	chrAC_000187.1	65348143	65354358	6216	-1.6769	3.83E-62
CNVR_876	chrAC_000187.1	65354803	65357022	2220	-1.4865	3.43E-20

CNVR_877	chrAC_000187.1	65456035	65459142	3108	-1.3651	9.11E-25
CNVR_878	chrAC_000187.1	65460031	65462694	2664	-1.7101	3.03E-28
CNVR_879	chrAC_000187.1	65463139	65466246	3108	-2.2208	9.31E-43
CNVR_880	chrAC_000187.1	65468467	65472018	3552	-1.7374	8.92E-38
CNVR_881	chrAC_000187.1	65485783	65487558	1776	-1.3202	3.31E-14
CNVR_882	chrAC_000187.1	65489779	65494218	4440	-1.6479	5.53E-44
CNVR_883	chrAC_000187.1	66106051	66108714	2664	-3.2578	1.11E-48
CNVR_884	chrAC_000187.1	66980287	66982062	1776	-1.2640	1.97E-13
CNVR_885	chrAC_000187.1	67246243	67248018	1776	-1.5925	7.28E-18
CNVR_886	chrAC_000187.1	67771051	67772826	1776	-1.6061	4.85E-18
CNVR_887	chrAC_000187.1	68013475	68017470	3996	-1.5645	2.42E-37
CNVR_888	chrAC_000187.1	68939659	68941434	1776	-1.3774	5.45E-15
CNVR_889	chrAC_000187.1	69244243	69246018	1776	-1.5751	1.23E-17
CNVR_890	chrAC_000187.1	71011807	71013582	1776	-1.7308	1.26E-19
CNVR_891	chrAC_000187.1	71448703	71450478	1776	-1.1816	2.71E-12
CNVR_892	chrAC_000187.1	72112483	72114702	2220	-1.3284	1.62E-17
CNVR_893	chrAC_000187.1	72538723	72540498	1776	-1.5438	3.17E-17
CNVR_894	chrAC_000187.1	72733195	72734970	1776	-1.2784	1.24E-13
CNVR_895	chrAC_000187.1	73347247	73349022	1776	-1.4613	3.98E-16
CNVR_896	chrAC_000187.1	73685131	73686906	1776	-1.2622	2.08E-13
CNVR_897	chrAC_000187.1	74126911	74128686	1776	-1.3169	3.67E-14
CNVR_898	chrAC_000187.1	75448699	75450918	2220	-1.4613	9.04E-20
CNVR_899	chrAC_000187.1	76561807	76563582	1776	-1.8736	2.33E-21
CNVR_900	chrAC_000187.1	77312611	77314386	1776	-1.4060	2.22E-15
CNVR_901	chrAC_000187.1	77982607	77984382	1776	-1.6924	3.81E-19
CNVR_902	chrAC_000187.1	79151215	79152990	1776	-1.8404	5.78E-21
CNVR_903	chrAC_000187.1	80224363	80226138	1776	-1.2389	4.37E-13
CNVR_904	chrAC_000187.1	80789575	80791350	1776	-1.8239	9.12E-21
CNVR_905	chrAC_000187.1	81106591	81108366	1776	-1.9415	3.77E-22
CNVR_906	chrAC_000187.1	81734851	81737514	2664	-1.5677	1.63E-25
CNVR_907	chrAC_000187.1	85932871	85934646	1776	-1.2906	8.46E-14
CNVR_908	chrAC_000187.1	92239003	92240778	1776	-1.7555	6.22E-20
CNVR_909	chrAC_000187.1	92282071	92283846	1776	-1.7319	1.22E-19
CNVR_910	chrAC_000187.1	100745599	100747374	1776	-1.9063	9.63E-22
CNVR_911	chrAC_000187.1	104247427	104249202	1776	-1.1465	8.26E-12
CNVR_912	chrAC_000187.1	105303259	105305478	2220	-1.0537	8.39E-13
CNVR_913	chrAC_000187.1	105679771	105681546	1776	-1.3320	2.28E-14
CNVR_914	chrAC_000187.1	108812635	108814410	1776	-1.8556	3.81E-21
CNVR_915	chrAC_000187.1	110531803	110534022	2220	-1.3764	2.45E-18
CNVR_916	chrAC_000187.1	111216895	111218670	1776	-1.5824	9.88E-18
CNVR_917	chrAC_000187.1	112398823	112401042	2220	-1.3544	5.82E-18
CNVR_918	chrAC_000187.1	112845043	112846818	1776	-1.5868	8.64E-18
CNVR_919	chrAC_000187.1	114819511	114821730	2220	-1.4567	1.08E-19
CNVR_920	chrAC_000187.1	115897987	115899762	1776	-1.6252	2.75E-18

CNVR_921	chrAC_000187.1	116275831	116278050	2220	-1.3245	1.89E-17
CNVR_922	chrAC_000187.1	117067039	117068814	1776	-1.3154	3.86E-14
CNVR_923	chrAC_000187.1	118721383	118724046	2664	-1.8066	5.04E-30
CNVR_924	chrAC_000187.1	118726711	118728930	2220	-1.5098	1.41E-20
CNVR_925	chrAC_000187.1	118778659	118780878	2220	-1.4478	1.52E-19
CNVR_926	chrAC_000187.1	118781767	118783542	1776	-1.4414	7.38E-16
CNVR_927	chrAC_000187.1	118784431	118787982	3552	-1.4236	1.19E-29
CNVR_928	chrAC_000187.1	118797307	118799082	1776	-1.5462	2.95E-17
CNVR_929	chrAC_000187.1	118809739	118811958	2220	-1.6174	2.44E-22
CNVR_930	chrAC_000187.1	118864795	118867458	2664	-1.1813	1.11E-17
CNVR_931	chrAC_000187.1	118895431	118897206	1776	-1.5562	2.17E-17
CNVR_932	chrAC_000187.1	118961587	118963806	2220	-2.9292	2.23E-38
CNVR_933	chrAC_000187.1	118970023	118974462	4440	-1.6384	1.11E-43
CNVR_934	chrAC_000187.1	118974907	118977570	2664	-2.4921	1.08E-40
CNVR_935	chrAC_000187.1	118983787	118985562	1776	-2.5772	2.05E-28
CNVR_936	chrAC_000187.1	119005987	119007762	1776	-4.5925	7.35E-38
CNVR_937	chrAC_000187.1	119068147	119069922	1776	-1.3544	1.12E-14
CNVR_938	chrAC_000187.1	119070811	119074806	3996	-1.4332	1.95E-33
CNVR_939	chrAC_000187.1	119115655	119119650	3996	-2.8379	3.03E-66
CNVR_940	chrAC_000187.1	119136079	119138742	2664	-2.8700	4.23E-45
CNVR_941	chrAC_000187.1	119143183	119145402	2220	-1.5671	1.60E-21
CNVR_942	chrAC_000187.1	119229319	119234646	5328	-2.2965	3.09E-74
CNVR_943	chrAC_000187.1	119236867	119239086	2220	-1.4358	2.42E-19
CNVR_944	chrAC_000187.1	119310571	119312346	1776	-1.2082	1.16E-12
CNVR_945	chrAC_000187.1	119353639	119361630	7992	-2.3368	4.03E-112
CNVR_946	chrAC_000187.1	119362519	119366514	3996	-2.2820	8.02E-56
CNVR_947	chrAC_000187.1	119395819	119398926	3108	-1.5327	1.08E-28
CNVR_948	chrAC_000187.1	119402923	119405142	2220	-1.9262	4.63E-27
CNVR_949	chrAC_000187.1	119412691	119414466	1776	-1.3220	3.13E-14
CNVR_950	chrAC_000187.1	119513035	119515254	2220	-1.6972	1.31E-23
CNVR_951	chrAC_000187.1	119536123	119537898	1776	-2.2233	3.52E-25
CNVR_952	chrAC_000187.1	120318451	120320670	2220	-1.2907	7.16E-17
CNVR_953	chrAC_000187.1	122129971	122131746	1776	-1.7046	2.68E-19
CNVR_954	chrAC_000187.1	122226319	122228094	1776	-1.4530	5.14E-16
CNVR_955	chrAC_000187.1	122994439	122996214	1776	-1.6039	5.18E-18
CNVR_956	chrAC_000187.1	123373615	123375834	2220	-1.3372	1.14E-17
CNVR_957	chrAC_000187.1	125023075	125024850	1776	-1.3598	9.47E-15
CNVR_958	chrAC_000187.1	125348527	125350302	1776	-1.2008	1.47E-12
CNVR_959	chrAC_000187.1	127394035	127395810	1776	-1.7169	1.87E-19
CNVR_960	chrAC_000187.1	129011083	129012858	1776	-1.6279	2.53E-18
CNVR_961	chrAC_000187.1	129153607	129155826	2220	-1.2849	8.99E-17
CNVR_962	chrAC_000187.1	129647335	129649110	1776	-1.2955	7.24E-14
CNVR_963	chrAC_000187.1	130496263	130498926	2664	-1.2238	1.48E-18
CNVR_964	chrAC_000187.1	132023623	132025398	1776	-1.2522	2.86E-13

CNVR_965	chrAC_000187.1	135866887	135868662	1776	-3.3954	9.53E-34
CNVR_966	chrAC_000187.1	136261159	136262934	1776	-1.2914	8.25E-14
CNVR_967	chrAC_000187.1	137088331	137090550	2220	-2.9614	1.21E-38
CNVR_968	chrAC_000187.1	137123407	137125626	2220	-1.2736	1.41E-16
CNVR_969	chrAC_000187.1	137135395	137137170	1776	-1.3886	3.83E-15
CNVR_970	chrAC_000187.1	137257051	137259270	2220	-1.5104	1.37E-20
CNVR_971	chrAC_000187.1	137498587	137503026	4440	-1.3730	6.20E-35
CNVR_972	chrAC_000187.1	137755219	137756994	1776	-1.1638	4.77E-12
CNVR_973	chrAC_000187.1	137797399	137799618	2220	-1.1971	2.92E-15
CNVR_974	chrAC_000187.1	137850679	137853342	2664	-1.2140	2.35E-18
CNVR_975	chrAC_000187.1	137996311	137998086	1776	-1.5090	9.16E-17
CNVR_976	chrAC_000187.1	138011407	138013182	1776	-2.2675	1.28E-25
CNVR_977	chrAC_000187.1	138395023	138397686	2664	-1.5962	4.52E-26
CNVR_978	chrAC_000187.1	138410119	138412338	2220	-1.9805	7.81E-28
CNVR_979	chrAC_000187.1	138447859	138450522	2664	-1.3686	1.57E-21
CNVR_980	chrAC_000187.1	139199995	139202658	2664	-1.9260	3.89E-32
CNVR_981	chrAC_000187.1	139378927	139380702	1776	-1.3936	3.28E-15
CNVR_982	chrAC_000187.1	139628899	139630674	1776	-1.9759	1.53E-22
CNVR_983	chrAC_000187.1	144055579	144058686	3108	-2.9858	1.13E-53
CNVR_984	chrAC_000187.1	144059575	144063570	3996	-2.1517	6.94E-53
CNVR_985	chrAC_000187.1	144208315	144210090	1776	-1.1520	6.95E-12
CNVR_986	chrAC_000187.1	146863435	146865210	1776	-3.7935	1.72E-35
CNVR_987	chrAC_000187.1	148044475	148046250	1776	-1.1887	2.16E-12
CNVR_988	chrAC_000187.1	148352167	148355274	3108	-1.4613	4.92E-27
CNVR_989	chrAC_000187.1	148515559	148524882	9324	-2.4264	8.69E-135
CNVR_990	chrAC_000187.1	148741555	148743330	1776	-1.2723	1.51E-13
CNVR_991	chrAC_000169.1	3331	5106	1776	1.3975	2.27E-25
CNVR_992	chrAC_000169.1	5770447	5772666	2220	1.3424	1.45E-29
CNVR_993	chrAC_000169.1	6620707	6622926	2220	1.7709	1.09E-42
CNVR_994	chrAC_000169.1	32023723	32026386	2664	1.3165	3.68E-34
CNVR_995	chrAC_000169.1	32040151	32041926	1776	1.2481	1.35E-21
CNVR_996	chrAC_000169.1	32067679	32069454	1776	1.1953	2.89E-20
CNVR_997	chrAC_000169.1	32072563	32075226	2664	1.1600	2.90E-28
CNVR_998	chrAC_000169.1	32077003	32078778	1776	1.1564	2.73E-19
CNVR_999	chrAC_000169.1	32082331	32085882	3552	1.2751	6.90E-43
CNVR_1000	chrAC_000169.1	32088991	32092542	3552	1.6351	8.69E-61
CNVR_1001	chrAC_000169.1	32093875	32095650	1776	1.5272	1.28E-28
CNVR_1002	chrAC_000169.1	32096983	32100090	3108	1.2900	2.13E-38
CNVR_1003	chrAC_000169.1	32105419	32107194	1776	1.4386	2.09E-26
CNVR_1004	chrAC_000169.1	32111191	32113854	2664	1.1933	1.63E-29
CNVR_1005	chrAC_000169.1	32114743	32116962	2220	1.1854	1.28E-24
CNVR_1006	chrAC_000169.1	32118739	32121402	2664	1.4799	2.57E-40
CNVR_1007	chrAC_000169.1	32122291	32124066	1776	1.2120	1.10E-20
CNVR_1008	chrAC_000169.1	32127175	32128950	1776	1.4985	6.61E-28

CNVR_1009	chrAC_000169.1	32129839	32131614	1776	1.5945	2.81E-30
CNVR_1010	chrAC_000169.1	32133391	32136942	3552	1.2571	5.55E-42
CNVR_1011	chrAC_000169.1	32137387	32141382	3996	1.4053	2.16E-55
CNVR_1012	chrAC_000169.1	32143603	32145378	1776	1.1477	4.50E-19
CNVR_1013	chrAC_000169.1	32146711	32150706	3996	1.3821	4.41E-54
CNVR_1014	chrAC_000169.1	32156479	32158698	2220	1.2148	1.53E-25
CNVR_1015	chrAC_000169.1	32166247	32168910	2664	1.2410	2.63E-31
CNVR_1016	chrAC_000169.1	32171131	32174238	3108	1.3874	1.09E-42
CNVR_1017	chrAC_000169.1	32180899	32184894	3996	1.2876	9.80E-49
CNVR_1018	chrAC_000169.1	32195995	32199990	3996	1.3731	1.43E-53
CNVR_1019	chrAC_000169.1	32203987	32207538	3552	1.4023	2.74E-49
CNVR_1020	chrAC_000169.1	32219971	32223522	3552	1.2609	3.60E-42
CNVR_1021	chrAC_000169.1	37704259	37706478	2220	1.2687	3.07E-27
CNVR_1022	chrAC_000169.1	37708699	37710918	2220	1.3820	8.23E-31
CNVR_1023	chrAC_000169.1	39224071	39226290	2220	1.2632	4.59E-27
CNVR_1024	chrAC_000169.1	39226735	39229398	2664	1.6235	1.28E-45
CNVR_1025	chrAC_000169.1	39476263	39478038	1776	1.3431	5.35E-24
CNVR_1026	chrAC_000169.1	49238491	49240266	1776	1.2381	2.42E-21
CNVR_1027	chrAC_000169.1	61332163	61335714	3552	1.8913	1.04E-72
CNVR_1028	chrAC_000169.1	65199403	65201178	1776	1.9922	1.87E-39
CNVR_1029	chrAC_000169.1	69507979	69510198	2220	1.4351	1.76E-32
CNVR_1030	chrAC_000169.1	70403083	70405746	2664	1.6595	6.40E-47
CNVR_1031	chrAC_000169.1	70472791	70475454	2664	1.4512	3.06E-39
CNVR_1032	chrAC_000169.1	70570915	70573134	2220	1.4328	2.07E-32
CNVR_1033	chrAC_000169.1	70580683	70583346	2664	1.3064	8.85E-34
CNVR_1034	chrAC_000169.1	70602883	70605102	2220	1.3133	1.21E-28
CNVR_1035	chrAC_000169.1	70624639	70626414	1776	1.1857	5.04E-20
CNVR_1036	chrAC_000169.1	70677031	70678806	1776	1.3139	2.94E-23
CNVR_1037	chrAC_000169.1	71295967	71302182	6216	1.2264	1.97E-69
CNVR_1038	chrAC_000169.1	71828767	71830542	1776	1.2519	1.08E-21
CNVR_1039	chrAC_000169.1	71834539	71836758	2220	1.4136	8.30E-32
CNVR_1040	chrAC_000169.1	71838979	71840754	1776	1.4120	9.79E-26
CNVR_1041	chrAC_000169.1	71844307	71846526	2220	1.5748	8.07E-37
CNVR_1042	chrAC_000169.1	71860291	71862510	2220	1.5848	3.99E-37
CNVR_1043	chrAC_000169.1	71897587	71899362	1776	1.2169	8.29E-21
CNVR_1044	chrAC_000169.1	72213271	72231474	18204	1.4323	2.20E-252
CNVR_1045	chrAC_000169.1	72620419	72622194	1776	1.4020	1.74E-25
CNVR_1046	chrAC_000169.1	72633739	72636402	2664	1.2467	1.60E-31
CNVR_1047	chrAC_000169.1	72699451	72701226	1776	1.2169	8.29E-21
CNVR_1048	chrAC_000169.1	72922783	72924558	1776	1.2963	8.21E-23
CNVR_1049	chrAC_000169.1	72940099	72941874	1776	1.4680	3.84E-27
CNVR_1050	chrAC_000169.1	72942319	72945426	3108	1.4859	5.26E-47
CNVR_1051	chrAC_000169.1	72947647	72949866	2220	1.5054	1.12E-34
CNVR_1052	chrAC_000169.1	72957859	72960078	2220	1.2886	7.28E-28

CNVR_1053	chrAC_000169.1	72962299	72964074	1776	1.3314	1.06E-23
CNVR_1054	chrAC_000169.1	72964963	72967626	2664	1.4175	5.66E-38
CNVR_1055	chrAC_000169.1	72968959	72973398	4440	1.6919	6.35E-79
CNVR_1056	chrAC_000169.1	72978727	72980502	1776	1.3544	2.77E-24
CNVR_1057	chrAC_000169.1	72991603	72998262	6660	1.6182	8.54E-111
CNVR_1058	chrAC_000169.1	73000483	73004034	3552	1.4301	1.11E-50
CNVR_1059	chrAC_000169.1	73023571	73025790	2220	1.1801	1.87E-24
CNVR_1060	chrAC_000169.1	73051099	73053318	2220	1.2950	4.56E-28
CNVR_1061	chrAC_000169.1	73068859	73071078	2220	1.3075	1.83E-28
CNVR_1062	chrAC_000169.1	73071523	73075074	3552	1.3671	1.62E-47
CNVR_1063	chrAC_000169.1	73083955	73085730	1776	1.2600	6.79E-22
CNVR_1064	chrAC_000169.1	73095055	73096830	1776	1.2665	4.65E-22
CNVR_1065	chrAC_000169.1	73100827	73110150	9324	1.4046	1.20E-126
CNVR_1066	chrAC_000169.1	73112815	73115478	2664	1.3607	7.83E-36
CNVR_1067	chrAC_000169.1	73115923	73119474	3552	1.4054	1.92E-49
CNVR_1068	chrAC_000169.1	73138123	73140342	2220	1.3200	7.41E-29
CNVR_1069	chrAC_000169.1	73244239	73247346	3108	1.3959	4.60E-43
CNVR_1070	chrAC_000169.1	73265995	73270434	4440	1.2197	1.08E-49
CNVR_1071	chrAC_000169.1	73271323	73275318	3996	1.1194	2.73E-39
CNVR_1072	chrAC_000169.1	73487107	73488882	1776	1.6219	6.04E-31
CNVR_1073	chrAC_000169.1	73647391	73650498	3108	1.3593	1.88E-41
CNVR_1074	chrAC_000169.1	73655827	73658046	2220	1.2118	1.91E-25
CNVR_1075	chrAC_000169.1	73686907	73690014	3108	1.4873	4.59E-47
CNVR_1076	chrAC_000169.1	73695343	73698894	3552	1.7371	1.19E-65
CNVR_1077	chrAC_000169.1	73704223	73705998	1776	1.4019	1.75E-25
CNVR_1078	chrAC_000169.1	73707775	73709994	2220	1.3934	3.59E-31
CNVR_1079	chrAC_000169.1	73733527	73737078	3552	1.7320	2.08E-65
CNVR_1080	chrAC_000169.1	73791691	73794798	3108	1.2874	2.78E-38
CNVR_1081	chrAC_000169.1	73795687	73798794	3108	1.2834	4.14E-38
CNVR_1082	chrAC_000169.1	73813447	73820550	7104	1.6425	2.13E-120
CNVR_1083	chrAC_000169.1	73820995	73822770	1776	1.3982	2.18E-25
CNVR_1084	chrAC_000169.1	73824547	73829430	4884	1.1975	5.93E-53
CNVR_1085	chrAC_000169.1	73832983	73834758	1776	1.2207	6.64E-21
CNVR_1086	chrAC_000169.1	73835203	73851630	16428	1.6238	1.51E-271
CNVR_1087	chrAC_000169.1	73959079	73968846	9768	1.7171	1.47E-174
CNVR_1088	chrAC_000169.1	73975507	73982610	7104	1.5566	4.85E-112
CNVR_1089	chrAC_000169.1	74023903	74028786	4884	1.2450	3.20E-56
CNVR_1090	chrAC_000169.1	74030563	74033670	3108	1.2657	2.50E-37
CNVR_1091	chrAC_000169.1	74046547	74048322	1776	1.2929	9.98E-23
CNVR_1092	chrAC_000169.1	74099383	74102490	3108	1.1964	2.75E-34
CNVR_1093	chrAC_000169.1	74103823	74114034	10212	1.4948	1.93E-151
CNVR_1094	chrAC_000169.1	74115367	74118474	3108	1.2847	3.63E-38
CNVR_1095	chrAC_000169.1	74133127	74137122	3996	1.4385	2.92E-57
CNVR_1096	chrAC_000169.1	74170423	74172198	1776	1.8367	5.15E-36

CNVR_1097	chrAC_000169.1	74174863	74183298	8436	1.4421	3.21E-119
CNVR_1098	chrAC_000169.1	74231251	74235246	3996	1.2177	8.69E-45
CNVR_1099	chrAC_000169.1	74238355	74240130	1776	1.1220	1.96E-18
CNVR_1100	chrAC_000169.1	74306287	74308506	2220	1.2454	1.67E-26
CNVR_1101	chrAC_000169.1	74690347	74692122	1776	1.6806	2.31E-32
CNVR_1102	chrAC_000169.1	74700115	74703666	3552	1.2078	1.66E-39
CNVR_1103	chrAC_000169.1	74787583	74789802	2220	1.6038	1.05E-37
CNVR_1104	chrAC_000169.1	74790691	74795130	4440	1.6570	7.68E-77
CNVR_1105	chrAC_000169.1	74795575	74797350	1776	1.1032	5.72E-18
CNVR_1106	chrAC_000169.1	74799127	74814666	15540	1.5522	9.81E-242
CNVR_1107	chrAC_000169.1	74816443	74820438	3996	1.6199	2.51E-67
CNVR_1108	chrAC_000169.1	74853295	74855514	2220	1.4958	2.23E-34
CNVR_1109	chrAC_000169.1	74858179	74859954	1776	1.6743	3.28E-32
CNVR_1110	chrAC_000169.1	75122359	75124134	1776	1.1856	5.08E-20
CNVR_1111	chrAC_000169.1	75184519	75186738	2220	1.2541	8.87E-27
CNVR_1112	chrAC_000169.1	75187183	75188958	1776	1.2474	1.41E-21
CNVR_1113	chrAC_000169.1	75406519	75408294	1776	1.2947	8.99E-23
CNVR_1114	chrAC_000169.1	75561919	75566358	4440	1.2815	1.44E-53
CNVR_1115	chrAC_000169.1	75715543	75718206	2664	1.1102	2.05E-26
CNVR_1116	chrAC_000169.1	75719095	75721314	2220	1.2643	4.23E-27
CNVR_1117	chrAC_000169.1	75723535	75727086	3552	1.1943	7.79E-39
CNVR_1118	chrAC_000169.1	75787027	75788802	1776	1.1928	3.33E-20
CNVR_1119	chrAC_000169.1	75799015	75800790	1776	1.0447	1.56E-16
CNVR_1120	chrAC_000169.1	75894475	75896694	2220	1.1732	3.07E-24
CNVR_1121	chrAC_000169.1	75898027	75902466	4440	1.7425	6.57E-82
CNVR_1122	chrAC_000169.1	75904687	75908682	3996	1.6627	1.19E-69
CNVR_1123	chrAC_000169.1	76076071	76078734	2664	1.5556	3.94E-43
CNVR_1124	chrAC_000169.1	76079179	76082286	3108	1.7014	3.39E-56
CNVR_1125	chrAC_000169.1	76236355	76238574	2220	1.5817	4.95E-37
CNVR_1126	chrAC_000169.1	76273207	76274982	1776	1.1964	2.71E-20
CNVR_1127	chrAC_000169.1	76279867	76282974	3108	1.7279	2.73E-57
CNVR_1128	chrAC_000169.1	76382431	76385538	3108	1.3950	5.02E-43
CNVR_1129	chrAC_000169.1	76386871	76389090	2220	1.2584	6.49E-27
CNVR_1130	chrAC_000169.1	76399747	76401522	1776	1.7000	7.96E-33
CNVR_1131	chrAC_000169.1	76409515	76411290	1776	1.7294	1.59E-33
CNVR_1132	chrAC_000169.1	76416175	76419282	3108	1.9490	4.50E-66
CNVR_1133	chrAC_000169.1	76441927	76444590	2664	2.6452	3.18E-76
CNVR_1134	chrAC_000169.1	76473007	76474782	1776	1.1925	3.40E-20
CNVR_1135	chrAC_000169.1	76476559	76479666	3108	1.6774	3.39E-55
CNVR_1136	chrAC_000169.1	76488991	76492098	3108	1.2988	8.67E-39
CNVR_1137	chrAC_000169.1	76505863	76512966	7104	1.3847	5.82E-95
CNVR_1138	chrAC_000169.1	76515631	76519626	3996	1.3637	4.84E-53
CNVR_1139	chrAC_000169.1	76580011	76585338	5328	1.7110	1.10E-95
CNVR_1140	chrAC_000169.1	76586227	76588890	2664	1.3654	5.21E-36

CNVR_1141	chrAC_000169.1	76589335	76593330	3996	1.5464	2.72E-63
CNVR_1142	chrAC_000169.1	76593775	76605318	11544	1.4499	1.15E-163
CNVR_1143	chrAC_000169.1	76607095	76611534	4440	1.3676	5.62E-59
CNVR_1144	chrAC_000169.1	86527831	86530050	2220	1.9634	4.20E-48
CNVR_1145	chrAC_000169.1	867799	869574	1776	-1.0879	2.42E-15
CNVR_1146	chrAC_000169.1	935731	945942	10212	-3.6657	3.26E-279
CNVR_1147	chrAC_000169.1	1827727	1829946	2220	-1.2483	8.73E-23
CNVR_1148	chrAC_000169.1	4034851	4037070	2220	-2.2073	3.74E-44
CNVR_1149	chrAC_000169.1	7586407	7588626	2220	-1.4629	4.36E-28
CNVR_1150	chrAC_000169.1	7648123	7650342	2220	-1.4668	3.51E-28
CNVR_1151	chrAC_000169.1	8503711	8505930	2220	-1.6293	4.72E-32
CNVR_1152	chrAC_000169.1	15567751	15570414	2664	-1.4932	3.00E-34
CNVR_1153	chrAC_000169.1	17400583	17402802	2220	-1.8376	9.56E-37
CNVR_1154	chrAC_000169.1	17673199	17675418	2220	-2.1995	5.21E-44
CNVR_1155	chrAC_000169.1	20763439	20765214	1776	-1.1217	5.14E-16
CNVR_1156	chrAC_000169.1	26689507	26692170	2664	-1.2065	9.08E-26
CNVR_1157	chrAC_000169.1	31982875	31984650	1776	-1.1523	1.26E-16
CNVR_1158	chrAC_000169.1	33957787	33959562	1776	-1.7544	2.89E-28
CNVR_1159	chrAC_000169.1	39078439	39080658	2220	-1.8275	1.59E-36
CNVR_1160	chrAC_000169.1	39604579	39606354	1776	-1.2519	1.28E-18
CNVR_1161	chrAC_000169.1	40415323	40417986	2664	-1.3665	1.54E-30
CNVR_1162	chrAC_000169.1	40870867	40872642	1776	-1.3638	7.55E-21
CNVR_1163	chrAC_000169.1	41745103	41747766	2664	-2.3456	1.45E-55
CNVR_1164	chrAC_000169.1	41772187	41774406	2220	-1.6272	5.28E-32
CNVR_1165	chrAC_000169.1	43869643	43871862	2220	-1.2775	1.62E-23
CNVR_1166	chrAC_000169.1	48330511	48334506	3996	-1.1959	2.43E-37
CNVR_1167	chrAC_000169.1	50175331	50177550	2220	-1.6312	4.26E-32
CNVR_1168	chrAC_000169.1	50401327	50403102	1776	-1.5607	1.11E-24
CNVR_1169	chrAC_000169.1	54744535	54747198	2664	-1.5751	1.35E-36
CNVR_1170	chrAC_000169.1	55924687	55926462	1776	-1.8564	4.65E-30
CNVR_1171	chrAC_000169.1	56802475	56805138	2664	-1.2136	5.57E-26
CNVR_1172	chrAC_000169.1	57582583	57587022	4440	-1.6381	9.20E-63
CNVR_1173	chrAC_000169.1	57936007	57937782	1776	-1.2950	1.77E-19
CNVR_1174	chrAC_000169.1	58201519	58203738	2220	-1.2124	6.86E-22
CNVR_1175	chrAC_000169.1	58402651	58404870	2220	-1.6423	2.35E-32
CNVR_1176	chrAC_000169.1	58611331	58613106	1776	-1.3027	1.24E-19
CNVR_1177	chrAC_000169.1	58742755	58744530	1776	-2.5459	3.19E-40
CNVR_1178	chrAC_000169.1	59980627	59983290	2664	-1.7619	9.44E-42
CNVR_1179	chrAC_000169.1	60145795	60148014	2220	-1.2425	1.21E-22
CNVR_1180	chrAC_000169.1	61207843	61209618	1776	-1.1721	5.06E-17
CNVR_1181	chrAC_000169.1	61353475	61357914	4440	-3.3560	2.27E-117
CNVR_1182	chrAC_000169.1	63497995	63499770	1776	-1.5705	7.26E-25
CNVR_1183	chrAC_000169.1	63777271	63779046	1776	-1.5168	7.70E-24
CNVR_1184	chrAC_000169.1	64650175	64651950	1776	-2.1411	1.11E-34

CNVR_1185	chrAC_000169.1	67546387	67548162	1776	-1.3356	2.74E-20
CNVR_1186	chrAC_000169.1	70320499	70322274	1776	-1.8800	1.83E-30
CNVR_1187	chrAC_000169.1	70322719	70325382	2664	-2.4999	1.36E-58
CNVR_1188	chrAC_000169.1	70639735	70641510	1776	-1.1093	9.12E-16
CNVR_1189	chrAC_000169.1	70818667	70824882	6216	-2.3215	5.57E-126
CNVR_1190	chrAC_000169.1	70831987	70835982	3996	-1.7757	2.77E-62
CNVR_1191	chrAC_000169.1	70836871	70843974	7104	-1.6516	2.71E-100
CNVR_1192	chrAC_000169.1	70848415	70854630	6216	-1.5027	3.51E-78
CNVR_1193	chrAC_000169.1	70857739	70861290	3552	-1.6442	8.49E-51
CNVR_1194	chrAC_000169.1	70865287	70868838	3552	-2.4718	8.47E-77
CNVR_1195	chrAC_000169.1	70875499	70877274	1776	-1.6663	1.16E-26
CNVR_1196	chrAC_000169.1	70878163	70880826	2664	-2.0268	1.77E-48
CNVR_1197	chrAC_000169.1	70881715	70887042	5328	-1.7377	2.13E-80
CNVR_1198	chrAC_000169.1	70887487	70889262	1776	-1.4607	9.41E-23
CNVR_1199	chrAC_000169.1	70891483	70893258	1776	-1.4048	1.17E-21
CNVR_1200	chrAC_000169.1	70895479	70897698	2220	-2.9139	6.81E-55
CNVR_1201	chrAC_000169.1	70899031	70901250	2220	-1.9995	3.84E-40
CNVR_1202	chrAC_000169.1	70901695	70903914	2220	-2.6528	2.01E-51
CNVR_1203	chrAC_000169.1	70904803	70907910	3108	-1.8800	5.11E-52
CNVR_1204	chrAC_000169.1	70908355	70910130	1776	-1.5629	1.01E-24
CNVR_1205	chrAC_000169.1	70911463	70940322	28860	-2.8588	0.00E+00
CNVR_1206	chrAC_000169.1	70941655	70945650	3996	-1.9504	5.13E-69
CNVR_1207	chrAC_000169.1	70946539	70952310	5772	-2.3738	1.95E-119
CNVR_1208	chrAC_000169.1	70954087	70957638	3552	-1.8662	1.02E-58
CNVR_1209	chrAC_000169.1	70958971	70961634	2664	-2.2581	9.84E-54
CNVR_1210	chrAC_000169.1	70965631	70967406	1776	-3.1626	1.56E-46
CNVR_1211	chrAC_000169.1	70973179	70975398	2220	-1.9033	3.75E-38
CNVR_1212	chrAC_000169.1	70980283	70982502	2220	-2.3269	2.79E-46
CNVR_1213	chrAC_000169.1	70982947	70985166	2220	-1.6087	1.43E-31
CNVR_1214	chrAC_000169.1	70992271	70994046	1776	-1.5697	7.50E-25
CNVR_1215	chrAC_000169.1	70994935	70999374	4440	-2.1952	4.82E-86
CNVR_1216	chrAC_000169.1	71000263	71006478	6216	-3.0414	1.73E-154
CNVR_1217	chrAC_000169.1	71019355	71021130	1776	-2.1494	8.31E-35
CNVR_1218	chrAC_000169.1	71041555	71046438	4884	-2.1981	1.42E-94
CNVR_1219	chrAC_000169.1	71047771	71052654	4884	-2.4308	1.97E-103
CNVR_1220	chrAC_000169.1	71054875	71107710	52836	-2.6152	0.00E+00
CNVR_1221	chrAC_000169.1	71108155	71128578	20424	-2.5430	0.00E+00
CNVR_1222	chrAC_000169.1	71129023	71133462	4440	-1.5019	2.47E-56
CNVR_1223	chrAC_000169.1	71141899	71146782	4884	-1.6158	1.03E-67
CNVR_1224	chrAC_000169.1	71147227	71151666	4440	-2.5384	1.33E-97
CNVR_1225	chrAC_000169.1	71165875	71168094	2220	-4.1170	3.52E-65
CNVR_1226	chrAC_000169.1	71172979	71175198	2220	-1.3896	2.71E-26
CNVR_1227	chrAC_000169.1	71176531	71183634	7104	-2.4400	5.05E-150
CNVR_1228	chrAC_000169.1	71186743	71189850	3108	-1.7008	1.72E-46

CNVR_1229	chrAC_000169.1	71194291	71196954	2664	-1.3226	3.11E-29
CNVR_1230	chrAC_000169.1	71197399	71199174	1776	-1.4895	2.60E-23
CNVR_1231	chrAC_000169.1	71203171	71206278	3108	-2.7179	1.53E-72
CNVR_1232	chrAC_000169.1	71206723	71215158	8436	-2.1822	8.41E-161
CNVR_1233	chrAC_000169.1	71331487	71336370	4884	-1.5320	2.27E-63
CNVR_1234	chrAC_000169.1	71347471	71349690	2220	-1.7546	6.44E-35
CNVR_1235	chrAC_000169.1	71353687	71358126	4440	-2.6851	6.64E-102
CNVR_1236	chrAC_000169.1	71360791	71364342	3552	-2.0561	5.73E-65
CNVR_1237	chrAC_000169.1	71368339	71370558	2220	-1.9306	1.00E-38
CNVR_1238	chrAC_000169.1	71371447	71376774	5328	-1.8570	1.12E-86
CNVR_1239	chrAC_000169.1	71378551	71383434	4884	-1.7693	2.11E-75
CNVR_1240	chrAC_000169.1	71390095	71396754	6660	-2.0741	3.30E-121
CNVR_1241	chrAC_000169.1	71402083	71404746	2664	-2.2539	1.21E-53
CNVR_1242	chrAC_000169.1	71405635	71407410	1776	-1.5565	1.34E-24
CNVR_1243	chrAC_000169.1	71411407	71415402	3996	-2.4107	2.19E-84
CNVR_1244	chrAC_000169.1	71415847	71418954	3108	-2.6961	4.04E-72
CNVR_1245	chrAC_000169.1	71421619	71426946	5328	-1.9854	4.07E-93
CNVR_1246	chrAC_000169.1	71427835	71434938	7104	-1.9123	1.11E-118
CNVR_1247	chrAC_000169.1	71438491	71442486	3996	-2.2448	3.00E-79
CNVR_1248	chrAC_000169.1	71444707	71446926	2220	-1.8893	7.43E-38
CNVR_1249	chrAC_000169.1	71459359	71468238	8880	-2.5777	1.41E-195
CNVR_1250	chrAC_000169.1	71469571	71486442	16872	-2.3888	0.00E+00
CNVR_1251	chrAC_000169.1	71486887	71489550	2664	-1.6349	2.81E-38
CNVR_1252	chrAC_000169.1	71491771	71496654	4884	-1.5535	1.71E-64
CNVR_1253	chrAC_000169.1	71498875	71502870	3996	-1.9827	3.29E-70
CNVR_1254	chrAC_000169.1	71526847	71534394	7548	-2.1994	3.66E-145
CNVR_1255	chrAC_000169.1	71547271	71550378	3108	-1.6104	1.43E-43
CNVR_1256	chrAC_000169.1	71553043	71555262	2220	-1.7619	4.43E-35
CNVR_1257	chrAC_000169.1	71556595	71576130	19536	-2.2785	0.00E+00
CNVR_1258	chrAC_000169.1	71576575	71581014	4440	-2.5542	4.41E-98
CNVR_1259	chrAC_000169.1	71581903	71587230	5328	-2.9712	9.32E-131
CNVR_1260	chrAC_000169.1	71587675	71591670	3996	-2.4603	7.84E-86
CNVR_1261	chrAC_000169.1	71598331	71607210	8880	-2.0579	1.14E-159
CNVR_1262	chrAC_000169.1	71608099	71611206	3108	-2.1969	7.82E-61
CNVR_1263	chrAC_000169.1	71612095	71619642	7548	-2.0014	3.38E-132
CNVR_1264	chrAC_000169.1	71623195	71624970	1776	-1.6845	5.35E-27
CNVR_1265	chrAC_000169.1	71628967	71640954	11988	-2.2629	1.55E-235
CNVR_1266	chrAC_000169.1	71641399	71647614	6216	-2.5836	9.16E-138
CNVR_1267	chrAC_000169.1	71652499	71654274	1776	-2.9306	1.96E-44
CNVR_1268	chrAC_000169.1	71681359	71692458	11100	-3.0244	2.92E-273
CNVR_1269	chrAC_000169.1	71761723	71763942	2220	-1.7587	5.24E-35
CNVR_1270	chrAC_000169.1	71941987	71944206	2220	-2.8560	3.60E-54
CNVR_1271	chrAC_000169.1	71945539	71951754	6216	-2.2627	4.20E-123
CNVR_1272	chrAC_000169.1	71952643	71954862	2220	-2.1759	1.42E-43

CNVR_1273	chrAC_000169.1	71959303	71964186	4884	-2.0953	2.61E-90
CNVR_1274	chrAC_000169.1	71964631	71966850	2220	-1.8734	1.62E-37
CNVR_1275	chrAC_000169.1	71969959	71972622	2664	-1.6307	3.66E-38
CNVR_1276	chrAC_000169.1	71974399	71977950	3552	-2.8773	5.43E-86
CNVR_1277	chrAC_000169.1	71978839	71982390	3552	-1.7648	3.53E-55
CNVR_1278	chrAC_000169.1	71987719	71989494	1776	-1.7750	1.24E-28
CNVR_1279	chrAC_000169.1	71990827	71993046	2220	-1.8326	1.23E-36
CNVR_1280	chrAC_000169.1	71995711	72006810	11100	-2.4265	3.73E-232
CNVR_1281	chrAC_000169.1	72012139	72024570	12432	-2.0226	8.54E-219
CNVR_1282	chrAC_000169.1	72027235	72029454	2220	-1.5979	2.57E-31
CNVR_1283	chrAC_000169.1	72092503	72095166	2664	-1.7934	1.38E-42
CNVR_1284	chrAC_000169.1	72096055	72097830	1776	-1.9488	1.27E-31
CNVR_1285	chrAC_000169.1	72467239	72471678	4440	-1.1854	8.90E-41
CNVR_1286	chrAC_000169.1	72474787	72476562	1776	-1.2087	9.37E-18
CNVR_1287	chrAC_000169.1	72733639	72735414	1776	-1.4649	7.79E-23
CNVR_1288	chrAC_000169.1	72745183	72761166	15984	-2.5168	0.00E+00
CNVR_1289	chrAC_000169.1	72762499	72764718	2220	-1.7421	1.23E-34
CNVR_1290	chrAC_000169.1	72765163	72767826	2664	-2.3754	3.60E-56
CNVR_1291	chrAC_000169.1	72770047	72781146	11100	-3.5015	9.34E-297
CNVR_1292	chrAC_000169.1	72781591	72784254	2664	-1.6478	1.23E-38
CNVR_1293	chrAC_000169.1	72785143	72789582	4440	-2.6149	6.86E-100
CNVR_1294	chrAC_000169.1	72790471	72800682	10212	-2.9246	3.61E-246
CNVR_1295	chrAC_000169.1	72801127	72806898	5772	-3.1422	1.82E-146
CNVR_1296	chrAC_000169.1	72807343	72815334	7992	-2.1872	8.91E-153
CNVR_1297	chrAC_000169.1	72817111	72823326	6216	-2.5402	6.22E-136
CNVR_1298	chrAC_000169.1	72829987	72833538	3552	-1.9493	1.61E-61
CNVR_1299	chrAC_000169.1	72834427	72837534	3108	-1.6786	8.80E-46
CNVR_1300	chrAC_000169.1	72838423	72840198	1776	-1.5678	8.16E-25
CNVR_1301	chrAC_000169.1	72865507	72867282	1776	-1.4820	3.63E-23
CNVR_1302	chrAC_000169.1	72885043	72886818	1776	-2.3147	3.33E-37
CNVR_1303	chrAC_000169.1	72893035	72894810	1776	-2.1170	2.59E-34
CNVR_1304	chrAC_000169.1	72897919	72900138	2220	-2.9571	2.04E-55
CNVR_1305	chrAC_000169.1	72900583	72904578	3996	-1.7421	6.13E-61
CNVR_1306	chrAC_000169.1	72909019	72912570	3552	-3.4065	5.08E-95
CNVR_1307	chrAC_000169.1	72916123	72919674	3552	-1.9511	1.41E-61
CNVR_1308	chrAC_000169.1	73012027	73016466	4440	-2.0811	1.01E-81
CNVR_1309	chrAC_000169.1	73301515	73317942	16428	-3.6110	0.00E+00
CNVR_1310	chrAC_000169.1	73318387	73329486	11100	-2.3997	5.92E-230
CNVR_1311	chrAC_000169.1	73357015	73362786	5772	-1.6934	2.46E-84
CNVR_1312	chrAC_000169.1	73379215	73383210	3996	-2.7063	1.93E-92
CNVR_1313	chrAC_000169.1	73383655	73387650	3996	-2.7253	6.50E-93
CNVR_1314	chrAC_000169.1	73401859	73404078	2220	-1.7242	3.11E-34
CNVR_1315	chrAC_000169.1	73552375	73554150	1776	-2.6118	5.16E-41
CNVR_1316	chrAC_000169.1	73565695	73567470	1776	-3.6024	9.41E-50

CNVR_1317	chrAC_000169.1	73622527	73624746	2220	-1.3969	1.80E-26
CNVR_1318	chrAC_000169.1	73753507	73755282	1776	-1.7421	4.81E-28
CNVR_1319	chrAC_000169.1	73894699	73896918	2220	-2.2932	1.08E-45
CNVR_1320	chrAC_000169.1	73897807	73903134	5328	-2.5917	1.17E-118
CNVR_1321	chrAC_000169.1	73923115	73925334	2220	-1.7391	1.44E-34
CNVR_1322	chrAC_000169.1	73994155	73995930	1776	-2.1881	2.19E-35
CNVR_1323	chrAC_000169.1	74057647	74059422	1776	-2.0961	5.45E-34
CNVR_1324	chrAC_000169.1	74060311	74062974	2664	-1.5774	1.16E-36
CNVR_1325	chrAC_000169.1	74063863	74066082	2220	-2.0101	2.34E-40
CNVR_1326	chrAC_000169.1	74140231	74143338	3108	-2.8502	5.32E-75
CNVR_1327	chrAC_000169.1	74143783	74147334	3552	-1.8871	1.97E-59
CNVR_1328	chrAC_000169.1	74153107	74154882	1776	-1.8452	7.24E-30
CNVR_1329	chrAC_000169.1	74194399	74197062	2664	-1.6840	1.22E-39
CNVR_1330	chrAC_000169.1	74278759	74282310	3552	-1.9435	2.51E-61
CNVR_1331	chrAC_000169.1	74282755	74288082	5328	-2.1764	4.94E-102
CNVR_1332	chrAC_000169.1	74321827	74330706	8880	-1.9206	1.52E-148
CNVR_1333	chrAC_000169.1	74331151	74333814	2664	-1.7427	3.10E-41
CNVR_1334	chrAC_000169.1	74334259	74338698	4440	-1.8054	2.61E-70
CNVR_1335	chrAC_000169.1	74344471	74347134	2664	-2.1566	1.68E-51
CNVR_1336	chrAC_000169.1	74348467	74350242	1776	-1.7917	6.25E-29
CNVR_1337	chrAC_000169.1	74398639	74400414	1776	-2.1257	1.91E-34
CNVR_1338	chrAC_000169.1	74430163	74432382	2220	-2.3260	2.89E-46
CNVR_1339	chrAC_000169.1	74451031	74455470	4440	-1.4748	4.97E-55
CNVR_1340	chrAC_000169.1	74457247	74462130	4884	-3.9015	3.03E-138
CNVR_1341	chrAC_000169.1	74464351	74467902	3552	-1.3995	1.91E-41
CNVR_1342	chrAC_000169.1	74469235	74475006	5772	-2.3792	1.13E-119
CNVR_1343	chrAC_000169.1	74476783	74493210	16428	-2.5700	0.00E+00
CNVR_1344	chrAC_000169.1	74496763	74504754	7992	-2.1768	4.36E-152
CNVR_1345	chrAC_000169.1	74505643	74507862	2220	-2.3274	2.73E-46
CNVR_1346	chrAC_000169.1	74530951	74534058	3108	-1.7670	1.42E-48
CNVR_1347	chrAC_000169.1	74534503	74558478	23976	-2.9035	0.00E+00
CNVR_1348	chrAC_000169.1	74560255	74562474	2220	-1.6793	3.29E-33
CNVR_1349	chrAC_000169.1	74570467	74573130	2664	-1.8164	3.40E-43
CNVR_1350	chrAC_000169.1	74578459	74583786	5328	-2.3316	1.10E-108
CNVR_1351	chrAC_000169.1	74584231	74588670	4440	-2.8251	1.11E-105
CNVR_1352	chrAC_000169.1	74590891	74593110	2220	-1.9681	1.68E-39
CNVR_1353	chrAC_000169.1	74605543	74622858	17316	-3.1811	0.00E+00
CNVR_1354	chrAC_000169.1	74623747	74636622	12876	-3.4182	0.00E+00
CNVR_1355	chrAC_000169.1	74637511	74640174	2664	-2.2575	1.02E-53
CNVR_1356	chrAC_000169.1	74660155	74661930	1776	-2.5226	6.16E-40
CNVR_1357	chrAC_000169.1	75160099	75161874	1776	-1.3738	4.80E-21
CNVR_1358	chrAC_000169.1	75216931	75219150	2220	-1.4702	2.90E-28
CNVR_1359	chrAC_000169.1	75231583	75233358	1776	-1.6576	1.68E-26
CNVR_1360	chrAC_000169.1	75599215	75600990	1776	-1.2600	8.83E-19

CNVR_1361	chrAC_000169.1	75961519	75965070	3552	-2.6069	4.15E-80
CNVR_1362	chrAC_000169.1	75968623	75971286	2664	-1.6814	1.44E-39
CNVR_1363	chrAC_000169.1	76102711	76104486	1776	-2.0679	1.51E-33
CNVR_1364	chrAC_000169.1	76250563	76252782	2220	-1.9715	1.43E-39
CNVR_1365	chrAC_000169.1	76253227	76255002	1776	-1.8087	3.14E-29
CNVR_1366	chrAC_000169.1	86891467	86893242	1776	-1.7561	2.70E-28
CNVR_1367	chrAC_000169.1	90793339	90795558	2220	-1.4397	1.60E-27
CNVR_1368	chrAC_000170.1	11079355	11081130	1776	1.0847	1.47E-24
CNVR_1369	chrAC_000170.1	11093119	11097558	4440	1.1789	3.20E-67
CNVR_1370	chrAC_000170.1	11098891	11101110	2220	1.2095	7.10E-36
CNVR_1371	chrAC_000170.1	11441215	11442990	1776	1.1537	5.06E-27
CNVR_1372	chrAC_000170.1	11495827	11497602	1776	1.0790	2.34E-24
CNVR_1373	chrAC_000170.1	16983667	16987662	3996	1.4464	1.75E-82
CNVR_1374	chrAC_000170.1	41441407	41443182	1776	1.3100	1.08E-32
CNVR_1375	chrAC_000170.1	52102291	52104066	1776	2.0568	3.44E-58
CNVR_1376	chrAC_000170.1	53888947	53890722	1776	1.2849	8.87E-32
CNVR_1377	chrAC_000170.1	53933347	53936010	2664	1.5862	4.13E-63
CNVR_1378	chrAC_000170.1	53936455	53938230	1776	1.4440	1.43E-37
CNVR_1379	chrAC_000170.1	53939563	53944002	4440	1.6200	6.76E-107
CNVR_1380	chrAC_000170.1	53950219	53952882	2664	1.4324	7.54E-55
CNVR_1381	chrAC_000170.1	53953771	53957766	3996	1.3939	3.24E-78
CNVR_1382	chrAC_000170.1	53958211	53959986	1776	1.4506	8.23E-38
CNVR_1383	chrAC_000170.1	53962207	53967534	5328	1.2897	2.68E-92
CNVR_1384	chrAC_000170.1	53967979	53971086	3108	1.2894	1.36E-54
CNVR_1385	chrAC_000170.1	53971531	53974194	2664	1.2084	1.21E-42
CNVR_1386	chrAC_000170.1	70146451	70150002	3552	1.5276	2.31E-79
CNVR_1387	chrAC_000170.1	1282939	1284714	1776	-1.7505	1.31E-39
CNVR_1388	chrAC_000170.1	2435119	2436894	1776	-1.5990	1.23E-35
CNVR_1389	chrAC_000170.1	5187919	5189694	1776	-2.0344	1.75E-46
CNVR_1390	chrAC_000170.1	5615491	5617710	2220	-1.2011	3.11E-30
CNVR_1391	chrAC_000170.1	8227987	8229762	1776	-1.5461	3.33E-34
CNVR_1392	chrAC_000170.1	8474851	8477514	2664	-2.6342	1.86E-86
CNVR_1393	chrAC_000170.1	9226543	9228318	1776	-1.5036	4.86E-33
CNVR_1394	chrAC_000170.1	13445431	13447650	2220	-1.3400	3.65E-35
CNVR_1395	chrAC_000170.1	15117091	15119310	2220	-1.1647	6.14E-29
CNVR_1396	chrAC_000170.1	16098775	16100550	1776	-1.3896	7.27E-30
CNVR_1397	chrAC_000170.1	16747015	16748790	1776	-1.3796	1.39E-29
CNVR_1398	chrAC_000170.1	22505695	22507914	2220	-6.4093	1.21E-101
CNVR_1399	chrAC_000170.1	23700499	23702274	1776	-1.3212	6.19E-28
CNVR_1400	chrAC_000170.1	31537099	31538874	1776	-1.5865	2.69E-35
CNVR_1401	chrAC_000170.1	38103415	38105634	2220	-1.4805	4.48E-40
CNVR_1402	chrAC_000170.1	40195987	40198206	2220	-1.3090	4.56E-34
CNVR_1403	chrAC_000170.1	41429419	41435634	6216	-3.9141	3.51E-250
CNVR_1404	chrAC_000170.1	48015715	48017490	1776	-1.3705	2.51E-29

CNVR_1405	chrAC_000170.1	53929351	53931126	1776	-1.2393	1.32E-25
CNVR_1406	chrAC_000170.1	56578255	56580030	1776	-2.6546	1.35E-58
CNVR_1407	chrAC_000170.1	57576367	57579030	2664	-1.2662	1.02E-38
CNVR_1408	chrAC_000170.1	63433171	63434946	1776	-2.8236	3.15E-61
CNVR_1409	chrAC_000170.1	63451375	63453150	1776	-1.8970	2.94E-43
CNVR_1410	chrAC_000170.1	63471799	63474462	2664	-1.3932	4.28E-44
CNVR_1411	chrAC_000170.1	74216599	74218374	1776	-1.3138	1.00E-27
CNVR_1412	chrAC_000170.1	74223259	74225034	1776	-1.1415	8.07E-23
CNVR_1413	chrAC_000170.1	74227699	74232138	4440	-1.4361	2.81E-75
CNVR_1414	chrAC_000170.1	74970067	74974950	4884	-3.5981	6.04E-190
CNVR_1415	chrAC_000170.1	84238123	84240786	2664	-1.3411	6.74E-42
CNVR_1416	chrAC_000183.1	1791319	1793538	2220	1.4998	5.93E-42
CNVR_1417	chrAC_000183.1	1794427	1796202	1776	1.7340	6.61E-41
CNVR_1418	chrAC_000183.1	1949827	1952934	3108	1.3287	8.45E-49
CNVR_1419	chrAC_000183.1	4796755	4798530	1776	1.3812	3.01E-30
CNVR_1420	chrAC_000183.1	9349975	9351750	1776	1.1032	1.15E-21
CNVR_1421	chrAC_000183.1	19710715	19713378	2664	1.2350	9.91E-38
CNVR_1422	chrAC_000183.1	25174579	25176798	2220	1.2653	6.76E-33
CNVR_1423	chrAC_000183.1	27127735	27129954	2220	1.5886	2.54E-45
CNVR_1424	chrAC_000183.1	31582831	31584606	1776	1.1810	4.81E-24
CNVR_1425	chrAC_000183.1	31585495	31598370	12876	1.9334	0.00E+00
CNVR_1426	chrAC_000183.1	50162899	50165562	2664	1.5886	4.97E-54
CNVR_1427	chrAC_000183.1	214231	216894	2664	-2.4614	1.19E-69
CNVR_1428	chrAC_000183.1	597403	599178	1776	-1.2947	2.87E-23
CNVR_1429	chrAC_000183.1	613831	615606	1776	-1.2975	2.46E-23
CNVR_1430	chrAC_000183.1	618271	620046	1776	-2.2408	2.08E-43
CNVR_1431	chrAC_000183.1	865135	882894	17760	-3.0046	0.00E+00
CNVR_1432	chrAC_000183.1	2418247	2420022	1776	-1.5269	8.99E-29
CNVR_1433	chrAC_000183.1	5022751	5024970	2220	-1.5631	1.37E-36
CNVR_1434	chrAC_000183.1	10933723	10935498	1776	-1.2089	3.37E-21
CNVR_1435	chrAC_000183.1	14214439	14216658	2220	-2.5329	1.05E-59
CNVR_1436	chrAC_000183.1	15085123	15087342	2220	-2.0422	2.84E-49
CNVR_1437	chrAC_000183.1	15217435	15219210	1776	-1.2739	9.11E-23
CNVR_1438	chrAC_000183.1	15220099	15221874	1776	-1.6130	9.50E-31
CNVR_1439	chrAC_000183.1	15342199	15343974	1776	-1.6545	1.11E-31
CNVR_1440	chrAC_000183.1	15748459	15751566	3108	-2.4453	1.64E-80
CNVR_1441	chrAC_000183.1	24687511	24689730	2220	-1.5294	1.28E-35
CNVR_1442	chrAC_000183.1	36361159	36363378	2220	-1.7061	1.32E-40
CNVR_1443	chrAC_000183.1	47963767	47966430	2664	-2.5017	1.41E-70
CNVR_1444	chrAC_000183.1	51306199	51308418	2220	-1.1587	1.40E-24
CNVR_1445	chrAC_000162.1	9275827	9278490	2664	1.2475	4.17E-43
CNVR_1446	chrAC_000162.1	9288259	9290478	2220	1.4566	2.84E-45
CNVR_1447	chrAC_000162.1	31914943	31916718	1776	1.2690	4.72E-30
CNVR_1448	chrAC_000162.1	42296995	42299658	2664	2.3893	7.10E-96

CNVR_1449	chrAC_000162.1	44491687	44493462	1776	1.4802	2.22E-37
CNVR_1450	chrAC_000162.1	44493907	44497458	3552	2.7637	1.66E-143
CNVR_1451	chrAC_000162.1	44501011	44502786	1776	2.0360	3.71E-55
CNVR_1452	chrAC_000162.1	44539195	44542746	3552	2.1064	1.94E-112
CNVR_1453	chrAC_000162.1	65895595	65898258	2664	1.5299	9.74E-58
CNVR_1454	chrAC_000162.1	73268215	73276206	7992	1.8442	1.43E-215
CNVR_1455	chrAC_000162.1	76349131	76355790	6660	1.5294	2.02E-141
CNVR_1456	chrAC_000162.1	76968067	76969842	1776	1.4411	4.96E-36
CNVR_1457	chrAC_000162.1	79951303	79953078	1776	1.2355	6.89E-29
CNVR_1458	chrAC_000162.1	88242559	88244334	1776	1.6482	4.60E-43
CNVR_1459	chrAC_000162.1	100614619	100616838	2220	1.3644	2.74E-41
CNVR_1460	chrAC_000162.1	100618615	100622610	3996	1.1649	2.35E-57
CNVR_1461	chrAC_000162.1	100626607	100628826	2220	1.1234	7.30E-31
CNVR_1462	chrAC_000162.1	100792219	100794882	2664	1.2674	3.88E-44
CNVR_1463	chrAC_000162.1	100990687	100992462	1776	1.2153	3.45E-28
CNVR_1464	chrAC_000162.1	102453667	102455886	2220	1.4732	5.47E-46
CNVR_1465	chrAC_000162.1	102649027	102651246	2220	1.6666	3.79E-54
CNVR_1466	chrAC_000162.1	103180939	103183158	2220	1.3355	4.95E-40
CNVR_1467	chrAC_000162.1	103252867	103254642	1776	1.2771	2.46E-30
CNVR_1468	chrAC_000162.1	103359427	103361202	1776	1.2139	3.87E-28
CNVR_1469	chrAC_000162.1	103364755	103368750	3996	1.2500	5.89E-64
CNVR_1470	chrAC_000162.1	103369195	103372302	3108	1.2601	9.22E-51
CNVR_1471	chrAC_000162.1	103376743	103378518	1776	1.1164	8.80E-25
CNVR_1472	chrAC_000162.1	103403827	103405602	1776	1.4093	6.25E-35
CNVR_1473	chrAC_000162.1	103407379	103409598	2220	1.4336	2.79E-44
CNVR_1474	chrAC_000162.1	103421587	103423806	2220	1.1921	8.17E-34
CNVR_1475	chrAC_000162.1	103424251	103426026	1776	1.1029	2.53E-24
CNVR_1476	chrAC_000162.1	105907987	105912870	4884	1.8051	3.63E-129
CNVR_1477	chrAC_000162.1	117564319	117566094	1776	1.4366	7.11E-36
CNVR_1478	chrAC_000162.1	18427	21090	2664	-1.2905	2.33E-39
CNVR_1479	chrAC_000162.1	22423	25974	3552	-1.3323	3.04E-54
CNVR_1480	chrAC_000162.1	26419	29082	2664	-1.2527	9.28E-38
CNVR_1481	chrAC_000162.1	32191	33966	1776	-1.2070	1.96E-24
CNVR_1482	chrAC_000162.1	50839	53058	2220	-1.2286	6.76E-31
CNVR_1483	chrAC_000162.1	85471	87690	2220	-1.7009	4.29E-47
CNVR_1484	chrAC_000162.1	116995	119214	2220	-1.3399	7.96E-35
CNVR_1485	chrAC_000162.1	138307	140082	1776	-1.1027	1.75E-21
CNVR_1486	chrAC_000162.1	143635	145410	1776	-1.1828	9.50E-24
CNVR_1487	chrAC_000162.1	148963	150738	1776	-1.6194	7.00E-36
CNVR_1488	chrAC_000162.1	151183	153402	2220	-1.7713	2.39E-49
CNVR_1489	chrAC_000162.1	156955	159174	2220	-1.1769	4.58E-29
CNVR_1490	chrAC_000162.1	201355	203130	1776	-1.9543	2.66E-44
CNVR_1491	chrAC_000162.1	223111	225330	2220	-1.2495	1.24E-31
CNVR_1492	chrAC_000162.1	228883	231546	2664	-1.3530	5.37E-42

CNVR_1493	chrAC_000162.1	237763	239538	1776	-1.1800	1.14E-23
CNVR_1494	chrAC_000162.1	267067	268842	1776	-1.4742	6.06E-32
CNVR_1495	chrAC_000162.1	276391	287046	10656	-3.6797	0.00E+00
CNVR_1496	chrAC_000162.1	296371	300366	3996	-1.3734	2.21E-63
CNVR_1497	chrAC_000162.1	307027	309246	2220	-1.6132	3.27E-44
CNVR_1498	chrAC_000162.1	317683	319458	1776	-1.2526	9.99E-26
CNVR_1499	chrAC_000162.1	331003	333222	2220	-1.4894	5.03E-40
CNVR_1500	chrAC_000162.1	2194915	2197134	2220	-1.8525	7.08E-52
CNVR_1501	chrAC_000162.1	3281827	3287598	5772	-3.8725	3.66E-230
CNVR_1502	chrAC_000162.1	5625703	5627478	1776	-1.1692	2.30E-23
CNVR_1503	chrAC_000162.1	5634139	5635914	1776	-2.0184	8.62E-46
CNVR_1504	chrAC_000162.1	10284151	10287702	3552	-3.0532	3.81E-126
CNVR_1505	chrAC_000162.1	11608159	11610822	2664	-1.4320	2.66E-45
CNVR_1506	chrAC_000162.1	13036507	13038726	2220	-1.2830	8.11E-33
CNVR_1507	chrAC_000162.1	13047163	13049826	2664	-1.4873	1.36E-47
CNVR_1508	chrAC_000162.1	13050271	13057374	7104	-1.3060	1.14E-103
CNVR_1509	chrAC_000162.1	13856575	13858350	1776	-1.3940	1.03E-29
CNVR_1510	chrAC_000162.1	14700175	14701950	1776	-1.4997	1.21E-32
CNVR_1511	chrAC_000162.1	16071247	16073910	2664	-1.7384	1.33E-57
CNVR_1512	chrAC_000162.1	16695511	16697286	1776	-1.2136	1.27E-24
CNVR_1513	chrAC_000162.1	18733915	18741018	7104	-1.4240	7.82E-117
CNVR_1514	chrAC_000162.1	18741463	18744126	2664	-1.2077	7.54E-36
CNVR_1515	chrAC_000162.1	20715487	20718150	2664	-2.5399	1.60E-83
CNVR_1516	chrAC_000162.1	22547431	22551426	3996	-2.9419	2.38E-138
CNVR_1517	chrAC_000162.1	31463839	31466502	2664	-1.2798	6.60E-39
CNVR_1518	chrAC_000162.1	33745999	33748662	2664	-3.2570	9.05E-99
CNVR_1519	chrAC_000162.1	34538539	34540314	1776	-3.4171	3.45E-68
CNVR_1520	chrAC_000162.1	35849227	35851002	1776	-1.3708	4.60E-29
CNVR_1521	chrAC_000162.1	36304771	36307878	3108	-1.6595	2.54E-63
CNVR_1522	chrAC_000162.1	40136047	40137822	1776	-1.2859	1.14E-26
CNVR_1523	chrAC_000162.1	40596919	40598694	1776	-3.4014	5.00E-68
CNVR_1524	chrAC_000162.1	40599583	40604022	4440	-4.1807	9.47E-183
CNVR_1525	chrAC_000162.1	41922259	41925366	3108	-1.3127	1.02E-46
CNVR_1526	chrAC_000162.1	42706807	42708582	1776	-1.6432	1.64E-36
CNVR_1527	chrAC_000162.1	42710359	42713022	2664	-1.3081	4.21E-40
CNVR_1528	chrAC_000162.1	42713467	42716574	3108	-1.4922	1.84E-55
CNVR_1529	chrAC_000162.1	42718351	42722790	4440	-1.7125	4.23E-93
CNVR_1530	chrAC_000162.1	46558951	46561170	2220	-1.1535	3.05E-28
CNVR_1531	chrAC_000162.1	48662623	48664842	2220	-1.4746	1.63E-39
CNVR_1532	chrAC_000162.1	48745651	48750090	4440	-1.6220	3.50E-87
CNVR_1533	chrAC_000162.1	50323183	50325402	2220	-1.1615	1.60E-28
CNVR_1534	chrAC_000162.1	50346715	50348490	1776	-1.7046	4.04E-38
CNVR_1535	chrAC_000162.1	50352043	50356038	3996	-1.2245	5.76E-54
CNVR_1536	chrAC_000162.1	52933459	52935234	1776	-1.2630	5.06E-26

CNVR_1537	chrAC_000162.1	58020811	58070982	50172	-2.9995	0.00E+00
CNVR_1538	chrAC_000162.1	58071427	58078530	7104	-1.6610	1.97E-142
CNVR_1539	chrAC_000162.1	58081639	58083858	2220	-1.6450	2.90E-45
CNVR_1540	chrAC_000162.1	58084747	58086966	2220	-1.7292	5.25E-48
CNVR_1541	chrAC_000162.1	58087855	58090962	3108	-1.4802	6.92E-55
CNVR_1542	chrAC_000162.1	58091851	58094958	3108	-2.1645	2.07E-84
CNVR_1543	chrAC_000162.1	58095403	58127814	32412	-3.2306	0.00E+00
CNVR_1544	chrAC_000162.1	58154455	58173990	19536	-3.9760	0.00E+00
CNVR_1545	chrAC_000162.1	58229491	58231266	1776	-1.1926	5.01E-24
CNVR_1546	chrAC_000162.1	58242811	58245474	2664	-1.2489	1.35E-37
CNVR_1547	chrAC_000162.1	58380007	58441722	61716	-4.2719	0.00E+00
CNVR_1548	chrAC_000162.1	58892383	58896822	4440	-1.7078	8.58E-93
CNVR_1549	chrAC_000162.1	58897267	58903482	6216	-2.8481	1.94E-209
CNVR_1550	chrAC_000162.1	59374123	59376786	2664	-1.4485	5.48E-46
CNVR_1551	chrAC_000162.1	59377675	59383002	5328	-1.6739	2.50E-108
CNVR_1552	chrAC_000162.1	59386111	59545062	158952	-3.9152	0.00E+00
CNVR_1553	chrAC_000162.1	59545951	59547726	1776	-1.5868	5.22E-35
CNVR_1554	chrAC_000162.1	59555719	59557938	2220	-1.1966	9.15E-30
CNVR_1555	chrAC_000162.1	59573923	59575698	1776	-1.2116	1.45E-24
CNVR_1556	chrAC_000162.1	59601007	59602782	1776	-1.3384	3.76E-28
CNVR_1557	chrAC_000162.1	59606779	59608554	1776	-1.6485	1.19E-36
CNVR_1558	chrAC_000162.1	59616547	59618766	2220	-1.3046	1.39E-33
CNVR_1559	chrAC_000162.1	59628979	59631198	2220	-1.3298	1.82E-34
CNVR_1560	chrAC_000162.1	59907811	59909586	1776	-1.4146	2.73E-30
CNVR_1561	chrAC_000162.1	63466027	63467802	1776	-1.4152	2.64E-30
CNVR_1562	chrAC_000162.1	63501547	63504654	3108	-1.8049	7.80E-70
CNVR_1563	chrAC_000162.1	63726655	63729318	2664	-1.8037	4.47E-60
CNVR_1564	chrAC_000162.1	72330931	72333150	2220	-1.5940	1.43E-43
CNVR_1565	chrAC_000162.1	78410179	78411954	1776	-2.3558	6.51E-53
CNVR_1566	chrAC_000162.1	81721087	81722862	1776	-2.4785	3.30E-55
CNVR_1567	chrAC_000162.1	87085939	87087714	1776	-1.2859	1.14E-26
CNVR_1568	chrAC_000162.1	93747715	93749490	1776	-1.2772	2.01E-26
CNVR_1569	chrAC_000162.1	96369091	96370866	1776	-1.1570	5.11E-23
CNVR_1570	chrAC_000162.1	97852939	97854714	1776	-1.1916	5.36E-24
CNVR_1571	chrAC_000162.1	99584539	99586758	2220	-1.1722	6.66E-29
CNVR_1572	chrAC_000162.1	99608071	99610290	2220	-1.7910	5.76E-50
CNVR_1573	chrAC_000162.1	99612067	99617394	5328	-2.2918	3.46E-151
CNVR_1574	chrAC_000162.1	99627607	99630714	3108	-2.7366	8.57E-103
CNVR_1575	chrAC_000162.1	99631603	99635598	3996	-1.7146	4.24E-84
CNVR_1576	chrAC_000162.1	99808759	99811866	3108	-1.6139	3.27E-61
CNVR_1577	chrAC_000162.1	99813643	99816750	3108	-2.2340	5.86E-87
CNVR_1578	chrAC_000162.1	99817195	99820302	3108	-1.3312	1.24E-47
CNVR_1579	chrAC_000162.1	99821191	99822966	1776	-2.0617	9.03E-47
CNVR_1580	chrAC_000162.1	99825187	99827406	2220	-1.6326	7.45E-45

CNVR_1581	chrAC_000162.1	100062727	100064502	1776	-2.1864	1.74E-49
CNVR_1582	chrAC_000162.1	102116227	102118002	1776	-1.5958	3.00E-35
CNVR_1583	chrAC_000162.1	102444787	102446562	1776	-1.6168	8.23E-36
CNVR_1584	chrAC_000162.1	102471871	102474534	2664	-2.2803	3.22E-76
CNVR_1585	chrAC_000162.1	102475867	102481638	5772	-1.8086	2.36E-128
CNVR_1586	chrAC_000162.1	102485635	102498510	12876	-2.9355	0.00E+00
CNVR_1587	chrAC_000162.1	102498955	102502950	3996	-2.1013	6.64E-105
CNVR_1588	chrAC_000162.1	102506059	102511830	5772	-2.6948	4.74E-187
CNVR_1589	chrAC_000162.1	102513163	102518934	5772	-2.6085	1.79E-182
CNVR_1590	chrAC_000162.1	102521155	102523374	2220	-1.6290	9.74E-45
CNVR_1591	chrAC_000162.1	102532699	102536694	3996	-1.5693	1.71E-75
CNVR_1592	chrAC_000162.1	102537583	102546462	8880	-2.0015	1.90E-219
CNVR_1593	chrAC_000162.1	102546907	102549126	2220	-1.9233	5.13E-54
CNVR_1594	chrAC_000162.1	102645475	102647250	1776	-1.3998	7.10E-30
CNVR_1595	chrAC_000162.1	103712851	103715070	2220	-1.4255	8.15E-38
CNVR_1596	chrAC_000162.1	112212787	112216338	3552	-1.2775	3.71E-51
CNVR_1597	chrAC_000162.1	114244087	114245862	1776	-2.4234	3.39E-54
CNVR_1598	chrAC_000162.1	117284155	117285930	1776	-1.1320	2.60E-22
CNVR_1599	chrAC_000162.1	117388939	117391602	2664	-1.6444	6.16E-54
CNVR_1600	chrAC_000162.1	117403147	117404922	1776	-1.6782	1.97E-37
CNVR_1601	chrAC_000162.1	117405367	117412914	7548	-1.9951	3.19E-186
CNVR_1602	chrAC_000162.1	117413359	117416466	3108	-1.5364	1.43E-57
CNVR_1603	chrAC_000162.1	117418687	117423570	4884	-1.8947	1.05E-114
CNVR_1604	chrAC_000162.1	117518587	117520362	1776	-1.7009	5.04E-38
CNVR_1605	chrAC_000162.1	117584299	117587406	3108	-3.6551	8.29E-122
CNVR_1606	chrAC_000162.1	117588295	117590958	2664	-3.2034	7.51E-98
CNVR_1607	chrAC_000162.1	117592735	117597618	4884	-1.9922	5.05E-121
CNVR_1608	chrAC_000162.1	117598063	117599838	1776	-3.0585	4.30E-64
CNVR_1609	chrAC_000162.1	117602947	117605610	2664	-2.9160	2.39E-92
CNVR_1610	chrAC_000162.1	117606499	117608274	1776	-2.9937	3.00E-63
CNVR_1611	chrAC_000162.1	117610939	117612714	1776	-2.0197	3.61E-83
CNVR_1612	chrAC_000162.1	117617155	117618930	1776	-1.8334	2.21E-41
CNVR_1613	chrAC_000162.1	117619375	117621594	2220	-3.2859	4.97E-83
CNVR_1614	chrAC_000162.1	117623371	117625590	2220	-3.5422	2.74E-86
CNVR_1615	chrAC_000162.1	117638467	117642906	4440	-3.6785	5.94E-205
CNVR_1616	chrAC_000162.1	119722603	119724822	2220	-1.3349	1.20E-34
CNVR_1617	chrAC_000180.1	15102439	15104658	2220	1.3938	7.96E-35
CNVR_1618	chrAC_000180.1	17591503	17594166	2664	1.2679	4.08E-36
CNVR_1619	chrAC_000180.1	20279479	20281698	2220	1.4310	3.91E-36
CNVR_1620	chrAC_000180.1	21209659	21211434	1776	1.1029	5.34E-20
CNVR_1621	chrAC_000180.1	25435207	25437426	2220	1.2671	2.39E-30
CNVR_1622	chrAC_000180.1	27686287	27688506	2220	1.3179	3.82E-32
CNVR_1623	chrAC_000180.1	28496587	28498362	1776	1.1460	3.37E-21
CNVR_1624	chrAC_000180.1	28826923	28830918	3996	1.9234	4.19E-93

CNVR_1625	chrAC_000180.1	28838911	28846458	7548	1.7194	5.64E-152
CNVR_1626	chrAC_000180.1	43436743	43438518	1776	1.1985	1.13E-22
CNVR_1627	chrAC_000180.1	4219111	4221774	2664	-1.5504	1.73E-40
CNVR_1628	chrAC_000180.1	5418799	5421462	2664	-1.6428	1.91E-43
CNVR_1629	chrAC_000180.1	6051943	6054162	2220	-1.4886	2.43E-32
CNVR_1630	chrAC_000180.1	12957919	12960138	2220	-4.3198	5.67E-75
CNVR_1631	chrAC_000180.1	21535999	21537774	1776	-2.1672	1.40E-39
CNVR_1632	chrAC_000180.1	25337527	25341966	4440	-2.0850	1.14E-92
CNVR_1633	chrAC_000180.1	25357063	25359726	2664	-1.7561	5.80E-47
CNVR_1634	chrAC_000180.1	25366387	25373934	7548	-3.1099	3.72E-215
CNVR_1635	chrAC_000180.1	25374823	25384590	9768	-2.9912	3.89E-271
CNVR_1636	chrAC_000180.1	25385479	25392138	6660	-3.5647	1.20E-204
CNVR_1637	chrAC_000180.1	25394359	25399242	4884	-2.7057	1.67E-127
CNVR_1638	chrAC_000180.1	25406347	25413450	7104	-3.0208	5.18E-199
CNVR_1639	chrAC_000180.1	25489819	25494258	4440	-2.9427	4.61E-123
CNVR_1640	chrAC_000180.1	25710931	25714038	3108	-1.2320	1.48E-34
CNVR_1641	chrAC_000180.1	25737571	25739346	1776	-1.7342	1.96E-31
CNVR_1642	chrAC_000180.1	25740235	25742454	2220	-1.2446	1.62E-25
CNVR_1643	chrAC_000180.1	25745119	25747338	2220	-1.4439	4.14E-31
CNVR_1644	chrAC_000180.1	25749115	25753554	4440	-2.7914	9.95E-119
CNVR_1645	chrAC_000180.1	25754887	25757550	2664	-4.4308	2.67E-90
CNVR_1646	chrAC_000180.1	25761547	25769094	7548	-4.6695	5.16E-256
CNVR_1647	chrAC_000180.1	25770871	25772646	1776	-1.4075	2.09E-24
CNVR_1648	chrAC_000180.1	25776199	25777974	1776	-1.6983	1.07E-30
CNVR_1649	chrAC_000180.1	25781527	25787742	6216	-2.9086	3.67E-170
CNVR_1650	chrAC_000180.1	25788187	25789962	1776	-1.9853	2.41E-36
CNVR_1651	chrAC_000180.1	25805059	25808610	3552	-2.6394	1.48E-91
CNVR_1652	chrAC_000180.1	25809055	25811274	2220	-1.4129	3.01E-30
CNVR_1653	chrAC_000180.1	25827703	25831698	3996	-1.7584	1.21E-69
CNVR_1654	chrAC_000180.1	25835695	25839690	3996	-1.6662	2.25E-65
CNVR_1655	chrAC_000180.1	26027947	26030610	2664	-1.4956	1.06E-38
CNVR_1656	chrAC_000180.1	26134063	26135838	1776	-1.4415	3.65E-25
CNVR_1657	chrAC_000180.1	26516791	26521230	4440	-1.4218	2.96E-59
CNVR_1658	chrAC_000180.1	26531443	26533218	1776	-1.2964	6.57E-22
CNVR_1659	chrAC_000180.1	26562967	26564742	1776	-1.5207	6.58E-27
CNVR_1660	chrAC_000180.1	26604703	26606922	2220	-1.5225	2.88E-33
CNVR_1661	chrAC_000180.1	26643331	26651322	7992	-2.3062	7.21E-182
CNVR_1662	chrAC_000180.1	26651767	26654874	3108	-1.4108	1.39E-41
CNVR_1663	chrAC_000180.1	29485819	29487594	1776	-1.3258	1.43E-22
CNVR_1664	chrAC_000180.1	29720251	29722470	2220	-1.2227	6.78E-25
CNVR_1665	chrAC_000180.1	29739343	29742006	2664	-1.6177	1.20E-42
CNVR_1666	chrAC_000180.1	29781523	29783742	2220	-1.6256	4.88E-36
CNVR_1667	chrAC_000180.1	29784631	29787738	3108	-1.6474	1.46E-50
CNVR_1668	chrAC_000180.1	49778839	49781946	3108	-7.3484	4.26E-114

CNVR_1669	chrAC_000180.1	52329619	52333170	3552	-1.2122	2.37E-38
CNVR_1670	chrAC_000180.1	52426411	52428186	1776	-2.0433	2.09E-37
CNVR_1671	chrAC_000184.1	903763	905982	2220	1.1236	7.61E-67
CNVR_1672	chrAC_000184.1	5190139	5191914	1776	1.1878	1.04E-58
CNVR_1673	chrAC_000184.1	5836159	5838378	2220	1.0820	6.01E-63
CNVR_1674	chrAC_000184.1	5845927	5848590	2664	1.1917	1.38E-87
CNVR_1675	chrAC_000184.1	6026635	6028854	2220	1.1863	8.72E-73
CNVR_1676	chrAC_000184.1	12357187	12358962	1776	1.5788	1.95E-88
CNVR_1677	chrAC_000184.1	14139847	14142066	2220	2.5517	1.08E-185
CNVR_1678	chrAC_000184.1	14144287	14146506	2220	1.4498	5.91E-98
CNVR_1679	chrAC_000184.1	28929931	28931706	1776	1.2964	5.08E-67
CNVR_1680	chrAC_000184.1	34654423	34656198	1776	1.2785	1.20E-65
CNVR_1681	chrAC_000184.1	35840347	35842122	1776	1.7746	1.33E-102
CNVR_1682	chrAC_000184.1	37578607	37580826	2220	1.1326	1.07E-67
CNVR_1683	chrAC_000184.1	38808931	38814702	5772	2.0121	0.00E+00
CNVR_1684	chrAC_000184.1	38815591	38817810	2220	2.2826	4.47E-168
CNVR_1685	chrAC_000184.1	5424127	5425902	1776	-1.1994	6.80E-54
CNVR_1686	chrAC_000184.1	5426347	5429454	3108	-1.5728	2.83E-135
CNVR_1687	chrAC_000184.1	5467639	5469858	2220	-1.7728	1.90E-112
CNVR_1688	chrAC_000184.1	5574643	5579082	4440	-1.4958	3.45E-180
CNVR_1689	chrAC_000184.1	5579971	5583078	3108	-1.9252	6.27E-172
CNVR_1690	chrAC_000184.1	5587519	5596842	9324	-2.3004	0.00E+00
CNVR_1691	chrAC_000184.1	5603503	5605722	2220	-2.2391	9.49E-144
CNVR_1692	chrAC_000184.1	5607943	5610606	2664	-2.1164	5.68E-163
CNVR_1693	chrAC_000184.1	5611495	5616822	5328	-1.6741	3.12E-249
CNVR_1694	chrAC_000184.1	5617711	5620374	2664	-2.1248	1.30E-163
CNVR_1695	chrAC_000184.1	5623483	5625258	1776	-1.8240	2.41E-93
CNVR_1696	chrAC_000184.1	5628367	5638134	9768	-3.1913	0.00E+00
CNVR_1697	chrAC_000184.1	5643463	5655450	11988	-3.7487	0.00E+00
CNVR_1698	chrAC_000184.1	5655895	5659446	3552	-1.8848	1.18E-191
CNVR_1699	chrAC_000184.1	5898763	5900982	2220	-1.4879	1.90E-90
CNVR_1700	chrAC_000184.1	5906755	5908974	2220	-1.1550	2.93E-63
CNVR_1701	chrAC_000184.1	5914303	5916522	2220	-1.7281	4.08E-109
CNVR_1702	chrAC_000184.1	6159391	6161166	1776	-1.5802	1.36E-78
CNVR_1703	chrAC_000184.1	6214447	6216666	2220	-1.5939	7.81E-99
CNVR_1704	chrAC_000184.1	6459979	6464418	4440	-1.3088	7.37E-150
CNVR_1705	chrAC_000184.1	11618815	11620590	1776	-1.3271	2.70E-62
CNVR_1706	chrAC_000184.1	12983227	12985002	1776	-2.0134	6.39E-104
CNVR_1707	chrAC_000184.1	13794415	13796190	1776	-1.2971	2.54E-60
CNVR_1708	chrAC_000184.1	13872559	13875666	3108	-1.5001	3.24E-127
CNVR_1709	chrAC_000184.1	14177587	14179362	1776	-1.3344	8.99E-63
CNVR_1710	chrAC_000184.1	15831487	15833262	1776	-1.2733	9.27E-59
CNVR_1711	chrAC_000184.1	18925723	18928386	2664	-4.0980	2.52E-253
CNVR_1712	chrAC_000184.1	19549099	19558422	9324	-4.3353	0.00E+00

CNVR_1713	chrAC_000184.1	22531003	22539438	8436	-2.7217	0.00E+00
CNVR_1714	chrAC_000184.1	34521667	34523442	1776	-4.7523	1.50E-177
CNVR_1715	chrAC_000184.1	39419875	39422094	2220	-3.2554	3.80E-190
CNVR_1716	chrAC_000184.1	39464275	39467382	3108	-1.3046	5.14E-105
CNVR_1717	chrAC_000184.1	39673843	39675618	1776	-2.3986	9.35E-123
CNVR_1718	chrAC_000184.1	44302543	44304318	1776	-1.2685	1.92E-58
CNVR_1719	chrAC_000160.1	11783539	11785314	1776	1.6600	1.87E-38
CNVR_1720	chrAC_000160.1	11785759	11788422	2664	1.2806	1.18E-39
CNVR_1721	chrAC_000160.1	11893207	11894982	1776	1.1683	1.44E-23
CNVR_1722	chrAC_000160.1	11897203	11903862	6660	2.1552	1.06E-188
CNVR_1723	chrAC_000160.1	13289587	13291806	2220	1.3994	7.07E-38
CNVR_1724	chrAC_000160.1	13295359	13299798	4440	1.7264	3.37E-98
CNVR_1725	chrAC_000160.1	13920067	13923174	3108	1.2016	9.21E-42
CNVR_1726	chrAC_000160.1	54354703	54356478	1776	1.1356	1.40E-22
CNVR_1727	chrAC_000160.1	54438619	54440838	2220	1.2919	9.11E-34
CNVR_1728	chrAC_000160.1	105712627	105714402	1776	1.3843	3.53E-30
CNVR_1729	chrAC_000160.1	105717067	105719286	2220	1.2318	1.81E-31
CNVR_1730	chrAC_000160.1	105719731	105721506	1776	1.3267	2.05E-28
CNVR_1731	chrAC_000160.1	105723727	105725502	1776	1.1715	1.15E-23
CNVR_1732	chrAC_000160.1	115329667	115331886	2220	1.2949	7.02E-34
CNVR_1733	chrAC_000160.1	1844155	1845930	1776	-2.0392	7.44E-41
CNVR_1734	chrAC_000160.1	2430679	2433342	2664	-1.1120	3.80E-28
CNVR_1735	chrAC_000160.1	8517475	8538786	21312	-3.8618	0.00E+00
CNVR_1736	chrAC_000160.1	8616931	8665326	48396	-3.7005	0.00E+00
CNVR_1737	chrAC_000160.1	8665771	8668878	3108	-2.5228	4.31E-85
CNVR_1738	chrAC_000160.1	8677759	8679978	2220	-1.2787	6.61E-29
CNVR_1739	chrAC_000160.1	8731483	8734590	3108	-2.8438	5.16E-93
CNVR_1740	chrAC_000160.1	8735035	8747910	12876	-5.1487	0.00E+00
CNVR_1741	chrAC_000160.1	8748799	8751906	3108	-2.3823	3.89E-81
CNVR_1742	chrAC_000160.1	11855911	11857686	1776	-1.7149	6.25E-34
CNVR_1743	chrAC_000160.1	11873227	11877222	3996	-1.5996	3.73E-68
CNVR_1744	chrAC_000160.1	11878111	11879886	1776	-1.4633	5.28E-28
CNVR_1745	chrAC_000160.1	11880775	11886102	5328	-2.0706	9.39E-121
CNVR_1746	chrAC_000160.1	13314007	13316226	2220	-2.5886	1.46E-62
CNVR_1747	chrAC_000160.1	13316671	13318446	1776	-2.7022	4.74E-52
CNVR_1748	chrAC_000160.1	15379495	15381714	2220	-1.2226	3.68E-27
CNVR_1749	chrAC_000160.1	17539999	17542218	2220	-1.2278	2.52E-27
CNVR_1750	chrAC_000160.1	18478615	18480834	2220	-1.3447	5.97E-31
CNVR_1751	chrAC_000160.1	21246067	21248286	2220	-1.7433	8.73E-43
CNVR_1752	chrAC_000160.1	21302011	21303786	1776	-4.0901	5.75E-65
CNVR_1753	chrAC_000160.1	21321991	21324210	2220	-1.5588	1.88E-37
CNVR_1754	chrAC_000160.1	22409791	22411566	1776	-4.6520	1.24E-67
CNVR_1755	chrAC_000160.1	22626907	22634454	7548	-5.3818	1.62E-290
CNVR_1756	chrAC_000160.1	22774315	22776090	1776	-4.3834	1.75E-66

CNVR_1757	chrAC_000160.1	36570727	36572502	1776	-1.4415	1.80E-27
CNVR_1758	chrAC_000160.1	38309875	38313426	3552	-5.7782	1.89E-139
CNVR_1759	chrAC_000160.1	40526323	40528098	1776	-1.3735	8.38E-26
CNVR_1760	chrAC_000160.1	43547299	43549074	1776	-1.1931	2.56E-21
CNVR_1761	chrAC_000160.1	46052347	46054122	1776	-1.3027	4.77E-24
CNVR_1762	chrAC_000160.1	49241599	49246926	5328	-3.2235	5.11E-171
CNVR_1763	chrAC_000160.1	49254475	49256694	2220	-2.0361	1.49E-50
CNVR_1764	chrAC_000160.1	49258027	49264686	6660	-3.1112	7.51E-209
CNVR_1765	chrAC_000160.1	54513655	54515874	2220	-3.0197	1.20E-69
CNVR_1766	chrAC_000160.1	54518539	54523866	5328	-1.5500	9.72E-87
CNVR_1767	chrAC_000160.1	54589579	54591354	1776	-1.4926	1.03E-28
CNVR_1768	chrAC_000160.1	54592243	54594906	2664	-1.7200	3.36E-50
CNVR_1769	chrAC_000160.1	54602455	54605118	2664	-1.5598	1.25E-44
CNVR_1770	chrAC_000160.1	54613999	54619770	5772	-1.5445	2.20E-93
CNVR_1771	chrAC_000160.1	54620659	54622434	1776	-1.7731	3.07E-35
CNVR_1772	chrAC_000160.1	54626875	54630426	3552	-1.3225	2.16E-47
CNVR_1773	chrAC_000160.1	54632647	54636198	3552	-1.8712	5.58E-73
CNVR_1774	chrAC_000160.1	54638863	54641082	2220	-1.2160	5.87E-27
CNVR_1775	chrAC_000160.1	54645523	54647298	1776	-1.3773	6.75E-26
CNVR_1776	chrAC_000160.1	54652627	54656178	3552	-1.2551	4.68E-44
CNVR_1777	chrAC_000160.1	54661507	54664170	2664	-1.2014	1.81E-31
CNVR_1778	chrAC_000160.1	54668167	54669942	1776	-1.2828	1.49E-23
CNVR_1779	chrAC_000160.1	54675271	54677046	1776	-1.2283	3.39E-22
CNVR_1780	chrAC_000160.1	54679267	54681042	1776	-1.2028	1.47E-21
CNVR_1781	chrAC_000160.1	54692587	54694362	1776	-1.0928	7.96E-19
CNVR_1782	chrAC_000160.1	54695251	54697914	2664	-1.4911	3.64E-42
CNVR_1783	chrAC_000160.1	54739207	54740982	1776	-1.2828	1.49E-23
CNVR_1784	chrAC_000160.1	54760519	54762738	2220	-1.6184	3.32E-39
CNVR_1785	chrAC_000160.1	54830227	54832002	1776	-1.1699	9.70E-21
CNVR_1786	chrAC_000160.1	54832891	54835554	2664	-1.2386	7.38E-33
CNVR_1787	chrAC_000160.1	54854647	54856866	2220	-1.4277	1.68E-33
CNVR_1788	chrAC_000160.1	54863971	54884838	20868	-4.1236	0.00E+00
CNVR_1789	chrAC_000160.1	54897271	54899046	1776	-1.4494	1.15E-27
CNVR_1790	chrAC_000160.1	54899935	54901710	1776	-1.2642	4.33E-23
CNVR_1791	chrAC_000160.1	54938119	54941670	3552	-1.2226	1.91E-42
CNVR_1792	chrAC_000160.1	54944779	54946998	2220	-1.1783	8.73E-26
CNVR_1793	chrAC_000160.1	54949219	54951438	2220	-1.1826	6.44E-26
CNVR_1794	chrAC_000160.1	54958099	54959874	1776	-1.1870	3.62E-21
CNVR_1795	chrAC_000160.1	55852315	55856310	3996	-1.6039	2.22E-68
CNVR_1796	chrAC_000160.1	56849095	56852646	3552	-1.8520	3.71E-72
CNVR_1797	chrAC_000160.1	58491451	58493670	2220	-2.4389	1.19E-59
CNVR_1798	chrAC_000160.1	58498555	58500330	1776	-1.2419	1.55E-22
CNVR_1799	chrAC_000160.1	62382223	62385774	3552	-1.8950	5.43E-74
CNVR_1800	chrAC_000160.1	62704567	62706786	2220	-1.1806	7.43E-26

CNVR_1801	chrAC_000160.1	63137467	63139242	1776	-2.4400	4.26E-48
CNVR_1802	chrAC_000160.1	63379891	63382110	2220	-2.8171	1.52E-66
CNVR_1803	chrAC_000160.1	64001935	64003710	1776	-1.1510	2.87E-20
CNVR_1804	chrAC_000160.1	67780819	67783926	3108	-1.5651	5.42E-52
CNVR_1805	chrAC_000160.1	68159995	68162214	2220	-4.6878	3.04E-84
CNVR_1806	chrAC_000160.1	68320723	68323830	3108	-1.4702	5.11E-48
CNVR_1807	chrAC_000160.1	68711887	68714550	2664	-2.1225	7.88E-63
CNVR_1808	chrAC_000160.1	70599331	70601550	2220	-1.6210	2.78E-39
CNVR_1809	chrAC_000160.1	70677475	70679250	1776	-1.4766	2.51E-28
CNVR_1810	chrAC_000160.1	70738747	70740966	2220	-1.2460	6.89E-28
CNVR_1811	chrAC_000160.1	72211495	72213270	1776	-1.3265	1.22E-24
CNVR_1812	chrAC_000160.1	72233695	72235470	1776	-1.5480	4.83E-30
CNVR_1813	chrAC_000160.1	75366559	75368334	1776	-1.2828	1.49E-23
CNVR_1814	chrAC_000160.1	76524067	76526286	2220	-1.5983	1.29E-38
CNVR_1815	chrAC_000160.1	80124907	80128902	3996	-2.3499	1.53E-102
CNVR_1816	chrAC_000160.1	81537271	81540378	3108	-2.2029	1.25E-75
CNVR_1817	chrAC_000160.1	86749831	86751606	1776	-1.2113	9.01E-22
CNVR_1818	chrAC_000160.1	97720183	97722402	2220	-2.8396	6.57E-67
CNVR_1819	chrAC_000160.1	97722847	97724622	1776	-2.2607	4.84E-45
CNVR_1820	chrAC_000160.1	98503843	98505618	1776	-1.2708	2.95E-23
CNVR_1821	chrAC_000160.1	103613839	103616946	3108	-2.2622	1.65E-77
CNVR_1822	chrAC_000160.1	104131987	104133762	1776	-1.5683	1.59E-30
CNVR_1823	chrAC_000160.1	104402827	104404602	1776	-1.9502	4.78E-39
CNVR_1824	chrAC_000160.1	111997891	111999666	1776	-1.6097	1.67E-31
CNVR_1825	chrAC_000160.1	113900431	113902206	1776	-1.3056	4.03E-24
CNVR_1826	chrAC_000160.1	113918635	113923518	4884	-4.2729	5.72E-178
CNVR_1827	chrAC_000160.1	116438779	116440554	1776	-2.3890	2.95E-47
CNVR_1828	chrAC_000160.1	120224767	120227430	2664	-1.9128	1.62E-56
CNVR_1829	chrAC_000182.1	21629239	21632346	3108	1.6897	1.67E-72
CNVR_1830	chrAC_000182.1	30350287	30352062	1776	1.2331	2.01E-27
CNVR_1831	chrAC_000182.1	2497723	2499498	1776	-1.2621	4.66E-25
CNVR_1832	chrAC_000182.1	2500387	2502162	1776	-1.3208	1.17E-26
CNVR_1833	chrAC_000182.1	3862135	3863910	1776	-1.9035	1.50E-41
CNVR_1834	chrAC_000182.1	17947147	17949810	2664	-3.2348	7.11E-95
CNVR_1835	chrAC_000182.1	21224311	21226974	2664	-3.0832	3.02E-92
CNVR_1836	chrAC_000182.1	30379591	30381810	2220	-2.0099	1.63E-54
CNVR_1837	chrAC_000182.1	32366491	32369154	2664	-1.3694	4.08E-41
CNVR_1838	chrAC_000182.1	35932699	35940246	7548	-5.0656	0.00E+00
CNVR_1839	chrAC_000182.1	36106303	36108078	1776	-3.4612	3.39E-66
CNVR_1840	chrAC_000182.1	42023109	42025773	2664	2.5084	1.36E-45
CNVR_1841	chrAC_000161.1	30119851	30121626	1776	1.1118	1.62E-23
CNVR_1842	chrAC_000161.1	76849519	76851294	1776	1.6352	9.48E-41
CNVR_1843	chrAC_000161.1	81706879	81708654	1776	1.4746	1.55E-35
CNVR_1844	chrAC_000161.1	86733847	86739618	5772	1.7618	3.69E-141

CNVR_1845	chrAC_000161.1	86740063	86745834	5772	1.4859	1.27E-112
CNVR_1846	chrAC_000161.1	86750275	86752938	2664	1.8756	2.49E-71
CNVR_1847	chrAC_000161.1	93814759	93816534	1776	1.9755	4.66E-51
CNVR_1848	chrAC_000161.1	98740495	98742270	1776	1.3035	7.39E-30
CNVR_1849	chrAC_000161.1	98755591	98757366	1776	2.0084	5.49E-52
CNVR_1850	chrAC_000161.1	106414591	106416366	1776	1.1536	6.91E-25
CNVR_1851	chrAC_000161.1	106442119	106444338	2220	1.1736	1.62E-31
CNVR_1852	chrAC_000161.1	106451443	106453218	1776	1.1380	2.24E-24
CNVR_1853	chrAC_000161.1	106501171	106502946	1776	1.2417	8.42E-28
CNVR_1854	chrAC_000161.1	106512715	106519374	6660	1.4654	4.03E-127
CNVR_1855	chrAC_000161.1	119715499	119717274	1776	1.1793	9.87E-26
CNVR_1856	chrAC_000161.1	477967	480186	2220	-1.3629	1.72E-33
CNVR_1857	chrAC_000161.1	512155	513930	1776	-1.6123	1.59E-33
CNVR_1858	chrAC_000161.1	1896103	1897878	1776	-2.4712	1.28E-51
CNVR_1859	chrAC_000161.1	2147407	2149182	1776	-1.1528	1.43E-21
CNVR_1860	chrAC_000161.1	2289487	2292594	3108	-1.9311	1.70E-70
CNVR_1861	chrAC_000161.1	2295259	2297478	2220	-1.4748	3.99E-37
CNVR_1862	chrAC_000161.1	2531911	2533686	1776	-1.5789	1.09E-32
CNVR_1863	chrAC_000161.1	2888443	2891550	3108	-1.8394	7.11E-67
CNVR_1864	chrAC_000161.1	2935507	2937282	1776	-1.3519	7.74E-27
CNVR_1865	chrAC_000161.1	3002995	3004770	1776	-1.6439	2.61E-34
CNVR_1866	chrAC_000161.1	3018535	3021198	2664	-5.0424	6.22E-109
CNVR_1867	chrAC_000161.1	3167275	3169050	1776	-1.3304	2.85E-26
CNVR_1868	chrAC_000161.1	3365299	3367074	1776	-1.6783	3.74E-35
CNVR_1869	chrAC_000161.1	4095679	4097898	2220	-2.0798	7.30E-55
CNVR_1870	chrAC_000161.1	7148623	7150398	1776	-1.3542	6.71E-27
CNVR_1871	chrAC_000161.1	8566315	8568534	2220	-2.4379	2.33E-63
CNVR_1872	chrAC_000161.1	11788867	11790642	1776	-1.3358	2.05E-26
CNVR_1873	chrAC_000161.1	14876443	14879994	3552	-3.7205	5.14E-131
CNVR_1874	chrAC_000161.1	15864343	15866118	1776	-1.5435	8.52E-32
CNVR_1875	chrAC_000161.1	16071691	16075242	3552	-4.0379	1.90E-135
CNVR_1876	chrAC_000161.1	16715047	16716822	1776	-1.6818	3.08E-35
CNVR_1877	chrAC_000161.1	16721707	16723482	1776	-1.4025	3.66E-28
CNVR_1878	chrAC_000161.1	18420895	18422670	1776	-2.2440	1.60E-47
CNVR_1879	chrAC_000161.1	27756439	27758214	1776	-1.3609	4.49E-27
CNVR_1880	chrAC_000161.1	28199995	28203546	3552	-1.5689	3.97E-63
CNVR_1881	chrAC_000161.1	29830363	29832138	1776	-1.4123	2.03E-28
CNVR_1882	chrAC_000161.1	30158479	30160698	2220	-2.5195	4.45E-65
CNVR_1883	chrAC_000161.1	31089103	31090878	1776	-1.6431	2.72E-34
CNVR_1884	chrAC_000161.1	31889191	31894962	5772	-2.8980	2.95E-184
CNVR_1885	chrAC_000161.1	32071231	32073006	1776	-1.8650	1.45E-39
CNVR_1886	chrAC_000161.1	32956123	32958342	2220	-1.8449	1.91E-48
CNVR_1887	chrAC_000161.1	35036707	35038482	1776	-1.4547	1.61E-29
CNVR_1888	chrAC_000161.1	36917047	36918822	1776	-1.2396	7.15E-24

CNVR_1889	chrAC_000161.1	38859547	38861322	1776	-1.3201	5.32E-26
CNVR_1890	chrAC_000161.1	42057679	42059898	2220	-2.9590	4.72E-73
CNVR_1891	chrAC_000161.1	48548959	48550734	1776	-1.2326	1.10E-23
CNVR_1892	chrAC_000161.1	53527087	53530194	3108	-1.5743	1.07E-55
CNVR_1893	chrAC_000161.1	54830227	54832890	2664	-1.4916	7.49E-45
CNVR_1894	chrAC_000161.1	59802139	59803914	1776	-1.5849	7.66E-33
CNVR_1895	chrAC_000161.1	61191859	61196742	4884	-2.6148	4.58E-145
CNVR_1896	chrAC_000161.1	64913911	64917906	3996	-4.4430	3.99E-157
CNVR_1897	chrAC_000161.1	69143011	69145230	2220	-1.2524	7.52E-30
CNVR_1898	chrAC_000161.1	70673035	70675698	2664	-2.2907	1.07E-71
CNVR_1899	chrAC_000161.1	73443151	73445370	2220	-1.6416	2.19E-42
CNVR_1900	chrAC_000161.1	75850075	75855402	5328	-2.0806	4.39E-129
CNVR_1901	chrAC_000161.1	76768267	76770042	1776	-2.3953	2.63E-50
CNVR_1902	chrAC_000161.1	76771375	76773150	1776	-1.5061	7.63E-31
CNVR_1903	chrAC_000161.1	80809555	80811774	2220	-1.9893	1.81E-52
CNVR_1904	chrAC_000161.1	83581447	83584998	3552	-1.5030	8.67E-60
CNVR_1905	chrAC_000161.1	84152431	84155094	2664	-2.6289	2.43E-80
CNVR_1906	chrAC_000161.1	84322927	84325146	2220	-1.1603	8.33E-27
CNVR_1907	chrAC_000161.1	84387751	84389970	2220	-1.5908	8.30E-41
CNVR_1908	chrAC_000161.1	84394411	84396186	1776	-1.5830	8.56E-33
CNVR_1909	chrAC_000161.1	84528055	84529830	1776	-2.0954	1.43E-44
CNVR_1910	chrAC_000161.1	84570235	84572010	1776	-1.2144	3.34E-23
CNVR_1911	chrAC_000161.1	84895687	84898350	2664	-3.4812	9.75E-96
CNVR_1912	chrAC_000161.1	85278859	85280634	1776	-1.1369	3.77E-21
CNVR_1913	chrAC_000161.1	86745835	86747610	1776	-2.2867	2.48E-48
CNVR_1914	chrAC_000161.1	87133003	87135666	2664	-1.2643	5.88E-36
CNVR_1915	chrAC_000161.1	87410059	87411834	1776	-1.3797	1.44E-27
CNVR_1916	chrAC_000161.1	87886915	87888690	1776	-1.8711	1.05E-39
CNVR_1917	chrAC_000161.1	90785791	90788010	2220	-1.3230	3.52E-32
CNVR_1918	chrAC_000161.1	90907891	90909666	1776	-3.5254	7.57E-65
CNVR_1919	chrAC_000161.1	91425151	91426926	1776	-2.1211	4.27E-45
CNVR_1920	chrAC_000161.1	106011439	106014990	3552	-2.6563	2.54E-107
CNVR_1921	chrAC_000161.1	108242539	108265182	22644	-3.4964	0.00E+00
CNVR_1922	chrAC_000161.1	108266515	108270510	3996	-2.0592	2.65E-96
CNVR_1923	chrAC_000161.1	108287383	108289158	1776	-1.5079	6.86E-31
CNVR_1924	chrAC_000161.1	108683875	108686094	2220	-3.1986	1.41E-76
CNVR_1925	chrAC_000161.1	109511491	109514598	3108	-1.9198	4.67E-70
CNVR_1926	chrAC_000161.1	109613611	109615386	1776	-2.2829	2.94E-48
CNVR_1927	chrAC_000161.1	109799203	109800978	1776	-1.3074	1.15E-25
CNVR_1928	chrAC_000161.1	109813855	109816074	2220	-1.4791	2.90E-37
CNVR_1929	chrAC_000161.1	110104675	110107338	2664	-1.8801	5.63E-59
CNVR_1930	chrAC_000161.1	110493175	110494950	1776	-1.4157	1.66E-28
CNVR_1931	chrAC_000161.1	110555335	110557110	1776	-1.1979	9.14E-23
CNVR_1932	chrAC_000161.1	113170495	113172270	1776	-2.1174	5.08E-45

CNVR_1933	chrAC_000161.1	116165719	116168826	3108	-2.3779	4.07E-86
CNVR_1934	chrAC_000161.1	118264951	118267170	2220	-1.7199	9.08E-45
CNVR_1935	chrAC_000161.1	119883775	119886882	3108	-1.3357	5.99E-45
CNVR_1936	chrAC_000161.1	120646123	120652338	6216	-2.2427	1.51E-161
CNVR_1937	chrAC_000161.1	120653671	120673650	19980	-2.3587	0.00E+00
CNVR_1938	chrAC_000161.1	120676315	120680310	3996	-1.6238	7.27E-74
CNVR_1939	chrAC_000161.1	120686083	120698958	12876	-2.2708	0.00E+00
CNVR_1940	chrAC_000161.1	120699403	120701622	2220	-2.1052	1.62E-55
CNVR_1941	chrAC_000161.1	120704287	120706062	1776	-1.6521	1.64E-34
CNVR_1942	chrAC_000161.1	120706951	120710058	3108	-1.2892	8.28E-43
CNVR_1943	chrAC_000161.1	120710947	120713610	2664	-1.2839	9.90E-37
CNVR_1944	chrAC_000161.1	120769999	120771774	1776	-1.2637	1.65E-24
CNVR_1945	chrAC_000161.1	120808627	120811290	2664	-1.3751	2.52E-40
CNVR_1946	chrAC_000173.1	25087	27306	2220	1.8892	7.87E-53
CNVR_1947	chrAC_000173.1	148963	171606	22644	1.5942	0.00E+00
CNVR_1948	chrAC_000173.1	36507235	36509010	1776	1.1237	4.66E-21
CNVR_1949	chrAC_000173.1	70371559	70373778	2220	1.4542	7.10E-38
CNVR_1950	chrAC_000173.1	71748403	71750178	1776	2.3721	8.18E-54
CNVR_1951	chrAC_000173.1	7771	9990	2220	-1.5282	6.42E-34
CNVR_1952	chrAC_000173.1	12211	13986	1776	-1.2356	7.76E-21
CNVR_1953	chrAC_000173.1	42847	44622	1776	-1.1910	8.24E-20
CNVR_1954	chrAC_000173.1	375847	377622	1776	-1.1322	1.85E-18
CNVR_1955	chrAC_000173.1	5560879	5562654	1776	-3.7720	3.27E-58
CNVR_1956	chrAC_000173.1	5742031	5744250	2220	-1.6237	1.61E-36
CNVR_1957	chrAC_000173.1	5746027	5748246	2220	-1.4562	6.44E-32
CNVR_1958	chrAC_000173.1	5787319	5789094	1776	-1.2664	1.52E-21
CNVR_1959	chrAC_000173.1	10392487	10396482	3996	-2.0201	3.60E-82
CNVR_1960	chrAC_000173.1	10401367	10404918	3552	-2.2676	7.27E-82
CNVR_1961	chrAC_000173.1	15073135	15074910	1776	-1.3022	2.29E-22
CNVR_1962	chrAC_000173.1	15082459	15086010	3552	-1.5767	2.40E-55
CNVR_1963	chrAC_000173.1	15087343	15089562	2220	-1.3711	1.62E-29
CNVR_1964	chrAC_000173.1	15096667	15099330	2664	-1.6638	9.24E-45
CNVR_1965	chrAC_000173.1	15113095	15114870	1776	-1.3059	1.88E-22
CNVR_1966	chrAC_000173.1	15117535	15119310	1776	-1.2466	4.33E-21
CNVR_1967	chrAC_000173.1	15959359	15961134	1776	-1.3042	2.06E-22
CNVR_1968	chrAC_000173.1	17396587	17398806	2220	-2.8071	2.10E-61
CNVR_1969	chrAC_000173.1	17400139	17402802	2664	-3.3727	6.13E-82
CNVR_1970	chrAC_000173.1	18049711	18051486	1776	-2.1635	4.40E-40
CNVR_1971	chrAC_000173.1	21542659	21544434	1776	-1.1016	9.35E-18
CNVR_1972	chrAC_000173.1	38531875	38533650	1776	-1.2292	1.09E-20
CNVR_1973	chrAC_000173.1	45317527	45319746	2220	-2.6392	9.63E-59
CNVR_1974	chrAC_000173.1	46334731	46336506	1776	-2.6803	6.78E-48
CNVR_1975	chrAC_000173.1	50617999	50619774	1776	-1.4963	9.19E-27
CNVR_1976	chrAC_000173.1	55854091	55858974	4884	-2.6763	2.64E-128

CNVR_1977	chrAC_000173.1	55916251	55919358	3108	-2.8522	3.68E-86
CNVR_1978	chrAC_000173.1	65672707	65674482	1776	-1.3211	8.45E-23
CNVR_1979	chrAC_000173.1	69037339	69039114	1776	-1.1935	7.22E-20
CNVR_1980	chrAC_000173.1	70373779	70375554	1776	-2.9493	3.96E-51
CNVR_1981	chrAC_000173.1	70503427	70505646	2220	-1.2034	1.02E-24
CNVR_1982	chrAC_000173.1	71743075	71745294	2220	-1.5484	1.79E-34
CNVR_1983	chrAC_000176.1	3744919	3747582	2664	1.2843	3.01E-39
CNVR_1984	chrAC_000176.1	14612263	14614038	1776	1.9148	5.75E-45
CNVR_1985	chrAC_000176.1	28487707	28489482	1776	2.1047	8.60E-50
CNVR_1986	chrAC_000176.1	51134371	51136590	2220	1.3831	1.02E-36
CNVR_1987	chrAC_000176.1	2710843	2712618	1776	-1.1963	5.77E-21
CNVR_1988	chrAC_000176.1	2718835	2721054	2220	-1.2441	2.94E-27
CNVR_1989	chrAC_000176.1	3969583	3971802	2220	-1.4251	1.02E-32
CNVR_1990	chrAC_000176.1	4545007	4547226	2220	-1.3388	3.97E-30
CNVR_1991	chrAC_000176.1	4562767	4564542	1776	-1.3083	1.08E-23
CNVR_1992	chrAC_000176.1	4577419	4579638	2220	-1.7337	1.34E-41
CNVR_1993	chrAC_000176.1	5950711	5952930	2220	-1.4985	6.80E-35
CNVR_1994	chrAC_000176.1	6273055	6275718	2664	-1.7078	1.04E-48
CNVR_1995	chrAC_000176.1	7422571	7426566	3996	-2.5628	9.47E-108
CNVR_1996	chrAC_000176.1	14502151	14511474	9324	-1.7781	7.81E-174
CNVR_1997	chrAC_000176.1	14536783	14539446	2664	-1.3854	1.71E-37
CNVR_1998	chrAC_000176.1	15744907	15748014	3108	-1.8329	2.83E-61
CNVR_1999	chrAC_000176.1	19149055	19151718	2664	-2.0779	3.50E-60
CNVR_2000	chrAC_000176.1	19152163	19153938	1776	-1.3053	1.28E-23
CNVR_2001	chrAC_000176.1	19796851	19798626	1776	-1.4098	3.85E-26
CNVR_2002	chrAC_000176.1	19799515	19803510	3996	-1.2481	6.44E-48
CNVR_2003	chrAC_000176.1	19808395	19810614	2220	-1.4170	1.78E-32
CNVR_2004	chrAC_000176.1	19811059	19812834	1776	-1.1838	1.16E-20
CNVR_2005	chrAC_000176.1	19814167	19815942	1776	-1.1098	7.30E-19
CNVR_2006	chrAC_000176.1	19827487	19841250	13764	-1.5937	7.89E-224
CNVR_2007	chrAC_000176.1	19843915	19845690	1776	-1.2396	5.07E-22
CNVR_2008	chrAC_000176.1	19848799	19851018	2220	-1.3747	3.30E-31
CNVR_2009	chrAC_000176.1	19862119	19865670	3552	-1.4244	2.82E-51
CNVR_2010	chrAC_000176.1	19876771	19878546	1776	-1.6675	3.78E-32
CNVR_2011	chrAC_000176.1	19904743	19907406	2664	-1.4875	3.87E-41
CNVR_2012	chrAC_000176.1	19914955	19920726	5772	-1.6987	1.01E-102
CNVR_2013	chrAC_000176.1	19922503	19925610	3108	-2.1098	5.81E-71
CNVR_2014	chrAC_000176.1	19927831	19953138	25308	-2.6544	0.00E+00
CNVR_2015	chrAC_000176.1	20330095	20335866	5772	-1.6955	1.71E-102
CNVR_2016	chrAC_000176.1	20336311	20362062	25752	-1.7118	0.00E+00
CNVR_2017	chrAC_000176.1	21704275	21707382	3108	-1.6544	1.94E-54
CNVR_2018	chrAC_000176.1	24550315	24552090	1776	-1.3478	1.20E-24
CNVR_2019	chrAC_000176.1	24767431	24769650	2220	-1.4730	3.86E-34
CNVR_2020	chrAC_000176.1	24772759	24781194	8436	-1.5999	1.17E-138

CNVR_2021	chrAC_000176.1	28874431	28876206	1776	-3.8871	2.68E-62
CNVR_2022	chrAC_000176.1	29535991	29538654	2664	-3.7674	2.55E-91
CNVR_2023	chrAC_000176.1	30241781	30246665	4884	-1.8429	3.72E-23
CNVR_2024	chrAC_000176.1	43972651	43974426	1776	-1.4423	6.44E-27
CNVR_2025	chrAC_000176.1	45019159	45020934	1776	-1.1799	1.44E-20
CNVR_2026	chrAC_000176.1	51080203	51082866	2664	-1.7578	2.33E-50
CNVR_2027	chrAC_000176.1	57555943	57560826	4884	-1.5350	5.64E-77
CNVR_2028	chrAC_000176.1	60292759	60294534	1776	-1.2370	5.88E-22
CNVR_2029	chrAC_000176.1	61413415	61415190	1776	-3.7713	1.57E-61
CNVR_2030	chrAC_000177.1	23805283	23807058	1776	1.4009	5.46E-27
CNVR_2031	chrAC_000177.1	31319983	31321758	1776	1.2088	7.88E-22
CNVR_2032	chrAC_000177.1	44927251	44930358	3108	1.9799	1.91E-71
CNVR_2033	chrAC_000177.1	44940571	44943678	3108	1.8484	4.86E-66
CNVR_2034	chrAC_000177.1	49547959	49552842	4884	3.8218	8.51E-184
CNVR_2035	chrAC_000177.1	66339151	66340926	1776	1.3810	1.87E-26
CNVR_2036	chrAC_000177.1	92575	96126	3552	-1.8034	3.81E-60
CNVR_2037	chrAC_000177.1	7339099	7340874	1776	-2.4212	4.50E-41
CNVR_2038	chrAC_000177.1	7342651	7344870	2220	-1.8231	1.03E-38
CNVR_2039	chrAC_000177.1	7830163	7831938	1776	-1.2006	1.21E-18
CNVR_2040	chrAC_000177.1	15669871	15672534	2664	-2.7697	2.46E-67
CNVR_2041	chrAC_000177.1	15943819	15945594	1776	-1.7714	2.44E-30
CNVR_2042	chrAC_000177.1	16091227	16094334	3108	-1.2640	9.10E-34
CNVR_2043	chrAC_000177.1	18307231	18309006	1776	-5.0694	3.37E-59
CNVR_2044	chrAC_000177.1	29692723	29694942	2220	-1.3914	5.86E-28
CNVR_2045	chrAC_000177.1	35553079	35554854	1776	-1.8742	2.97E-32
CNVR_2046	chrAC_000177.1	38265919	38268582	2664	-1.7266	2.27E-43
CNVR_2047	chrAC_000177.1	43403887	43406550	2664	-2.9760	1.18E-70
CNVR_2048	chrAC_000177.1	45918259	45920034	1776	-1.1523	1.29E-17
CNVR_2049	chrAC_000177.1	49036471	49038246	1776	-1.4354	1.30E-23
CNVR_2050	chrAC_000177.1	49529755	49531974	2220	-1.3471	8.58E-27
CNVR_2051	chrAC_000177.1	49880959	49882734	1776	-1.2348	2.26E-19
CNVR_2052	chrAC_000177.1	49894723	49896498	1776	-1.0798	4.47E-16
CNVR_2053	chrAC_000177.1	52653295	52658178	4884	-3.3425	5.65E-137
CNVR_2054	chrAC_000177.1	53785495	53787714	2220	-1.8113	1.94E-38
CNVR_2055	chrAC_000177.1	56702131	56706570	4440	-5.6026	6.37E-148
CNVR_2056	chrAC_000177.1	65285095	65286870	1776	-1.8166	3.44E-31
CNVR_2057	chrAC_000177.1	70956751	70958526	1776	-1.4782	1.67E-24
CNVR_2058	chrAC_000159.1	5875675	5878338	2664	1.8246	2.91E-57
CNVR_2059	chrAC_000159.1	38085655	38087430	1776	1.2119	2.40E-22
CNVR_2060	chrAC_000159.1	78343135	78345354	2220	1.5163	5.95E-38
CNVR_2061	chrAC_000159.1	89575891	89578110	2220	1.0897	2.17E-23
CNVR_2062	chrAC_000159.1	92069839	92072058	2220	1.7499	1.17E-45
CNVR_2063	chrAC_000159.1	110788435	110791542	3108	1.2972	5.91E-42
CNVR_2064	chrAC_000159.1	110791987	110795982	3996	1.1868	1.21E-46

CNVR_2065	chrAC_000159.1	110796871	110802642	5772	1.4170	5.02E-87
CNVR_2066	chrAC_000159.1	110803087	110806194	3108	1.3167	6.85E-43
CNVR_2067	chrAC_000159.1	135094771	135098322	3552	1.2896	2.83E-47
CNVR_2068	chrAC_000159.1	135099211	135108090	8880	1.4130	2.80E-132
CNVR_2069	chrAC_000159.1	135108535	135114306	5772	1.1983	1.37E-67
CNVR_2070	chrAC_000159.1	135114751	135117414	2664	1.2034	2.38E-32
CNVR_2071	chrAC_000159.1	135119191	135124518	5328	1.3468	1.03E-74
CNVR_2072	chrAC_000159.1	135125407	135129402	3996	1.2936	3.30E-53
CNVR_2073	chrAC_000159.1	135130291	135133398	3108	1.3489	1.95E-44
CNVR_2074	chrAC_000159.1	135133843	135137838	3996	1.3316	1.47E-55
CNVR_2075	chrAC_000159.1	135138283	135140946	2664	1.5076	7.64E-45
CNVR_2076	chrAC_000159.1	135142279	135153378	11100	1.2854	4.78E-143
CNVR_2077	chrAC_000159.1	135153823	135161814	7992	1.3248	2.10E-108
CNVR_2078	chrAC_000159.1	135163591	135170694	7104	1.3071	1.23E-94
CNVR_2079	chrAC_000159.1	135171583	135175578	3996	1.1671	1.93E-45
CNVR_2080	chrAC_000159.1	135178687	135180462	1776	1.4548	4.99E-29
CNVR_2081	chrAC_000159.1	135180907	135188454	7548	1.3836	2.48E-109
CNVR_2082	chrAC_000159.1	135188899	135191562	2664	1.2943	4.38E-36
CNVR_2083	chrAC_000159.1	135198223	135204882	6660	1.3018	2.72E-88
CNVR_2084	chrAC_000159.1	135205771	135207990	2220	1.1662	5.74E-26
CNVR_2085	chrAC_000159.1	135212431	135215094	2664	1.2053	1.99E-32
CNVR_2086	chrAC_000159.1	135216427	135218202	1776	1.2650	8.33E-24
CNVR_2087	chrAC_000159.1	135220867	135230190	9324	1.3833	1.51E-134
CNVR_2088	chrAC_000159.1	135231079	135237294	6216	1.3033	1.10E-82
CNVR_2089	chrAC_000159.1	135238183	135240846	2664	1.2173	6.40E-33
CNVR_2090	chrAC_000159.1	135241735	135246618	4884	1.3073	1.16E-65
CNVR_2091	chrAC_000159.1	135247063	135250614	3552	1.2355	2.58E-44
CNVR_2092	chrAC_000159.1	135251059	135253722	2664	1.4771	1.34E-43
CNVR_2093	chrAC_000159.1	135254167	135259050	4884	1.2180	5.97E-59
CNVR_2094	chrAC_000159.1	135260383	135262602	2220	1.4367	3.03E-35
CNVR_2095	chrAC_000159.1	135265267	135270150	4884	1.3052	1.65E-65
CNVR_2096	chrAC_000159.1	135272371	135275478	3108	1.2206	2.79E-38
CNVR_2097	chrAC_000159.1	135275923	135298566	22644	1.3646	0.00E+00
CNVR_2098	chrAC_000159.1	135299899	135304338	4440	1.3791	8.81E-65
CNVR_2099	chrAC_000159.1	135309223	135316326	7104	1.3020	4.47E-94
CNVR_2100	chrAC_000159.1	135317215	135333198	15984	1.3555	2.46E-222
CNVR_2101	chrAC_000159.1	135333643	135339858	6216	1.2131	4.67E-74
CNVR_2102	chrAC_000159.1	135340303	135347850	7548	1.2030	2.36E-88
CNVR_2103	chrAC_000159.1	135348295	135356286	7992	1.3010	1.80E-105
CNVR_2104	chrAC_000159.1	135357175	135363834	6660	1.3027	2.20E-88
CNVR_2105	chrAC_000159.1	135366943	135372270	5328	1.2850	1.25E-69
CNVR_2106	chrAC_000159.1	135372715	135377154	4440	1.2431	1.83E-55
CNVR_2107	chrAC_000159.1	135377599	135379374	1776	1.1504	1.14E-20
CNVR_2108	chrAC_000159.1	135379819	135389142	9324	1.3294	8.45E-127

CNVR_2109	chrAC_000159.1	135390475	135396246	5772	1.2971	2.34E-76
CNVR_2110	chrAC_000159.1	135397579	135400686	3108	1.2824	3.05E-41
CNVR_2111	chrAC_000159.1	135401575	135406014	4440	1.2331	8.72E-55
CNVR_2112	chrAC_000159.1	135406459	135410010	3552	1.3505	1.28E-50
CNVR_2113	chrAC_000159.1	135411787	135418446	6660	1.2904	4.01E-87
CNVR_2114	chrAC_000159.1	135420223	135428214	7992	1.3484	2.65E-111
CNVR_2115	chrAC_000159.1	135430435	135432654	2220	1.2586	3.96E-29
CNVR_2116	chrAC_000159.1	135433099	135437538	4440	1.2510	5.28E-56
CNVR_2117	chrAC_000159.1	135439315	135452190	12876	1.3303	1.62E-174
CNVR_2118	chrAC_000159.1	135453967	135457074	3108	1.3086	1.68E-42
CNVR_2119	chrAC_000159.1	135457519	135460626	3108	1.3426	3.91E-44
CNVR_2120	chrAC_000159.1	135461071	135462846	1776	1.2652	8.24E-24
CNVR_2121	chrAC_000159.1	135463291	135465510	2220	1.3610	1.20E-32
CNVR_2122	chrAC_000159.1	135465955	135467730	1776	1.2381	4.57E-23
CNVR_2123	chrAC_000159.1	135468175	135473946	5772	1.3007	1.11E-76
CNVR_2124	chrAC_000159.1	135474391	135478386	3996	1.4125	1.53E-60
CNVR_2125	chrAC_000159.1	135479719	135490374	10656	1.3286	1.72E-144
CNVR_2126	chrAC_000159.1	135490819	135498366	7548	1.2527	4.22E-94
CNVR_2127	chrAC_000159.1	135498811	135502362	3552	1.3165	9.34E-49
CNVR_2128	chrAC_000159.1	135508579	135511242	2664	1.3600	8.58E-39
CNVR_2129	chrAC_000159.1	135513019	135516570	3552	1.1807	2.50E-41
CNVR_2130	chrAC_000159.1	135517903	135524118	6216	1.2382	1.90E-76
CNVR_2131	chrAC_000159.1	135526339	135529890	3552	1.3188	7.05E-49
CNVR_2132	chrAC_000159.1	135532999	135536550	3552	1.2651	6.22E-46
CNVR_2133	chrAC_000159.1	135537883	135539658	1776	1.2334	6.16E-23
CNVR_2134	chrAC_000159.1	135540103	135544098	3996	1.2378	9.00E-50
CNVR_2135	chrAC_000159.1	135546763	135549870	3108	1.2439	2.15E-39
CNVR_2136	chrAC_000159.1	135550759	135552534	1776	1.1923	8.24E-22
CNVR_2137	chrAC_000159.1	135557419	135559638	2220	1.1606	8.90E-26
CNVR_2138	chrAC_000159.1	135560971	135564522	3552	1.3616	3.16E-51
CNVR_2139	chrAC_000159.1	135566299	135570294	3996	1.2200	1.11E-48
CNVR_2140	chrAC_000159.1	135571627	135574734	3108	1.2820	3.18E-41
CNVR_2141	chrAC_000159.1	135581395	135583614	2220	1.1750	2.87E-26
CNVR_2142	chrAC_000159.1	135586723	135589386	2664	1.1059	2.17E-28
CNVR_2143	chrAC_000159.1	135590719	135592494	1776	1.2701	6.04E-24
CNVR_2144	chrAC_000159.1	136980439	136982658	2220	1.3089	7.42E-31
CNVR_2145	chrAC_000159.1	136994647	136997310	2664	1.6950	2.60E-52
CNVR_2146	chrAC_000159.1	2071483	2073258	1776	-1.8621	2.92E-32
CNVR_2147	chrAC_000159.1	5969803	5972022	2220	-3.0616	1.25E-60
CNVR_2148	chrAC_000159.1	6919075	6920850	1776	-1.8055	3.30E-31
CNVR_2149	chrAC_000159.1	6926179	6929730	3552	-1.8124	6.28E-61
CNVR_2150	chrAC_000159.1	7248967	7250742	1776	-1.2778	1.88E-20
CNVR_2151	chrAC_000159.1	8579635	8581410	1776	-1.4884	6.64E-25
CNVR_2152	chrAC_000159.1	8654227	8656002	1776	-1.7185	1.53E-29

CNVR_2153	chrAC_000159.1	10023967	10026186	2220	-1.3444	6.14E-27
CNVR_2154	chrAC_000159.1	10279711	10286370	6660	-4.4799	2.84E-211
CNVR_2155	chrAC_000159.1	10295695	10297470	1776	-1.4380	7.46E-24
CNVR_2156	chrAC_000159.1	10648231	10650006	1776	-1.2830	1.45E-20
CNVR_2157	chrAC_000159.1	10814287	10816062	1776	-1.4045	3.79E-23
CNVR_2158	chrAC_000159.1	11205451	11213886	8436	-2.7399	8.54E-209
CNVR_2159	chrAC_000159.1	13103551	13105326	1776	-1.1712	3.63E-18
CNVR_2160	chrAC_000159.1	13816615	13818834	2220	-1.3192	2.87E-26
CNVR_2161	chrAC_000159.1	19494487	19496706	2220	-2.2025	3.55E-47
CNVR_2162	chrAC_000159.1	19501591	19503366	1776	-1.3846	9.98E-23
CNVR_2163	chrAC_000159.1	22875547	22877322	1776	-1.3418	8.10E-22
CNVR_2164	chrAC_000159.1	24124963	24127182	2220	-1.2825	2.74E-25
CNVR_2165	chrAC_000159.1	26165143	26166918	1776	-1.3926	6.77E-23
CNVR_2166	chrAC_000159.1	26837359	26839134	1776	-1.9681	3.57E-34
CNVR_2167	chrAC_000159.1	29336635	29340186	3552	-1.7702	2.46E-59
CNVR_2168	chrAC_000159.1	29611471	29613246	1776	-1.8779	1.49E-32
CNVR_2169	chrAC_000159.1	29850343	29852118	1776	-1.4338	9.16E-24
CNVR_2170	chrAC_000159.1	32099647	32101422	1776	-2.5344	6.83E-43
CNVR_2171	chrAC_000159.1	33865879	33868098	2220	-1.3435	6.49E-27
CNVR_2172	chrAC_000159.1	35027383	35030046	2664	-2.6563	9.08E-66
CNVR_2173	chrAC_000159.1	35621011	35626782	5772	-2.6232	6.22E-139
CNVR_2174	chrAC_000159.1	35633887	35637438	3552	-3.4890	4.72E-103
CNVR_2175	chrAC_000159.1	40789615	40794054	4440	-3.2322	3.07E-123
CNVR_2176	chrAC_000159.1	41604799	41607018	2220	-2.3168	2.32E-49
CNVR_2177	chrAC_000159.1	41690047	41693598	3552	-3.3730	2.68E-101
CNVR_2178	chrAC_000159.1	44053015	44058786	5772	-3.0116	5.34E-153
CNVR_2179	chrAC_000159.1	46320967	46323186	2220	-1.6253	3.30E-34
CNVR_2180	chrAC_000159.1	46456387	46458606	2220	-2.1468	4.61E-46
CNVR_2181	chrAC_000159.1	48348715	48350934	2220	-1.3344	1.14E-26
CNVR_2182	chrAC_000159.1	51237823	51239598	1776	-1.1194	4.68E-17
CNVR_2183	chrAC_000159.1	51422527	51424302	1776	-1.7990	4.38E-31
CNVR_2184	chrAC_000159.1	51889171	51890946	1776	-1.4715	1.49E-24
CNVR_2185	chrAC_000159.1	53438287	53440062	1776	-1.3633	2.82E-22
CNVR_2186	chrAC_000159.1	54109171	54115386	6216	-1.4474	7.32E-80
CNVR_2187	chrAC_000159.1	57007603	57009822	2220	-1.2706	5.70E-25
CNVR_2188	chrAC_000159.1	61063987	61066206	2220	-1.9907	8.79E-43
CNVR_2189	chrAC_000159.1	68405527	68407746	2220	-1.8727	3.81E-40
CNVR_2190	chrAC_000159.1	68408191	68413518	5328	-2.0906	3.30E-105
CNVR_2191	chrAC_000159.1	68457031	68458806	1776	-1.6080	2.37E-27
CNVR_2192	chrAC_000159.1	72655051	72657270	2220	-3.2461	1.02E-62
CNVR_2193	chrAC_000159.1	77982163	77983938	1776	-1.3552	4.19E-22
CNVR_2194	chrAC_000159.1	77987047	77989710	2664	-1.4870	1.76E-36
CNVR_2195	chrAC_000159.1	79763491	79767042	3552	-1.4508	1.48E-46
CNVR_2196	chrAC_000159.1	82045207	82046982	1776	-1.6711	1.30E-28

CNVR_2197	chrAC_000159.1	82051423	82053642	2220	-1.6300	2.53E-34
CNVR_2198	chrAC_000159.1	82213039	82215258	2220	-1.6627	3.89E-35
CNVR_2199	chrAC_000159.1	83362111	83363886	1776	-1.2082	5.85E-19
CNVR_2200	chrAC_000159.1	85830751	85832526	1776	-1.1716	3.56E-18
CNVR_2201	chrAC_000159.1	90111799	90114462	2664	-3.1485	1.33E-73
CNVR_2202	chrAC_000159.1	90774247	90776910	2664	-3.6647	1.40E-79
CNVR_2203	chrAC_000159.1	94054075	94056294	2220	-1.3071	6.03E-26
CNVR_2204	chrAC_000159.1	98499847	98506506	6660	-2.3921	1.98E-148
CNVR_2205	chrAC_000159.1	100192375	100194594	2220	-1.2579	1.24E-24
CNVR_2206	chrAC_000159.1	101943511	101945286	1776	-2.9316	1.45E-47
CNVR_2207	chrAC_000159.1	109084807	109086582	1776	-1.1263	3.32E-17
CNVR_2208	chrAC_000159.1	117157615	117162942	5328	-2.1640	8.47E-109
CNVR_2209	chrAC_000159.1	117392491	117394710	2220	-1.6051	1.06E-33
CNVR_2210	chrAC_000159.1	117398707	117400482	1776	-1.7199	1.44E-29
CNVR_2211	chrAC_000159.1	117586075	117587850	1776	-1.3633	2.82E-22
CNVR_2212	chrAC_000159.1	136786411	136788186	1776	-1.3298	1.45E-21
CNVR_2213	chrAC_000159.1	136796623	136798398	1776	-2.1163	1.06E-36
CNVR_2214	chrAC_000159.1	136805503	136808166	2664	-2.3522	1.18E-59
CNVR_2215	chrAC_000159.1	136915615	136917834	2220	-1.3869	4.63E-28
CNVR_2216	chrAC_000159.1	137006191	137007966	1776	-2.0996	2.00E-36
CNVR_2217	chrAC_000159.1	137008855	137012406	3552	-1.8893	9.12E-64
CNVR_2218	chrAC_000163.1	2337883	2339658	1776	1.5980	3.81E-36
CNVR_2219	chrAC_000163.1	7014535	7016754	2220	2.0980	1.02E-61
CNVR_2220	chrAC_000163.1	8912191	8915298	3108	2.3364	2.53E-95
CNVR_2221	chrAC_000163.1	9092455	9104442	11988	2.3469	0.00E+00
CNVR_2222	chrAC_000163.1	23137063	23140614	3552	1.1891	5.51E-46
CNVR_2223	chrAC_000163.1	45769075	45772182	3108	1.2337	8.39E-43
CNVR_2224	chrAC_000163.1	52068103	52070322	2220	1.2897	3.51E-33
CNVR_2225	chrAC_000163.1	79481551	79483326	1776	1.3082	1.92E-27
CNVR_2226	chrAC_000163.1	81672691	81674466	1776	2.1951	4.56E-52
CNVR_2227	chrAC_000163.1	81698887	81700662	1776	1.3556	6.99E-29
CNVR_2228	chrAC_000163.1	104246095	104247870	1776	1.1006	3.45E-21
CNVR_2229	chrAC_000163.1	20647	22866	2220	-1.4330	2.06E-32
CNVR_2230	chrAC_000163.1	103231	106338	3108	-1.4511	1.99E-45
CNVR_2231	chrAC_000163.1	1048063	1050726	2664	-3.0475	2.98E-80
CNVR_2232	chrAC_000163.1	1173715	1175490	1776	-1.4510	1.10E-26
CNVR_2233	chrAC_000163.1	6120319	6122094	1776	-1.4751	3.04E-27
CNVR_2234	chrAC_000163.1	9246967	9248742	1776	-1.5096	4.84E-28
CNVR_2235	chrAC_000163.1	9912079	9913854	1776	-1.3869	3.52E-25
CNVR_2236	chrAC_000163.1	10116763	10118982	2220	-1.4553	4.62E-33
CNVR_2237	chrAC_000163.1	11027851	11029626	1776	-1.2079	6.35E-21
CNVR_2238	chrAC_000163.1	11807959	11809734	1776	-1.2721	1.86E-22
CNVR_2239	chrAC_000163.1	12275047	12276822	1776	-1.2418	9.88E-22
CNVR_2240	chrAC_000163.1	14309899	14312118	2220	-2.9762	3.33E-66

CNVR_2241	chrAC_000163.1	19133959	19136622	2664	-1.2322	1.95E-31
CNVR_2242	chrAC_000163.1	20359399	20361618	2220	-1.4404	1.25E-32
CNVR_2243	chrAC_000163.1	23480719	23482494	1776	-2.2163	1.71E-42
CNVR_2244	chrAC_000163.1	23489155	23491374	2220	-1.6171	1.11E-37
CNVR_2245	chrAC_000163.1	24585835	24588942	3108	-1.9914	9.22E-66
CNVR_2246	chrAC_000163.1	26110975	26112750	1776	-1.1403	2.62E-19
CNVR_2247	chrAC_000163.1	26744119	26746338	2220	-1.1387	1.09E-23
CNVR_2248	chrAC_000163.1	28108087	28112082	3996	-3.9610	3.74E-136
CNVR_2249	chrAC_000163.1	28139611	28141386	1776	-1.4679	4.46E-27
CNVR_2250	chrAC_000163.1	33857887	33859662	1776	-1.2122	5.03E-21
CNVR_2251	chrAC_000163.1	33964447	33968442	3996	-2.3622	7.72E-99
CNVR_2252	chrAC_000163.1	35251603	35254266	2664	-1.5342	4.66E-42
CNVR_2253	chrAC_000163.1	38196211	38199762	3552	-2.9404	5.64E-104
CNVR_2254	chrAC_000163.1	42328519	42330294	1776	-1.2305	1.84E-21
CNVR_2255	chrAC_000163.1	46571383	46574046	2664	-1.7809	3.24E-50
CNVR_2256	chrAC_000163.1	48505447	48508998	3552	-3.0289	5.86E-106
CNVR_2257	chrAC_000163.1	48777175	48781614	4440	-3.0289	5.90E-132
CNVR_2258	chrAC_000163.1	49079095	49080870	1776	-1.3252	1.01E-23
CNVR_2259	chrAC_000163.1	49143475	49145250	1776	-1.8252	5.46E-35
CNVR_2260	chrAC_000163.1	49152355	49154130	1776	-2.7622	9.61E-51
CNVR_2261	chrAC_000163.1	49583923	49586142	2220	-2.0019	9.61E-48
CNVR_2262	chrAC_000163.1	49862755	49866306	3552	-3.7203	3.81E-118
CNVR_2263	chrAC_000163.1	50730775	50732550	1776	-1.2237	2.67E-21
CNVR_2264	chrAC_000163.1	50967427	50969202	1776	-1.1525	1.34E-19
CNVR_2265	chrAC_000163.1	53449387	53451606	2220	-1.6327	4.09E-38
CNVR_2266	chrAC_000163.1	53461819	53463594	1776	-1.2302	1.87E-21
CNVR_2267	chrAC_000163.1	54279223	54280998	1776	-1.1149	1.05E-18
CNVR_2268	chrAC_000163.1	59961091	59963310	2220	-1.4815	8.03E-34
CNVR_2269	chrAC_000163.1	63588127	63590346	2220	-1.7166	2.03E-40
CNVR_2270	chrAC_000163.1	63590791	63593454	2664	-1.5100	3.15E-41
CNVR_2271	chrAC_000163.1	65806795	65808570	1776	-1.6171	1.75E-30
CNVR_2272	chrAC_000163.1	66406639	66408414	1776	-1.5214	2.59E-28
CNVR_2273	chrAC_000163.1	66484783	66486558	1776	-1.8679	7.17E-36
CNVR_2274	chrAC_000163.1	66488779	66490554	1776	-1.2771	1.41E-22
CNVR_2275	chrAC_000163.1	71799463	71801238	1776	-1.4980	8.99E-28
CNVR_2276	chrAC_000163.1	75929107	75930882	1776	-1.2308	1.80E-21
CNVR_2277	chrAC_000163.1	78613087	78614862	1776	-1.3020	3.61E-23
CNVR_2278	chrAC_000163.1	79933987	79935762	1776	-1.3758	6.44E-25
CNVR_2279	chrAC_000163.1	80310055	80311830	1776	-2.3779	3.36E-45
CNVR_2280	chrAC_000163.1	80414839	80416614	1776	-1.1478	1.74E-19
CNVR_2281	chrAC_000163.1	80429935	80432154	2220	-2.0608	2.29E-87
CNVR_2282	chrAC_000163.1	80433043	80436594	3552	-3.4313	1.05E-113
CNVR_2283	chrAC_000163.1	80930323	80932098	1776	-2.2666	2.32E-43
CNVR_2284	chrAC_000163.1	85457791	85460010	2220	-3.0829	1.17E-67

CNVR_2285	chrAC_000163.1	88038763	88042314	3552	-2.5226	5.01E-93
CNVR_2286	chrAC_000163.1	88045867	88047642	1776	-3.9591	1.47E-61
CNVR_2287	chrAC_000163.1	90937195	90941190	3996	-2.0537	1.07E-86
CNVR_2288	chrAC_000163.1	91533487	91537482	3996	-3.8358	2.08E-134
CNVR_2289	chrAC_000163.1	92546251	92550690	4440	-2.0232	1.14E-94
CNVR_2290	chrAC_000163.1	93815647	93817422	1776	-3.4181	1.11E-57
CNVR_2291	chrAC_000163.1	96937411	96939186	1776	-1.9005	1.55E-36
CNVR_2292	chrAC_000163.1	97416043	97419594	3552	-1.4720	1.25E-52
CNVR_2293	chrAC_000163.1	97446679	97448454	1776	-1.5774	1.37E-29
CNVR_2294	chrAC_000163.1	97448899	97450674	1776	-2.0695	7.83E-40
CNVR_2295	chrAC_000163.1	101891563	101893338	1776	-3.1943	1.25E-55
CNVR_2296	chrAC_000163.1	102708523	102710298	1776	-1.4609	6.49E-27
CNVR_2297	chrAC_000163.1	107620495	107622714	2220	-3.3224	1.37E-70
CNVR_2298	chrAC_000163.1	115431343	115433562	2220	-1.4813	8.15E-34
CNVR_2299	chrAC_000163.1	117844927	117846702	1776	-1.9995	1.69E-38
CNVR_2300	chrAC_000163.1	118043395	118045170	1776	-1.8493	1.73E-35
CNVR_2301	chrAC_000163.1	118421683	118423458	1776	-1.4658	5.01E-27
CNVR_2302	chrAC_000186.1	27755107	27756882	1776	1.7616	5.54E-150
CNVR_2303	chrAC_000186.1	28031719	28033938	2220	1.7335	2.76E-183
CNVR_2304	chrAC_000186.1	1803307	1805526	2220	-1.8521	5.57E-179
CNVR_2305	chrAC_000186.1	3358639	3360414	1776	-1.1783	8.28E-79
CNVR_2306	chrAC_000186.1	3862579	3864798	2220	-1.5581	4.33E-145
CNVR_2307	chrAC_000186.1	4305691	4307466	1776	-1.1592	6.79E-77
CNVR_2308	chrAC_000186.1	4432675	4434450	1776	-1.3991	6.76E-101
CNVR_2309	chrAC_000186.1	4473079	4476186	3108	-1.2779	6.54E-154
CNVR_2310	chrAC_000186.1	5393935	5396154	2220	-1.3673	1.12E-121
CNVR_2311	chrAC_000186.1	5433895	5436558	2664	-1.3098	5.23E-137
CNVR_2312	chrAC_000186.1	5461867	5467194	5328	-1.6678	0.00E+00
CNVR_2313	chrAC_000186.1	5467639	5472078	4440	-1.7919	0.00E+00
CNVR_2314	chrAC_000186.1	5472523	5474742	2220	-1.3602	8.56E-121
CNVR_2315	chrAC_000186.1	5488951	5490726	1776	-1.4128	2.96E-102
CNVR_2316	chrAC_000186.1	5493391	5496054	2664	-1.1770	4.70E-117
CNVR_2317	chrAC_000186.1	5501827	5503602	1776	-1.1017	3.70E-71
CNVR_2318	chrAC_000186.1	5521807	5524914	3108	-2.0887	2.83E-284
CNVR_2319	chrAC_000186.1	5525803	5531574	5772	-1.4860	0.00E+00
CNVR_2320	chrAC_000186.1	5540455	5542230	1776	-1.1393	6.60E-75
CNVR_2321	chrAC_000186.1	5544451	5547114	2664	-1.4019	8.85E-151
CNVR_2322	chrAC_000186.1	5549335	5577750	28416	-1.9384	0.00E+00
CNVR_2323	chrAC_000186.1	5601727	5616378	14652	-1.3365	0.00E+00
CNVR_2324	chrAC_000186.1	5616823	5619486	2664	-1.7794	1.15E-204
CNVR_2325	chrAC_000186.1	5622151	5624370	2220	-1.1931	6.97E-100
CNVR_2326	chrAC_000186.1	5678095	5680758	2664	-1.3023	7.03E-136
CNVR_2327	chrAC_000186.1	5685643	5687418	1776	-1.2012	4.23E-81
CNVR_2328	chrAC_000186.1	5692303	5695410	3108	-1.4043	5.71E-176

CNVR_2329	chrAC_000186.1	5695855	5700738	4884	-1.3579	1.16E-262
CNVR_2330	chrAC_000186.1	5708287	5710062	1776	-1.1194	6.33E-73
CNVR_2331	chrAC_000186.1	5714947	5717610	2664	-1.2924	2.19E-134
CNVR_2332	chrAC_000186.1	5725159	5727378	2220	-1.3949	4.18E-125
CNVR_2333	chrAC_000186.1	5733595	5741142	7548	-1.3317	0.00E+00
CNVR_2334	chrAC_000186.1	5758015	5759790	1776	-1.0510	3.80E-66
CNVR_2335	chrAC_000186.1	5762455	5764674	2220	-1.1683	8.88E-97
CNVR_2336	chrAC_000186.1	5777107	5778882	1776	-1.3988	7.19E-101
CNVR_2337	chrAC_000186.1	5781991	5791758	9768	-1.4940	0.00E+00
CNVR_2338	chrAC_000186.1	5792647	5794866	2220	-1.4342	5.68E-130
CNVR_2339	chrAC_000186.1	5900539	5902314	1776	-1.7415	1.08E-133
CNVR_2340	chrAC_000186.1	6081247	6083466	2220	-1.3609	7.02E-121
CNVR_2341	chrAC_000186.1	6113215	6115878	2664	-1.5575	1.31E-173
CNVR_2342	chrAC_000186.1	7898095	7900314	2220	-1.1759	9.84E-98
CNVR_2343	chrAC_000186.1	7902091	7903866	1776	-1.2233	2.61E-83
CNVR_2344	chrAC_000186.1	13541779	13543554	1776	-1.4267	1.25E-103
CNVR_2345	chrAC_000186.1	13580851	13583514	2664	-2.0085	2.83E-234
CNVR_2346	chrAC_000186.1	15086899	15088674	1776	-1.5854	4.40E-119
CNVR_2347	chrAC_000186.1	18454195	18455970	1776	-1.6106	1.72E-121
CNVR_2348	chrAC_000186.1	19063363	19066914	3552	-1.2689	8.51E-174
CNVR_2349	chrAC_000186.1	19305787	19307562	1776	-1.6108	1.66E-121
CNVR_2350	chrAC_000186.1	19355515	19357734	2220	-1.2855	1.85E-111
CNVR_2351	chrAC_000186.1	20483719	20486382	2664	-1.6137	1.16E-181
CNVR_2352	chrAC_000186.1	20625799	20627574	1776	-2.3722	2.84E-184
CNVR_2353	chrAC_000186.1	23827039	23830146	3108	-1.5010	1.26E-192
CNVR_2354	chrAC_000186.1	25260271	25262490	2220	-2.7541	3.00E-259
CNVR_2355	chrAC_000186.1	27060691	27067350	6660	-3.0376	0.00E+00
CNVR_2356	chrAC_000186.1	27345295	27347070	1776	-1.2051	1.74E-81
CNVR_2357	chrAC_000186.1	27351067	27355506	4440	-2.3109	0.00E+00
CNVR_2358	chrAC_000186.1	27415003	27416778	1776	-1.7479	2.82E-134
CNVR_2359	chrAC_000186.1	27476719	27479382	2664	-1.4682	1.37E-160
CNVR_2360	chrAC_000186.1	27479827	27482490	2664	-1.4816	1.47E-162
CNVR_2361	chrAC_000186.1	27496255	27498918	2664	-2.3804	3.40E-276
CNVR_2362	chrAC_000186.1	27638779	27673854	35076	-3.7803	0.00E+00
CNVR_2363	chrAC_000186.1	27752443	27754662	2220	-1.2297	1.80E-104
CNVR_2364	chrAC_000186.1	29472943	29475162	2220	-3.5238	2.55E-301
CNVR_2365	chrAC_000186.1	29475607	29477826	2220	-4.1984	0.00E+00
CNVR_2366	chrAC_000186.1	32039263	32041038	1776	-1.5815	1.04E-118
CNVR_2367	chrAC_000186.1	33589267	33591042	1776	-1.0911	4.19E-70
CNVR_2368	chrAC_000186.1	36470383	36472602	2220	-1.6242	5.61E-153
CNVR_2369	chrAC_000186.1	39115291	39142374	27084	-3.5670	0.00E+00
CNVR_2370	chrAC_000186.1	39340843	39344394	3552	-3.1532	0.00E+00
CNVR_2371	chrAC_000186.1	39345283	39366594	21312	-3.4433	0.00E+00
CNVR_2372	chrAC_000186.1	41444959	41446734	1776	-1.5296	1.03E-113

CNVR_2373	chrAC_000186.1	41749099	41750874	1776	-1.3124	3.03E-92
CNVR_2374	chrAC_000186.1	42513667	42515886	2220	-1.1550	4.04E-95
CNVR_2375	chrAC_000186.1	42523879	42526098	2220	-1.9287	3.06E-187
CNVR_2376	chrAC_000186.1	44134267	44136042	1776	-1.4083	8.28E-102
CNVR_2377	chrAC_000186.1	49294435	49296210	1776	-1.6825	3.00E-128
CNVR_2378	chrAC_000186.1	49908487	49910706	2220	-1.1545	4.67E-95
CNVR_2379	chrAC_000186.1	49912483	49915146	2664	-2.0690	1.29E-241
CNVR_2380	chrAC_000186.1	49916035	49917810	1776	-1.3784	7.66E-99
CNVR_2381	chrAC_000186.1	50705911	50709462	3552	-2.0610	0.00E+00
CNVR_2382	chrAC_000186.1	50879515	50881290	1776	-2.5376	4.19E-195
CNVR_2383	chrAC_000185.1	1642135	1643910	1776	1.3190	4.00E-39
CNVR_2384	chrAC_000185.1	2538571	2540346	1776	1.3082	1.18E-38
CNVR_2385	chrAC_000185.1	2582971	2584746	1776	1.1971	8.09E-34
CNVR_2386	chrAC_000185.1	3270727	3272502	1776	1.2699	5.53E-37
CNVR_2387	chrAC_000185.1	11356411	11358186	1776	1.2871	9.78E-38
CNVR_2388	chrAC_000185.1	16946371	16948590	2220	1.7101	2.31E-69
CNVR_2389	chrAC_000185.1	27979327	27981102	1776	1.3528	1.33E-40
CNVR_2390	chrAC_000185.1	43022047	43023822	1776	1.6434	4.31E-53
CNVR_2391	chrAC_000185.1	911755	913530	1776	-1.5811	3.56E-41
CNVR_2392	chrAC_000185.1	3150847	3155730	4884	-2.3610	9.20E-169
CNVR_2393	chrAC_000185.1	14776543	14778318	1776	-1.6921	1.21E-44
CNVR_2394	chrAC_000185.1	19250287	19252062	1776	-2.1985	9.81E-59
CNVR_2395	chrAC_000185.1	20675527	20678634	3108	-1.7377	3.39E-79
CNVR_2396	chrAC_000185.1	22677967	22680630	2664	-1.3502	1.33E-49
CNVR_2397	chrAC_000185.1	22768543	22771206	2664	-1.2536	8.97E-45
CNVR_2398	chrAC_000185.1	23481163	23483826	2664	-1.5248	3.54E-58
CNVR_2399	chrAC_000185.1	25474723	25476942	2220	-1.7530	1.14E-57
CNVR_2400	chrAC_000185.1	29155039	29156814	1776	-2.8236	7.51E-72
CNVR_2401	chrAC_000185.1	29158591	29163918	5328	-2.6559	1.96E-202
CNVR_2402	chrAC_000185.1	29733571	29735346	1776	-1.3802	1.20E-34
CNVR_2403	chrAC_000185.1	33000079	33001854	1776	-1.5381	8.40E-40
CNVR_2404	chrAC_000185.1	38066119	38068782	2664	-1.9757	1.89E-78
CNVR_2405	chrAC_000185.1	38081659	38083434	1776	-1.4487	6.69E-37
CNVR_2406	chrAC_000185.1	46265911	46267686	1776	-1.5790	4.14E-41
CNVR_2407	chrAC_000174.1	2400931	2403150	2220	1.1822	1.22E-32
CNVR_2408	chrAC_000174.1	8861575	8863350	1776	1.2650	2.96E-29
CNVR_2409	chrAC_000174.1	16008643	16010418	1776	2.4489	1.40E-64
CNVR_2410	chrAC_000174.1	25048483	25050258	1776	1.0400	1.05E-21
CNVR_2411	chrAC_000174.1	28284799	28287462	2664	1.3437	4.85E-47
CNVR_2412	chrAC_000174.1	35524663	35526438	1776	1.3348	1.25E-31
CNVR_2413	chrAC_000174.1	38341843	38343618	1776	2.2363	2.12E-59
CNVR_2414	chrAC_000174.1	40318087	40322526	4440	1.6011	9.51E-99
CNVR_2415	chrAC_000174.1	46368919	46370694	1776	1.2319	3.95E-28
CNVR_2416	chrAC_000174.1	50579815	50584254	4440	1.6489	1.20E-102

CNVR_2417	chrAC_000174.1	50586475	50589138	2664	1.5726	1.37E-58
CNVR_2418	chrAC_000174.1	50618443	50620218	1776	1.1718	4.29E-26
CNVR_2419	chrAC_000174.1	50653963	50657958	3996	1.3152	1.74E-67
CNVR_2420	chrAC_000174.1	50700583	50702358	1776	1.7857	1.60E-46
CNVR_2421	chrAC_000174.1	50846215	50847990	1776	1.4655	4.55E-36
CNVR_2422	chrAC_000174.1	51135703	51137922	2220	1.0665	8.22E-28
CNVR_2423	chrAC_000174.1	51151243	51153906	2664	1.0808	9.55E-34
CNVR_2424	chrAC_000174.1	51296431	51300870	4440	1.1546	3.26E-61
CNVR_2425	chrAC_000174.1	51433183	51437622	4440	1.2766	1.69E-71
CNVR_2426	chrAC_000174.1	51440731	51442950	2220	1.0833	1.67E-28
CNVR_2427	chrAC_000174.1	51447835	51461598	13764	1.5304	9.00E-284
CNVR_2428	chrAC_000174.1	51471811	51473586	1776	1.3557	2.40E-32
CNVR_2429	chrAC_000174.1	51476251	51489126	12876	1.3404	2.69E-219
CNVR_2430	chrAC_000174.1	51490015	51493122	3108	1.1379	2.08E-42
CNVR_2431	chrAC_000174.1	51493567	51498006	4440	1.4000	5.89E-82
CNVR_2432	chrAC_000174.1	51498895	51505554	6660	1.9219	8.28E-185
CNVR_2433	chrAC_000174.1	51506887	51515766	8880	1.3320	1.89E-150
CNVR_2434	chrAC_000174.1	51516655	51518874	2220	1.1507	2.58E-31
CNVR_2435	chrAC_000174.1	51519763	51529086	9324	1.1675	1.00E-128
CNVR_2436	chrAC_000174.1	70229923	70231698	1776	1.3652	1.14E-32
CNVR_2437	chrAC_000174.1	72869059	72870834	1776	1.1286	1.22E-24
CNVR_2438	chrAC_000174.1	72881047	72888150	7104	1.3376	1.59E-121
CNVR_2439	chrAC_000174.1	72901915	72904578	2664	1.1029	7.64E-35
CNVR_2440	chrAC_000174.1	72905467	72908130	2664	1.2443	5.61E-42
CNVR_2441	chrAC_000174.1	72930331	72939654	9324	1.3685	2.30E-164
CNVR_2442	chrAC_000174.1	72941431	72944538	3108	1.4604	1.71E-61
CNVR_2443	chrAC_000174.1	75137011	75141450	4440	1.4093	9.58E-83
CNVR_2444	chrAC_000174.1	75142339	75144558	2220	1.1767	2.08E-32
CNVR_2445	chrAC_000174.1	1431679	1433454	1776	-1.2630	3.09E-25
CNVR_2446	chrAC_000174.1	1471195	1472970	1776	-1.7477	5.40E-38
CNVR_2447	chrAC_000174.1	1591963	1593738	1776	-1.8520	1.62E-40
CNVR_2448	chrAC_000174.1	5563099	5567094	3996	-1.8962	3.13E-91
CNVR_2449	chrAC_000174.1	6992335	6994998	2664	-1.5418	2.97E-48
CNVR_2450	chrAC_000174.1	14344087	14347194	3108	-1.5155	8.70E-55
CNVR_2451	chrAC_000174.1	14347639	14350302	2664	-1.4121	4.35E-43
CNVR_2452	chrAC_000174.1	14395591	14397366	1776	-2.0041	5.19E-44
CNVR_2453	chrAC_000174.1	14401807	14404026	2220	-1.5703	1.96E-41
CNVR_2454	chrAC_000174.1	15323107	15326214	3108	-6.2671	1.00E-135
CNVR_2455	chrAC_000174.1	15327991	15332430	4440	-2.9180	1.30E-147
CNVR_2456	chrAC_000174.1	16010863	16013082	2220	-2.2599	2.93E-61
CNVR_2457	chrAC_000174.1	16159603	16161378	1776	-1.1112	4.42E-21
CNVR_2458	chrAC_000174.1	18759223	18761886	2664	-1.6821	1.16E-53
CNVR_2459	chrAC_000174.1	21900523	21903186	2664	-1.5885	4.48E-50
CNVR_2460	chrAC_000174.1	22014187	22016406	2220	-1.5897	4.61E-42

CNVR_2461	chrAC_000174.1	22202443	22204218	1776	-1.6046	2.22E-34
CNVR_2462	chrAC_000174.1	22212211	22214430	2220	-1.3494	4.49E-34
CNVR_2463	chrAC_000174.1	22713043	22715262	2220	-1.2107	2.41E-29
CNVR_2464	chrAC_000174.1	23502031	23503806	1776	-1.1642	1.57E-22
CNVR_2465	chrAC_000174.1	24559639	24561858	2220	-1.7327	1.36E-46
CNVR_2466	chrAC_000174.1	24679963	24681738	1776	-1.1437	5.72E-22
CNVR_2467	chrAC_000174.1	25637227	25639002	1776	-1.9209	3.96E-42
CNVR_2468	chrAC_000174.1	25780195	25781970	1776	-1.5584	3.52E-33
CNVR_2469	chrAC_000174.1	31422103	31423878	1776	-1.5377	1.23E-32
CNVR_2470	chrAC_000174.1	35641879	35645430	3552	-1.5522	2.80E-64
CNVR_2471	chrAC_000174.1	35647207	35648982	1776	-1.7876	5.70E-39
CNVR_2472	chrAC_000174.1	35758651	35760426	1776	-1.1267	1.66E-21
CNVR_2473	chrAC_000174.1	35974435	35976210	1776	-2.5265	3.01E-54
CNVR_2474	chrAC_000174.1	35989087	35996634	7548	-1.9055	1.78E-171
CNVR_2475	chrAC_000174.1	36093427	36095202	1776	-1.2510	6.60E-25
CNVR_2476	chrAC_000174.1	38247715	38249490	1776	-1.6888	1.59E-36
CNVR_2477	chrAC_000174.1	39510451	39513114	2664	-1.3569	7.54E-41
CNVR_2478	chrAC_000174.1	39872311	39875418	3108	-1.3396	1.52E-46
CNVR_2479	chrAC_000174.1	39877195	39884298	7104	-2.2913	3.17E-194
CNVR_2480	chrAC_000174.1	41474707	41478258	3552	-2.8542	9.65E-117
CNVR_2481	chrAC_000174.1	41646535	41648754	2220	-1.3878	2.25E-35
CNVR_2482	chrAC_000174.1	42060343	42062562	2220	-2.2009	8.64E-60
CNVR_2483	chrAC_000174.1	42979423	42981198	1776	-1.2446	9.85E-25
CNVR_2484	chrAC_000174.1	44130271	44133378	3108	-1.4075	9.34E-50
CNVR_2485	chrAC_000174.1	44438407	44440182	1776	-1.1983	1.83E-23
CNVR_2486	chrAC_000174.1	44840227	44843334	3108	-1.5390	7.25E-56
CNVR_2487	chrAC_000174.1	44898391	44900166	1776	-1.5461	7.41E-33
CNVR_2488	chrAC_000174.1	45342835	45348162	5328	-2.6493	5.65E-165
CNVR_2489	chrAC_000174.1	50770291	50772954	2664	-1.4271	1.09E-43
CNVR_2490	chrAC_000174.1	52443727	52446390	2664	-2.0447	1.84E-66
CNVR_2491	chrAC_000174.1	52449055	52450830	1776	-2.7374	1.28E-57
CNVR_2492	chrAC_000174.1	52471255	52473918	2664	-2.3385	2.09E-75
CNVR_2493	chrAC_000174.1	52474807	52478802	3996	-2.8207	5.07E-130
CNVR_2494	chrAC_000174.1	70205503	70209054	3552	-1.6277	3.52E-68
CNVR_2495	chrAC_000174.1	73090171	73096386	6216	-4.5446	4.04E-253
CNVR_2496	chrAC_000174.1	75028675	75030450	1776	-1.0921	1.46E-20
CNVR_2497	chrAC_000165.1	15692515	15695178	2664	1.4612	5.06E-72
CNVR_2498	chrAC_000165.1	61266007	61267782	1776	1.4379	1.50E-47
CNVR_2499	chrAC_000165.1	89391187	89392962	1776	1.9556	4.92E-70
CNVR_2500	chrAC_000165.1	91970827	91973934	3108	1.5480	1.24E-90
CNVR_2501	chrAC_000165.1	91974379	91977486	3108	1.4678	3.02E-84
CNVR_2502	chrAC_000165.1	92025883	92028546	2664	1.5836	2.32E-80
CNVR_2503	chrAC_000165.1	99199147	99201366	2220	1.5555	1.07E-65
CNVR_2504	chrAC_000165.1	106302703	106304478	1776	1.3505	1.66E-43

CNVR_2505	chrAC_000165.1	169387	182706	13320	-3.8171	0.00E+00
CNVR_2506	chrAC_000165.1	937951	939726	1776	-1.4216	6.76E-41
CNVR_2507	chrAC_000165.1	2142523	2144298	1776	-1.5302	5.58E-45
CNVR_2508	chrAC_000165.1	2285935	2289042	3108	-3.8419	3.96E-169
CNVR_2509	chrAC_000165.1	3349759	3351534	1776	-1.3920	9.02E-40
CNVR_2510	chrAC_000165.1	3715615	3717390	1776	-1.6559	1.42E-49
CNVR_2511	chrAC_000165.1	4179595	4181814	2220	-2.9663	6.51E-106
CNVR_2512	chrAC_000165.1	4283935	4286598	2664	-2.9174	2.51E-125
CNVR_2513	chrAC_000165.1	4314127	4316790	2664	-2.0000	7.24E-91
CNVR_2514	chrAC_000165.1	4343875	4346094	2220	-2.2233	2.21E-84
CNVR_2515	chrAC_000165.1	6293923	6297474	3552	-3.4486	1.33E-183
CNVR_2516	chrAC_000165.1	11296471	11300466	3996	-1.3227	2.82E-81
CNVR_2517	chrAC_000165.1	13100887	13102662	1776	-1.4372	1.73E-41
CNVR_2518	chrAC_000165.1	13327771	13329546	1776	-1.6190	3.05E-48
CNVR_2519	chrAC_000165.1	13352191	13353966	1776	-1.2013	1.95E-32
CNVR_2520	chrAC_000165.1	13427671	13429890	2220	-1.7042	1.15E-63
CNVR_2521	chrAC_000165.1	14358295	14361846	3552	-1.8397	4.47E-110
CNVR_2522	chrAC_000165.1	14571415	14575854	4440	-2.2690	5.49E-170
CNVR_2523	chrAC_000165.1	14841367	14843142	1776	-1.2695	4.54E-35
CNVR_2524	chrAC_000165.1	19959355	19961574	2220	-4.3897	2.26E-127
CNVR_2525	chrAC_000165.1	26002639	26006190	3552	-2.5940	7.59E-153
CNVR_2526	chrAC_000165.1	26339635	26341410	1776	-2.2635	5.26E-69
CNVR_2527	chrAC_000165.1	26882203	26883978	1776	-1.2742	3.00E-35
CNVR_2528	chrAC_000165.1	30496363	30498138	1776	-2.1757	1.62E-66
CNVR_2529	chrAC_000165.1	30732127	30733902	1776	-1.2709	4.02E-35
CNVR_2530	chrAC_000165.1	31807939	31810158	2220	-2.0292	3.53E-77
CNVR_2531	chrAC_000165.1	32669299	32671074	1776	-1.1940	3.74E-32
CNVR_2532	chrAC_000165.1	33866767	33868542	1776	-1.1721	2.62E-31
CNVR_2533	chrAC_000165.1	34296559	34299222	2664	-1.4633	8.10E-63
CNVR_2534	chrAC_000165.1	35664079	35667186	3108	-1.2245	5.06E-57
CNVR_2535	chrAC_000165.1	36372703	36374478	1776	-1.4375	1.68E-41
CNVR_2536	chrAC_000165.1	37474711	37477374	2664	-1.1526	5.57E-45
CNVR_2537	chrAC_000165.1	37678063	37679838	1776	-1.5813	7.23E-47
CNVR_2538	chrAC_000165.1	39579715	39583266	3552	-1.3214	2.36E-72
CNVR_2539	chrAC_000165.1	40732783	40735002	2220	-2.8133	3.28E-102
CNVR_2540	chrAC_000165.1	41117287	41139486	22200	-3.2957	0.00E+00
CNVR_2541	chrAC_000165.1	44798047	44799822	1776	-1.4090	2.04E-40
CNVR_2542	chrAC_000165.1	44933911	44935686	1776	-1.3214	4.57E-37
CNVR_2543	chrAC_000165.1	44939683	44941458	1776	-1.2809	1.65E-35
CNVR_2544	chrAC_000165.1	45465379	45472482	7104	-3.2615	0.00E+00
CNVR_2545	chrAC_000165.1	52465039	52467258	2220	-1.3385	1.02E-46
CNVR_2546	chrAC_000165.1	53890723	53893830	3108	-1.3216	1.49E-63
CNVR_2547	chrAC_000165.1	54029695	54032802	3108	-1.3275	6.04E-64
CNVR_2548	chrAC_000165.1	54704575	54707238	2664	-1.6094	6.21E-71

CNVR_2549	chrAC_000165.1	55123267	55125486	2220	-2.0685	1.08E-78
CNVR_2550	chrAC_000165.1	55896271	55898046	1776	-1.7153	1.09E-51
CNVR_2551	chrAC_000165.1	62146015	62147790	1776	-1.1857	7.78E-32
CNVR_2552	chrAC_000165.1	62158003	62162442	4440	-1.3331	4.40E-91
CNVR_2553	chrAC_000165.1	62174875	62177982	3108	-1.3239	1.04E-63
CNVR_2554	chrAC_000165.1	62182867	62185530	2664	-1.7036	5.40E-76
CNVR_2555	chrAC_000165.1	62187751	62190858	3108	-1.3674	1.27E-66
CNVR_2556	chrAC_000165.1	66049663	66051438	1776	-2.0534	7.29E-63
CNVR_2557	chrAC_000165.1	66212167	66213942	1776	-1.3109	1.16E-36
CNVR_2558	chrAC_000165.1	66214831	66216606	1776	-1.3164	7.08E-37
CNVR_2559	chrAC_000165.1	66286315	66289422	3108	-1.7384	1.76E-90
CNVR_2560	chrAC_000165.1	66933223	66936330	3108	-1.5273	3.63E-77
CNVR_2561	chrAC_000165.1	67944655	67946874	2220	-1.1486	1.39E-37
CNVR_2562	chrAC_000165.1	68413075	68414850	1776	-1.2866	9.94E-36
CNVR_2563	chrAC_000165.1	70893259	70895478	2220	-2.1220	1.05E-80
CNVR_2564	chrAC_000165.1	70949203	70950978	1776	-1.4790	4.56E-43
CNVR_2565	chrAC_000165.1	70951423	70957638	6216	-2.7610	9.13E-279
CNVR_2566	chrAC_000165.1	70960303	70962966	2664	-2.8565	1.48E-123
CNVR_2567	chrAC_000165.1	71081071	71082846	1776	-1.8753	3.63E-57
CNVR_2568	chrAC_000165.1	79807891	79810110	2220	-2.0672	1.21E-78
CNVR_2569	chrAC_000165.1	80047207	80051646	4440	-1.7605	2.37E-130
CNVR_2570	chrAC_000165.1	80052979	80054754	1776	-2.4133	5.35E-73
CNVR_2571	chrAC_000165.1	80084947	80087166	2220	-1.2477	2.35E-42
CNVR_2572	chrAC_000165.1	80776255	80779362	3108	-1.7432	8.94E-91
CNVR_2573	chrAC_000165.1	81347239	81349014	1776	-1.4020	3.76E-40
CNVR_2574	chrAC_000165.1	84699883	84701658	1776	-1.2690	4.76E-35
CNVR_2575	chrAC_000165.1	87552139	87553914	1776	-1.2191	3.99E-33
CNVR_2576	chrAC_000165.1	88594207	88596870	2664	-1.4262	1.03E-60
CNVR_2577	chrAC_000165.1	88754935	88757154	2220	-1.6953	2.86E-63
CNVR_2578	chrAC_000165.1	89069731	89071950	2220	-1.3068	3.38E-45
CNVR_2579	chrAC_000165.1	89474215	89476434	2220	-1.8978	6.27E-72
CNVR_2580	chrAC_000165.1	89484427	89486202	1776	-1.3860	1.53E-39
CNVR_2581	chrAC_000165.1	92889019	92891682	2664	-1.8317	1.23E-82
CNVR_2582	chrAC_000165.1	93313483	93315702	2220	-1.8822	2.77E-71
CNVR_2583	chrAC_000165.1	93508399	93510618	2220	-1.4056	6.35E-50
CNVR_2584	chrAC_000165.1	93962167	93968382	6216	-1.5775	7.40E-159
CNVR_2585	chrAC_000165.1	94287175	94292946	5772	-1.3447	2.84E-119
CNVR_2586	chrAC_000165.1	94429699	94432806	3108	-2.0838	3.25E-110
CNVR_2587	chrAC_000165.1	94764919	94766694	1776	-1.5928	2.76E-47
CNVR_2588	chrAC_000165.1	94773799	94775574	1776	-1.7649	2.02E-53
CNVR_2589	chrAC_000165.1	94777351	94779126	1776	-2.0681	2.58E-63
CNVR_2590	chrAC_000165.1	110995783	110998002	2220	-1.5782	5.46E-58
CNVR_2591	chrAC_000165.1	110998447	111001110	2664	-1.6507	3.61E-73
CNVR_2592	chrAC_000165.1	111001555	111008658	7104	-2.1098	1.98E-252

CNVR_2593	chrAC_000165.1	111995227	111997890	2664	-1.5804	2.41E-69
CNVR_2594	chrAC_000165.1	112413475	112416582	3108	-2.0407	6.58E-108
CNVR_2595	chrAC_000158.1	2810743	2812518	1776	2.2398	1.07E-108
CNVR_2596	chrAC_000158.1	7449211	7450986	1776	1.3184	7.13E-56
CNVR_2597	chrAC_000158.1	12931279	12933054	1776	2.0935	1.95E-101
CNVR_2598	chrAC_000158.1	15584179	15586398	2220	2.0692	9.84E-125
CNVR_2599	chrAC_000158.1	23768431	23770206	1776	1.6474	2.41E-76
CNVR_2600	chrAC_000158.1	46188211	46189986	1776	2.1693	2.89E-105
CNVR_2601	chrAC_000158.1	49563943	49565718	1776	1.8198	1.60E-86
CNVR_2602	chrAC_000158.1	57443611	57446274	2664	1.2483	2.85E-76
CNVR_2603	chrAC_000158.1	93011119	93016446	5328	1.4043	4.98E-180
CNVR_2604	chrAC_000158.1	93717079	93727290	10212	1.7523	0.00E+00
CNVR_2605	chrAC_000158.1	93731287	93733506	2220	1.4902	7.07E-83
CNVR_2606	chrAC_000158.1	93737503	93739278	1776	1.3674	5.78E-59
CNVR_2607	chrAC_000158.1	93746827	93750378	3552	1.7066	7.65E-158
CNVR_2608	chrAC_000158.1	93751711	93753486	1776	1.3425	2.14E-57
CNVR_2609	chrAC_000158.1	93754819	93757038	2220	1.6427	1.32E-94
CNVR_2610	chrAC_000158.1	93757927	93760146	2220	1.9566	3.03E-117
CNVR_2611	chrAC_000158.1	93763255	93766806	3552	1.5333	8.71E-137
CNVR_2612	chrAC_000158.1	93773023	93776574	3552	1.4816	2.26E-130
CNVR_2613	chrAC_000158.1	93777463	93782346	4884	1.7005	2.88E-215
CNVR_2614	chrAC_000158.1	93782791	93789450	6660	1.7255	2.60E-298
CNVR_2615	chrAC_000158.1	93790783	93795666	4884	1.3815	2.92E-161
CNVR_2616	chrAC_000158.1	93796555	93798774	2220	1.6574	1.04E-95
CNVR_2617	chrAC_000158.1	93803215	93808098	4884	1.5694	1.95E-193
CNVR_2618	chrAC_000158.1	93811651	93814314	2664	1.9574	2.32E-140
CNVR_2619	chrAC_000158.1	93815203	93819198	3996	1.7405	7.89E-182
CNVR_2620	chrAC_000158.1	93820087	93824082	3996	1.6776	2.01E-173
CNVR_2621	chrAC_000158.1	97527043	97531482	4440	1.2503	3.86E-126
CNVR_2622	chrAC_000158.1	97533703	97536810	3108	1.3004	2.12E-94
CNVR_2623	chrAC_000158.1	97538143	97540362	2220	1.3869	8.72E-75
CNVR_2624	chrAC_000158.1	113459983	113461758	1776	1.2869	6.95E-54
CNVR_2625	chrAC_000158.1	124147951	124149726	1776	1.1235	1.16E-43
CNVR_2626	chrAC_000158.1	135000643	135002862	2220	1.1064	8.19E-53
CNVR_2627	chrAC_000158.1	141217531	141219306	1776	1.3481	9.45E-58
CNVR_2628	chrAC_000158.1	144106639	144108858	2220	1.1317	9.18E-55
CNVR_2629	chrAC_000158.1	148610131	148611906	1776	1.5248	7.84E-69
CNVR_2630	chrAC_000158.1	5218555	5220330	1776	-1.6580	1.50E-62
CNVR_2631	chrAC_000158.1	5353975	5355750	1776	-1.7396	3.51E-66
CNVR_2632	chrAC_000158.1	10563871	10565646	1776	-1.3703	4.38E-49
CNVR_2633	chrAC_000158.1	11706727	11708946	2220	-2.0295	2.74E-97
CNVR_2634	chrAC_000158.1	13811287	13813506	2220	-1.8673	4.25E-89
CNVR_2635	chrAC_000158.1	14653999	14659326	5328	-2.6398	5.53E-292
CNVR_2636	chrAC_000158.1	15063367	15068694	5328	-2.2648	1.16E-256

CNVR_2637	chrAC_000158.1	16020187	16028178	7992	-4.8757	0.00E+00
CNVR_2638	chrAC_000158.1	16426447	16429554	3108	-2.7464	2.65E-176
CNVR_2639	chrAC_000158.1	31351063	31353726	2664	-1.3772	3.25E-73
CNVR_2640	chrAC_000158.1	33018727	33020502	1776	-2.0702	8.80E-80
CNVR_2641	chrAC_000158.1	33224299	33226074	1776	-2.0341	2.16E-78
CNVR_2642	chrAC_000158.1	39285787	39288006	2220	-1.5024	9.14E-69
CNVR_2643	chrAC_000158.1	39430531	39432306	1776	-1.2583	1.16E-43
CNVR_2644	chrAC_000158.1	39450955	39454506	3552	-1.3250	5.80E-92
CNVR_2645	chrAC_000158.1	41194099	41196318	2220	-1.8079	5.89E-86
CNVR_2646	chrAC_000158.1	44524987	44527650	2664	-2.9155	8.46E-158
CNVR_2647	chrAC_000158.1	47229835	47231610	1776	-1.0796	5.52E-35
CNVR_2648	chrAC_000158.1	51169003	51171222	2220	-1.2496	1.32E-53
CNVR_2649	chrAC_000158.1	51174775	51176550	1776	-1.4112	4.75E-51
CNVR_2650	chrAC_000158.1	51180547	51182322	1776	-1.3396	1.32E-47
CNVR_2651	chrAC_000158.1	51185875	51189426	3552	-1.2351	2.95E-83
CNVR_2652	chrAC_000158.1	51220063	51224946	4884	-1.6443	6.02E-167
CNVR_2653	chrAC_000158.1	51228499	51230274	1776	-1.3000	1.09E-45
CNVR_2654	chrAC_000158.1	51234271	51236046	1776	-1.5408	3.56E-57
CNVR_2655	chrAC_000158.1	51243595	51246702	3108	-1.3356	1.34E-81
CNVR_2656	chrAC_000158.1	54249475	54251250	1776	-1.4917	7.16E-55
CNVR_2657	chrAC_000158.1	54372463	54374238	1776	-2.6227	3.38E-98
CNVR_2658	chrAC_000158.1	54467479	54469698	2220	-1.4902	4.81E-68
CNVR_2659	chrAC_000158.1	55253359	55255134	1776	-1.2464	4.39E-43
CNVR_2660	chrAC_000158.1	55321291	55323510	2220	-1.2666	1.23E-54
CNVR_2661	chrAC_000158.1	56366467	56368242	1776	-2.7102	1.25E-100
CNVR_2662	chrAC_000158.1	60357583	60360246	2664	-2.2124	1.35E-126
CNVR_2663	chrAC_000158.1	62254795	62257902	3108	-1.4924	1.08E-94
CNVR_2664	chrAC_000158.1	62262343	62265894	3552	-1.4471	1.15E-103
CNVR_2665	chrAC_000158.1	65481787	65484894	3108	-2.4680	2.74E-162
CNVR_2666	chrAC_000158.1	67040671	67042890	2220	-1.5793	3.08E-73
CNVR_2667	chrAC_000158.1	67065979	67067754	1776	-1.6898	5.61E-64
CNVR_2668	chrAC_000158.1	82549591	82551810	2220	-2.7354	3.82E-126
CNVR_2669	chrAC_000158.1	87449575	87452238	2664	-2.7737	1.75E-152
CNVR_2670	chrAC_000158.1	88215919	88218138	2220	-1.1746	4.74E-49
CNVR_2671	chrAC_000158.1	91186279	91189830	3552	-1.3159	4.35E-91
CNVR_2672	chrAC_000158.1	92320699	92322474	1776	-1.2334	1.87E-42
CNVR_2673	chrAC_000158.1	93678007	93680670	2664	-1.1328	1.86E-55
CNVR_2674	chrAC_000158.1	94253875	94255650	1776	-1.4677	9.65E-54
CNVR_2675	chrAC_000158.1	95767915	95771022	3108	-2.2647	1.41E-150
CNVR_2676	chrAC_000158.1	96641707	96643482	1776	-1.8840	2.38E-72
CNVR_2677	chrAC_000158.1	103541911	103543686	1776	-1.3631	9.74E-49
CNVR_2678	chrAC_000158.1	104683879	104685654	1776	-1.9387	1.34E-74
CNVR_2679	chrAC_000158.1	104968483	104970258	1776	-1.3687	5.23E-49
CNVR_2680	chrAC_000158.1	105200251	105206022	5772	-1.4548	2.15E-168

CNVR_2681	chrAC_000158.1	105293491	105296154	2664	-2.9732	7.82E-160
CNVR_2682	chrAC_000158.1	114377287	114379062	1776	-1.4765	3.70E-54
CNVR_2683	chrAC_000158.1	120887215	120891210	3996	-1.3624	2.88E-107
CNVR_2684	chrAC_000158.1	122586847	122589066	2220	-1.2491	1.42E-53
CNVR_2685	chrAC_000158.1	122745355	122747130	1776	-1.4100	5.43E-51
CNVR_2686	chrAC_000158.1	125899087	125904858	5772	-3.0950	0.00E+00
CNVR_2687	chrAC_000158.1	134097103	134098878	1776	-1.3482	5.07E-48
CNVR_2688	chrAC_000158.1	136465843	136468062	2220	-1.6022	1.50E-74
CNVR_2689	chrAC_000158.1	137222419	137225082	2664	-1.5577	5.15E-86
CNVR_2690	chrAC_000158.1	137773867	137776530	2664	-2.0287	2.62E-116
CNVR_2691	chrAC_000158.1	141210871	141213978	3108	-1.6134	1.64E-104
CNVR_2692	chrAC_000158.1	144669187	144670962	1776	-3.8191	2.59E-122
CNVR_2693	chrAC_000158.1	150055795	150059346	3552	-1.5869	9.77E-117
CNVR_2694	chrAC_000181.1	24851791	24853566	1776	1.8182	1.72E-43
CNVR_2695	chrAC_000181.1	56952103	56954322	2220	1.3735	2.18E-37
CNVR_2696	chrAC_000181.1	4663	10878	6216	-3.4652	8.95E-214
CNVR_2697	chrAC_000181.1	12211	26862	14652	-2.9862	0.00E+00
CNVR_2698	chrAC_000181.1	31303	33966	2664	-1.5687	2.78E-46
CNVR_2699	chrAC_000181.1	34411	98790	64380	-3.2950	0.00E+00
CNVR_2700	chrAC_000181.1	557443	560106	2664	-1.5461	1.88E-45
CNVR_2701	chrAC_000181.1	2945275	2947938	2664	-1.4277	5.04E-41
CNVR_2702	chrAC_000181.1	10631359	10633578	2220	-1.2519	7.22E-29
CNVR_2703	chrAC_000181.1	11518027	11519802	1776	-1.2516	2.06E-23
CNVR_2704	chrAC_000181.1	13163047	13166154	3108	-1.9155	9.41E-68
CNVR_2705	chrAC_000181.1	13232311	13234086	1776	-1.2409	3.87E-23
CNVR_2706	chrAC_000181.1	13389931	13391706	1776	-1.6003	3.37E-32
CNVR_2707	chrAC_000181.1	13404139	13405914	1776	-1.4069	2.19E-27
CNVR_2708	chrAC_000181.1	15196123	15198342	2220	-1.8581	2.47E-47
CNVR_2709	chrAC_000181.1	15669427	15671202	1776	-1.2478	2.57E-23
CNVR_2710	chrAC_000181.1	29413891	29415666	1776	-1.1583	5.17E-21
CNVR_2711	chrAC_000181.1	36988975	36992970	3996	-1.7371	1.48E-77
CNVR_2712	chrAC_000181.1	37188331	37190550	2220	-1.2506	7.96E-29
CNVR_2713	chrAC_000181.1	38899063	38902614	3552	-1.2517	3.73E-45
CNVR_2714	chrAC_000181.1	40647091	40649310	2220	-3.0914	6.79E-73
CNVR_2715	chrAC_000181.1	41493355	41495130	1776	-2.8014	4.52E-55
CNVR_2716	chrAC_000181.1	46484803	46487022	2220	-1.4376	8.74E-35
CNVR_2717	chrAC_000181.1	53261131	53263794	2664	-1.3494	4.80E-38
CNVR_2718	chrAC_000181.1	53322847	53327730	4884	-2.2856	1.10E-125
CNVR_2719	chrAC_000181.1	55594795	55596570	1776	-2.1273	7.03E-44
CNVR_2720	chrAC_000181.1	57774835	57777498	2664	-3.7288	5.34E-96
CNVR_2721	chrAC_000181.1	60126259	60128478	2220	-1.1526	1.11E-25
CNVR_2722	chrAC_000181.1	62276107	62277882	1776	-2.0186	1.13E-41
CNVR_2723	chrAC_000178.1	4251523	4253742	2220	1.2929	7.86E-33
CNVR_2724	chrAC_000178.1	5212339	5214114	1776	1.0868	1.66E-20

CNVR_2725	chrAC_000178.1	2740147	2744142	3996	-4.2249	9.00E-139
CNVR_2726	chrAC_000178.1	3482515	3485178	2664	-1.2659	1.86E-32
CNVR_2727	chrAC_000178.1	3741811	3743586	1776	-1.2344	1.95E-21
CNVR_2728	chrAC_000178.1	3775555	3777330	1776	-1.4054	1.77E-25
CNVR_2729	chrAC_000178.1	3901651	3903870	2220	-1.8672	3.60E-44
CNVR_2730	chrAC_000178.1	4861135	4863354	2220	-2.6779	2.62E-61
CNVR_2731	chrAC_000178.1	5004547	5006322	1776	-1.5429	1.18E-28
CNVR_2732	chrAC_000178.1	5572867	5575086	2220	-2.1020	7.19E-50
CNVR_2733	chrAC_000178.1	9401035	9403254	2220	-1.4211	6.82E-32
CNVR_2734	chrAC_000178.1	9630583	9635022	4440	-1.7490	2.31E-80
CNVR_2735	chrAC_000178.1	9638575	9640350	1776	-1.8190	1.09E-34
CNVR_2736	chrAC_000178.1	9951595	9953814	2220	-1.3131	1.01E-28
CNVR_2737	chrAC_000178.1	11445211	11446986	1776	-1.2534	6.91E-22
CNVR_2738	chrAC_000178.1	13270051	13272714	2664	-1.7142	7.88E-48
CNVR_2739	chrAC_000178.1	13286035	13287810	1776	-1.3874	4.67E-25
CNVR_2740	chrAC_000178.1	13294471	13297134	2664	-1.4209	6.39E-38
CNVR_2741	chrAC_000178.1	13298023	13299798	1776	-1.4358	3.45E-26
CNVR_2742	chrAC_000178.1	13301131	13303350	2220	-1.1784	9.93E-25
CNVR_2743	chrAC_000178.1	15051823	15054042	2220	-2.1805	1.22E-51
CNVR_2744	chrAC_000178.1	15241411	15243186	1776	-1.6112	3.39E-30
CNVR_2745	chrAC_000178.1	17241631	17243406	1776	-1.4407	2.66E-26
CNVR_2746	chrAC_000178.1	18730807	18733914	3108	-2.4680	7.85E-80
CNVR_2747	chrAC_000178.1	20059255	20062362	3108	-1.8332	6.71E-60
CNVR_2748	chrAC_000178.1	20066803	20069466	2664	-1.5413	4.45E-42
CNVR_2749	chrAC_000178.1	20069911	20072574	2664	-2.3397	1.16E-65
CNVR_2750	chrAC_000178.1	20073463	20077014	3552	-1.6139	8.98E-59
CNVR_2751	chrAC_000178.1	20077903	20081454	3552	-1.7504	1.01E-64
CNVR_2752	chrAC_000178.1	20082787	20089002	6216	-3.3705	9.14E-195
CNVR_2753	chrAC_000178.1	20091223	20097882	6660	-1.8814	6.92E-130
CNVR_2754	chrAC_000178.1	20101435	20106318	4884	-2.0994	2.37E-107
CNVR_2755	chrAC_000178.1	20115643	20119638	3996	-1.8772	1.64E-78
CNVR_2756	chrAC_000178.1	20120971	20135622	14652	-3.0968	0.00E+00
CNVR_2757	chrAC_000178.1	20136955	20140062	3108	-2.5974	3.82E-83
CNVR_2758	chrAC_000178.1	37269139	37270914	1776	-1.4468	1.92E-26
CNVR_2759	chrAC_000178.1	37599475	37601250	1776	-1.1347	4.59E-19
CNVR_2760	chrAC_000178.1	38003071	38006178	3108	-1.3158	1.21E-39
CNVR_2761	chrAC_000178.1	38642431	38644206	1776	-2.4585	2.97E-46
CNVR_2762	chrAC_000178.1	39278683	39280902	2220	-1.8534	8.12E-44
CNVR_2763	chrAC_000178.1	39345283	39347502	2220	-1.1326	2.26E-23
CNVR_2764	chrAC_000178.1	41335735	41337510	1776	-1.7413	4.79E-33
CNVR_2765	chrAC_000178.1	42566947	42568722	1776	-1.9945	3.24E-38
CNVR_2766	chrAC_000178.1	46288111	46290774	2664	-1.3901	7.64E-37
CNVR_2767	chrAC_000178.1	48022375	48024150	1776	-1.8190	1.09E-34
CNVR_2768	chrAC_000178.1	49284223	49286442	2220	-1.5187	1.04E-34

CNVR_2769	chrAC_000178.1	50034583	50036358	1776	-2.3070	7.75E-44
CNVR_2770	chrAC_000178.1	51676939	51679602	2664	-1.3621	7.37E-36
CNVR_2771	chrAC_000178.1	52070767	52073874	3108	-1.9533	3.86E-64
CNVR_2772	chrAC_000178.1	52172887	52174662	1776	-1.5577	5.42E-29
CNVR_2773	chrAC_000178.1	52549843	52551618	1776	-1.2345	1.94E-21
CNVR_2774	chrAC_000178.1	52748755	52752750	3996	-1.5407	3.39E-62
CNVR_2775	chrAC_000178.1	53551063	53552838	1776	-1.5715	2.65E-29
CNVR_2776	chrAC_000178.1	54027475	54029250	1776	-1.3968	2.82E-25
CNVR_2777	chrAC_000178.1	54531415	54533634	2220	-1.4300	3.77E-32
CNVR_2778	chrAC_000178.1	56521867	56531190	9324	-2.3006	1.43E-221
CNVR_2779	chrAC_000178.1	56861527	56869962	8436	-3.5426	2.54E-270
CNVR_2780	chrAC_000178.1	58039903	58042122	2220	-1.6717	5.40E-39
CNVR_2781	chrAC_000178.1	62290315	62293422	3108	-1.7106	2.30E-55
CNVR_2782	chrAC_000178.1	62300971	62302746	1776	-1.2492	8.69E-22
CNVR_2783	chrAC_000178.1	66962527	66965190	2664	-2.5317	4.05E-70
CNVR_2784	chrAC_000178.1	67166323	67168098	1776	-4.7307	7.33E-65
CNVR_2785	chrAC_000178.1	71507755	71514414	6660	-1.1992	1.41E-72
CNVR_2786	chrAC_000178.1	71515747	71517966	2220	-1.2966	3.11E-28
CNVR_2787	chrAC_000178.1	71519743	71521962	2220	-1.0996	2.13E-22
CNVR_2788	chrAC_000178.1	71584567	71586786	2220	-1.1480	7.94E-24
CNVR_2789	chrAC_000167.1	22397803	22399578	1776	1.1671	7.88E-23
CNVR_2790	chrAC_000167.1	22400023	22403130	3108	1.2596	1.74E-43
CNVR_2791	chrAC_000167.1	23166367	23169030	2664	1.3743	1.23E-42
CNVR_2792	chrAC_000167.1	23183239	23188566	5328	1.5427	2.63E-98
CNVR_2793	chrAC_000167.1	23195227	23197002	1776	1.2661	9.10E-26
CNVR_2794	chrAC_000167.1	23210323	23213430	3108	1.5113	1.60E-56
CNVR_2795	chrAC_000167.1	23218759	23220534	1776	1.1573	1.53E-22
CNVR_2796	chrAC_000167.1	23222755	23226306	3552	1.3939	2.02E-57
CNVR_2797	chrAC_000167.1	23267599	23269374	1776	1.3337	8.78E-28
CNVR_2798	chrAC_000167.1	23283139	23285802	2664	1.8032	4.27E-61
CNVR_2799	chrAC_000167.1	23372383	23374602	2220	1.2010	2.22E-29
CNVR_2800	chrAC_000167.1	23378155	23380374	2220	1.3496	6.72E-35
CNVR_2801	chrAC_000167.1	23427883	23430546	2664	1.1766	7.70E-34
CNVR_2802	chrAC_000167.1	23438539	23440314	1776	1.2306	1.04E-24
CNVR_2803	chrAC_000167.1	23463403	23466066	2664	1.6576	4.94E-55
CNVR_2804	chrAC_000167.1	23474503	23476278	1776	1.1728	5.34E-23
CNVR_2805	chrAC_000167.1	23486491	23488710	2220	1.3074	2.50E-33
CNVR_2806	chrAC_000167.1	23602375	23604150	1776	1.0724	4.64E-20
CNVR_2807	chrAC_000167.1	23722699	23726250	3552	1.3731	3.45E-56
CNVR_2808	chrAC_000167.1	24389587	24391806	2220	1.7810	1.86E-50
CNVR_2809	chrAC_000167.1	24400243	24402018	1776	1.2095	4.39E-24
CNVR_2810	chrAC_000167.1	24873547	24875322	1776	1.3125	3.77E-27
CNVR_2811	chrAC_000167.1	27380371	27382146	1776	1.5214	2.43E-33
CNVR_2812	chrAC_000167.1	27955351	27957126	1776	1.5204	2.61E-33

CNVR_2813	chrAC_000167.1	27959347	27964230	4884	1.5182	2.62E-88
CNVR_2814	chrAC_000167.1	35644987	35646762	1776	1.5826	4.01E-35
CNVR_2815	chrAC_000167.1	35647651	35649426	1776	1.5762	6.15E-35
CNVR_2816	chrAC_000167.1	41781067	41782842	1776	1.4261	1.57E-30
CNVR_2817	chrAC_000167.1	68737639	68739858	2220	1.4031	6.87E-37
CNVR_2818	chrAC_000167.1	67711	69486	1776	-1.1699	7.38E-20
CNVR_2819	chrAC_000167.1	6665551	6667770	2220	-2.2452	6.42E-53
CNVR_2820	chrAC_000167.1	9184363	9186582	2220	-1.2112	1.21E-25
CNVR_2821	chrAC_000167.1	9188803	9191022	2220	-1.3933	5.24E-31
CNVR_2822	chrAC_000167.1	9194131	9195906	1776	-1.4243	7.34E-26
CNVR_2823	chrAC_000167.1	22446199	22448862	2664	-3.2065	4.00E-82
CNVR_2824	chrAC_000167.1	22715263	22718814	3552	-1.7537	1.05E-64
CNVR_2825	chrAC_000167.1	22721923	22724142	2220	-2.0509	1.46E-48
CNVR_2826	chrAC_000167.1	22725475	22729470	3996	-2.0550	4.10E-86
CNVR_2827	chrAC_000167.1	22730359	22734354	3996	-2.0509	6.06E-86
CNVR_2828	chrAC_000167.1	22741015	22746342	5328	-1.9206	2.13E-106
CNVR_2829	chrAC_000167.1	22772983	22779642	6660	-2.6108	3.52E-176
CNVR_2830	chrAC_000167.1	22784971	22787190	2220	-1.7614	2.57E-41
CNVR_2831	chrAC_000167.1	22791187	22795626	4440	-1.3273	2.03E-56
CNVR_2832	chrAC_000167.1	22799623	22801398	1776	-2.5570	1.33E-47
CNVR_2833	chrAC_000167.1	23301787	23304450	2664	-3.2972	9.76E-104
CNVR_2834	chrAC_000167.1	23311111	23314218	3108	-2.3518	1.88E-76
CNVR_2835	chrAC_000167.1	23668975	23672970	3996	-2.3874	4.98E-99
CNVR_2836	chrAC_000167.1	23673415	23675634	2220	-2.7367	3.98E-62
CNVR_2837	chrAC_000167.1	23676523	23680518	3996	-1.8131	2.29E-75
CNVR_2838	chrAC_000167.1	23686735	23694726	7992	-1.9142	4.96E-158
CNVR_2839	chrAC_000167.1	23695171	23701830	6660	-2.3344	1.54E-160
CNVR_2840	chrAC_000167.1	23711599	23714706	3108	-1.4782	3.64E-46
CNVR_2841	chrAC_000167.1	23815495	23818158	2664	-1.5350	9.12E-42
CNVR_2842	chrAC_000167.1	23824819	23827482	2664	-1.6069	3.45E-44
CNVR_2843	chrAC_000167.1	23827927	23831922	3996	-1.6323	1.19E-66
CNVR_2844	chrAC_000167.1	23834143	23839470	5328	-2.1890	1.84E-121
CNVR_2845	chrAC_000167.1	23842135	23845242	3108	-2.0662	9.22E-68
CNVR_2846	chrAC_000167.1	23845687	23849238	3552	-2.5454	4.97E-93
CNVR_2847	chrAC_000167.1	23850127	23855898	5772	-2.0176	3.12E-121
CNVR_2848	chrAC_000167.1	23859451	23866110	6660	-2.3169	1.81E-159
CNVR_2849	chrAC_000167.1	23866999	23878098	11100	-2.3596	1.01E-268
CNVR_2850	chrAC_000167.1	23941147	23947362	6216	-2.0423	4.82E-132
CNVR_2851	chrAC_000167.1	23949583	23953134	3552	-3.0388	1.97E-105
CNVR_2852	chrAC_000167.1	23954911	23958462	3552	-1.8302	6.59E-68
CNVR_2853	chrAC_000167.1	24027283	24029058	1776	-2.1125	2.42E-40
CNVR_2854	chrAC_000167.1	24029947	24033942	3996	-1.6477	2.07E-67
CNVR_2855	chrAC_000167.1	24035275	24041046	5772	-2.3821	6.77E-142
CNVR_2856	chrAC_000167.1	24092995	24094770	1776	-1.2972	7.10E-23

CNVR_2857	chrAC_000167.1	24224419	24227526	3108	-1.3942	9.17E-43
CNVR_2858	chrAC_000167.1	24247951	24250170	2220	-2.7537	2.11E-62
CNVR_2859	chrAC_000167.1	24330979	24338970	7992	-2.4896	3.98E-203
CNVR_2860	chrAC_000167.1	24339415	24341634	2220	-2.6205	3.55E-60
CNVR_2861	chrAC_000167.1	24346519	24354954	8436	-1.8952	6.75E-165
CNVR_2862	chrAC_000167.1	24472171	24473946	1776	-1.6403	9.03E-31
CNVR_2863	chrAC_000167.1	24475279	24477942	2664	-2.0992	2.93E-59
CNVR_2864	chrAC_000167.1	24478387	24483270	4884	-2.1702	1.35E-110
CNVR_2865	chrAC_000167.1	25054255	25056474	2220	-2.1111	5.92E-50
CNVR_2866	chrAC_000167.1	25108867	25111086	2220	-1.1539	5.97E-24
CNVR_2867	chrAC_000167.1	25153267	25155486	2220	-1.4521	1.02E-32
CNVR_2868	chrAC_000167.1	25165255	25167918	2664	-1.4879	3.73E-40
CNVR_2869	chrAC_000167.1	25169251	25172802	3552	-2.4844	3.13E-91
CNVR_2870	chrAC_000167.1	25200331	25202106	1776	-1.1177	1.27E-18
CNVR_2871	chrAC_000167.1	26583391	26586054	2664	-1.1952	7.11E-30
CNVR_2872	chrAC_000167.1	26913727	26915502	1776	-3.9640	3.62E-61
CNVR_2873	chrAC_000167.1	27039379	27041154	1776	-1.4658	7.99E-27
CNVR_2874	chrAC_000167.1	27108643	27117522	8880	-1.7483	2.61E-158
CNVR_2875	chrAC_000167.1	27635227	27660534	25308	-3.7921	0.00E+00
CNVR_2876	chrAC_000167.1	28133839	28135614	1776	-1.6663	2.42E-31
CNVR_2877	chrAC_000167.1	28136947	28139166	2220	-5.1961	3.74E-82
CNVR_2878	chrAC_000167.1	32138719	32140494	1776	-2.0288	8.80E-39
CNVR_2879	chrAC_000167.1	32957011	32959230	2220	-2.0426	2.29E-48
CNVR_2880	chrAC_000167.1	33192331	33194550	2220	-1.1610	3.69E-24
CNVR_2881	chrAC_000167.1	37497799	37500018	2220	-2.6960	1.85E-61
CNVR_2882	chrAC_000167.1	38339179	38340954	1776	-1.3482	4.43E-24
CNVR_2883	chrAC_000167.1	39005179	39007398	2220	-1.2840	8.49E-28
CNVR_2884	chrAC_000167.1	50285887	50288550	2664	-2.0413	1.21E-57
CNVR_2885	chrAC_000167.1	52249699	52251474	1776	-1.5486	1.02E-28
CNVR_2886	chrAC_000167.1	54574483	54576258	1776	-1.3312	1.11E-23
CNVR_2887	chrAC_000167.1	60192859	60195522	2664	-2.7643	1.03E-74
CNVR_2888	chrAC_000167.1	60206623	60211950	5328	-1.9882	2.28E-110
CNVR_2889	chrAC_000167.1	63653839	63655614	1776	-1.4621	9.73E-27
CNVR_2890	chrAC_000167.1	64655059	64656834	1776	-1.1588	1.36E-19
CNVR_2891	chrAC_000167.1	75139231	75141006	1776	-1.1445	2.96E-19
CNVR_2892	chrAC_000167.1	78284083	78286302	2220	-2.0211	7.42E-48
CNVR_2893	chrAC_000167.1	79252891	79255554	2664	-1.6722	2.39E-46
CNVR_2894	chrAC_000167.1	80775367	80778030	2664	-1.6039	4.34E-44
CNVR_2895	chrAC_000167.1	81406735	81408510	1776	-5.4212	1.25E-66
CNVR_2896	chrAC_000167.1	81410287	81412062	1776	-2.0120	1.84E-38
CNVR_2897	chrAC_000167.1	84189727	84191502	1776	-1.3738	1.11E-24
CNVR_2898	chrAC_000167.1	84324703	84327366	2664	-1.6761	1.78E-46
CNVR_2899	chrAC_000167.1	84330919	84332694	1776	-1.8267	9.14E-35
CNVR_2900	chrAC_000167.1	88565791	88570674	4884	-2.2395	6.53E-114

CNVR_2901	chrAC_000167.1	90935863	90938082	2220	-2.3234	1.48E-54
CNVR_2902	chrAC_000167.1	95350555	95353218	2664	-2.3205	4.96E-65
CNVR_2903	chrAC_000167.1	96034759	96036978	2220	-2.2165	2.67E-52
CNVR_2904	chrAC_000167.1	96297163	96298938	1776	-1.2512	8.70E-22
CNVR_2905	chrAC_000167.1	96710971	96720294	9324	-3.9808	0.00E+00
CNVR_2906	chrAC_000167.1	99150307	99152082	1776	-1.4647	8.46E-27
CNVR_2907	chrAC_000167.1	104227003	104229222	2220	-4.3919	9.76E-79
