# Supplementary material for: Whole-genome sequencing reveals mutational landscape underlying phenotypic differences between two widespread Chinese cattle breeds
Source: PLoS One. 2017 Aug 25;12(8):e0183921. doi: 10.1371/journal.pone.0183921 (PMC5571935; doi:10.1371/journal.pone.0183921)
Supplement: S6 Table — (PDF) [file pone.0183921.s012.pdf]

**S6 Table.** The CNVs in our study were identical or overlapped with those reported in previous papers.

| CNVs information in our study | | | | | | The study reported in previous papers | | | | | | |
|---|---|---|---|---|---|---|---|---|---|---|---|---|
| **Chr.** | **CNVR_ID** | **Start** | **End** | **Size** | **CNV_type** | **Chr.** | **CNVR_ID** | **Start** | **End** | **Size** | **CNV_type** | **Previous study** |
| Chr3 | CNVR_1736 | 8616931 | 8665326 | 48396 | Loss | Chr3 | CNVR#83 | 8621927 | 8642213 | 20286 | Gain | Bickhart et al. 2012 |
| Chr3 | CNVR_1737 | 8665771 | 8668878 | 3108 | Loss | Chr3 | CNVR#84 | 8653927 | 8666779 | 12852 | Gain | Bickhart et al. 2012 |
| Chr3 | CNVR_1755 | 22626907 | 22634454 | 7548 | Loss | Chr3 | CNVR#110 | 22614961 | 22701627 | 86666 | Gain | Bickhart et al. 2012 |
| Chr4 | CNVR_1925 | 109511491 | 109514598 | 3108 | Loss | Chr4 | CNVR#184 | 109419476 | 109528496 | 109020 | Gain | Bickhart et al. 2012 |
| Chr4 | CNVR_1926 | 109613611 | 109615386 | 1776 | Loss | Chr4 | CNVR#185 | 109611853 | 109691354 | 79501 | Gain | Bickhart et al. 2012 |
| Chr4 | CNVR_1927 | 109799203 | 109800978 | 1776 | Loss | Chr4 | CNVR#186 | 109766052 | 109847305 | 81253 | Gain | Bickhart et al. 2012 |
| Chr4 | CNVR_1928 | 109813855 | 109816074 | 2220 | Loss | | | | | | | |
| Chr5 | CNVR_1483 | 85471 | 87690 | 2220 | Loss | Chr5 | CNVR#200 | 75927 | 295096 | 219169 | Gain | Bickhart et al. 2012 |
| Chr5 | CNVR_1504 | 10284151 | 10287702 | 3552 | Loss | Chr5 | CNVR#207 | 10274349 | 10302404 | 28055 | Gain | Bickhart et al. 2012 |
| Chr5 | CNVR_1537 | 58020811 | 58070982 | 50172 | Loss | Chr5 | CNVR#232 | 58016389 | 58031389 | 15000 | Gain | Bickhart et al. 2012 |
| Chr5 | CNVR_1561 | 63466027 | 63467802 | 1776 | Loss | Chr5 | CNVR#237 | 63445770 | 63520990 | 75220 | Gain | Bickhart et al. 2012 |
| Chr5 | CNVR_1562 | 63501547 | 63504654 | 3108 | Loss | | | | | | | |
| Chr5 | CNVR_1457 | 79951303 | 79953078 | 1776 | Gain | Chr5 | CNVR#248 | 79899256 | 80001248 | 101992 | Gain | Bickhart et al. 2012 |
| Chr6 | CNVR_2301 | 118421683 | 118423458 | 1776 | Loss | Chr6 | CNVR#302 | 118388105 | 118427501 | 39396 | Gain | Bickhart et al. 2012 |
| Chr7 | CNVR_82 | 7989115 | 7991334 | 2220 | Loss | Chr7 | CNVR#316 | 7702569 | 8091292 | 388723 | Gain | Bickhart et al. 2012 |
| Chr7 | CNVR_100 | 11790199 | 11794638 | 4440 | Loss | Chr7 | CNVR#324 | 11794149 | 11819458 | 25309 | Gain | Bickhart et al. 2012 |
| Chr7 | CNVR_101 | 11799523 | 11802186 | 2664 | Loss | | | | | | | |
| Chr7 | CNVR_104 | 11815063 | 11822610 | 7548 | Loss | | | | | | | |
| Chr7 | CNVR_102 | 11803519 | 11807070 | 3552 | Loss | Chr7 | Chr7_CNVR_17 | 11805366 | 11808976 | 3611 | Gain | Stothard et al. 2011 |
| Chr7 | CNVR_103 | 11811067 | 11812842 | 1776 | Loss | Chr7 | Chr7_CNVR_18 | 11809926 | 11812206 | 2281 | Gain | Stothard et al. 2011 |
| Chr8 | CNVR_2562 | 68413075 | 68414850 | 1776 | Loss | Chr8 | CNVR#396 | 68408007 | 68439400 | 31393 | Gain | Bickhart et al. 2012 |
| Chr8 | CNVR_2577 | 88754935 | 88757154 | 2220 | Loss | Chr8 | CNVR#406 | 88747611 | 88760099 | 12488 | Gain | Bickhart et al. 2012 |
| Chr10 | CNVR_2824 | 22715263 | 22718814 | 3552 | Loss | Chr10 | Chr10_CNVR_8 | 22714304 | 22717130 | 2827 | Loss-Gain | Stothard et al. 2011 |

| | | | | | | | | | | | | |
|---|---|---|---|---|---|---|---|---|---|---|---|---|
| Chr10 | CNVR_2791 | 23166367 | 23169030 | 2664 | Gain | Chr10 | CNVR#449 | 23064651 | 23178443 | 113792 | Gain | Bickhart et al. 2012 |
| Chr10 | CNVR_2799 | 23372383 | 23374602 | 2220 | Gain | Chr10 | CNVR#450 | 23348269 | 23421686 | 73417 | Gain | Bickhart et al. 2012 |
| Chr10 | CNVR_2800 | 23378155 | 23380374 | 2220 | Gain | | | | | | | |
| Chr10 | CNVR_2801 | 23427883 | 23430546 | 2664 | Gain | | | | | | | |
| Chr10 | CNVR_2802 | 23438539 | 23440314 | 1776 | Gain | | | | | | | |
| Chr10 | CNVR_2803 | 23463403 | 23466066 | 2664 | Gain | | | | | | | |
| Chr10 | CNVR_2804 | 23474503 | 23476278 | 1776 | Gain | | | | | | | |
| Chr10 | CNVR_2805 | 23486491 | 23488710 | 2220 | Gain | | | | | | | |
| Chr10 | CNVR_2806 | 23602375 | 23604150 | 1776 | Gain | | | | | | | |
| Chr10 | CNVR_2835 | 23668975 | 23672970 | 3996 | Loss | Chr10 | CNVR#451 | 23426684 | 23725793 | 299109 | Gain | Bickhart et al. 2012 |
| Chr10 | CNVR_2836 | 23673415 | 23675634 | 2220 | Loss | | | | | | | |
| Chr10 | CNVR_2837 | 23676523 | 23680518 | 3996 | Loss | | | | | | | |
| Chr10 | CNVR_2838 | 23686735 | 23694726 | 7992 | Loss | | | | | | | |
| Chr10 | CNVR_2839 | 23695171 | 23701830 | 6660 | Loss | | | | | | | |
| Chr10 | CNVR_2840 | 23711599 | 23714706 | 3108 | Loss | | | | | | | |
| Chr10 | CNVR_2807 | 23722699 | 23726250 | 3552 | Gain | | | | | | | |
| Chr10 | CNVR_2853 | 24027283 | 24029058 | 1776 | Loss | | | | | | | |
| Chr10 | CNVR_2854 | 24029947 | 24033942 | 3996 | Loss | | | | | | | |
| Chr10 | CNVR_2855 | 24035275 | 24041046 | 5772 | Loss | Chr10 | CNVR#453 | 23974385 | 24245971 | 271586 | Gain | Bickhart et al. 2012 |
| Chr10 | CNVR_2856 | 24092995 | 24094770 | 1776 | Loss | | | | | | | |
| Chr10 | CNVR_2857 | 24224419 | 24227526 | 3108 | Loss | | | | | | | |
| Chr10 | CNVR_2859 | 24330979 | 24338970 | 7992 | Loss | Chr10 | CNVR#454 | 24307765 | 24352110 | 44345 | Gain | Bickhart et al. 2012 |
| Chr10 | CNVR_2860 | 24339415 | 24341634 | 2220 | Loss | | | | | | | |
| Chr10 | CNVR_2872 | 26913727 | 26915502 | 1776 | Loss | Chr10 | CNVR#464 | 26876031 | 26935294 | 59263 | Gain | Bickhart et al. 2012 |
| Chr11 | CNVR_513 | 24326983 | 24328758 | 1776 | Loss | Chr11 | Chr11_CNVR_6 | 24327564 | 24329812 | 2249 | Loss-Gain | Stothard et al. 2011 |
| Chr12 | CNVR_1031 | 70472791 | 70475454 | 2664 | Gain | Chr12 | Chr12_CNVR_45 | 70470916 | 70473696 | 2781 | Loss | Stothard et al. 2011 |
| | | | | | | Chr12 | CNVR#551 | 70447425 | 70520787 | 73362 | Gain | Bickhart et al. 2012 |

| | | | | | | | | | | | | |
|---|---|---|---|---|---|---|---|---|---|---|---|---|
| Chr13 | CNVR_1397 | 16747015 | 16748790 | 1776 | Loss | Chr13 | CNVR#562 | 16567986 | 16767413 | 199427 | Gain | Bickhart et al. 2012 |
| Chr15 | CNVR_408 | 5266507 | 5268282 | 1776 | Loss | Chr15 | CNVR#620 | 5256608 | 5286627 | 30019 | Gain | Bickhart et al. 2012 |
| Chr15 | CNVR_433 | 46572715 | 46574490 | 1776 | Loss | Chr15 | CNVR#644 | 46574147 | 46597560 | 23413 | Gain | Bickhart et al. 2012 |
| Chr15 | CNVR_434 | 46768075 | 46769850 | 1776 | Loss | Chr15 | CNVR#646 | 46751372 | 46774825 | 23453 | Gain | Bickhart et al. 2012 |
| Chr15 | CNVR_435 | 46774291 | 46776954 | 2664 | Loss | | | | | | | |
| Chr15 | CNVR_437 | 46807591 | 46809366 | 1776 | Loss | Chr15 | CNVR#647 | 46807189 | 46916764 | 109575 | Gain | Bickhart et al. 2012 |
| Chr15 | CNVR_438 | 46824019 | 46825794 | 1776 | Loss | | | | | | | |
| Chr15 | CNVR_439 | 46827127 | 46835118 | 7992 | Loss | | | | | | | |
| Chr15 | CNVR_440 | 46836007 | 46837782 | 1776 | Loss | | | | | | | |
| Chr15 | CNVR_441 | 46844443 | 46846218 | 1776 | Loss | | | | | | | |
| Chr15 | CNVR_442 | 46849771 | 46851546 | 1776 | Loss | | | | | | | |
| Chr15 | CNVR_443 | 46862647 | 46866198 | 3552 | Loss | | | | | | | |
| Chr15 | CNVR_444 | 46875079 | 46877298 | 2220 | Loss | | | | | | | |
| Chr15 | CNVR_445 | 46877743 | 46892838 | 15096 | Loss | | | | | | | |
| Chr15 | CNVR_446 | 46895503 | 46908822 | 13320 | Loss | | | | | | | |
| Chr15 | CNVR_447 | 46909711 | 46922142 | 12432 | Loss | | | | | | | |
| Chr15 | CNVR_453 | 47958439 | 47961546 | 3108 | Loss | Chr15 | CNVR#651 | 47924154 | 48044883 | 120729 | Gain | Bickhart et al. 2012 |
| Chr15 | CNVR_387 | 47991295 | 47993070 | 1776 | Gain | | | | | | | |
| Chr15 | CNVR_388 | 48011275 | 48013494 | 2220 | Gain | | | | | | | |
| Chr15 | CNVR_454 | 48026815 | 48028590 | 1776 | Loss | | | | | | | |
| Chr15 | CNVR_389 | 48072991 | 48075210 | 2220 | Gain | Chr15 | CNVR#652 | 48064280 | 48100629 | 36349 | Gain | Bickhart et al. 2012 |
| Chr15 | CNVR_390 | 48467263 | 48469482 | 2220 | Gain | Chr15 | CNVR#655 | 48408857 | 48517771 | 108914 | Gain | Bickhart et al. 2012 |
| Chr15 | CNVR_487 | 79717315 | 79720422 | 3108 | Loss | Chr15 | CNVR#681 | 79641690 | 79749838 | 108148 | Gain | Bickhart et al. 2012 |
| Chr15 | CNVR_494 | 82859059 | 82861278 | 2220 | Loss | Chr15 | CNVR#690 | 82826436 | 82894108 | 67672 | Gain | Bickhart et al. 2012 |
| Chr18 | CNVR_245 | 57565711 | 57571926 | 6216 | Loss | Chr18 | CNVR#787 | 57552509 | 57565871 | 13362 | Gain | Bickhart et al. 2012 |
| Chr18 | CNVR_246 | 58569151 | 58571814 | 2664 | Loss | Chr18 | CNVR#793 | 58474489 | 58618798 | 144309 | Gain | Bickhart et al. 2012 |
| Chr18 | CNVR_247 | 59650291 | 59652510 | 2220 | Loss | Chr18 | CNVR#800 | 59483194 | 59780268 | 297074 | Gain | Bickhart et al. 2012 |

| | | | | | | | | | | | | |
|---|---|---|---|---|---|---|---|---|---|---|---|---|
| Chr18 | CNVR_248 | 60529411 | 60531186 | 1776 | Loss | | | | | | | |
| Chr18 | CNVR_249 | 60532519 | 60541398 | 8880 | Loss | Chr18 | CNVR#803 | 60334628 | 60563285 | 228657 | Gain | Bickhart et al. 2012 |
| Chr18 | CNVR_250 | 60554719 | 60559158 | 4440 | Loss | | | | | | | |
| Chr18 | CNVR_182 | 61549279 | 61551054 | 1776 | Gain | Chr18 | CNVR#813 | 61530651 | 61631780 | 101129 | Gain | Bickhart et al. 2012 |
| Chr18 | CNVR_256 | 61620763 | 61622982 | 2220 | Loss | | | | | | | |
| Chr18 | CNVR_183 | 61662943 | 61664718 | 1776 | Gain | Chr18 | Chr18_CNVR_80 | 61662496 | 61666562 | 4067 | Gain | Stothard et al. 2011 |
| Chr18 | CNVR_184 | 61666495 | 61675374 | 8880 | Gain | | | | | | | |
| Chr18 | CNVR_194 | 61826335 | 61831662 | 5328 | Gain | Chr18 | Chr18_CNVR_84 | 61825136 | 61828988 | 3853 | Gain | Stothard et al. 2011 |
| | | | | | | Chr18 | Chr18_CNVR_85 | 61829844 | 61833054 | 3211 | Gain | Stothard et al. 2011 |
| Chr18 | CNVR_195 | 61868071 | 61872954 | 4884 | Gain | Chr18 | Chr18_CNVR_90 | 61867936 | 61870932 | 2997 | Gain | Stothard et al. 2011 |
| Chr18 | CNVR_196 | 61874731 | 61876506 | 1776 | Gain | | | | | | | |
| Chr18 | CNVR_197 | 61883167 | 61884942 | 1776 | Gain | Chr18 | CNVR#814 | 61820264 | 61889686 | 69422 | Gain | Bickhart et al. 2012 |
| Chr18 | CNVR_198 | 61888495 | 61891602 | 3108 | Gain | | | | | | | |
| Chr18 | CNVR_258 | 63224491 | 63226266 | 1776 | Loss | | | | | | | |
| Chr18 | CNVR_259 | 63235147 | 63237366 | 2220 | Loss | Chr18 | CNVR#820 | 63204974 | 63315699 | 110725 | Gain | Bickhart et al. 2012 |
| Chr18 | CNVR_260 | 63237811 | 63245358 | 7548 | Loss | | | | | | | |
| Chr18 | CNVR_261 | 63259123 | 63260898 | 1776 | Loss | Chr18 | Chr18_CNVR_112 | 63252302 | 63260006 | 7705 | Loss | Stothard et al. 2011 |
| Chr18 | CNVR_262 | 63341707 | 63343482 | 1776 | Loss | Chr18 | CNVR#821 | 63320697 | 63378137 | 57440 | Gain | Bickhart et al. 2012 |
| Chr18 | CNVR_204 | 63508651 | 63510870 | 2220 | Loss | Chr18 | CNVR#822 | 63394536 | 63513292 | 118756 | Gain | Bickhart et al. 2012 |
| Chr23 | CNVR_1659 | 26562967 | 26564742 | 1776 | Loss | Chr23 | CNVR#961 | 26549733 | 26630308 | 80575 | Gain | Bickhart et al. 2012 |
| Chr23 | CNVR_1660 | 26604703 | 26606922 | 2220 | Loss | | | | | | | |
| Chr23 | CNVR_1623 | 28496587 | 28498362 | 1776 | Gain | Chr23 | CNVR#968 | 28462837 | 28523990 | 61153 | Gain | Bickhart et al. 2012 |
| Chr23 | CNVR_1664 | 29720251 | 29722470 | 2220 | Loss | Chr23 | CNVR#975 | 29726131 | 29750759 | 24628 | Gain | Bickhart et al. 2012 |
| Chr23 | CNVR_1665 | 29739343 | 29742006 | 2664 | Loss | | | | | | | |
| Chr24 | CNVR_2697 | 12211 | 26862 | 14652 | Loss | Chr24 | CNVR#988 | 15000 | 33298 | 18298 | Gain | Bickhart et al. 2012 |
| Chr24 | CNVR_2698 | 31303 | 33966 | 2664 | Loss | | | | | | | |
| Chr24 | CNVR_2704 | 13163047 | 13166154 | 3108 | Loss | Chr24 | CNVR#990 | 13160364 | 13182431 | 22067 | Gain | Bickhart et al. 2012 |

| | | | | | | | | | | | | |
|---|---|---|---|---|---|---|---|---|---|---|---|---|
| Chr27 | CNVR_1705 | 11618815 | 11620590 | 1776 | Loss | Chr27 | Chr27_CNVR_11 | 11617743 | 11624879 | 7137 | Gain | Stothard et al. 2011 |
| | | | | | | Chr27 | CNVR#1060 | 11579904 | 11622517 | 42613 | Gain | Bickhart et al. 2012 |
| Chr27 | CNVR_1679 | 28929931 | 28931706 | 1776 | Gain | Chr27 | CNVR#1064 | 28921875 | 28938820 | 16945 | Gain | Bickhart et al. 2012 |
| Chr29 | CNVR_2316 | 5493391 | 5496054 | 2664 | Loss | | | | | | | |
| Chr29 | CNVR_2317 | 5501827 | 5503602 | 1776 | Loss | | | | | | | |
| Chr29 | CNVR_2318 | 5521807 | 5524914 | 3108 | Loss | | | | | | | |
| Chr29 | CNVR_2319 | 5525803 | 5531574 | 5772 | Loss | Chr29 | CNVR#1080 | 5494956 | 5558948 | 63992 | Gain | Bickhart et al. 2012 |
| Chr29 | CNVR_2320 | 5540455 | 5542230 | 1776 | Loss | | | | | | | |
| Chr29 | CNVR_2321 | 5544451 | 5547114 | 2664 | Loss | | | | | | | |
| Chr29 | CNVR_2322 | 5549335 | 5577750 | 28416 | Loss | | | | | | | |
| Chr29 | CNVR_2323 | 5601727 | 5616378 | 14652 | Loss | | | | | | | |
| Chr29 | CNVR_2324 | 5616823 | 5619486 | 2664 | Loss | | | | | | | |
| Chr29 | CNVR_2325 | 5622151 | 5624370 | 2220 | Loss | | | | | | | |
| Chr29 | CNVR_2326 | 5678095 | 5680758 | 2664 | Loss | Chr29 | CNVR#1081 | 5560573 | 5702154 | 141581 | Gain | Bickhart et al. 2012 |
| Chr29 | CNVR_2327 | 5685643 | 5687418 | 1776 | Loss | | | | | | | |
| Chr29 | CNVR_2328 | 5692303 | 5695410 | 3108 | Loss | | | | | | | |
| Chr29 | CNVR_2329 | 5695855 | 5700738 | 4884 | Loss | | | | | | | |
| Chr29 | CNVR_2330 | 5708287 | 5710062 | 1776 | Loss | | | | | | | |
| Chr29 | CNVR_2331 | 5714947 | 5717610 | 2664 | Loss | | | | | | | |
| Chr29 | CNVR_2332 | 5725159 | 5727378 | 2220 | Loss | | | | | | | |
| Chr29 | CNVR_2333 | 5733595 | 5741142 | 7548 | Loss | | | | | | | |
| Chr29 | CNVR_2334 | 5758015 | 5759790 | 1776 | Loss | Chr29 | CNVR#1082 | 5707152 | 5814271 | 107119 | Gain | Bickhart et al. 2012 |
| Chr29 | CNVR_2335 | 5762455 | 5764674 | 2220 | Loss | | | | | | | |
| Chr29 | CNVR_2336 | 5777107 | 5778882 | 1776 | Loss | | | | | | | |
| Chr29 | CNVR_2337 | 5781991 | 5791758 | 9768 | Loss | | | | | | | |
| Chr29 | CNVR_2338 | 5792647 | 5794866 | 2220 | Loss | | | | | | | |
| Chr29 | CNVR_2364 | 29472943 | 29475162 | 2220 | Loss | Chr29 | CNVR#1098 | 29403271 | 29480744 | 77473 | Gain | Bickhart et al. 2012 |

| | | | | | | | | | | | | |
|---|---|---|---|---|---|---|---|---|---|---|---|---|
| Chr29 | CNVR_2365 | 29475607 | 29477826 | 2220 | Loss | | | | | | | |
| Chr29 | CNVR_2377 | 49294435 | 49296210 | 1776 | Loss | Chr29 | CNVR#1117 | 49295365 | 49330661 | 35296 | Gain | Bickhart et al. 2012 |
| ChrX | CNVR_587 | 19530895 | 19532670 | 1776 | Loss | ChrX | CNVR#1139 | 19530862 | 19562486 | 31624 | Gain | Bickhart et al. 2012 |
| ChrX | CNVR_592 | 24622243 | 24624018 | 1776 | Loss | ChrX | CNVR#1150 | 24522210 | 24762267 | 240057 | Gain | Bickhart et al. 2012 |
| ChrX | CNVR_548 | 24626239 | 24628014 | 1776 | Gain | | | | | | | |
| ChrX | CNVR_610 | 35516227 | 35564178 | 47952 | Loss | ChrX | CNVR#1167 | 35508447 | 35535366 | 26919 | Gain | Bickhart et al. 2012 |
| ChrX | CNVR_712 | 36939247 | 36941910 | 2664 | Loss | ChrX | CNVR#1169 | 36926571 | 36964578 | 38007 | Gain | Bickhart et al. 2012 |
| ChrX | CNVR_550 | 36957451 | 36959226 | 1776 | Gain | | | | | | | |
| ChrX | CNVR_714 | 37031155 | 37032930 | 1776 | Loss | ChrX | CNVR#1171 | 36999585 | 37210755 | 211170 | Gain | Bickhart et al. 2012 |
| ChrX | CNVR_715 | 37035151 | 37036926 | 1776 | Loss | | | | | | | |
| ChrX | CNVR_716 | 37052911 | 37054686 | 1776 | Loss | | | | | | | |
| ChrX | CNVR_717 | 37061347 | 37063122 | 1776 | Loss | | | | | | | |
| ChrX | CNVR_718 | 37092871 | 37095090 | 2220 | Loss | | | | | | | |
| ChrX | CNVR_719 | 37139491 | 37141266 | 1776 | Loss | | | | | | | |
| ChrX | CNVR_720 | 37146151 | 37151478 | 5328 | Loss | | | | | | | |
| ChrX | CNVR_721 | 37159915 | 37162134 | 2220 | Loss | | | | | | | |
| ChrX | CNVR_722 | 37164355 | 37167462 | 3108 | Loss | | | | | | | |
| ChrX | CNVR_723 | 37242055 | 37244274 | 2220 | Loss | ChrX | CNVR#1172 | 37232409 | 37256013 | 23604 | Gain | Bickhart et al. 2012 |
| ChrX | CNVR_724 | 37246051 | 37248714 | 2664 | Loss | | | | | | | |
| ChrX | CNVR_746 | 44566279 | 44568498 | 2220 | Loss | ChrX | CNVR#1182 | 44534006 | 44587958 | 53952 | Gain | Bickhart et al. 2012 |
| ChrX | CNVR_890 | 71011807 | 71013582 | 1776 | Loss | ChrX | CNVR#1218 | 70941827 | 71155280 | 213453 | Gain | Bickhart et al. 2012 |
| ChrX | CNVR_901 | 77982607 | 77984382 | 1776 | Loss | ChrX | CNVR#1228 | 77985141 | 78006193 | 21052 | Gain | Bickhart et al. 2012 |
| ChrX | CNVR_907 | 85932871 | 85934646 | 1776 | Loss | ChrX | CNVR#1247 | 85886230 | 85978715 | 92485 | Gain | Bickhart et al. 2012 |
