# Supplementary material for: Whole-genome sequencing reveals mutational landscape underlying phenotypic differences between two widespread Chinese cattle breeds
Source: PLoS One. 2017 Aug 25;12(8):e0183921. doi: 10.1371/journal.pone.0183921 (PMC5571935; doi:10.1371/journal.pone.0183921)
Supplement: S7 Table — A total of 20 primer pairs were used for validation in mRNA level, and 2 primer pairs were used for detection in DNA level. (PDF) [file pone.0183921.s013.pdf]

S7 Table. The information of primers used in this study. A total of 20 primer pairs were used for validation in mRNA level, and 2 primer pairs were used for detection in DNA level.

	Locus	Primer sequences (5' to 3')	Amplification length (bp)
DNA level	Chr2_CNV_2113 (*ACTL8*)	1F: AATCGCCACAACCTGTTCCA	109
		1R: CCGGCAGGTTCTGGTAGAAG	
	Chr2_CNV_2062 (*RAPH1*)	2F: TGGTTCCACATTCCGTAG	107
		2R: CCTTCGTGTTGACTTCATT	
	Chr5_CNV_1452 (*LOC617219*)	3F: CATCCTGCTGATCCTGAA	112
		3R: AACTCATCTGCCTCTGAAT	
	Chr21_CNV_2724 (*GABRG3*)	4F: GCTAACAGGGTCCTTAAAGCCA	102
		4R: GATCCAGATTCAGGCAGGCA	
	ChrX_CNV_558 (*IL1RAPL2*)	5F: TAATGGTAGGCCGTGTGTGC	100
		5R: ATTTGAGTACATTGGGCTGCT	
	Chr12_CNV_1028	6F: TGGTTCCACATTCCGTAG	105
		6R: TTCGTGTTGACTTCATTCA	
	Chr14_CNV_27	7F: AACTATTAGCAAGGTGAA	191
		7R: CCATCTGTATGTCTTCTT	
	Chr15_CNV_392	8F: CACTTGCTCCTCCTCCCTT	172
		8R: TGGGAGTCAGGGAAGGAAT	
	Chr20_CNV_2034	9F: CAACCTTAGAATCTGCGAGTTGC	100
		9R: AGGGGTGGAAGTATTGACTGG	
	Chr27_CNV_1684	10F: TCCCTGCTGAAGGGGAGAA	149
		10R: CCAGGTCTCTGAGCGGTTC	
	Chr2_CNV_2150 (*COL5A2*)	11F: AAGTGGTTGTCTTCTGTG	112
		11R: TTAAGGACTGCCATCTGA	
	Chr3_CNV_1815 (*LEPR*)	12F: TCACTCCAGATGCTCGCTTT	162
		12R: GCCAGGGTTACCACCGTTAG	
	Chr4_CNV_1934 (*SHH*)	13F: AAGCTGAACGCCTTAGCCAT	113
		13R: TCGTAGTGCAGCGACTCTTC	
	Chr7_CNV_161 (*SSBP2*)	14F: ATCAGCAGCAATCAAGTTC	169
		14R: AAGTCTCATCTGTGGTATCAT	
	Chr13_CNV_1410 (*SNTA1*)	15F: AATGCTGAATAACCAACAA	147
		15R: GCCTGTATTGCTATGAAC	
	Chr7_CNV_136	16F: TCTTCCACATACACATTCTTC	102
		16R: AGAGCAAGGTAGATTCCAT	
	Chr11_CNV_505	17F: CTGTTAGGTCCATATCAT	172
		17R: AAGAATAGCAAGAAGAGA	
	Chr13_CNV_1398	18F: CATAACATAAGAAACCGAGTA	195
		18R: GGTGTAAAGTTTGCTGAA	
	Chr20_CNV_2047	19F: TTCTCCATAGTGGCTGTA	181

		19R: CGGATTCCTGTTGATGTAT	
	Chr22_CNV_7	20F: AACAAGCGACTAAGCATA	134
		20R: GAAGCAACAGTTAGAATGG	
mRNA level	*LEPR*	21F: AAGAGTCCGGCGTACAGTGA	117
		21R: GGGGAGGGGATACCCTAGTTA	
	GAPDH	22F: AATGAAAGGGCCATCACCATC	204
		22R: GTGGTTCACGCCCATCACA	
